# Experimental and Theoretical Analysis of the Thiol-Promoted Fragmentation of 2-Halo-3-tosyl-oxanorbornadienes

**DOI:** 10.1021/acs.orglett.3c02548

**Published:** 2023-10-10

**Authors:** Marina Carranza, Ana T. Carmona, Claudio D. Navo, Inmaculada Robina, Simone Fratta, Carlos Newburn, Gonzalo Jiménez-Osés, Antonio J. Moreno-Vargas

**Affiliations:** † Departamento de Química Orgánica, Facultad de Química, Universidad de Sevilla, Sevilla, 41012, Spain; ‡ Center for Cooperative Research in Biosciences (CIC bioGUNE), Basque Research and Technology Alliance (BRTA), Bizkaia Technology Park, Building 800, 48160 Derio, Spain; § Ikerbasque, Basque Foundation for Science, 48013 Bilbao, Spain

## Abstract

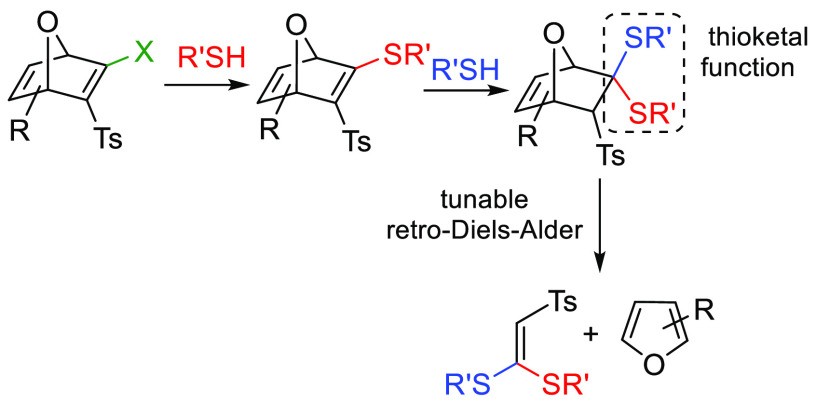

2-Halo-3-tosyl-oxanorbornadienes are able to accept two thiol molecules through an initial nucleophilic substitution, giving isolable oxabicyclic thiovinyl sulfones that, subsequently, can react with a second thiol molecule via thio-Michael addition. The resulting oxanorbornenic thioketals undergo retro-Diels–Alder (rDA) fragmentation to release a furan derivative and a ketene *S*,*S*-acetal. The substitution pattern of the oxanorbornadienic skeleton influences the rate of the rDA through electronic and steric factors examined by quantum mechanical calculations.

Cleavable linkers are valuable chemical tools for chemical biology with applications in drug development, proteomics, imaging, and DNA sequencing.^1^ In the particular case of antibody–drug conjugates (ADCs), this type of linkers is present in the majority of ADCs in clinical development.^2^ Chemical systems acting as cleavable linkers should be able to form a stable bond between the protein and the cytotoxic agent (payload) in the circulating macromolecule and to fragment as a response to an intracellular stimulus. Biological thiols such as glutathione (GSH), which is abundant in the cytosol,^3^ often play this role.^2b^ In this sense, Finn, Houk, and co-workers have recently explored the thiol-promoted fragmentation of electrophilic oxanorbornadienes (ONDs) in the search for thiol-responsive cleavable linkers for drug delivery systems (Scheme 1, previous ONDs).^4−6^ As a result, an exhaustive (theoretical and experimental) study of the influence of the substitution pattern of ONDs in the rate of fragmentation via retro-Diels–Alder (rDA) reaction was reported.^5,6^ Some of these ONDs were successfully applied in drug delivery systems.^7^ Bercovici and co-workers recently extended this chemistry of ONDs to ylidenenorbornadiene carboxylate analogues.^8^ From our side, we have recently reported that azanorbornadienes containing the bromovinyl sulfone functionality are useful reagents for the selective modification of proteins.^9^ This functionality is crucial for the bioconjugation process as it allows the reaction with the thiol function of cysteine residues of proteins through nucleophilic vinylic substitution (S_N_V_σ_). We report herein a new family of electrophilic ONDs containing a halovinyl sulfone/ester/phosphonate functionality that could be useful in linker chemistry (Scheme 1, this work). The novelty of our systems lies in their ability to accept two thiol molecules at different stages, the first one mimicking the Cys residue of a protein in the bioconjugation step and the second one the intracellular GSH for the cleavage of the linker. We present a detailed study of the reactivity of the new ONDs toward *N*-acetylcysteamine, as a simple model of a biological thiol. These studies allowed us to identify the most reactive system for the initial thiol-substitution and, additionally, the most sensitive one toward the further thiol-promoted fragmentation.

**Scheme 1:**
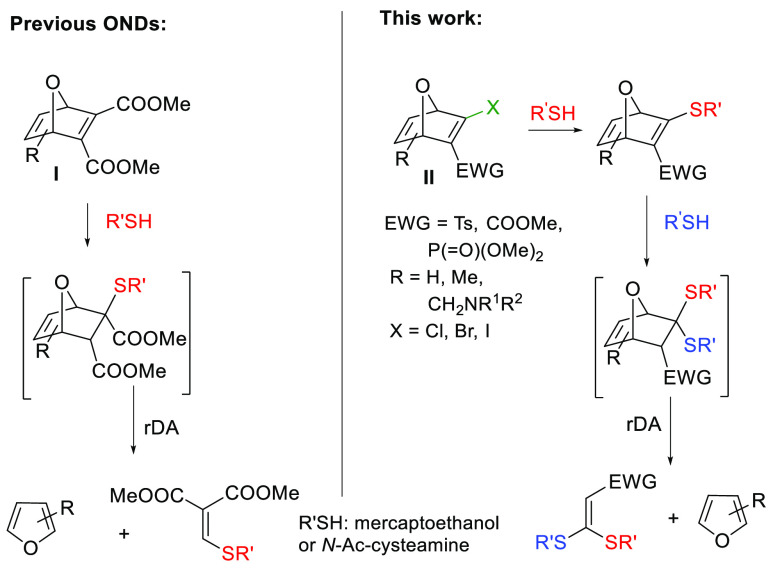
Thiol-Promoted Fragmentation of ONDs: New and Previous Strategies

First, we carried out the synthesis of a collection of halo-ONDs (Figure 1). These compounds were obtained by the Diels–Alder reaction of the corresponding furan and the adequate electron-deficient alkyne (see the Supporting Information for details).

**Figure 1:**
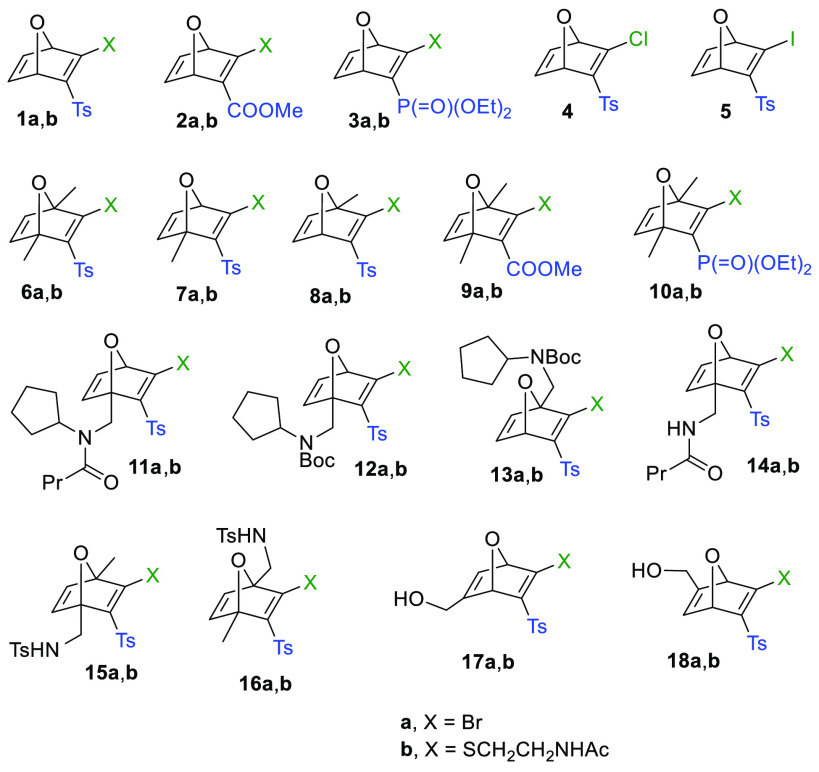
Structures of halo/thio-ONDs.

Special effort was paid to introduce an amino-functionalized substituent on the furan derivative that could act as a handle for future cargo-delivery purposes. Then, competition experiments between pairs of halo-OND systems and *N*-acetylcysteamine in DMF-phosphate buffer solution (PBS, pH 8.0) were performed (Table 1). The bromo-OND **1a** was chosen as a reference for the competition experiments, pairing this compound with a selection of representative halo-ONDs (**2a**, **3a**, **4**, **5** and **8a**, Figure 1). All the reactions yielded a mixture of stable 2-thio-oxanorbornadienic (thio-ONDs) derivatives (**1b**/**Bb**) in a fast (less than 15 min) and clean reaction (no byproducts were detected).

**Table 1:**
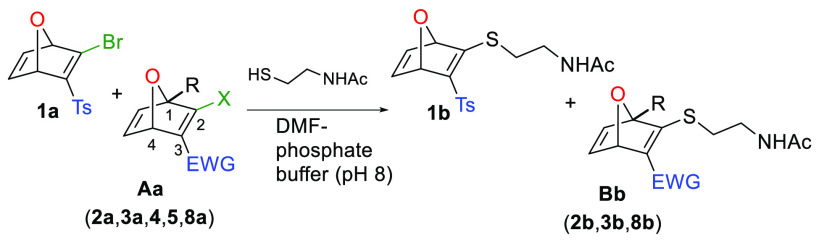
Competition Experiments: Reaction of a Mixture of Two Halo-ONDs with *N*-Acetylcysteamine^a^

Entry	Halo-ONDs	Structural variation	% Conversion into **1b**:**Bb**
1	**1a**:**2a**	EWG: Ts/COOMe	37:63
2	**1a**:**3a**	EWG: Ts/P(=O)OEt_2_	58:42
3	**1a**:**4**	X: Br/Cl	48:52
4	**1a**:**5**	X: Br/I	62:38
5	**1a**:**8a**	R: H/Me	54:46

a Reaction conditions: To an equimolar solution of ONDs **1a**/**Aa** (0.072–0.15 mM each, 1.0 equiv each) and *N*-acetylcysteamine (0.9 equiv) in DMF (2.0 mL), a similar volume of phosphate buffer solution (pH 8.0) was added. The reaction mixture was stirred for 30 min at room temperature. After workup, the ratio **1b**:**Bb** was analyzed by ^1^H NMR.

These experiments allowed us to establish some structure–reactivity relationships: (i) the use of a −COOMe group as an electron-withdrawing group accelerated the substitution reaction with respect to the tosyl or phosphonate group, the latter being the less reactive one (entries 1 and 2); (ii) chloro/bromo-oxanorbornadienes are more reactive than yodo-derivative in the S_N_V_σ_ (entries 3 and 4); (iii) the steric hindrance of a substituent at C1, close to the C2 electrophilic center, was not significant (entry 5).

Next, we explored the thiol-promoted fragmentation of selected thio-ONDs **1b**, **2b**, and **6b**–**18b** that were also obtained from bromo-OND precursors via nucleophilic displacement (Figure 1; see the Supporting Information for details). This fragmentation proceeded via a two-reaction sequence: a selective thio-Michael addition of *N*-acetylcysteamine at C2 of the thiovinyl sulfone/ester/phosphonate function of the oxanorbornadiene, followed by a retro-Diels–Alder reaction to afford a furan derivative and the corresponding ketene *S*,*S*-acetal **19** or **20** (Scheme 2). Scheme 2 shows the half-life (*t*_1/2_) for the transformation of the starting thio-OND into the final furan derivative. The parameter *t*_1/2_ was determined through ^1^H NMR by monitoring the concentration of the furan derivative vs time. In all the cases, except for thio-ONDs **8b** and **13b**,^10^ the thioketal intermediate was formed quantitatively and immediately (detected by ^1^H NMR). Thus, the comparative study of the structural effects of the bicyclic skeleton in this series will only reflect the influence on the rDA but not the conjugate addition. Electronic as well as steric substituent effects might be responsible for the different rates observed for the rDA reactions. We observed that the presence of an ester group instead of a tosyl at C3 (compounds **2b** and **9b**) remarkably slowed down the reaction rate (**1b** vs **2b**; **6b** vs **9b**). This could be explained considering that the tosyl group is more electron-withdrawing than the methoxycarbonyl, which could provide a stronger dienophilic character to the forming ketene *S*,*S*-acetal.^11^ This fact would help to improve the orbital interaction of this dienophile with the furanic diene, stabilizing the transition state of the rDA. This hypothesis is also plausible to explain the fact that OND-phosphonate **10b** is less reactive toward the thio-Michael addition and, additionally, the corresponding thioketal adduct does not fragment. The presence of Me substituents at the bridgehead positions (C1 and/or C4) accelerated the rDA reaction (**1b** vs **6b**, **7b**, **8b**; **2b** vs **9b**), as previously demonstrated by Finn, Houk, and co-workers in other oxanorbornene systems.^4−7^ As these authors indicated, the methyl group generally improves the electron-rich character of the forming furanic diene, which also enhances the interaction between orbitals of the diene and dienophile in the transition state. Surprisingly, a big difference was observed between regioisomers **7b** and **8b**. While the thioketal intermediate was detected by ^1^H NMR in the fragmentation of **7b**, only fragmented products and starting OND were observed when monitoring the fragmentation of regioisomer **8b**; a relatively short half-life (38 min) was measured for this compound. A similar behavior was observed for regioisomers **12b** and **13b**. While the thioketal intermediate is quantitatively and immediately formed for **12b**, the corresponding thioketal is not observed when the fragmentation of **13b** is monitored (*t*_1/2_ = 13 min). The different results obtained for the fragmentations of C4-functionalized derivatives **11b**, **12b**, and **14b**, in comparison with **7b**, reveal that the rate of the rDA is extremely sensitive to the topology/typology of the C4-substituent. In the particular case of **14b**, with a pendant secondary amide on C4, its long half-life might be in part explained by a stabilizing intramolecular H-bonding between the amide group and the sulfone on C3, despite the effect of intramolecular H-bonding being mitigated in polar protic solvents. We also examined the simultaneous introduction of a sulfonamide-functionalized substituent and a Me group at the bridgehead carbons (**15b** and **16b**). We observed that the presence of the sulfonamide substituent in C4 slowed down (5-fold) the fragmentation process (**15b** vs **8b**). Regioisomers **15b** and **16b** could be directly compared only in DMSO-*d*_6_,^12^ and no difference was observed in the fragmentation rate. This was also the case for the couple **17b**/**18b** with the presence of a substituent on C5/C6 combined with the absence of substituents at C1 and C4; a similar *t*_1/2_ value was observed for both.

**Scheme 2:**
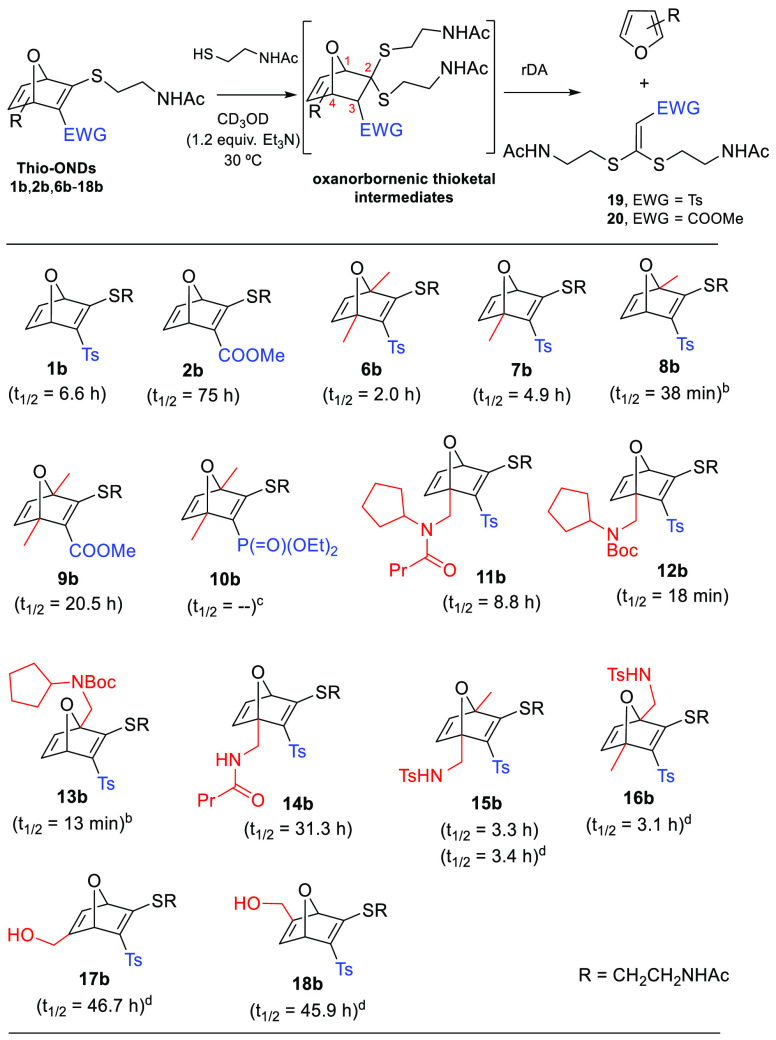
Half-Lives (*t*_1/2_) for the Fragmentation of Thio-ONDs Reaction conditions: To a solution of thio-OND in CD_3_OD (80 mM), a solution of *N*-acetyl cysteamine (1.1 equiv) and Et_3_N (1.2 equiv) in the same solvent was added (final concentration of thio-OND: 70 mM). The mixture was heated at 30 °C, and ^1^H NMR spectra were registered at different intervals; *t*_1/2_ (in h or min) was determined from the plot [concentration of the resulting furan] vs time. Thioketal intermediate was not detected. Slow thio-Michael addition (2 h) and rDA not observed. DMSO-*d*_6_ instead of CD_3_OD was used as solvent.

In order to explore the importance of the thioketal function in the intermediate for the fragmentation, the reactivity of vinyl sulfone OND **21** (Scheme 3) toward *N*-acetylcysteamine was studied. In contrast to the result obtained with **1b** (Scheme 2), the resulting thio-adduct **22** was stable and did not undergo rDA at 30 °C within a week. A similar result was obtained for oxanorbornenic ketal **23**,^13^ which proved to be stable to the fragmentation process.

**Scheme 3:**
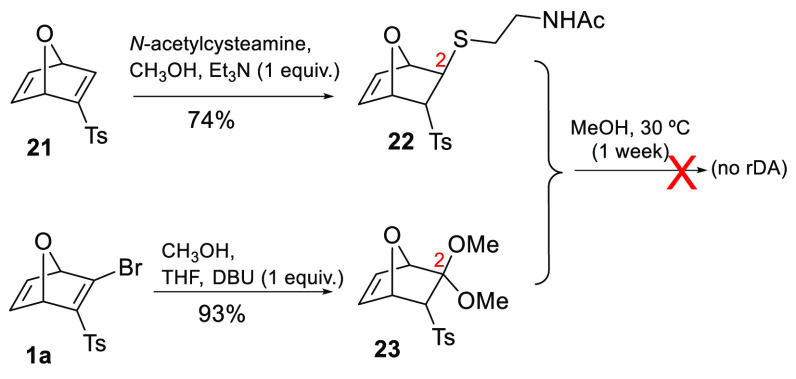
Studies on the Stabilities of Oxanorbornene Derivatives

The influence of the substitution patterns of the oxabicycle in the rDA reaction was analyzed by quantum mechanics (see the Supporting Information for full computational methods). Methylsulfonyl and methylthioether/ketal groups were used as models for the tosyl and reacting *N*-acetylcysteaminyl groups, respectively. First, the propensity of the parent Br-OND **1a** and thio-OND **1b** to undergo the rDA reaction was analyzed (see the Supporting Information). Reactions from both derivatives have quite high calculated activation energies (Δ*G*^⧧^ = 35.3 and 30.4 kcal mol^–1^, respectively) and are endergonic (Δ*G* = +9.9 and +4.9 kcal mol^–1^, respectively), in agreement with their lack of rDA reactivity observed experimentally. In agreement with previous studies performed by Finn, Houk, and co-workers for analogous systems,^4,6^ a moderate correlation was found between the calculated rDA activation barriers and the experimentally observed half-lives (Figure 2a). Indeed, this correlation was excellent within the subset of substrates bearing methyl groups at different positions of the bicyclic scaffold (Figure 2a, in orange). Likewise, a moderate linear correlation between reaction (Δ*G*_rxn_) and activation (Δ*G*^⧧^) energies with a slope of ∼0.5 was found for the calculated rDA reactions; this is in agreement with the Hammond postulate and Marcus theory which state that for similar reactions ΔΔ*G*^⧧^ ≈ ^1^/_2_ΔΔ*G*_rxn_.^14^ All the transition structures (TS) are concerted but asynchronous, with often a shorter distance for the breaking C3–C4 (1.96–2.16 Å) bond and a longer distance for the C1–C2 (2.10–2.37 Å) bond, with some exceptions (Figure 2b). Of note, the presence of a methyl group at the bridgehead position (C1) adjacent to the thioketal (**TS-8b**) significantly lowers the activation energy for the fragmentation reaction (Δ*G*^⧧^ = 21.4 vs 23.5 kcal mol^–1^ in unsubstituted **TS-1b**); this is likely due to the combined effect of the electron-donating character and the steric hindrance exerted by the methyl group. In fact, the TS for this rDA reaction is the most asynchronous of all of the calculated ones. On the contrary, the presence of a methyl group in the opposite and less sterically hindered bridgehead position (C4) inverts the polarization of the forming furanic diene in **TS-7b**, leading to an inversion of the asynchronicity at the TS and exerting a smaller activating effect (Δ*G*^⧧^ = 23.0 kcal mol^–1^). As expected, such effects are counterbalanced in C1,C4-dimethylated **TS-6b**, both geometrically and energetically (Δ*G*^⧧^ = 22.5 kcal mol^–1^). Somewhat counterintuitively, and in agreement with the experiments, substitutions with electron-donating methyl groups at C5/C6 positions of the forming diene have a destabilizing effect (Δ*G*^⧧^ = 24.7 kcal mol^–1^ for both **TS-17b′** and **TS-18b′**),^15^ although this is not translated into significant changes in the breaking C–C bond distances.

**Figure 2:**
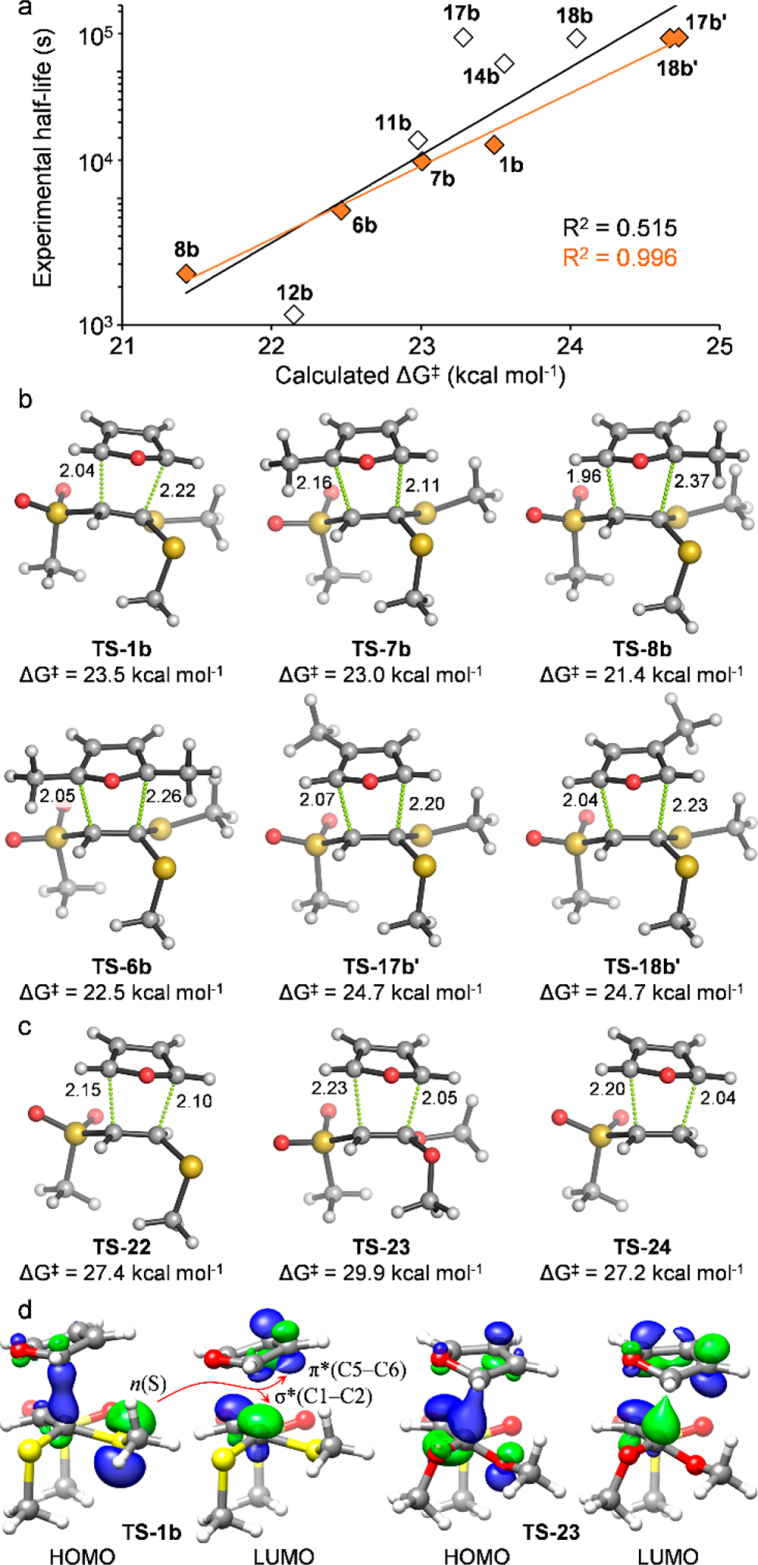
Computational studies on rDA reactions. (a) Observed half-life (*t*_1/2_) vs calculated activation energies (Δ*G*^⧧^) for model retro-Diels–Alder (rDA) reactions. (b, c) Transition structures and calculated activation energies for rDA from selected di-thio-ONDs, thioether **22**, ketal **23**, and unsubstituted alkene **24**. (d) HOMO and LUMO in **TS-1b** and **TS-23** (side view); potentially stabilizing orbital interactions are highlighted with red arrows. Calculations were performed at the PCM(MeOH)/M06-2X/6-311+G(d,p) level. Distances are given in angstroms.

We also analyzed the triggering effect on the rDA reaction observed for the parent OND bearing a thioketal (**TS-1b**) versus those featuring a thioether (**TS-22**), a ketal (**TS-23**), or no substituents at C2 (**TS-24**) (Figure 2c). While the four transition structures are concerted and asynchronous, those lacking the two geminal sulfur atoms (**TS-22**, **TS-23**, and **TS-24**) show an opposite asynchronicity trend: the breaking C1–C2 bonds are shorter (2.04–2.10 Å) than the breaking C3–C4 bonds (2.15–2.23 Å). Of note, the rDA reaction in these cases, especially from ketal **23**, have much higher activation energies (Δ*G*^⧧^ ≈ 27–30 kcal mol^–1^) in agreement with the experiments (i.e., no reaction is observed from **22** or **23**, Scheme 3). The unusual effect of the thioketal derived from OND **1b** might be attributed to a higher fragmentation propensity resulting from their bulkier nature and the interactions between the S lone pairs (*n* orbitals) and the antibonding σ*(C1–C2) and/or π*(C5–C6) orbitals (see HOMO and LUMO in Figure 2d); of note, the C–C breaking bonds are slightly elongated (i.e., distorted) in thioketal derived from **1b** (∼1.58 Å) compared to its ketal counterpart **23**. The smaller size and higher electronegativity of the oxygen atoms in **23** might prevent the interaction of the O lone pairs and the antibonding π*(C5–C6) orbitals, resulting in a lower interaction energy (as shown by a distortion/interaction analysis of the reaction; see the Supporting Information) and translating into a higher activation energy.

In conclusion, electrophilic halo-oxanorbornadienic systems able to accept two thiol molecules at different stages have been developed. The second thiol molecule triggers the fragmentation of the OND system through conjugate addition, followed by a rDA reaction. The substitution pattern of the OND skeleton was crucial for the modulation of the fragmentation process, with a broad range of half-lives from 2 days to 13 min under rDA conditions. Part of the developed systems contain an amino-functionalized substituent that can act as a handle for the incorporation of biologically relevant molecules. These features make these ONDs amenable to thiol-sensitive linker chemistry in drug-delivery systems.

## Data Availability

The data underlying this study are available in the published article and its Supporting Information.

## Supporting Information Available

Synthetic procedures, characterization data, computational details, and NMR spectra for the new compounds (PDF)
