# Supplementary material for: Experimental and Theoretical Analysis of the Thiol-Promoted Fragmentation of 2-Halo-3-tosyl-oxanorbornadienes
Source: Org Lett. 2023 Oct 10;25(41):7481–5. doi: 10.1021/acs.orglett.3c02548 (PMC10594659; doi:10.1021/acs.orglett.3c02548)
Supplement: Supplementary file 1 — ol3c02548_si_001.pdf [file ol3c02548_si_001.pdf]

SUPPORTING INFORMATION

Experimental and theoretical analysis of the thiol-promoted fragmentation of 2-halo-3-tosyl-oxanorbornadienes

Marina Carranza,[a] Ana T. Carmona,[a] Claudio D. Navo,[b] Inmaculada Robina,[a] Simone Fratta,[a] Carlos Newburn,[a] Gonzalo Jiménez-Osés[b,c,*] and Antonio J. Moreno-Vargas[a,*]

[a] *Departamento de Química Orgánica, Facultad de Química, Universidad de Sevilla, Sevilla, 41012, Spain.*

[b] *Center for Cooperative Research in Biosciences (CIC bioGUNE), Basque Research and Technology Alliance (BRTA), Bizkaia Technology Park, Building 800, 48160 Derio, Spain*

[c] *Ikerbasque, Basque Foundation for Science, 48013 Bilbao, Spain*

TABLE OF CONTENTS

1. Synthesis of activated alkynes **S1-S5**. S2

2. Synthesis of furan derivatives **S6-S9**. S2

3. Diels-Alder (DA) reaction for the preparation of halo-ONDs **1a-3a**, **4**, **5**, **6a-18a**. S4

4. Synthesis of thio-oxanorbornadienes (thio-ONDs) **1b-3b**, **6b-18b**. S10

5. Competition experiments. S17

6. Studies of the fragmentation of thio-ONDs via ^{1}H-NMR. Selected examples. S22

7. Quantum mechanical calculations. S42

8. ^{1}H and ^{13}C-NMR spectra for new compounds. S63

9. References. S94

General methods

^{1}H- and ^{13}C-NMR spectra were recorded with a Bruker AVIII300, NEO300, NEO400, NEO500 and Spinsolve80 spectrometer for solutions in $CDCl_3$, CD_3OD, DMSO-d_6, and C_6D_6. δ are given in ppm and *J* in Hz. Chemical shifts are calibrated using residual solvent signals. All the assignments were confirmed by 2D spectra (COSY and HSCQ). High resolution mass spectra were recorded on a Q-Exactive-quadrupole mass spectrometer. TLC was performed on silica gel 60 F_{254} (Merck), with detection by UV light charring with $KMnO_4$, ninhydrin, or with reagent [$(NH_4)_6MoO_4$, $Ce(SO_4)_2$, H_2SO_4, H_2O]. Purification by silica gel chromatography was carried out using either hand-packed glass columns (Silica gel 60 Merck, 40-60 and 63-200 µm) or Puriflash XS520 Plus Interchim system with prepacked cartridges.

1. Synthesis of activated alkynes S1-S5.

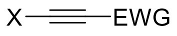

S1; X = Br, EWG = Ts
S2; X = Br, EWG = COOMe
S3; X = Cl, EWG = Ts
S4; X = I, EWG = Ts
S5; X = Br, EWG = $P(=O)(OMe)_2$

- Alkyne **S1** was prepared according to the procedure previously described by Trudell and coworkers.[1]

- Alkyne **S2** was prepared according to the procedure previously described by Leroy.[2]

- Alkyne **S3** was prepared according to the procedure previously described by Jørgensen and coworkers.[3]

- Alkyne **S4** was prepared according to the procedure previously described by us.[4]

- Alkyne **S5** was prepared according to the procedure previously described.[5]

2. Synthesis of furan derivatives S6-S9

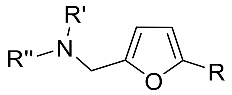

S6; R = H, R' = cyclopentyl, R'' = Boc
S7; R = Me, R' = H, R'' = Ts
S8; R= H, R'=H, R''= butanoyl

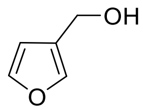

S9

***Tert*-Butyl cyclopentyl(furan-2-ylmethyl)carbamate (S6)**

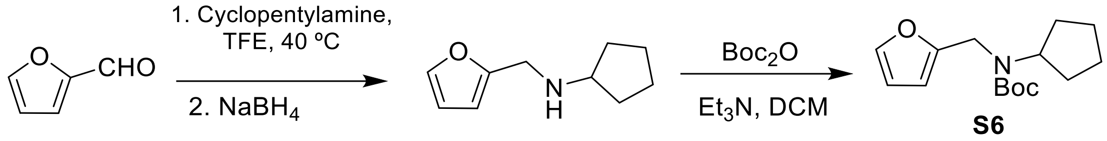

A solution of commercial furfural (1.3 mL, 15.6 mmol) in 2,2,2-trifluoroethanol (14 mL) was stirred at 40 °C for 5 min., then commercial cyclopentylamine (1.4 mL, 14.2 mmol) was added and the rection mixture was stirred for 30 min at 40 °C (heat-on block system). After this time, $NaBH_4$ (1.10 g, 28.4 mmol) was added, and the reaction was stirred vigorously for 1 h at 40 °C. Then, the mixture was filtered through celite and the residue was washed with abs EtOH (30 mL), and the dissolvent was evaporated to yield *N*-(furan-2-ylmethyl) cyclopentamine as a brown oil, which was used without further purification in the following step. This amine (2.3 g, 14.2 mmol) was dissolved in DCM (30 mL) and Boc_2O (9.3 g, 42.6 mmol) and Et_3N (2.4 mL, 17 mmol) were added. The reaction was stirred for 3 h at r.t and then the mixture was washed with brine (3x15 mL), and the organic phase was dried with anh. Na_2SO_4, filtered, and concentrated. The residue was purified by column chromatography on silica gel (Et_2O: CyHex 1:15 → 1:8) to yield **S6** (2.94 g, 11.1 mmol, 78 %) as pale-yellow oil. ^1H NMR (300 MHz, $CDCl_3$, δ ppm): δ 7.27 (d, 1H, *J*=1.0 Hz, H-5), 6.26 (m, 1H, H-4), 6.11 (d, 1H, *J*= 2.8 Hz, H-3), 4.27 (s, 3H, CH_2, CH cyclopentyl), 1.80-1.46 (m, 17H, CH_2 cyclopentyl, CH_3 of Boc). ^{13}C NMR (75.4 MHz, $CDCl_3$, δ ppm, mixture of rotamers): δ 155.4 (C=O), 153.4 (C=O), 146.6 (C-2), 140.9 (C-5), 110.2 (C-4), 106.4 (C-3), 79.5 (C_q of Boc), 57.0 (CH of cyclopentyl), 40.8 (CH_2), 29.2 (CH_2 of cyclopentyl), 28.3 (CH_2 of cyclopentyl), 27.3 (CH_2 of cyclopentyl), 23.6 (CH_3 of Boc). HRMS (ESI) m/z: found, 288.1575; calcd. for $C_{15}H_{23}O_3NNa$ $[M+Na]^+$: 288.1576.

-*N*-(Furan-2-ylmethyl)-4-methylbenzenesulfonamide (**S7**) was prepared according to the procedure described by Hashmi and coworkers.[6]

- *N*-(Furan-2-ylmethyl)butyramide (**S8**).[7]

- Furan-3-ylmethanol (**S9**) was prepared according to the procedure described by Peng Xu and coworkers.[8]

3. Diels-Alder (DA) reaction for the preparation of halo-ONDs 1a-3a, 4, 5, 6a-18a.

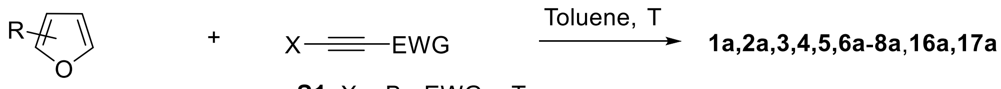

furan
2-methylfuran
2,5-dimethylfuran

S1; X = Br, EWG = Ts
S2; X = Br, EWG = COOMe
S3; X = Cl, EWG = Ts
S4; X = I, EWG = Ts
S5; X = Br, EWG = P(=O)(OMe)$_2$

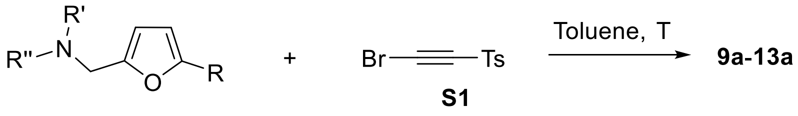

S6; R = H, R' = cyclopentyl, R'' = Boc
S7; R = Me, R' = H, R'' = Ts
S8; R= H, R'=H, R''= butanoyl

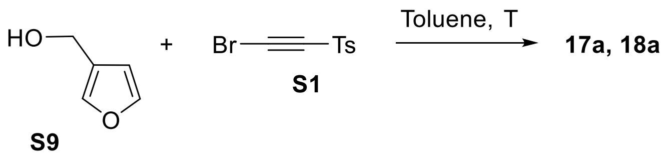

S9

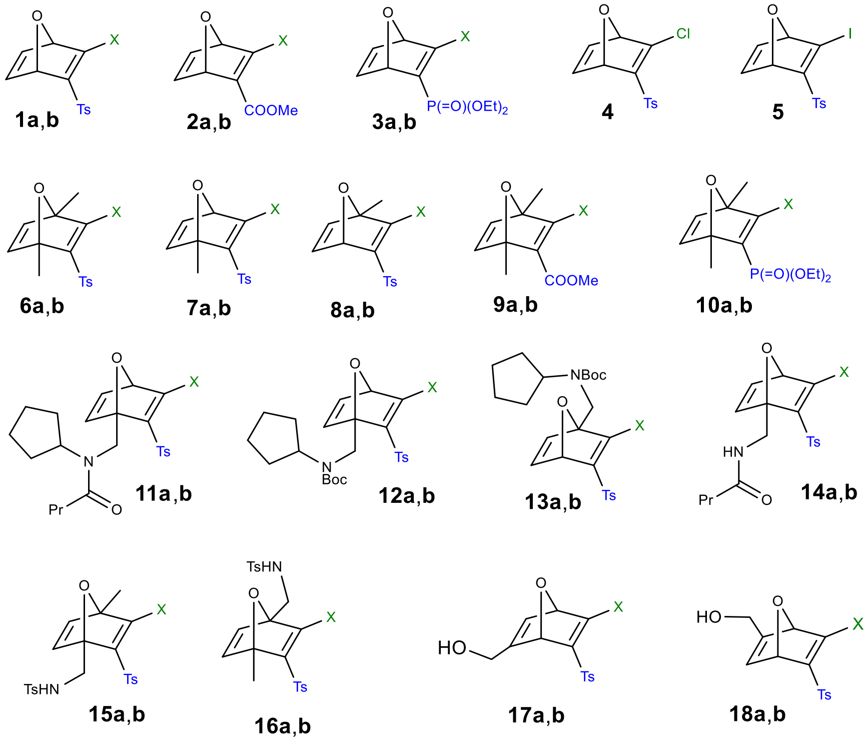

a, X = Br
b, X = SCH_2CH_2NHAc

- Halo-ONDs **1a**-**3a**, **4**, **5** and **10a** were prepared as previously described.[9]

*General procedure for the preparation of halo-ONDs **6a**-**9a**, **11a**-**18a** via DA.*

To a solution of activated alkyne **S1**-**S5** (x mmol) in toluene (2 mL/mmol), the corresponding commercial or synthetic furan derivative (z mmol) was added, and the reaction mixture was stirred at 45-55 °C (heat-on block system). After the reaction was completed, the solvent was evaporated, and the resulting residue was purified by a chromatography column on silica gel to give the corresponding halo-OND. When two regioisomers were expected, NOE experiments were performed for structure elucidation.

***rac*-2-Bromo-1,4-dimethyl-3-tosyl-7-oxabicyclo [2.2.1]hepta-2,5-diene (6a)**

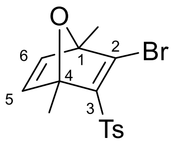

Reaction of 2,5-dimethylfuran (0.25 mL, 2.32 mmol) and alkyne **S1** (200 mg, 0.77 mmol) following the general procedure (55 °C, 6 h), afforded after chromatographic purification (EtOAc:CyHex 1:6) compound **6a** (242 mg, 89 %) as a yellow oil. ^{1}H NMR (300 MHz, $CDCl_3$, δ ppm): δ 7.73 (d, 2H, *J*= 8.5 Hz, Ar-H), 7.33 (d, 2H, *J*= 8.0 Hz, Ar-H), 6.72 (d, 2H, H-5, H-6), 2.43 (s, 3H, CH_3 of Ts), 1.75 (s, 3H, CH_3), 1.68 (s, 3H, CH_3). ^{13}C NMR (75.4 MHz, $CDCl_3$, δ ppm): δ 152.7, 151.5 (C-2, C-3), 147.7 (C-5 or C-6), 145.0 (C_qAr), 144.2 (C-5 or C-6), 136.6 (C_qAr), 129.9 (C-Ar), 127.7 (C-Ar), 94.4, 93.7 (C-1, C-4), 21.7 (CH_3 of Ts), 16.5 (CH_3), 15.5 (CH_3). HRMS (ESI) m/z: found, 354.9995; calcd. for $C_{15}H_{16}{}^{79}BrO_3S$ $[M+H]^+$: 355.0004.

***rac*-3-Bromo-1-methyl-2-tosyl-7-oxabicyclo [2.2.1] hepta-2,5-diene (7a) and (*rac*)-2-bromo-1-methyl-3-tosyl-7-oxabicyclo [2.2.1] hepta-2,5-diene (8a)**

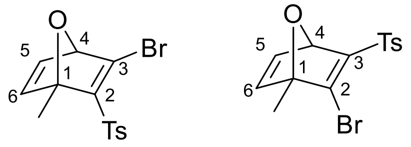

7a **8a**

Reaction of 2-methylfuran (0.7 mL, 7.72 mmol) and alkyne **S1** (500 mg, 1.92 mmol) following the general procedure (50 °C, 9 h), afforded after chromatographic purification (Et_2O: CyHex 1:5) compounds **7a** (344 mg, 53 %) and **8a** (234 mg, 36%) both as yellow oils. Data for **7a**: ^{1}H NMR (300 MHz, $CDCl_3$, δ ppm): δ 7.76 (d, 2H, *J*= 7.5 Hz, Ar-H), 7.35 (d, 2H, *J*= 7.5 Hz, Ar-H), 7.01 (dd, 1H, *J*= 5.5 Hz, *J*= 1.6 Hz, H-5), 6.77 (d, 1H, *J*= 5.5 Hz, H-6), 5.51 (d, 1H, *J*= 1.6 Hz, H-4), 2.45 (s, 3H, CH_3 of Ts),

1.71 (s, 3H, CH_3). ^{13}C NMR (75.4 MHz, $CDCl_3$, δ ppm): δ 151.5, 150.0 (C-2, C-3), 145.2 (C_qAr), 144.65 (C-5), 143.5 (C-6), 136.2 (C_qAr), 130.0 (C-Ar), 127.7 (C-Ar), 96.3 (C-1), 84.3 (C-4), 21.7 (CH_3 of Ts), 15.4 (CH_3). Data for **8a**: ^1H NMR (300 MHz, $CDCl_3$, δ ppm): δ 7.73 (d, 2H, *J*=8.1 Hz, Ar-H), 7.33 (d, 2H, *J*=8.1 Hz, Ar-H), 7.03 (dd, 1H, *J*=5.2 Hz, *J*= 2.0 Hz, H-5), 6.74 (d, 1H, *J*=5.2 Hz, H-6), 5.22 (d, 1H, *J*=2.0 Hz, H-4), 2.44 (s, 3H, CH_3 of Ts), 1.78 (s, 3H, CH_3). ^{13}C NMR (75.4 MHz, $CDCl_3$, δ ppm): δ 150.6, 148.7 (C-2, C-3), 146.8 (C-6), 145.1 (C_qAr), 141.0 (C-5), 136.6 (C_qAr), 130.0 (C-Ar), 127.7 (C-Ar), 94.7 (C-1), 89.0 (C-4), 21.7 (CH_3 of Ts), 16.4 (CH_3). HRMS (ESI) m/z: found, 340.9840; calcd. for $C_{14}H_{14}O_3{}^{79}BrS$ $[M+H]^+$: 340.9847.

(*rac*)-Methyl-3-bromo-1,4-dimethyl-7-oxabicyclo[2.2.1]hepta-2,5-diene-2-carboxylate (9a)

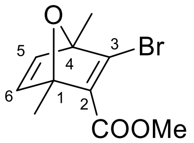

Reaction of 2,5-dimethylfuran (1.4 mL, 13.0 mmol) and alkyne **S2** (751 mg, 4.63 mmol) following the general procedure (50 °C, 28 h), afforded after chromatographic purification (Et_2O: CyHex 1:15) compound **9a** (418 mg, 35 %) as a brown oil. ^1H NMR (300 MHz, $CDCl_3$, δ ppm): δ 6.97 (d, 1H, *J*= 5.0 Hz), 6.87 (d, 1H, *J*= 5.0 Hz), 3.81 (s, 3H), 1.86 (s, 3H), 1.72 (s, 3H). ^{13}C NMR (75.4 MHz, $CDCl_3$, δ ppm): δ 163.6, 153.7, 148.0, 145.6, 145.3, 93.3, 92.8, 51.6, 16.5, 15.6. HRMS (ESI) m/z: found, 280.9789; calcd. for $C_{10}H_{11}O_3{}^{79}BrNa$ $[M+Na]^+$: 280.9789.

(*rac*)-*tert*-Butyl (3-bromo-2-tosyl-7-oxabicyclo[2.2.1]hepta-2,5-dien-1-yl)methyl)(cyclopentyl)carbamate (12a) and (*rac*)-*tert*-Butyl (2-bromo-3-tosyl-7-oxabicyclo[2.2.1]hepta-2,5-dien-1-yl)methyl)(cyclopentyl)carbamate (13a)

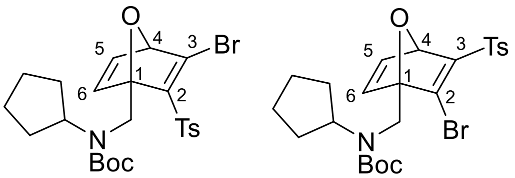

12a **13a**

Reaction of diene **S6** (1.98 g, 3.77mmol) and alkyne **S1** (751 mg, 2.90 mmol) following the general procedure (45 °C, 24 h), afforded after chromatographic purification (Et_2O:CyHex 1:3→1:1) compounds **12a** (838 mg, 55%) and **13a** (452 mg, 30%) as yellow solids. Data for **12a**: ^1H NMR (300 MHz, $CDCl_3$, δ ppm, mixture of rotamers): δ 7.78 (d, 2H, *J* = 8.0 Hz, Ar-H), 7.34 (d, 2H, *J* = 8.0 Hz, Ar-H), 6.97 (d, 1H, *J* = 5.2 Hz, H-6), 6.85 (br s, 1H, H-5), 5.25 (d, 1H, *J* = 1.9 Hz, H-4), 4.91 (br d, 1H, *J* =

12.0 Hz, CH_2), 3.80-3.68 (m, 1H, CH of cyclopentyl), 3.46 (d, 1H, *J* = 16.0 Hz, CH_2), 2.44 (s, 3H, CH_3 of Ts), 1.73 – 1.62 (m, 8H, CH_2 of cyclopentyl), 1.47 (s, 9H, CH_3 of Boc). ^{13}C NMR (75.4 MHz, $CDCl_3$, δ ppm, mixture of rotamers): δ 150.3 (C=O of Boc), 149.6 (C-2 or C-3), 149.5 (C-2 or C-3), 145.3 (C-5), 139.6 (C-6) 129.9 (C-Ar), 127.7 (C-Ar), 98.6 (C-1), 88.9 (C-4), 79.8 (C_q of Boc), 60.7 (CH of cyclopentyl), 46.7 (CH_2), 29.4 (CH_3 of Boc), 24.0 (CH_2 of cyclopentyl), 23.9 (CH_2 of cyclopentyl), 21.7 (CH_3 of Ts). Data for **13a**: ^{1}H NMR (300 MHz, $CDCl_3$, δ ppm, mixture of rotamers): δ 7.76 (d, 2H, *J* = 8.2 Hz, Ar-H), 7.34 (d, 2H, *J* = 8.2 Hz, Ar-H), 6.97–6.93 (m, 2H, H-5, H-6), 5.54 (s, 1H, H-4), 4.62 (br d, 1H, *J* = 15.8 Hz, CH_2), 3.83 (br s, 1H, CH of cyclopentyl), 3.35 (d, 1H, *J* = 16.5 Hz, CH_2), 2.44 (s, 3H, CH_3 of Ts), 1.73-1.50 (m, 8H, CH_2 of cyclopentyl), 1.46 (s, 9H, CH_3 of Boc). ^{13}C NMR (75.4 MHz, $CDCl_3$, δ ppm, mixture of rotamers): δ 157.3 (C=O), 147.3, 145.5 (C-2, C-3), 145.3, 141.7 (C-5, C-6), 136.0 (C_qAr), 130.0 (C-Ar), 127.8 (C-Ar), 127.6 (C_qAr), 99.3 (C-1), 84.3 (C-4), 80.2 (C_q of Boc), 59.8 (CH of cyclopentyl), 45.1 (CH_2), 29.7 (CH_3 of Boc), 28.5 (CH_2 of cyclopentyl), 23.8 (CH_2 of cyclopentyl), 21.7 (CH_3 of Ts). HRMS (ESI) m/z: found, 546.0916; calcd for $C_{24}H_{30}{}^{79}BrNNaO_5S$ $[M+Na]^+$: 546.0926.

***N*-(*rac*)-3-Bromo-2-tosyl-7-oxabicyclo[2.2.1]hepta-2,5-dien-1-yl)methyl)butyramide (14a)**

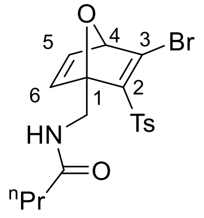

Reaction of diene **S8** (290 mg, 1.73 mmol) and alkyne **S1** (674 mg, 2.6 mmol) following the general procedure (50 °C, 15 h), afforded after chromatographic purification (AcOEt: CyHex 1:2→2:1) compound **14a** (618 mg, 84%) as a yellow solid. ^{1}HNMR (300 MHz, $CDCl_3$, δ ppm): δ 7.73 (d, 2H, *J*= 8.3 Hz, Ar-H), 7.36 (d, 2H, *J*= 8.6 Hz, Ar-H), 6.93 (dd, 1H, *J*= 5.2 Hz, *J*= 1.8 Hz, H-5), 6.62 (d, 1H, *J*= 5.2 Hz, H-6), 6.09 (br s, 1H, NH), 5.30 (d, 1H, *J*= 1.9 Hz, H-4), 4.58, 4.55 (2d, 1H, *J*= 15.0, CH_2), 3.66, 3.64 (2d, 1H, *J*= 15.0, CH_2), 2.45 (s, 3H, CH_3 of Ts), 2.15 (t, 2H, *J*= 7.5 Hz, $C\underline{H_2}$-CH_2-CH_3), 1.64 (m, 2H, CH_2-$C\underline{H_2}$-CH_3), 0.93 (t, 3H, *J*= 7.4 Hz, CH_2-CH_2-$C\underline{H_3}$). ^{13}C NMR (75.4 MHz, $CDCl_3$, δ ppm): δ 173.3 (C=O), 149.4, 149.3 (C-2, C-3), 145.8 (C_qAr), 143.9 (C-6), 140.8 (C-5), 135.3 (C_qAr), 130.3 (C-Ar), 127.9 (C-Ar), 97.5 (C-1), 89.3 (C-4), 38.8 ($\underline{C}H_2$-CH_2-CH_3), 38.3 (CH_2), 21.9 (CH_3 of Ts), 19.2 (CH_2-$\underline{C}H_2$-CH_3), 13.9 (CH_2-CH_2-$\underline{C}H_3$). HRMS (ESI) m/z: found, 448.0189; calcd. for $C_{18}H_{20}{}^{79}BrNNaO_4S$ $[M+Na]^+$: 448.0194.

N-(*rac*)-3-Bromo-4-methyl-2-tosyl-7-oxabicyclo[2.2.1]hepta-2,5-dien-1-yl)methyl)-4-methylbenzenesulfonamide (15a) and N-(*rac*)-2-bromo-4-methyl-3-tosyl-7-oxabicyclo[2.2.1]hepta-2,5-dien-1-yl)methyl)-4-methylbenzenesulfonamide (16a)

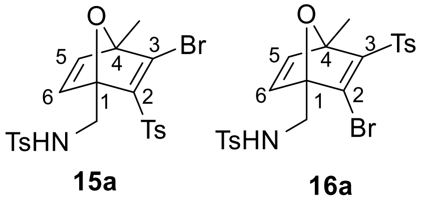

Reaction of diene **S7** (680 mg, 2.56 mmol) and alkyne **S1** (443 mg, 1.71 mmol) following the general procedure (50 °C, 15 h), afforded after chromatographic purification (Et_2O: CyHex 1:6→1:2) compounds **15a** and **16a** (750 mg, 84%, ratio 4.5:1) as a white solid. Data for **15a**: ^{1}H NMR (300 MHz, $CDCl_3$, δ ppm): δ 7.77-7.65 (m, 4H, Ar-H), 7.35-7.31 (m, 4H, Ar-H), 6.75 (d, 1H, *J*= 5.6 Hz, H-5), 6.63 (d, 1H, *J*= 5.4 Hz, H-6), 5.10 (dd, 1H, *J* = 8.8, 4.5 Hz, NH), 3.92 (dd, 1H, *J*= 14.0, 9.1 Hz, CH_2), 3.56 (dd, 1H, *J*= 14.0, 4.5 Hz, CH_2), 2.44 (s, 6H, CH_3 of Ts), 1.60 (s, 3H, CH_3). ^{13}C NMR (75.4 MHz, $CDCl_3$, δ ppm): δ 148.8, 146.2, 145.4, 143.7, 136.3, 130.1, 129.9, 127.7, 127.6, 127.2, 94.7, 94.2, 93.6, 42.6, 21.8, 21.6, 16.2. Data for **16a:** ^{1}H NMR (300 MHz, $CDCl_3$, δ ppm): δ 7.77-7.65 (m, 4H, Ar-H), 7.35-7.31 (m, 4H, Ar-H), 6.71 (d, 1H, *J*= 5.5 Hz, H-5), 6.63 (d, 1H, *J*= 5.4 Hz, H-6), 4.65 (t, 1H, *J*= 5.8 Hz, NH), 3.67 (dd, 1H, *J*=13.7, 6.8 Hz, CH_2), 3.51 (dd, 1H, *J*= 13.7, 5.5 Hz, CH_2), 2.44 (s, 6H, CH_3 of Ts), 1.60 (s, 3H, CH_3). ^{13}C NMR (75.4 MHz, $CDCl_3$, δ ppm): δ 148.6, 146.2, 144.9, 143.6, 136.3, 135.6, 130.1, 129.9, 127.7, 127.2, 94.7, 94.2, 42.0, 21.8, 21.6, 14.2.

((*rac*)-5-Bromo-6-tosyl-7-oxabicyclo[2.2.1]hepta-2,5-dien-2-yl)methanol (17a) and ((*rac*)-6-bromo-5-tosyl-7-oxabicyclo[2.2.1]hepta-2,5-dien-2-yl)metanol (18a)

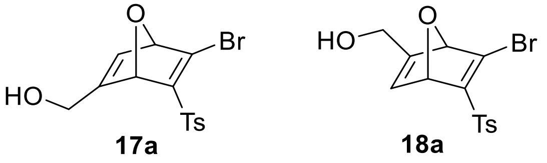

Reaction of diene **S9** (650 mg, 6.62 mmol) and alkyne **S1** (685 mg, 2.65 mmol) following the general procedure (45 °C, 7 h), afforded after chromatographic purification (Et_2O:CyHex 1:2→3:1) compounds **17a** and **18a** (717 mg, 76 %, ratio 2:1) as a brown oil. Data for **17a**: ^{1}H NMR (300 MHz, $CDCl_3$, δ ppm): δ 7.83 (d, 2H, *J*= 8.4 Hz, Ar-H), 7.37 (d, 2H, *J*= 8.0 Hz, Ar-H), 6.84-6.82 (m, 1H, H-3), 5.46 (d, 1H, *J*= 1.4 Hz, H-4), 5.30- 5.29 (m, 1H, H-1), 4.41 (br s, 2H, CH_2), 2.45 (s, 3H, CH_3 of Ts), 1.68

(br s, 1H, OH). ^{13}C NMR (75 MHz, $CDCl_3$, δ ppm): δ 159.5, 150.2, 146.9, 145.6, 136.4, 133.0, 130.3, 127.9, 90.9, 85.9, 59.1, 21.8. Data for **18a**: ^{1}H NMR (300 MHz, $CDCl_3$, δ ppm, *J* Hz): δ 7.79 (d, 2H, *J*= 8.3 Hz, Ar-H), 7.36 (d, 2H, *J*= 8.2 Hz, Ar-H), 6.77-6.75 (m, 1H, H-3), 5.53 (t, 1H, *J*= 1.6 Hz, H-4), 5.30-5.29 (m, 1H, H-1), 4.39 (br s, 2H, CH_2), 2.45 (s, 3H, CH_3 of Ts), 2.22 (br s, 1H, OH). ^{13}C NMR (75 MHz, $CDCl_3$, δ ppm): δ 156.3, 151.2, 145.4, 145.3, 136.3, 133.0, 130.2, 127.7, 91.2, 85.9, 59.1, 21.8. HRMS (ESI) m/z: found, 378.9602; calcd. for $C_{14}H_{13}{}^{79}BrNaO_4S$ $[M+Na]^+$: 378.9616.

***N*-(((*rac*)-3-Bromo-2-tosyl-7-oxabicyclo[2.2.1]hepta-2,5-dien-1-yl)methyl)-*N*-cyclopentylbutyramide (11a)**

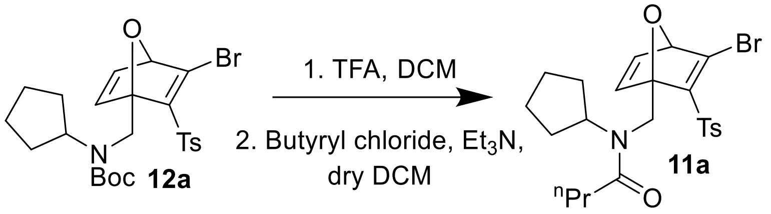

A solution of compound **12a** (175 mg, 0.33 mmol) in DCM (4 mL), was added TFA (0.32 mL, 4.2 mmol) and the mixture was stirred at r.t for 30 min. Then, TFA and DCM were evaporated to afford the corresponding unprotected amine that was used without further purification in the following step. This amine was dissolved in anh. DCM (5 mL), cooled to 0 °C and butyryl chloride (220 µL, 2.10 mmol) and Et_3N (295 µL, 2.10 mmol) were added under Ar. The mixture was stirred for 1.5 h at r.t. and then it was diluted with DCM and washed with 1M HCl and brine. The organic phase was dried (Na_2SO_4), filtered and concentrated. The residue was purified by column chromatography on silica gel (EtOAc:CyHex 1:3) to yield compound **11a** (86.5 mg, 0.17 mmol, 53 %) as a yellow oil. ^{1}H NMR (300 MHz, $CDCl_3$, δ ppm, mixture of rotamers): δ 7.80 (d, 2H, *J*= 8.1 Hz, Ar-H), 7.34 (d, 2H, *J*= 8.2 Hz, Ar-H), 6.88 (d, 1H, *J*= 5.2 Hz, H-6), 6.67 (br d, 1H, H-5), 5.19 (s, 1H, H-4), 5.07 (d, 1H, *J*= 15.4 Hz, CH_2), 4.05-4.01 (m, 1H, CH of cyclopentyl), 3.48 (d, 1H, *J*= 15.4 Hz, CH_2), 2.41 (s, 3H, CH_3 of Ts), 2.36-2.32 (m, 2H, C<u>H</u>$\underline{H_2}$-CH_2-CH_3), 1.78–1.49 (m, 10H, CH_2 of cyclopentyl, CH_2-C$\underline{H_2}$-CH_3), 0.91 (t, 3H, *J*= 7.3 Hz, CH_2-CH_2-C$\underline{H_3}$). ^{13}C NMR (75.4 MHz, $CDCl_3$, δ ppm, mixture of rotamers): δ 170.5 (C=O), 150.3, 149.3 (C-2, C-3), 146.1 (C-6), 145.0 (C_qAr), 138.0 (C-5), 135.2 (C_qAr), 129.9 (C-Ar), 127.7 (C-Ar), 97.4 (C-1), 88.5 (C-4), 59.1 (CH of cyclopentyl), 40.9 (CH_2), 35.7 ($\underline{C}H_2$-CH_2-CH_3), 30.4 (CH_2 of cyclopentyl), 30.0 (CH_2 of cyclopentyl), 23.3 (CH_2 of cyclopentyl), 23.2 (CH_2 of cyclopentyl), 21.6 (CH_3 of Ts), 18.9 (CH_2-$\underline{C}H_2$-CH_3), 13.8 (CH_2-CH_2-$\underline{C}H_3$). HRMS (ESI) m/z: found, 494.0995; calcd. for $C_{23}H_{29}O_4N^{79}BrS$ $[M+H]^+$: 494.1001.

4. Synthesis of thio-oxanorbornadienes (thio-ONDs) 1b-3b, 6b-18b.

Procedure 1: To a solution of halo-OND (x mmol) in THF (1.8 mL/mmol) and H_2O (0.9 mL/mmol), a solution of *N*-acetylcysteamine (z mmol) in THF (1.0 mL/mmol) was added. Then, K_2CO_3 was added in portions (1 eq) each 30 minutes. The reaction was vigorously stirred at r.t for 2 h. The mixture was diluted with DCM and washed twice with water. Then, the organic layer was dried with Na_2SO_4 anhydrous, filtered and the solvent was removed. Purification by chromatography column on silica gel afforded the corresponding thio-OND.

Procedure 2: To a solution of halo-OND (x mmol) in DMF or MeCN (10 mL/ mmol), a solution of *N*-acetylcysteamine (z mmol) in DMF or MeCN (5 mL/ mmol) and phosphate buffer solution (pH 8.0, 50 mM, 10 mL/mmol), were added simultaneously and the mixture was stirred at r.t. for 30 min. Then, solvents were evaporated, and the residue was dissolved in AcOEt and washed with water. The organic phase was dried (Na_2SO_4), filtered and concentrated. Purification by silica gel column chromatography afforded the corresponding thio-ONDs.

- Thio-ONDs **1b**, **3b** and **10b** were prepared as previously described.[9]

Methyl (*rac*)-3-((2-acetamidoethyl) thio)-7-oxabicyclo[2.2.1]hepta-2,5-diene-2-carboxylate (2b)

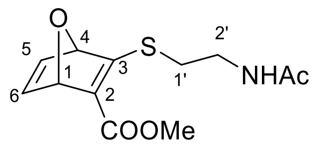

Starting from **2a** (90 mg, 0.39 mmol) and *N*-acetylcysteamine (42 mg, 0.35 mmol) in DMF following general procedure 2, afforded after chromatographic purification (AcOEt:CyHex 10:1→AcOEt) compound **2b** (56 mg, 60%) as a brown oil. ^{1}H NMR (300 MHz, $CDCl_3$, δ ppm): δ 7.14 (dd, 1H, *J*= 5.4 Hz, *J*= 1.7 Hz, H-5 or H-6), 7.06 (dd, 1H, *J*=5.6 Hz, *J*= 1.6 Hz, H-5 or H-6), 6.28 (s, 1H, NH), 5.74-5.71 (m, 2H, H-1, H-4), 3.73 (s, 3H, CH_3 of COOC$\underline{H_3}$), 3.55-3.34 (m, 2H, H-2’), 3.25-3.02 (m, 2H, H-1’), 1.96 (s, 3H, CH_3CO). ^{13}C NMR (75.4 MHz, $CDCl_3$, δ ppm): δ 170.8 (C=O), 169.7 (C=O), 164.0 (C-3), 144.4 (C-5 or C-6), 140.6 (C-5 or C-6), 133.6 (C-2), 85.6, 84.4 (C-1, C-4), 51.4 (COO$\underline{C}H_3$), 40.7 (C-2’), 29.7 (C-1’), 23.1 ($\underline{C}H_3$CO). HRMS (ESI) m/z: found, 292.0614; calcd. for $C_{12}H_{15}O_4N_2NaS_2$ $[M+Na]^+$: 292.0619.

***N*-(2-(((*rac*)-1,4-dimethyl-3-tosyl-7-oxabicyclo[2.2.1]hepta-2,5-dien-2-yl)thio)ethyl)acetamide (6b)**

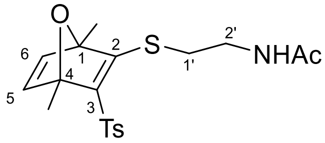

Starting from **6a** (200 mg, 0.56 mmol) and *N*-acetylcysteamine (55 mg, 0.47 mmol) in MeCN following general procedure 2, afforded after chromatographic purification (AcOEt: CyHex 10:1 → AcOEt) compound **6b** (101 mg, 55%) as a brown oil. ^{1}H NMR (300 MHz, CD_3OD, δ ppm): δ 7.61 (d, 2H, *J*= 8.3 Hz, Ar-H), 7.32 (d, 2H, *J*= 8.3 Hz, Ar-H), 6.69 (d, 1H, *J*= 5.3 Hz, H-5 or H-6), 6.58 (d, 1H, *J*= 5.3 Hz, H-5 or H-6), 3.10-2.97 (m, 4H, H-1', H-2'), 2.33 (s, 3H, CH_3 of Ts), 1.83 (s, 3H, CH_3CO), 1.64 (s, 3H, CH_3), 1.58 (s, 3H, CH_3). ^{13}C NMR (75.4 MHz, CD_3OD, δ ppm): δ 173.2 (C=O), 169.5, 150.2 (C-2, C-3), 148.2 (C-5 or C-6), 146.2 (C_qAr), 144.9 (C-5 or C-6), 138.6 (C_qAr), 130.9 (CAr), 128.3 (CAr), 96.9, 93.6 (C-1, C-4), 41.0, 33.2 (C-1', C-2'), 22.5 (CH_3 of Ts), 21.5 ($\underline{C}H_3CO$), 17.1 (CH_3), 16.8 (CH_3). HRMS (ESI) m/z: found, 416.0951; calcd. for $C_{19}H_{23}O_4NNaS_2$ $[M+Na]^+$: 416.0966.

***N*-(2-(((*rac*)-4-methyl-3-tosyl-7-oxabicyclo[2.2.1]hepta-2,5-dien-2-yl)thio)ethyl)acetamide (7b) and *N*-(2-(((*rac*)-1-methyl-3-tosyl-7-oxabicyclo[2.2.1]hepta-2,5-dien-2-yl)thio)ethyl)acetamide (8b)**

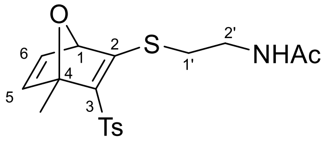

7b

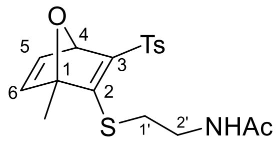

8b

Starting from a mixture of **7a** and **8a** (432 mg, 1.27 mmol) and *N*-acetylcysteamine (120 mg, 1.00 mmol) in MeCN following general procedure 2, afforded after chromatographic purification (Et_2O: Acetone 15:1 → 3:1) compounds **7b** (130 mg, 34%) and **8b** (20 mg, 5%) as a yellow solid. Data for **7b**: ^{1}H NMR (300 MHz, CD_3OD, δ ppm): δ 7.70 (d, 2H, *J*= 8.3 Hz, Ar-H), 7.43 (d, 2H, *J*= 8.3 Hz, Ar-H), 7.00 (dd, 1H, *J*= 5.2, 2.0 Hz, H-6), 6.60 (d, 1H, *J*= 5.1 Hz, H-5), 5.91 (d, 1H, *J*= 2.0 Hz, H-1), 3.45-3.42 (m, 2H, H-1'a, H-2'a), 3.27-3.18 (m, 1H, H-2'b), 3.13-3.03 (m, 1H, H-1'b), 2.46 (s, 3H, CH_3 of Ts), 1.97 (s, 3H, CH_3CO), 1.67 (s, 3H, CH_3). ^{13}C NMR (75.4 MHz, CD_3OD, δ ppm): δ 172.4 (C=O), 170.5 (C-2 or C-3), 145.8 (C-6), 144.9 (C_qAr), 139.8 (C-2 or C-3), 139.4 (C-5), 137.2 (C_qAr), 129.8 (C-Ar), 126.7 (C-Ar), 93.7 (C-1), 84.8 (C-4), 40.4 (C-2'), 30.3 (C-1'), 21.1 ($\underline{C}H_3CO$), 20.2 (CH_3 of Ts), 15.1 (CH_3). Data for

8b: ^{1}H NMR (300 MHz, CD_3OD, δ ppm): δ 7.65 (d, 2H, *J*= 8.4 Hz, ArH), 7.35 (d, 2H, *J*= 8.5 Hz, ArH), 6.86 (dd, 1H, *J*=5.2, 1.8 Hz, H-5), 6.70 (d, 1H, *J*= 5.2 Hz, H-6), 5.34 (d, 1H, *J*= 1.8 Hz, H-4), 3.18-3.08 (m, 4H, H-1', H-2'), 2.36 (s, 3H, CH_3 of Ts), 1.83 (s, 3H, CH_3CO), 1.62 (s, 3H, CH_3). ^{13}C NMR (75.4 MHz, CD_3OD, δ ppm): δ 172.0 (C=O), 165.1, 146.2 (C-2, C-3), 145.0 (C_qAr), 144.1 (C-5), 142.6 (C-6), 137.3 (C_qAr), 129.8 (C-Ar), 127.1 (C-Ar), 97.5 (C-1), 84.2 (C-4), 39.1, 32.8 (C-1', C-2'), 21.1 ($\underline{C}H_3CO$), 20.2 (CH_3 of Ts), 14.9 (CH_3). HRMS (ESI) m/z: found, 402.0801; calcd. for $C_{18}H_{21}O_4NNaS_2$ $[M+Na]^+$: 402.0810.

Methyl (*rac*)-3-((2-acetamidoethyl)thio)-1,4-dimethyl-7-oxabicyclo[2.2.1]hepta-2,5-diene-2-carboxylate (9b)

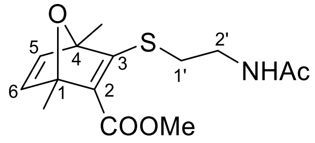

Starting from **9a** (52 mg, 0.17 mmol) and *N*-acetylcysteamine (19 mg, 0.16 mmol) in DMF following general procedure 2, afforded after chromatographic purification (AcOEt:CyHex 10:1→AcOEt) compound **9b** (24 mg, 51%) as a yellow oil. ^{1}H NMR (300 MHz, CD_3OD, δ ppm): δ 6.97 (d, 1H, *J*= 5.1 Hz, H-5 or H-6), 6.90 (d, 1H, *J*= 5.0 Hz, H-5 or H-6), 3.80 (s, 3H, $COOC\underline{H_3}$), 3.26-3.13 (m, 4H, H-1', H-2'), 1.95 (s, 3H, CH_3CO), 1.80 (s, 3H, CH_3), 1.69 (s, 3H, CH_3). ^{13}C NMR (75.4 MHz, CD_3OD, δ ppm): δ 175.4 (C=O), 170.5, (C=O), 168.5 (C-2 or C-3), 150.8, 147.7 (C-5, C-6), 142.6 (C-2 or C-3), 97.1, 95.4 (C-1, C-4), 53.9 ($COO\underline{C}H_3$), 42.0, 34.8 (C-1', C-2'), 24.5 ($\underline{C}H_3CO$), 18.9 (CH_3), 17.8 (CH_3). HRMS (ESI) m/z: found, 320.0926; calcd. for $C_{14}H_{19}O_4NNaS$ $[M+Na]^+$: 320.0932.

***N*-(((*rac*)-3-((2-Acetamidoethyl)thio)-2-tosyl-7-oxabicyclo[2.2.1]hepta-2,5-dien-1-yl)methyl)-*N*-cyclopentylbutyramide (11b)**

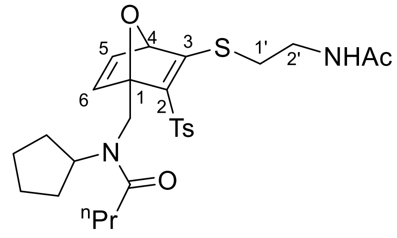

Starting from **11a** (85 mg, 0.17 mmol) and *N*-acetylcysteamine (17 mg, 0.14 mmol) following general procedure 1, afforded after chromatographic purification (AcOEt:CyHex 10:1→AcOEt) compound **11b** (40 mg, 55%) as a yellow oil. ^{1}H NMR (300 MHz, CD_3OD, δ ppm, mixture of rotamers, ratio 3:1):

δ 8.21 (br t, 0.3H, *J*= 4.5 Hz, NH rotamer A), 7.66 (d, 2H, *J*= 8.2 Hz, ArH-rotamer A), 7.61 (d, 2H, *J*= 8.4 Hz, ArH-rotamer B), 7.32 (d, 2H, *J*=8.0 Hz, ArH), 7.00 (br s, 0.3H, NH rotamer B), 6.65 (br d, 1H, *J*= 4.1 Hz, H-5), 6.57 (d, 1H, *J*=4.8 Hz, H-6), 5.95 (br s, 1H, H-4-rotamer B), 5.82 (br s, 1H, H-4-rotamer A), 4.97 (d, 1H, *J*=15.3 Hz, CH_2), 4.24 (d, 1H, *J*=16.5 Hz, CH_2-rotamer B), 4.08-3.99 (m, 1H, CH of cyclopentyl), 3.76 (d, 1H, *J*=16.4 Hz, CH_2- rotamer A), 3.31-3.24 (m, 2H, H-2'), 3.14-2.87 (m, 2H, H-1'), 2.51-2.46 (m, 1H, $\underline{CH_2}$-CH_2-CH_3), 2.34-2.29 (m, 4H, CH_3 of Ts, $\underline{CH_2}$-CH_2-CH_3), 1.85-1.81 (m, 5H, CH_3CO, CH_2-$\underline{CH_2}$-CH_3), 1.70-1.41 (m, 11H, CH_2 of cyclopentyl rotamer A and rotamer B), 0.84 (t, 3H, *J*=7.3 Hz, CH_2-CH_2-$\underline{CH_3}$). ^{13}C NMR (75.4 MHz, CD_3OD, δ ppm, mixture of rotamers): δ 175.7, 172.4, 171.4, 144.8, 144.6, 143.0, 138.3, 138.0, 136.6, 129.9, 129.6, 126.9, 97.0, 84.6, 59.4, 42.6, 41.1, 30.1, 32.2, 28.0, 22.0, 23.0, 21.1, 20.2, 18.9, 12.8. HRMS (ESI) m/z: found, 555.1956; calcd. for $C_{27}H_{36}O_5N_2NaS_2$ $[M+Na]^+$: 555.1963.

***tert*-Butyl (((*rac*)-3-((2-acetamidoethyl)thio)-2-tosyl-7-oxabicyclo[2.2.1]hepta-2,5-dien-1-yl)methyl)(cyclopentyl)carbamate (12b) and *tert*-butyl (((*rac*)-2-((2-acetamidoethyl)thio)-3-tosyl-7-oxabicyclo[2.2.1]hepta-2,5-dien-1-yl)methyl)(cyclopentyl)carbamate (13b)**

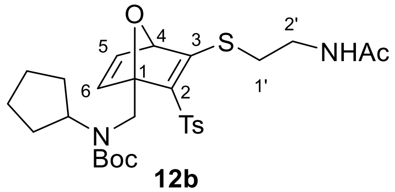

12b

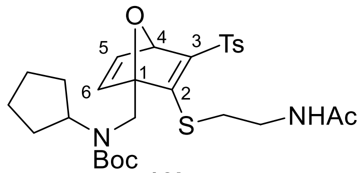

13b

Starting from a mixture of **12a** and **13a** (349 mg, 0.66 mmol) and *N*-acetylcysteamine (66 mg, 0.55 mmol) in MeCN following general procedure 2, afforded after chromatographic purification (AcOEt: CyHex 1:1 → 5:1) compounds **12b** (71 mg, 23%) and **13b** (56 mg, 18%) as a brown oil. Data for **12b**: ^{1}H NMR (300 MHz, CD_3OD, δ ppm, mixture of rotamers): δ 7.79 (d, 2H, *J*= 8.4 Hz, Ar-H), 7.47 (d, 2H, *J*= 8.4 Hz, Ar-H), 5.04 (d, 1H, *J*= 5.3 Hz, H-5), 6.91 (d, 1H, *J*= 5.3 Hz, H-6), 5.53 (d, 1H, *J*= 1.7 Hz, H-4), 4.57 (d, 1H, *J*= 15.0 Hz, CH_2), 3.83-3.78 (m, 1H, CH of cyclopentyl), 3.52 (d, 1H, *J*= 15.0 Hz, CH_2), 3,33-3.27 (m, 4H, H-1', H-2'), 2.47 (s, 3H, CH_3 of Ts), 1.96 (s, 3H, CH_3CO), 1.74-1.69 (m, 6H, CH_2 of cyclopentyl), 1.49-1.44 (m, 11H, $(CH_3)_3$, CH_2 of cyclopentyl). ^{13}C NMR (75MHz, CD_3OD, δ ppm, mixture of rotamers): δ 173.2, 164.2, 146.5, 145.5, 142.0, 138.4, 131.2, 128.5, 102.3, 85.6, 81.5, 61.7, 46.7, 40.3, 35.0, 30.4, 30.2, 28.8, 24.91, 24.87, 22.5, 21.6. Data for **13b**: ^{1}H NMR (300 MHz, CD_3OD, δ ppm, mixture of rotamers): δ 7.75 (d, 2H, *J*= 8.4 Hz, Ar-H), 7.43 (d, 2H, *J*= 8.3 Hz, Ar-H), 6.92 (br s, 1H, H-5), 6.78 (br s, 1H, H-6), 5.98 (d, 1H, *J*= 1.7 Hz, H-4), 3.78-3.70 (m, 1H, CH of cyclopentyl), 3.45-3.38 (m, 2H, H-1'), 3.28-3.19 (m, 1H, H-2'), 3.13-3.00 (m, 1H, H-2'), 2.46 (s, 3H,

CH_3 of Ts), 1.97 (s, 3H, CH_3CO), 1.78-1.63 (m, 6H, CH_2 of cyclopentyl), 1.52-1.42 (m, 11H, $(CH_3)_3$, CH_2 of cyclopentyl). ^{13}C NMR (75 MHz CD_3OD, δ ppm, mixture of rotamers): δ 173.8, 146.2, 145.1, 139.6, 131.0, 128.1, 99.2, 86.3, 81.3, 62.2, 47.2, 41.8, 31.8, 30.5, 29.4, 28.9, 25.0, 22.5, 21.6. HRMS (ESI): found, 585.2069; calcd for $C_{28}H_{38}O_6N_2NaS_2$ $[M+Na]^+$: 585.2069.

***N*-(((*rac*)-3-((2-Acetamidoethyl)thio)-2-tosyl-7-oxabicyclo[2.2.1]hepta-2,5-dien-1-yl)methyl)butyramide (14b)**

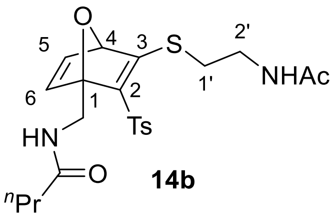

14b

Starting from **14a** (390 mg, 0.91 mmol) and *N*-acetylcysteamine (99 mg, 0.83 mmol) in MeCN following general procedure 2, afforded after chromatographic purification (AcOEt: MeOH 15:1) compound **14b** (270 mg, 70%) as a pale-yellow solid. ^{1}H NMR (300 MHz, CD_3OD, δ ppm): δ 7.74 (d, 2H, *J*= 8.5 Hz, Ar-H), 7.43 (d, 2H, *J*= 8.4 Hz, Ar-H), 6.93 (dd, 1H, *J*= 5.3 Hz, *J*=1.3 Hz, H-5), 6.56 (d, 1H, *J*=5.2 Hz, H-6), 5.99 (d, 1H, *J*=1.9 Hz, H-4), 4.49 (d, 1H, *J*= 14.7 Hz, CH_2), 3.51 (d, 1H, *J*= 15.1 Hz, CH_2), 3.44-3.38 (m, 2H, H-2'), 3.25-3.18 (m, 1H, H-1'), 3,14-3.09 (m, 1H, H-1'), 2.46 (s, 3H, CH_3 of Ts), 2.14 (t, 2H, *J*=7.2 Hz, $C\underline{H_2}$-CH_2-CH_3), 1.98 (s, 3H, CH_3CO), 1.66-1.54 (m, 2H, CH_2-$C\underline{H_2}$-CH_3), 0.92 (t, 3H, *J*= 7.6 Hz, CH_2-CH_2-$C\underline{H_3}$). ^{13}CNMR (75 MHz, CD_3OD, δ ppm): δ 174.9 (C=O), 171.3 (C=O), 145.0 (C-2 or C-3), 143.0 (C-6), 139.7 (C-5), 137.6 (C-2 or C-3), 136.5 (C_qAr), 129.7 (CAr), 126.8 (CAr), 96.7 (C-1), 85.1 (C-4), 40.3 (C-2'), 38.0 (CH_2), 37.9 ($\underline{C}H_2$-CH_2-CH_3), 30.4 (C-1'), 21.1 ($\underline{C}H_3CO$), 20.2 (CH_3 of Ts), 18.9 (CH_2-$\underline{C}H_2$-CH_3), 12.6 (CH_2-CH_2-$\underline{C}H_3$). HRMS (ESI): *m/z* calcd for $C_{22}H_{28}N_2O_5S_2Na$ $[M+Na]^+$, 487.1328; found 487.1332.

***N*-(2-(((*rac*)-1-methyl-4-(((4-methylphenyl)sulfonamido)methyl)-3-tosyl-7-oxabicyclo[2.2.1]hepta-2,5-dien-2-yl)thio)ethyl)acetamide (15b) and *N*-(2-(((*rac*)-4-methyl-1-(((4-methylphenyl)sulfonamido)methyl)-3-tosyl-7-oxabicyclo[2.2.1]hepta-2,5-dien-2-yl)thio)ethyl)acetamide (16b).**

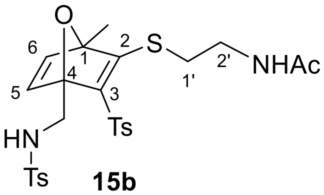

15b

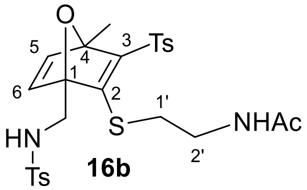

16b

Starting from a mixture of **15a** and **16a** (450 mg, 0.86 mmol) and *N*-acetylcysteamine (85 mg, 0.72 mmol) in MeCN following general procedure 2, afforded after chromatographic purification (AcOEt:CyHex 10:1 → AcOEt) compounds **15b** (122 mg, 27 %) and **16b** (58 mg, 13 %) as a white solid. Data for **15b**: ^{1}H NMR (300 MHz, $CDCl_3$, δ ppm): δ 7.75-7.72 (d, 2H, *J*= 8.5 Hz, ArH), 7.70-7.68 (d, 2H, *J*= 8.6 Hz, ArH), 7.32, 7.31 (2d, 4H, *J*=8.2, ArH), 6.71 (d, 1H, *J*=5.5 Hz, H-6), 6.64 (d, 1H, *J*=5.5 Hz, H-5), 6.52 (br s, 1H, NHCO), 5.01 (dd, 1H, *J*= 8.5, 4.3 Hz, N<u>H</u>-CH_2), 3.87 (dd, 1H, *J*=14.0 Hz, *J*=5.5 Hz, CH_2), 3.52 (dd, 1H, *J*=13.9 Hz, *J*=4.6 Hz, CH_2), 3.42-3.25 (m, 2H, H-2'), 3.07-3.01 (m, 2H, H-1'), 2.43 (s, 6H, 2CH_3 of Ts), 1.93 (s, 3H, CH_3CO), 1.68 (s, 3H, CH_3). ^{13}C NMR (75 MHz, $CDCl_{3,}$ δ ppm): δ 170.5 (C=O), 168.6, 150.7 (C-2, C-3), 145.2 (C_qAr), 144.5 (C-6), 144.0 (C-5), 143.6 (C_qAr), 136.8 (C_qAr), 136.7 (C_qAr), 130.1 (C-Ar), 129.8 (C-Ar), 127.4 (C-Ar), 127.2 (C-Ar), 96.3, 94.6 (C-1, C-4), 42.7 (CH_2), 39.8 (C-2'), 33.3 (C-1'), 23.2 (CH_3CO), 21.7 (CH_3 of Ts), 21.6 (CH_3 of Ts), 16.0 (CH_3). Data for **16b**: ^{1}H NMR (300 MHz, $CDCl_3$, δ ppm): δ 7.73 (d, 2H, *J*=7.7 Hz, ArH), 7.70 (d, 2H, *J*=7.7 Hz, ArH), 7.35 (d, 2H, *J*= 8.3, ArH), 7.31 (d, 2H, *J*= 8.3, ArH), 6.72 (d, 1H, *J*= 5.3 Hz, H-5 or H-6), 6.69 (d, 1H, *J*= 5.3 Hz, H-5 or H-6), 6.65 (t, 1H, *J*= 6.1 Hz, NH) 4.84 (t, 1H, *J*=4.7 Hz, N<u>H</u>-CH_2), 3.62- 3.57 (m, 2H, H-2'), 3.48- 3.42 (m, 1H, CH_2), 3.33- 3.25 (m, 1H, CH_2), 3.11 (t, 2H, *J*= 6.0, H-1'), 2.44 (s, 3H, CH_3 of Ts), 2.42 (s, 3H, CH_3 of Ts), 1.97 (s, 3H, CH_3CO), 1.67 (s, 3H, CH_3). ^{13}C NMR (75 MHz, $CDCl_3$, δ ppm): δ 170.5 (C=O), 168.6, 154.0 (C-2, C-3), 147.8 (C-5 or C-6), 145.1 (C_qAr), 144.0 (C_qAr), 141.0 (C-5 or C-6), 137.1 (C_qAr), 136.1 (C_qAr), 130.0 (C-Ar), 129.9 (C-Ar), 127.4 (C-Ar), 127.1 (CAr), 96.7, 93.4 (C-1, C-4), 42.2 (C-2'), 39.6 (CH_2), 34.3 (C-1'), 23.7 (<u>C</u>H_3CO), 21.7 (CH_3 of Ts), 21.6 (CH_3 of Ts), 16.3 (CH_3).). HRMS (ESI) m/z: found, 585.1147; calcd. for $C_{26}H_{30}O_6N_2NaS_3$ $[M+Na]^+$: 585.1164.

***N*-(2-(((*rac*)-5-(Hydroxymethyl)-3-tosyl-7-oxabicyclo[2.2.1]hepta-2,5-dien-2-yl)thio)ethyl)acetamide (17b) and *N*-(2-(((*rac*)-6-(hydroxymethyl)-3-tosyl-7-oxabicyclo[2.2.1]hepta-2,5-dien-2-yl)thio)ethyl)acetamide (18b)**

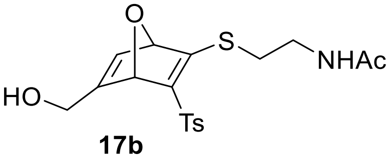

17b

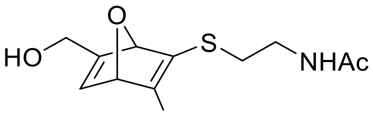

18b

A solution of mixture of compounds **17a** and **18a** (370 mg, 1.04 mmol) in MeCN (10 mL), a solution of *N*-acetylcysteamine (112 mg, 0.94 mmol) in MeCN (5 mL) and phosphate buffer solution (pH 8.0, 50 mM, 10 mL), were added simultaneously and the mixture was stirred at r.t. for 30 min. When finished the reaction, solvents were evaporated. The residue was dissolved in DCM, left overnight at 0°C and filtered to give pure compound **17b** (112 mg, 30%) as white solid. The filtrate was concentrated and the residue purified by chromatography column (AcOEt → AcOEt: MeOH 10:1) to obtain the pure compound **18b** (82 mg, 22 %) as a brown oil. Data for **17b**: ^{1}H NMR (300 MHz, DMSO-d_6, δ ppm, *J* Hz): δ 8.19 (t, 1H, *J*=5.3, NH), 7.73 (d, 2H, *J*=8.2, Ar-H), 7.46 (d, 2H, *J*=8.0, Ar-H), 6.72 (d, 1H, *J*=1.8, H-6), 6.00 (s, 1H, H-1), 5.43 (d, 1H, *J*=1.3, H-4), 5.00 (t, 1H, *J*=5.1, OH), 3.96 (ddd, 2H, *J*=17.7, *J*=5.8, *J*=1.9, CH_2), 3.24-3.20 (m, 2H, H-2'), 3.14-3.11 (m, 2H, H-1'), 2.42 (s, 3H, CH_3 of Ts), 1.83 (s, 3H, CH_3CO). ^{13}C NMR (75 MHz, DMSO-d_6, δ ppm): δ 170.2 (C=O), 161.0 (C-5 or C-6), 144.9 (C_qAr), 138.9 (C-5 or C-6), 138.7 (C_qAr), 131.5 (C-3), 130.6 (C-Ar), 127. 0 (C-Ar), 86.9 (C-1), 84.9 (C-4), 58.1 (CH_2), 40.4 (C-2'), 31.3 (C-1'), 23.0 ($\underline{C}H_3CO$), 21.6 (CH_3 of Ts). Data for **18b**: ^{1}H NMR (300 MHz, CD_3OD, δ ppm, *J* Hz): δ 7.75 (s, 2H, *J*=8.3, Ar-H), 7.44 (d, 2H, *J*=8.0, Ar-H), 6.63-6.61 (m, 1H, H-5), 5.96 (d, 1H, *J*=1.5, H-1), 5.48 (t, 1H, *J*=1.7, H-4), 4.32 (d, 2H, *J*=2.0, CH_2), 3.52-3.37 (m, 4H, H-1', H-2'), 2.47 (s, 3H, CH_3 of Ts), 1.96 (s, 3H, CH_3CO).^{13}C NMR (75 MHz, CD_3OD, δ ppm): δ 172.4 (C=O), 166.7, 155.8 (C-2, C-3), 144.8 (C_qAr), 140.2 (C_qAr), 135.4 (C-5), 129.7 (C-Ar), 126.8 (C-Ar), 87.1 (C-1), 85.3 (C-4), 57.5 (CH_2), 39.7, 31.0 (C-1', C-2'), 21.1 ($\underline{C}H_3CO$), 20.2 (CH_3 of Ts). HRMS (ESI) m/z: found, 418.0756; calcd. for $C_{18}H_{21}O_5NNaS_2$ $[M+Na]^+$: 418.0759.

N,N'-(((2-tosylethene-1,1-diyl)bis(sulfanediyl))bis(ethane-2,1-diyl))diacetamide (20)

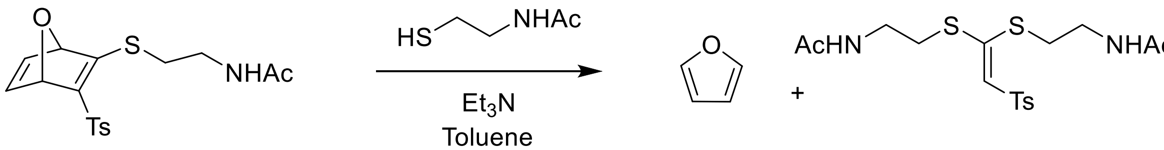

1b

20

To a solution of compound **1b** (92 mg, 0.25 mmol) in toluene (1.5 mL), was added *N*-acetylcysteamine (45 mg, 0.38 mmol) and triethylamine (53 µL, 0.38 mmol) and the mixture was stirred at 50 °C (heat-on block system) for 5 hours. When the reaction was finished, the solvent was evaporated. The residue was purified by column chromatography on silica gel (AcOEt: Acetone 10:1 → Acetone) to yield compound **20** (48 mg, 46%) as colourless oil. ^{1}H-NMR (500 MHz, C_6D_6, δ ppm, *J* Hz): δ 8.02 (d, 2H, *J*= 8.0 Hz, ArH), 6.85 (d, 2H, *J*= 7.9, ArH), 6.64 (s, 1H, H of alkene), 6.47 (br s, 1H, NH), 6.24 (br s, 1H, NH), 3.17 (q, 2H, *J*= 6.0 Hz, CH_2), 3.10 (q, 2H, *J*= 6.0 Hz, CH_2), 2.83 (t, 2H, *J*= 6.1

Hz, CH_2), 2.48 (t, 2H, *J*= 6.1 Hz, CH_2), 1.89 (s, 3H, CH_3CO), 1.81 (s, 3H, CH_3CO), 1.69 (s, 3H, CH_3). ^{13}C-NMR (125 MHz, C_6D_6, δ ppm): δ 170.1, 169.9, 154.1, 143.9, 140.5, 129.7, 126.6, 39.5, 37.4, 34.1, 33.5, 22.8, 22.7, 21.1. HRMS (ESI) m/z: found, 439.0796; calcd. for $C_{17}H_{24}O_4N_2NaS_3$ $[M+Na]^+$: 439.0796.

Methyl 3,3-bis((2-acetamidoethyl)thio)acrylate (21)

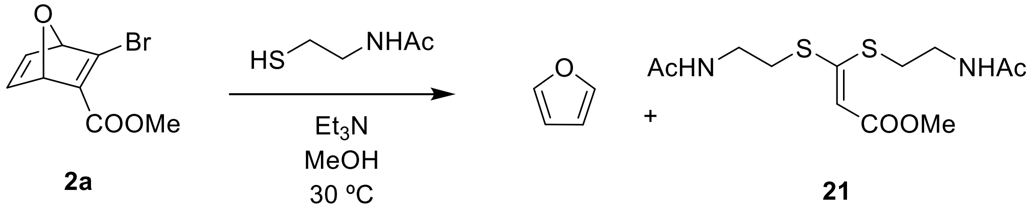

To a solution of compound **2a** (120 mg, 0.52 mmol) in MeOH (5 mL), was added *N*-acetylcysteamine (150 mg, 1.04 mmol) and triethylamine (182 µL, 1.30 mmol) and the mixture was stirred at 30 °C for 65 h (heat-on block system). When the reaction was finished, the solvent was evaporated. The residue was purified by column chromatography on silica gel (DCM:MeOH 30:1 → 10:1) to yield compound **21** (94 mg, 57%) as white solid. ^{1}H-NMR (300 MHz, $CDCl_3$, δ ppm, *J* Hz): δ 6.77 (br.s, 1H, NH), 6.46 (br.s, 1H, NH), 5.95 (s, 1H, H of alkene), 3.70 (s, 3H, CH_3 of COOMe), 3.52-3.48 (m, 4H, 2xCH_2), 3.14 (t, 2H, *J*= 6.5 Hz, CH_2), 3.07 (t, 2H, *J*= 6.3 Hz, CH_2), 1.98 (s, 3H, CH_3CO), 1.97 (s, 3H, CH_3CO). ^{13}C-NMR (75 MHz, $CDCl_3$, δ ppm): δ 170.9, 170.6 (C=O of $COCH_3$), 165.2 (C=O of COOMe), 155.7 (C_q of alkene), 111.3 (CH of alkene), 51.3 (CH_3 of COOMe), 39.0 (CH_2), 37.6 (CH_2), 33.6 (CH_2), 32.3 (CH_2), 23.2, 23.0 ($CO\underline{C}H_3$). HRMS (ESI) m/z: found, 343.0757; calcd. for $C2_1H_{20}O_4N_2NaS_2$ $[M+Na]^+$: 343.0769.

5. Competition experiments.

5.1. General procedure: reaction of a mixture of two halo-ONDs with *N*-acetylcysteamine

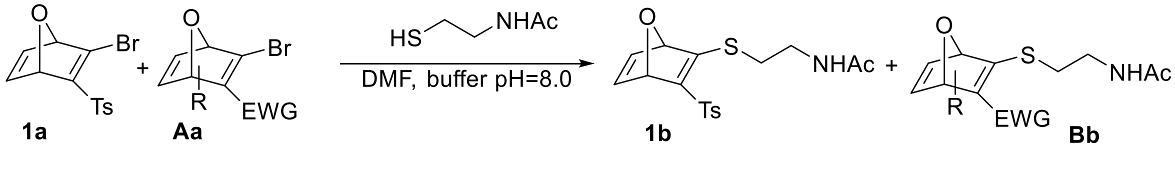

To a solution of compound **1a** (1.2 eq) and **Aa** (1.2 eq) in DMF (20 mL/mmol), phosphate buffer solution (pH 8.0, 50 mM, 25 mL/mmol) and a solution of *N*-acetylcysteamine (1.0 eq) in DMF (4 mL/mmol) were added. The mixture was stirred at r.t. for 30 min and then, solvents were

evaporated, and the residue was dissolved in AcOEt and washed with water. The aqueous phase was extracted with EtOAc (x3) and the combined organic phases were dried (Na_2SO_4), filtered and concentrated. Purification was performed by silica gel column chromatography (DCM:MeOH 100:1 → 15:1). % Conversion **1a**:**Aa** into **1b**:**Bb** was determined by ^{1}H NMR.

5.1.1. Competition experiment **1a**:**2a**

Starting from *N*-acetylcysteamine (7 mg, 0.06 mmol) following the general procedure. % Conversion of **1a**:**2a** into **1b**:**2b** was 37:63 (determined by ^{1}H NMR).

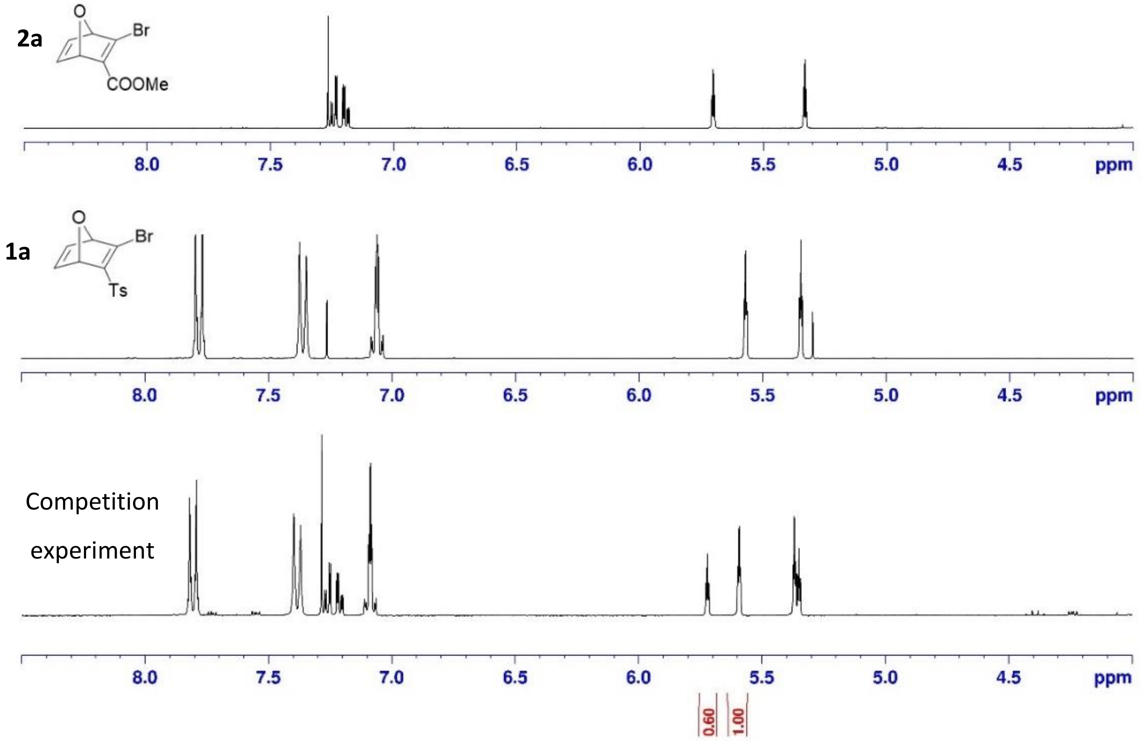

Figure S1. ^{1}H-NMR (300 MHz, $CDCl_3$) of the competition experiment **1a**:**2a** *vs* *N*-acetylcysteamine.

5.1.2. Competition experiment **1a:3a**

Starting from *N*-acetylcysteamine (14.5 mg, 0.12 mmol) following the general procedure. % Conversion of **1a**:**3a** into **1b**:**3b** was 58:42 (determined by ^{1}H NMR).

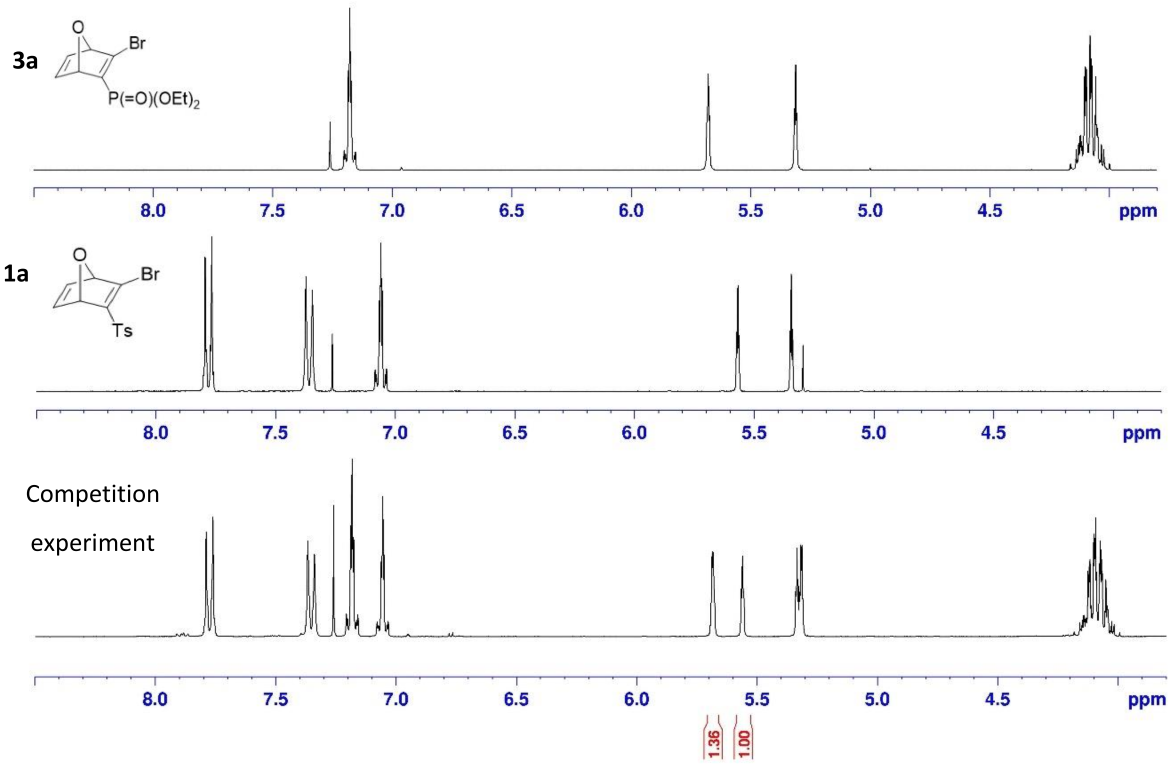

Figure S2. ^{1}H-NMR (300 MHz, $CDCl_3$) of the competition experiment **1a**:**3a** *vs* *N*-acetylcysteamine.

5.1.3. Competition experiment **1a:4**

Starting from *N*-acetylcysteamine (7 mg, 0.06 mmol) following the general procedure. % Conversion of **1a**:**4** into **1b** was 48:52 (determined by ^{1}H NMR).

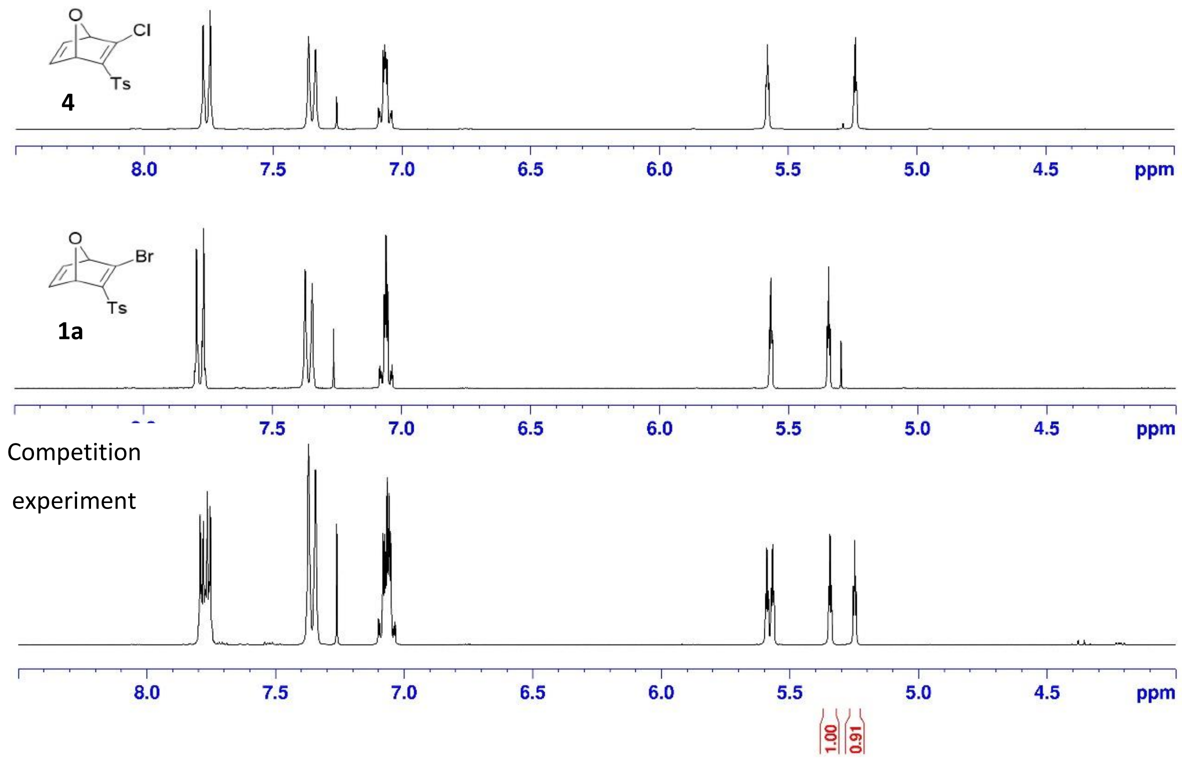

Figure S3. ^{1}H-NMR (300 MHz, $CDCl_3$) of the competition experiment **1a**:**4** *vs* *N*-acetylcysteamine.

5.1.4. Competition experiment **1a:5**

Starting from *N*-acetylcysteamine (7 mg, 0.06 mmol) following general procedure. % Conversion of **1a**:**5** into **1b** was 62:38 (determined by ^{1}H NMR).

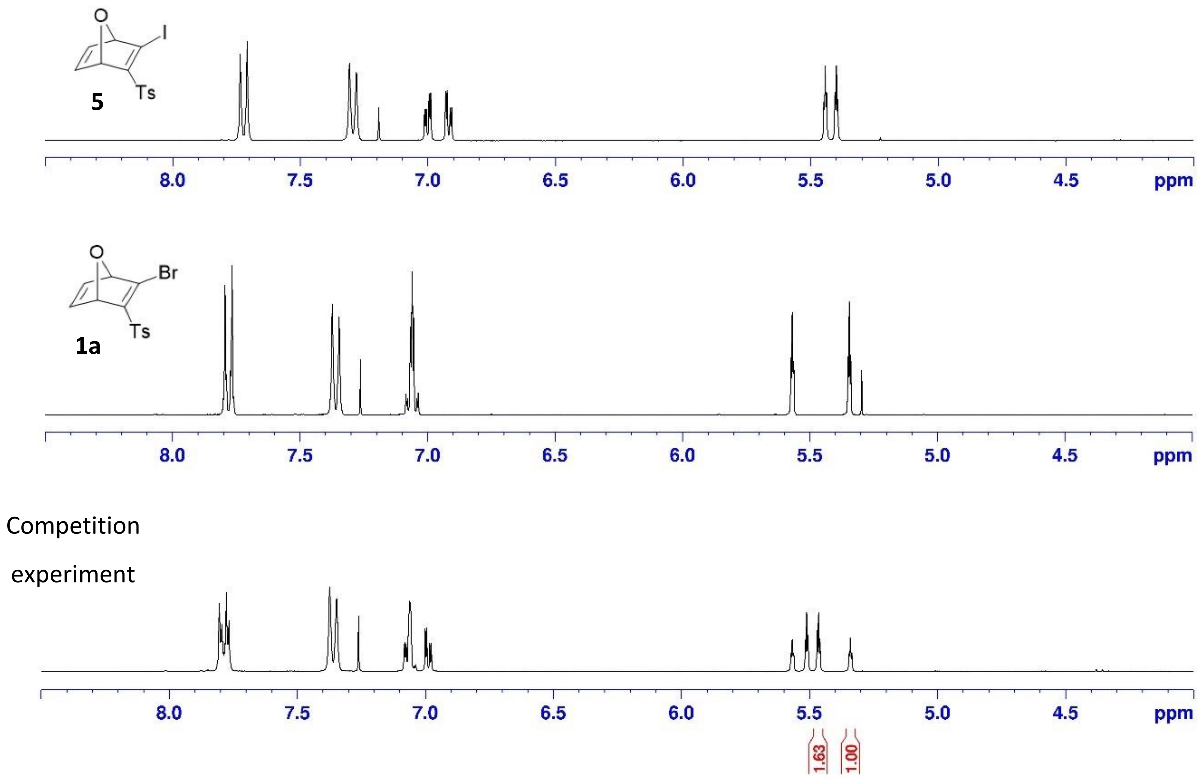

Figure S4. ^{1}H-NMR (300 MHz, $CDCl_3$) of the competition experiment **1a**:**5** *vs* *N*-acetylcysteamine.

5.1.5 Competition experiment **1a:8a**

Starting from *N*-acetylcysteamine (7.5 mg, 0.062 mmol) following general procedure. % Conversion of **1a**:**8a** into **1b**:**8b** was 54:46 (determined by ^{1}H NMR).

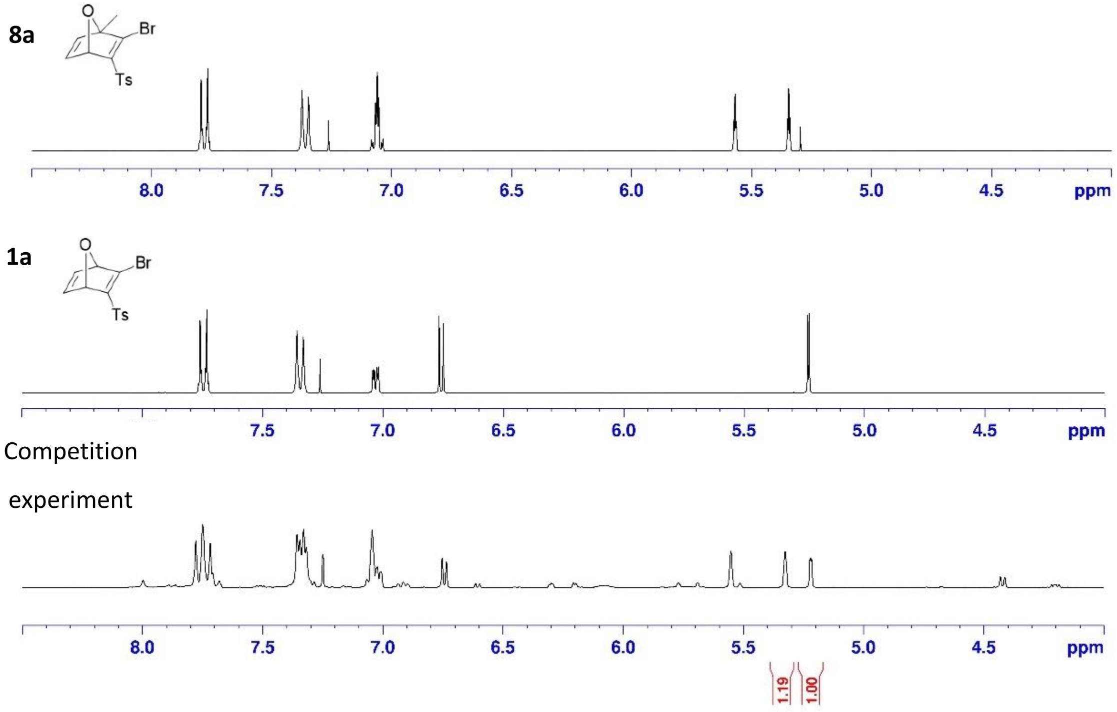

Figure S5. ^{1}H-NMR (300 MHz, 300 $CDCl_3$) of the competition experiment **1a**:**8a** *vs* *N*-acetylcysteamine.

6. Studies of the fragmentation of thio-ONDs *via* ^{1}H-NMR. Selected examples

6.1. General procedure.

The compound **Bb** (0.04 mmol) was dissolved in CD_3OD or DMSO-d_6 (0.5 mL) in a NMR tube. Then, *N*-acetylcysteamine (0.05 mmol) and triethylamine (0.05 mmol) in MeOD or DMSO-d_6 (0.1 mL) were added to the NMR tube, and the reaction was left to complete at 30 °C. ^{1}H-NMR spectra were registered at regular intervals.

6.2. Selected examples of the ^{1}H-NMR experiments.

6.2.1. ^{1}H-NMR experiments for the thiol-promoted fragmentation of **6b** at different intervals.

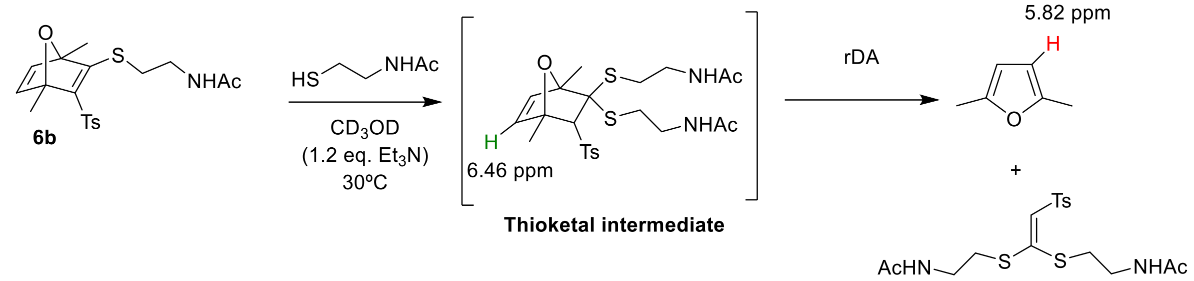

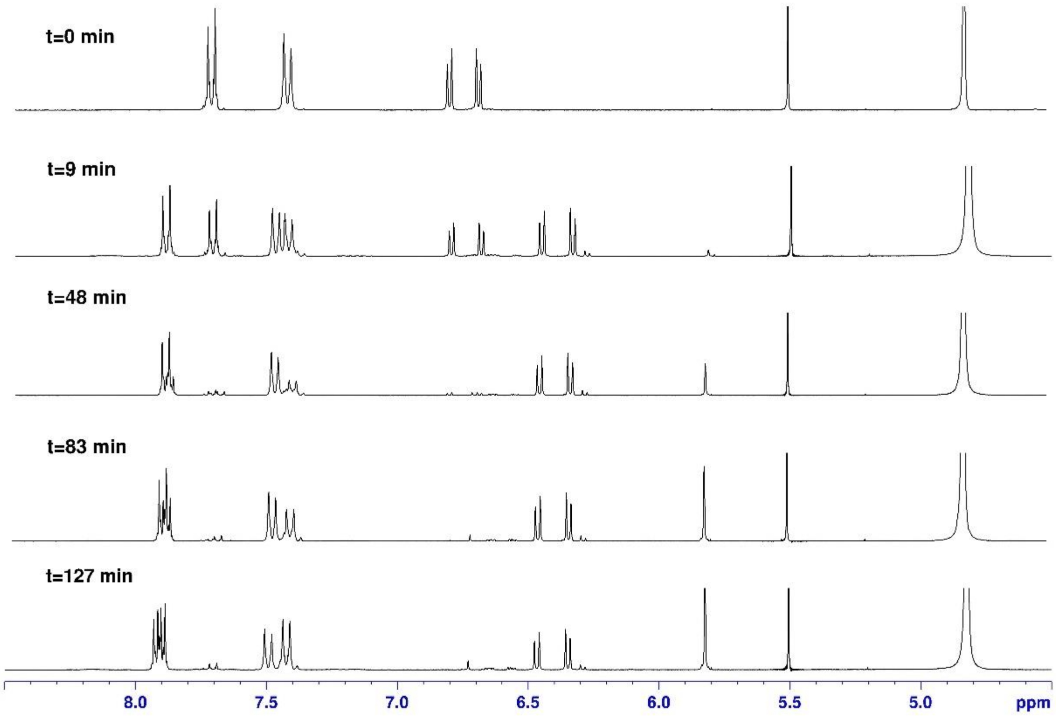

Figure S6. ^{1}H NMR (300 MHz, CD_3OD, 303 K).

6.2.2. ^{1}H-NMR experiments for the thiol-promoted fragmentation of **8b** at different intervals.

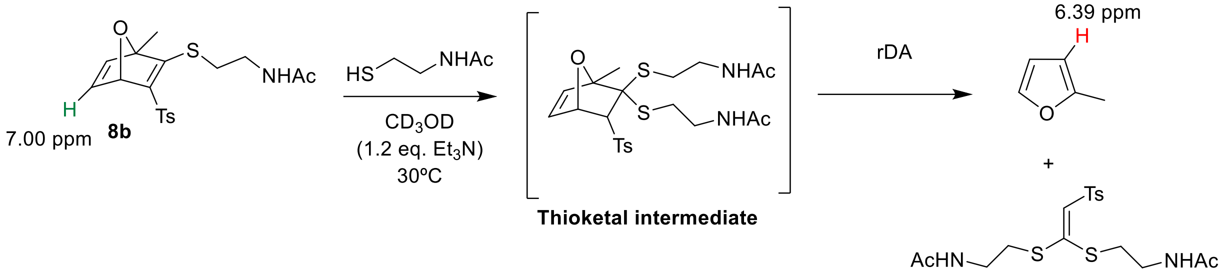

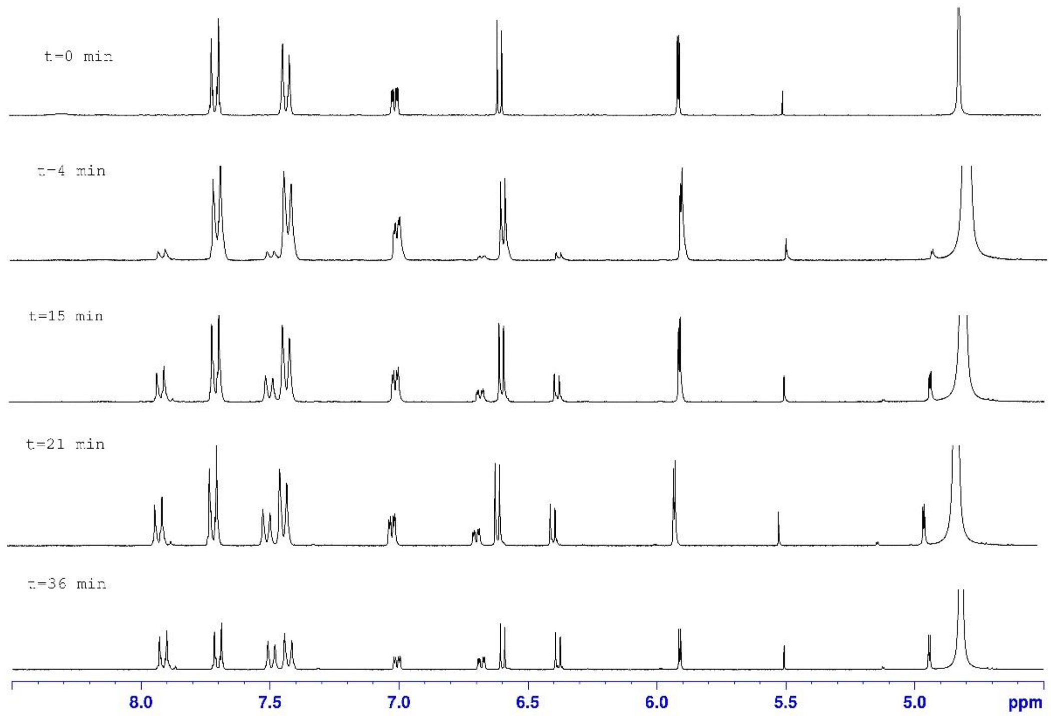

Figure S7. ^{1}H NMR (300 MHz, CD_3OD, 303 K).

6.2.3. ^{1}H-NMR experiments for the thiol-promoted fragmentation of **14b** at different intervals.

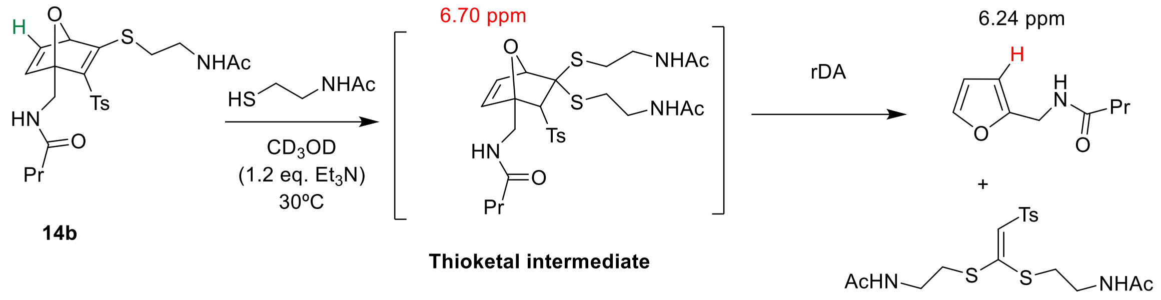

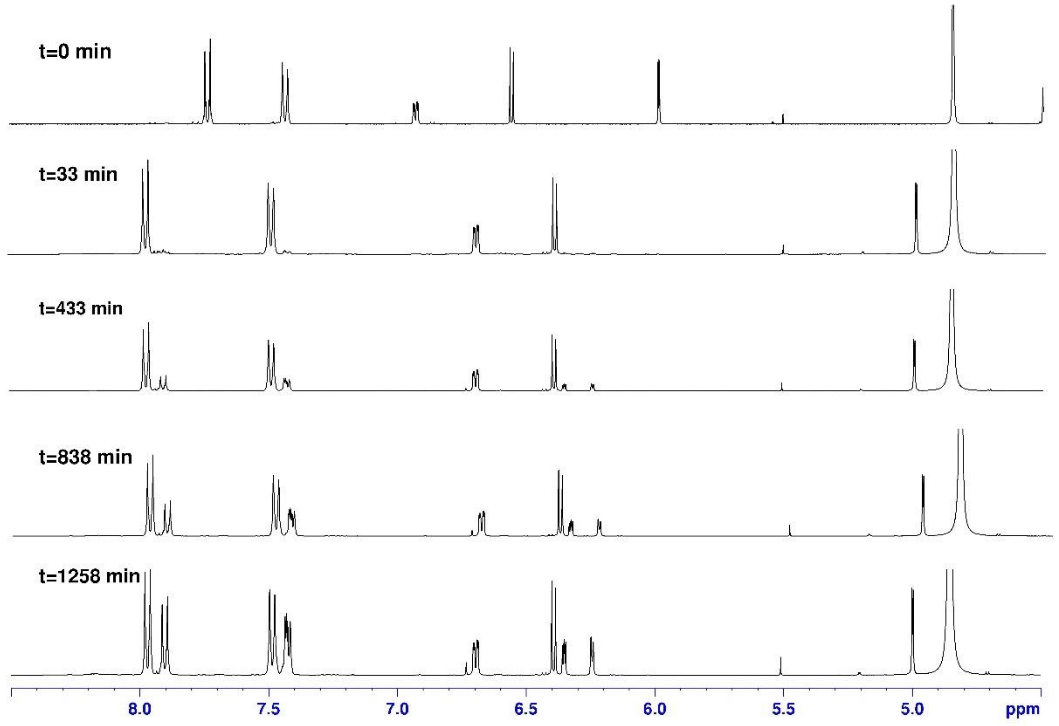

Figure S8. ^{1}H NMR (400 MHz, CD_3OD, 303 K)

6.2.4. ^{1}H-NMR experiments for the thiol-promoted fragmentation of **15b** at different intervals.

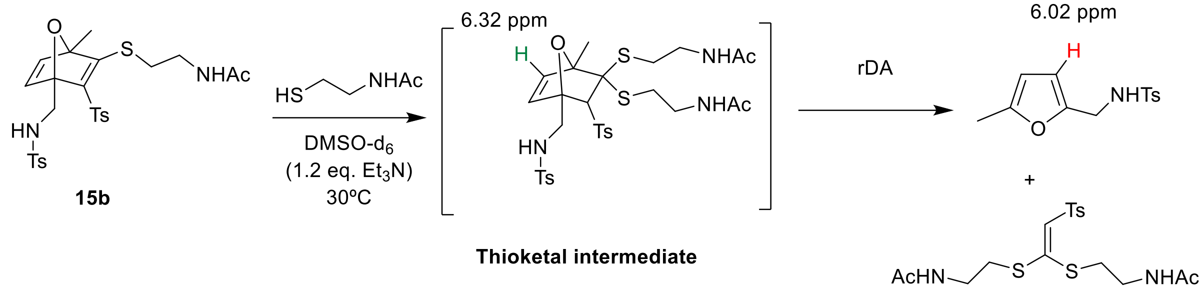

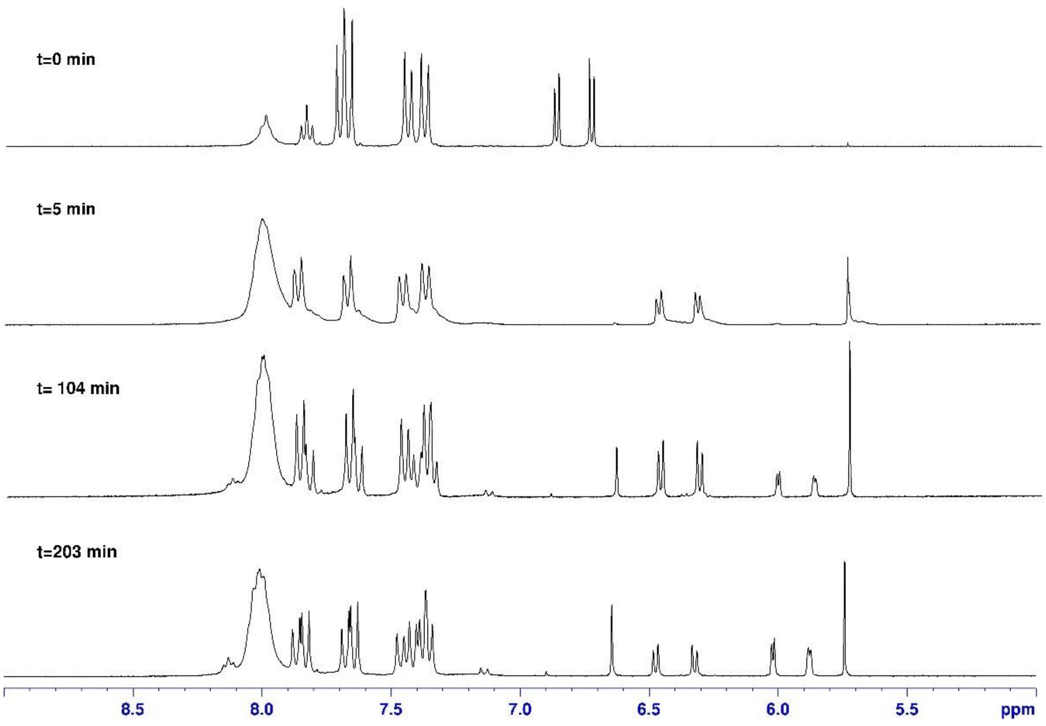

Figure S9. ^{1}H NMR (300 MHz, DMSO-d_6, 303 K).

6.3. Plots for the % conversion *vs* time in the thiol-promoted fragmentation of ONDs.

6.3.1. Fragmentation of compound **1b**.

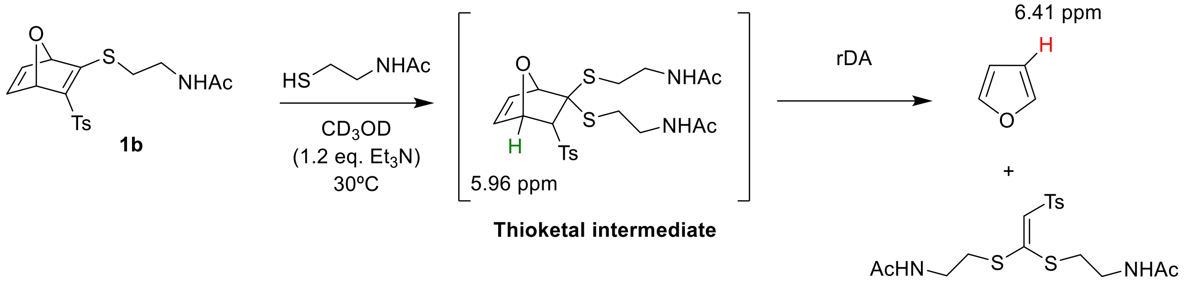

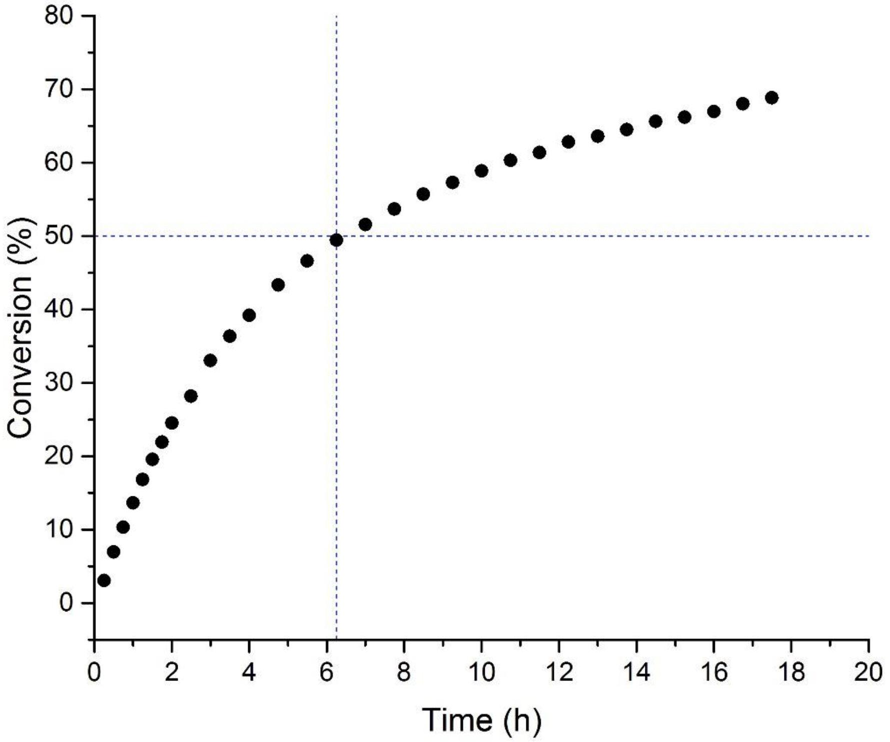

Figure S10. Plot for conversion (%) *vs* time (h) of the fragmentation of **1b**. % Conversion was determined by ^{1}H NMR (500 MHz, CD_3OD, 303 K).

6.3.2. Fragmentation of compound **2b**

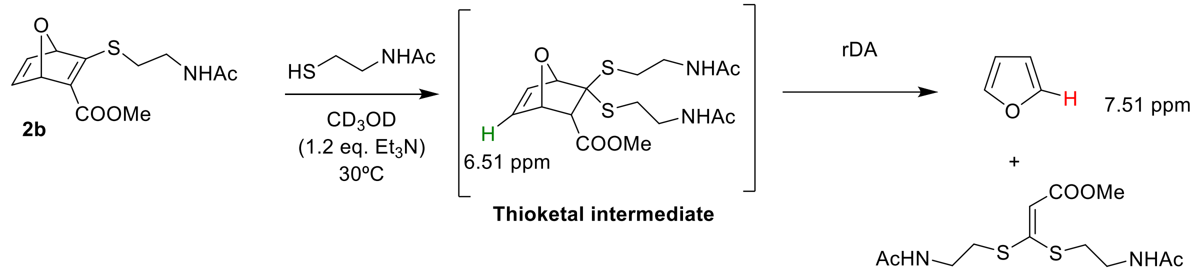

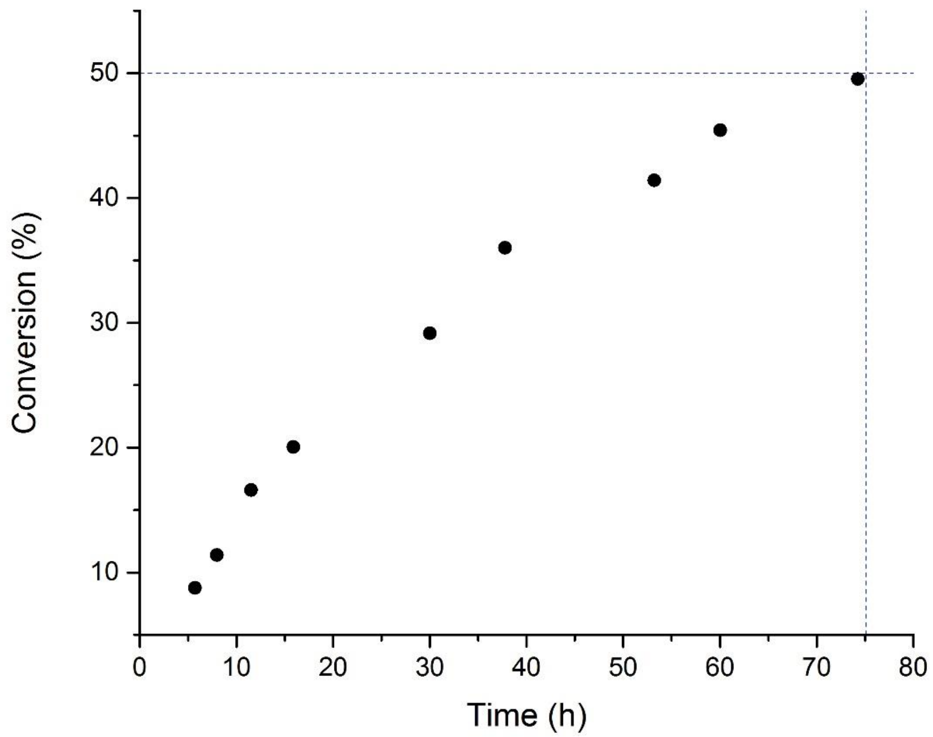

Figure S11. Plot for conversion (%) *vs* time (h) of the fragmentation of **2b**. % Conversion was determined by ^{1}H NMR (500 MHz, CD_3OD, 303 K).

6.3.3. Fragmentation of compound **6b**

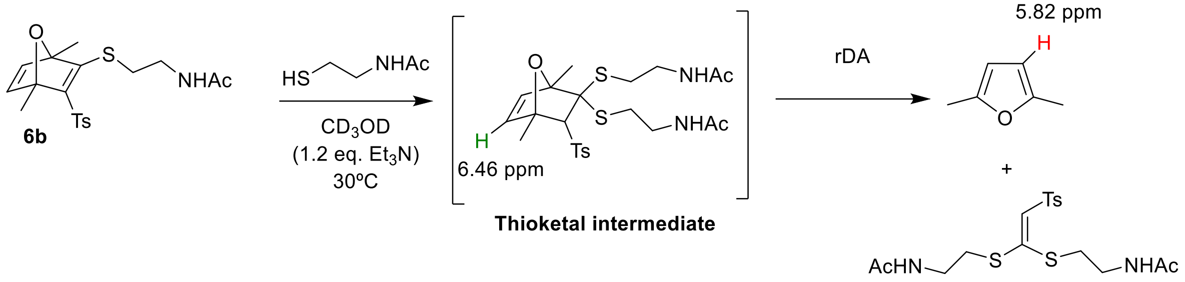

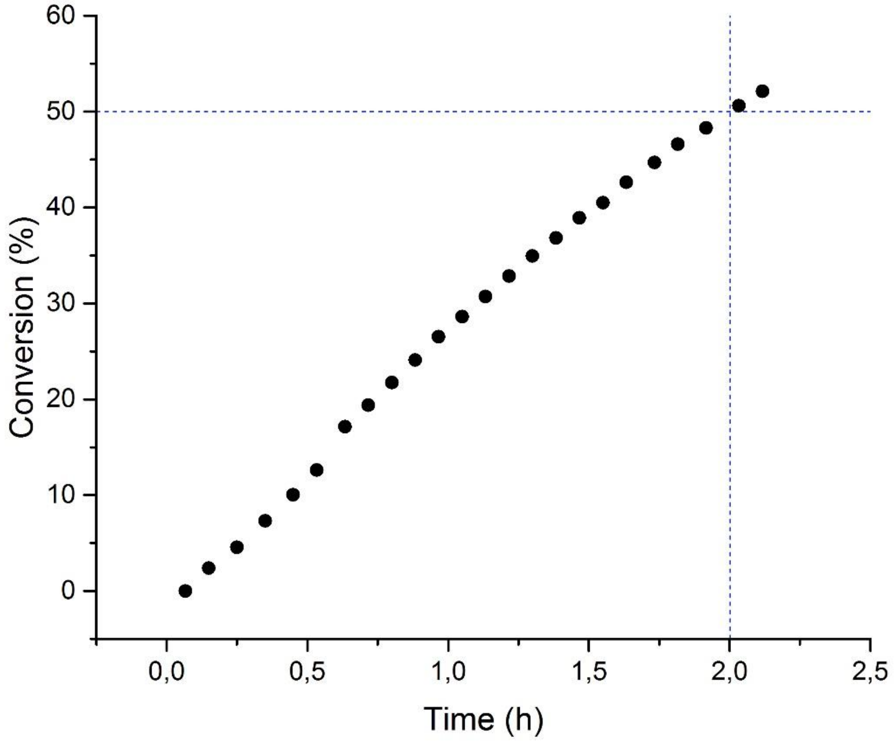

Figure S12. Plot for conversion (%) *vs* time (h) of the fragmentation of **6b**. % Conversion was determined by ^{1}H NMR (300 MHz, CD_3OD, 303 K).

6.3.4. Fragmentation of compound **7b**

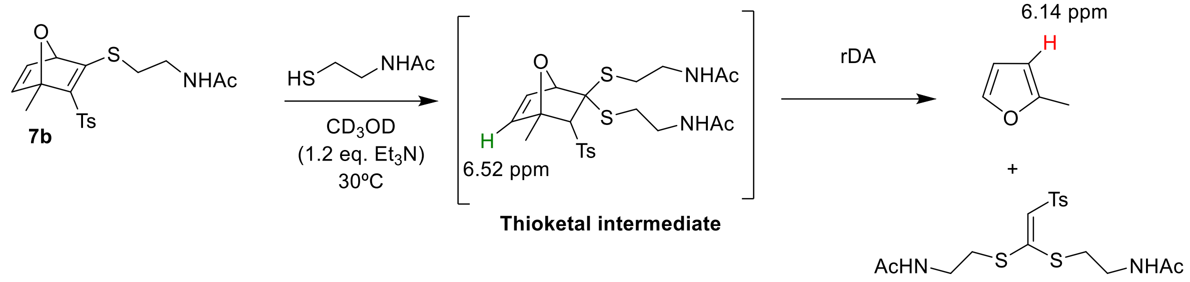

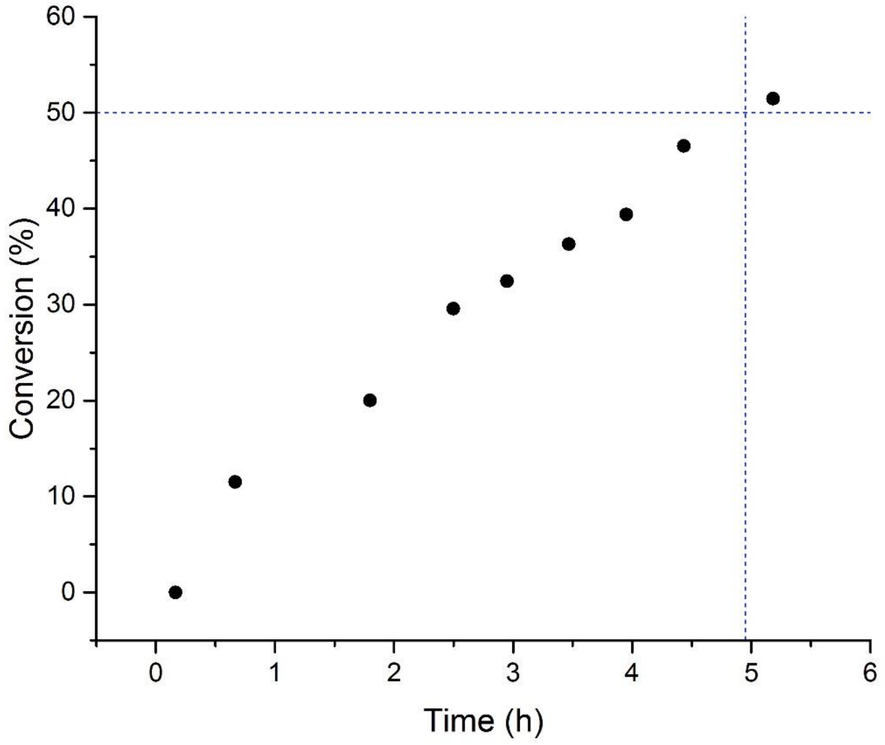

Figure S13. Plot for conversion (%) *vs* time (h) of the fragmentation of **7b**. % Conversion was determined by ^{1}H NMR (80 MHz, CD_3OD, 303 K).

6.3.4. Fragmentation of compound **8b**.

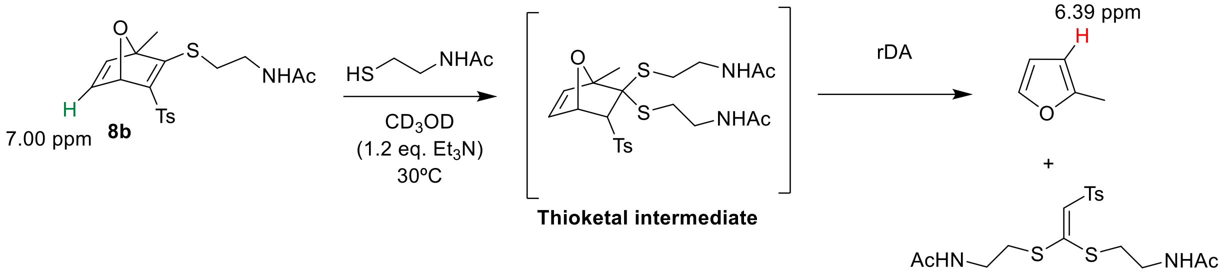

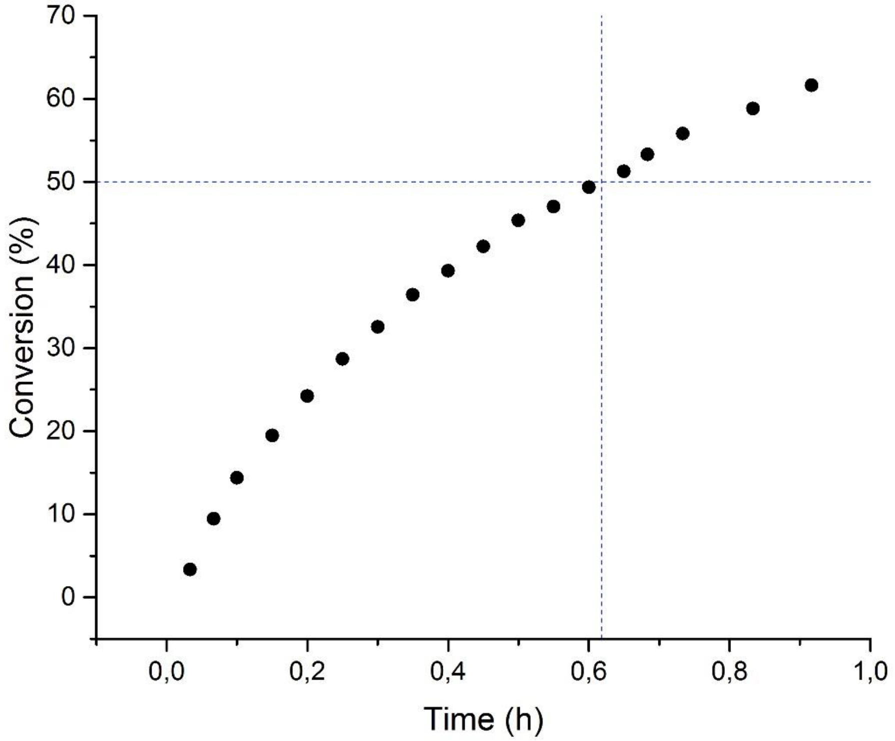

Figure S14. Plot for conversion (%) *vs* time (h) of the fragmentation of **8b**. % Conversion was determined by ^{1}H NMR (300 MHz, CD_3OD, 303 K).

6.3.6. Fragmentation of compound **9b**.

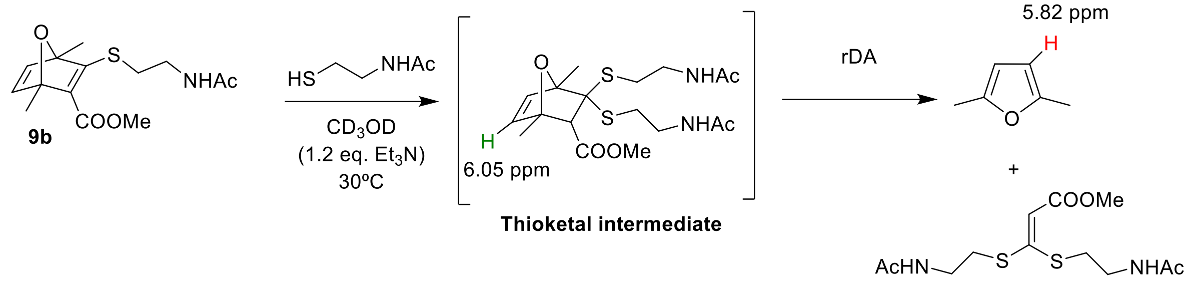

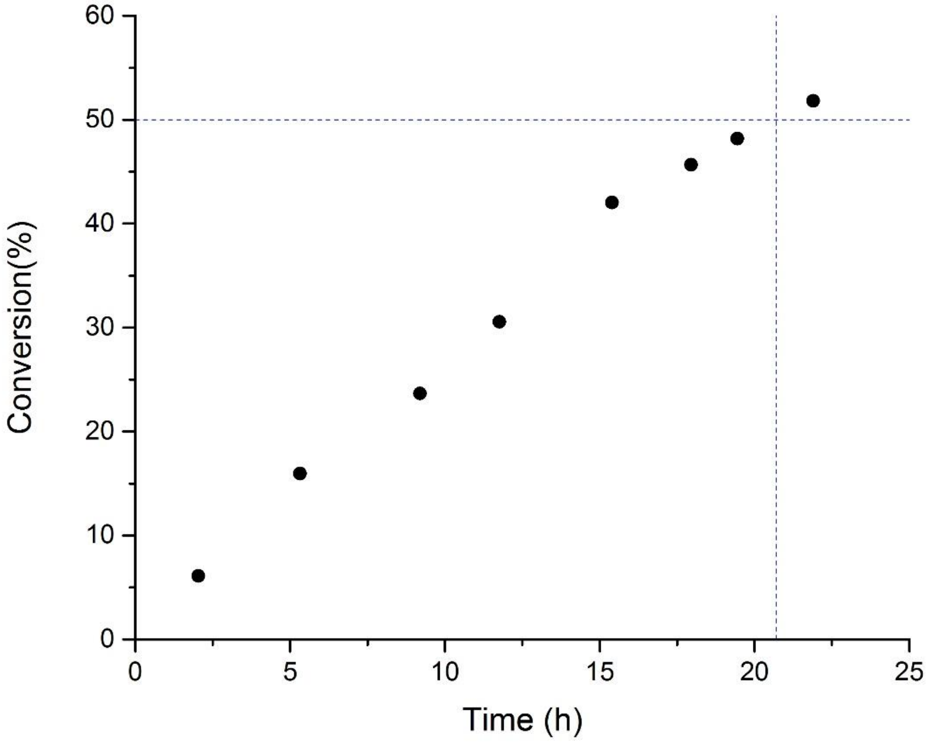

Figure S15. Plot for conversion (%) *vs* time (h) of the fragmentation of **9b**. % Conversion was determined by ^{1}H NMR (80 MHz, CD_3OD, 303 K).

6.3.7. Fragmentation of compound **11b**

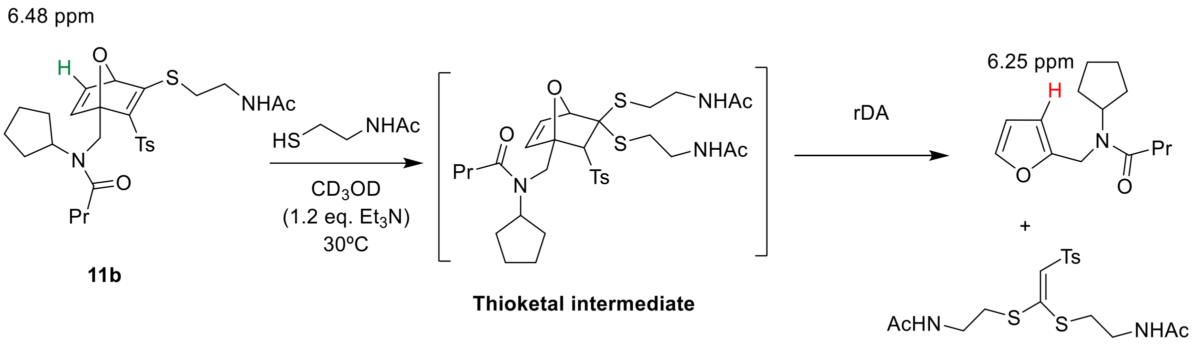

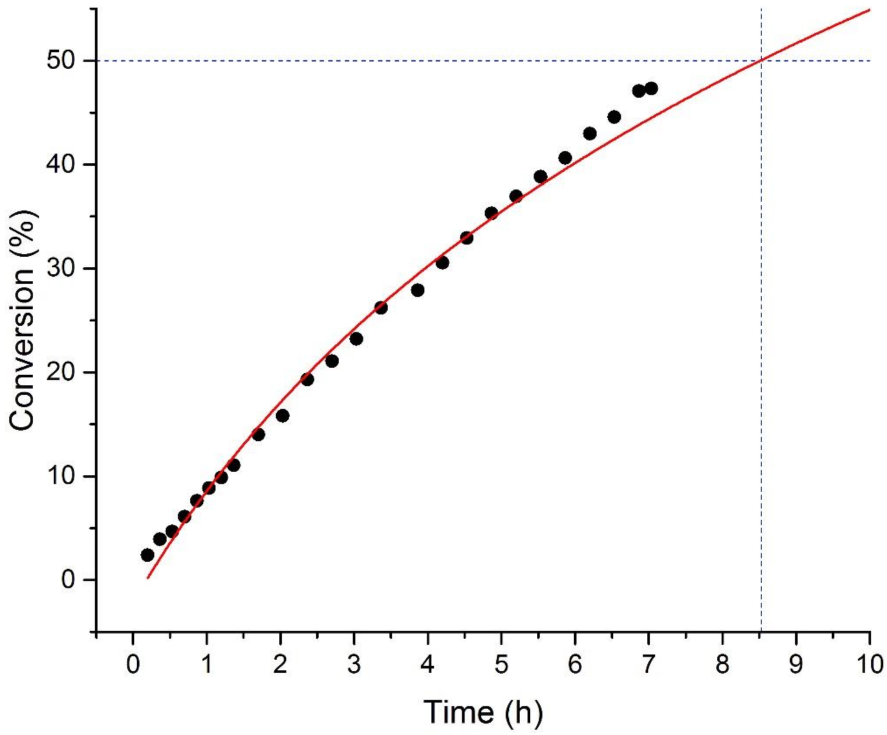

Figure S16. Plot for conversion (%) *vs* time (h) of the fragmentation of **11b**. % Conversion was determined by ^{1}H NMR (500 MHz, CD_3OD, 303 K).

6.3.8. Fragmentation of compound **12b**.

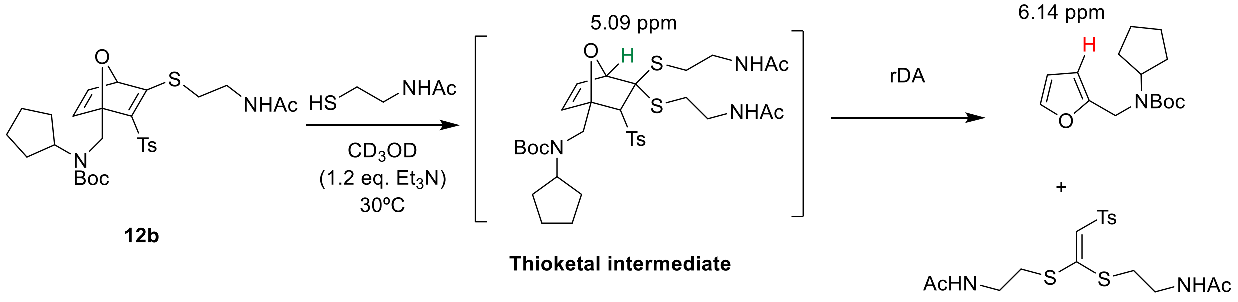

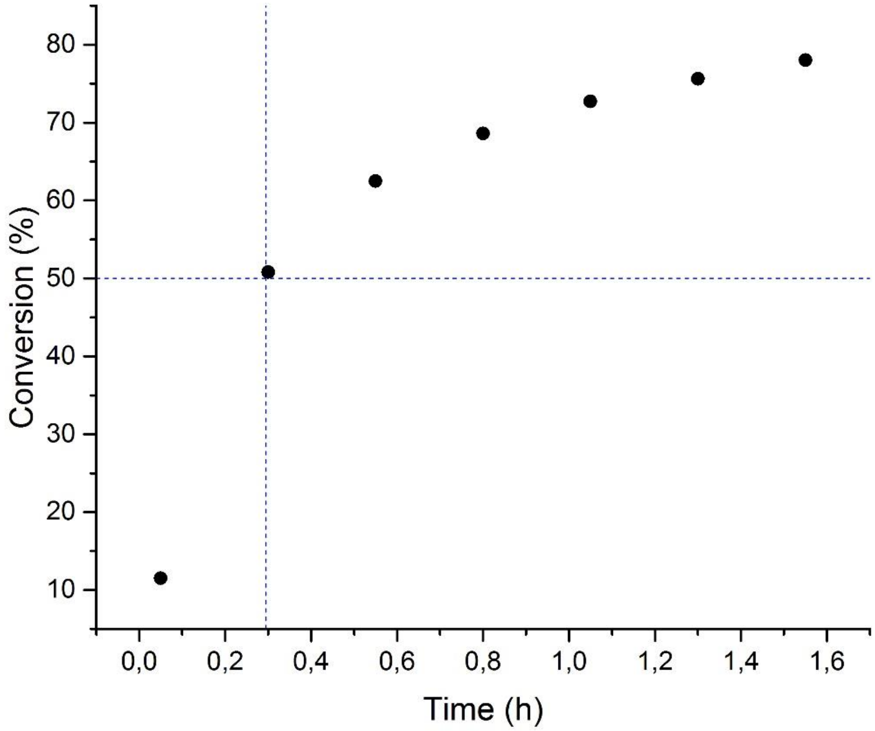

Figure S17. Plot for conversion (%) *vs* time (h) of the fragmentation of **12b**. % Conversion was determined by ^{1}H NMR (400 MHz, CD_3OD, 303 K).

6.3.9. Fragmentation of compound **13b**

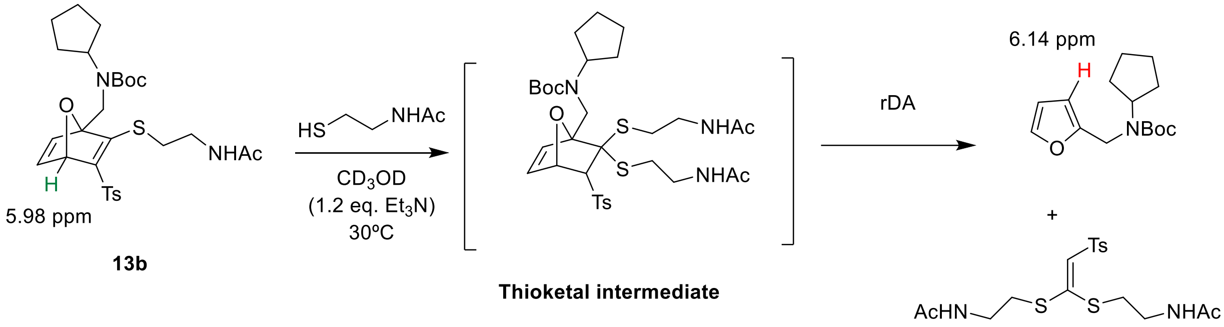

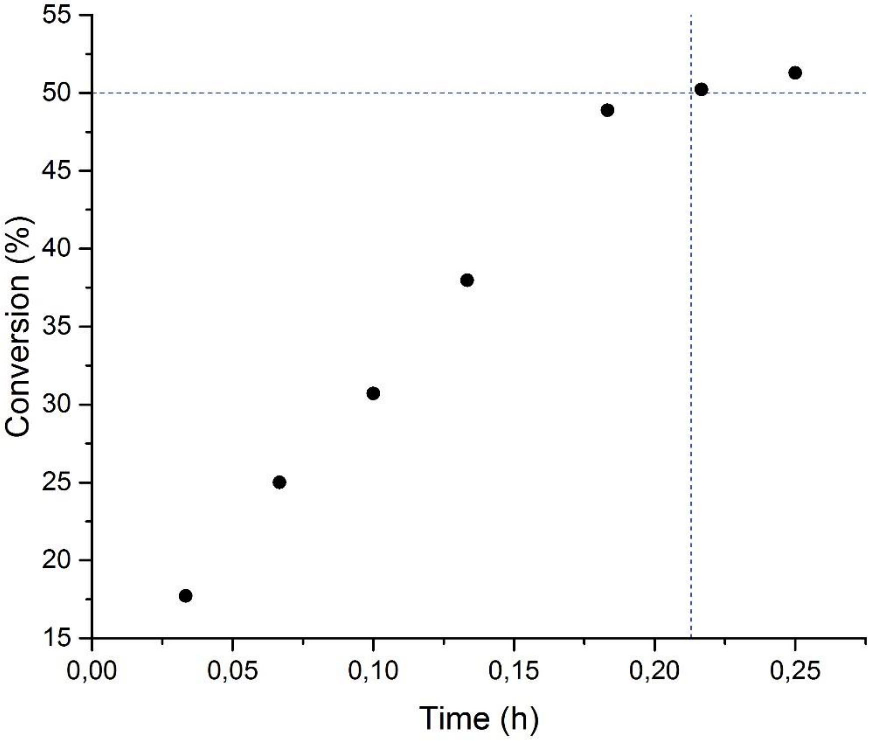

Figure S18. Plot for conversion (%) *vs* time (h) of the fragmentation of **13b**. % Conversion was determined by ^1H NMR (300 MHz, CD_3OD, 303 K).

6.3.10. Fragmentation of compound **14b**

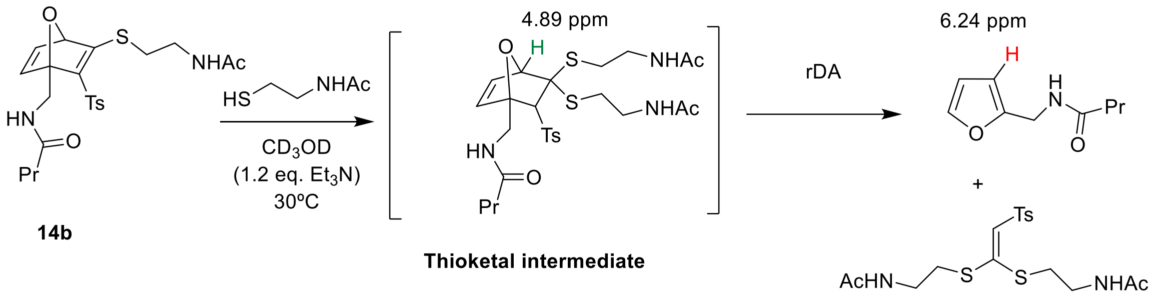

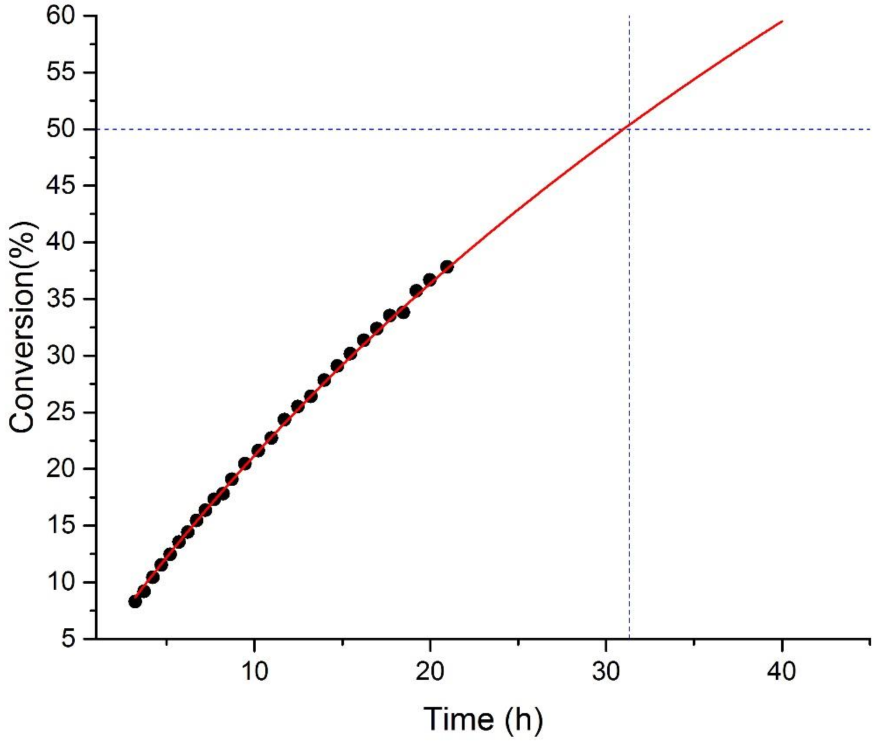

Figure S19. Plot for conversion (%) *vs* time (h) of the fragmentation of **14b**. % Conversion was determined by ^{1}H NMR (400 MHz, CD_3OD, 303 K).

6.3.11. Fragmentation of compound **15b**

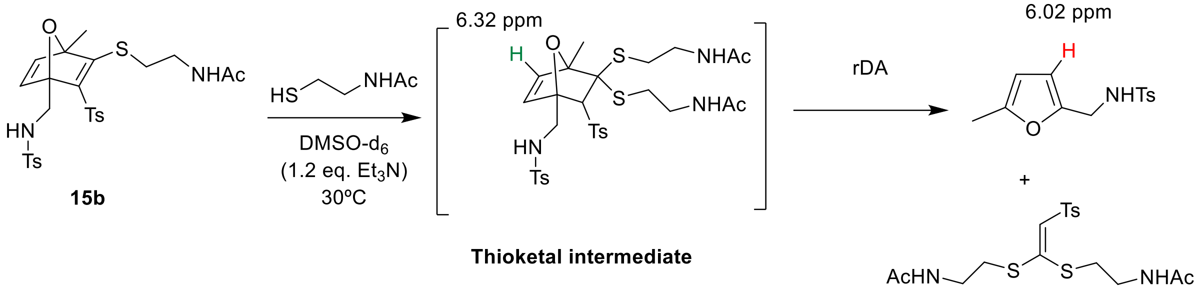

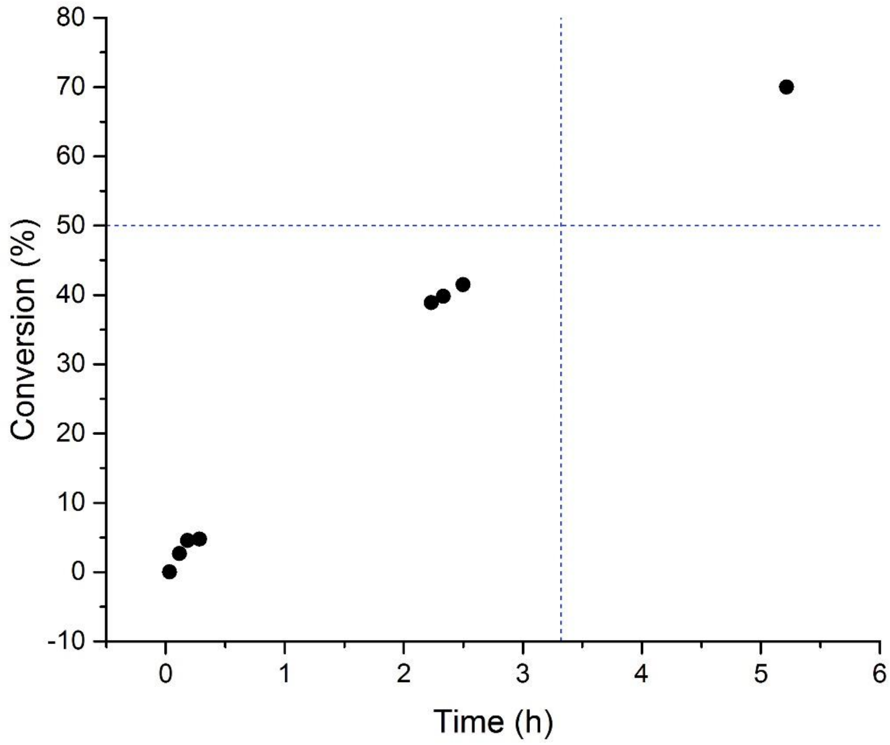

Figure S20. Plot for conversion (%) *vs* time (h) of the fragmentation of **15b**. % Conversion was determined by ^{1}H NMR (300 MHz, DMSO-d$_6$, 303 K).

6.3.12. Fragmentation of compound **16b**.

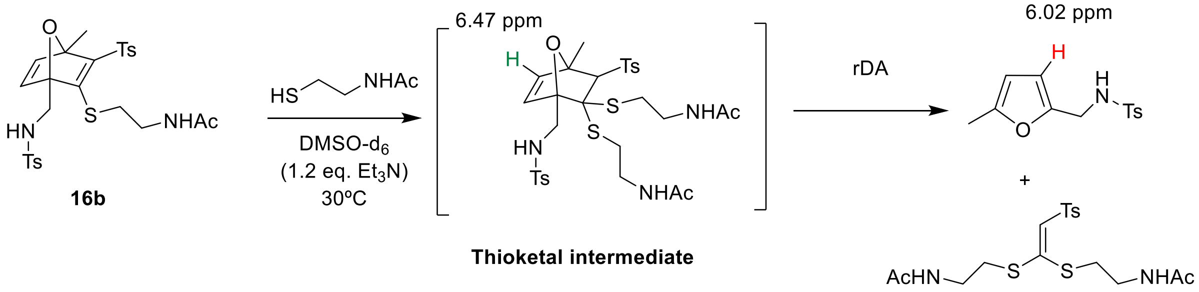

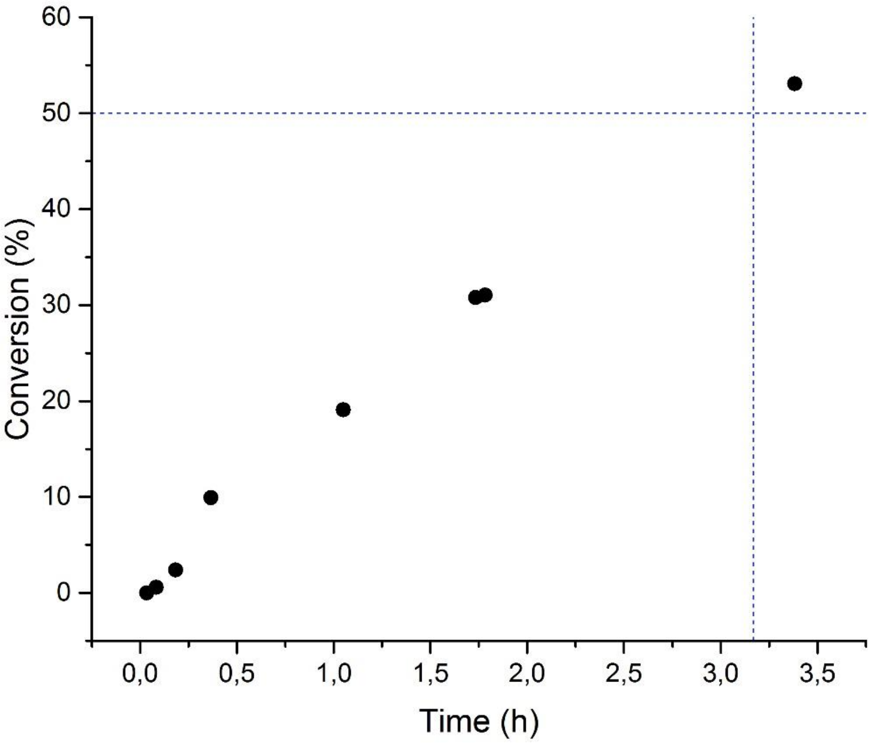

Figure S21. Plot for conversion (%) *vs* time (h) of the fragmentation of **16b**. % Conversion was determined by ^{1}H NMR (300 MHz, DMSO-d$_6$, 303 K).

6.3.13. Fragmentation of compound **17b**

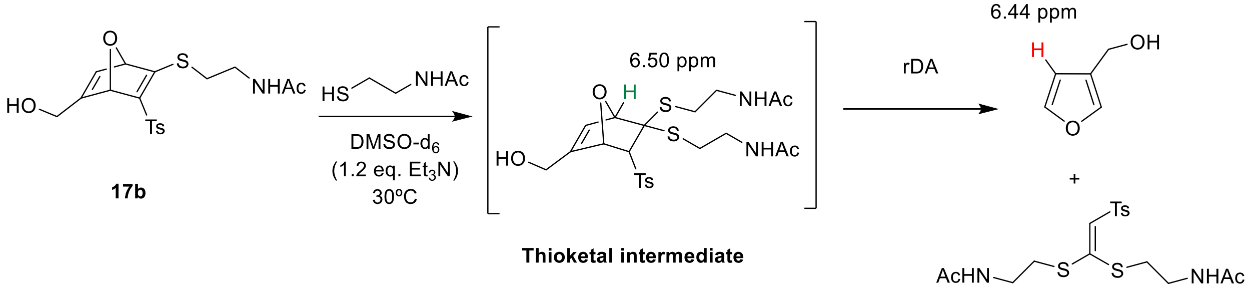

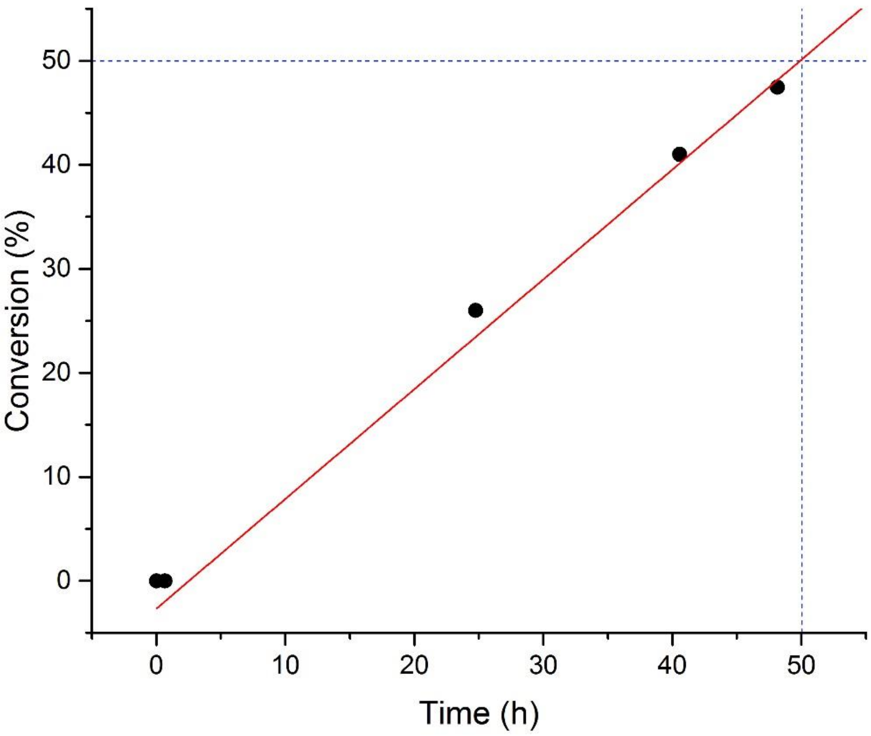

Figure S22. Plot for conversion (%) *vs* time (h) of the fragmentation of **17b**. % Conversion was determined by ^{1}H NMR (300 MHz, DMSO-d$_6$, 303 K).

6.3.14. Fragmentation of compound **18b**

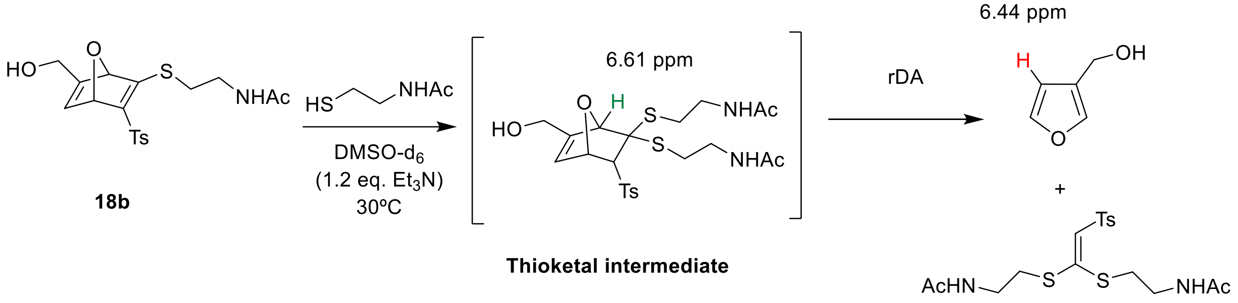

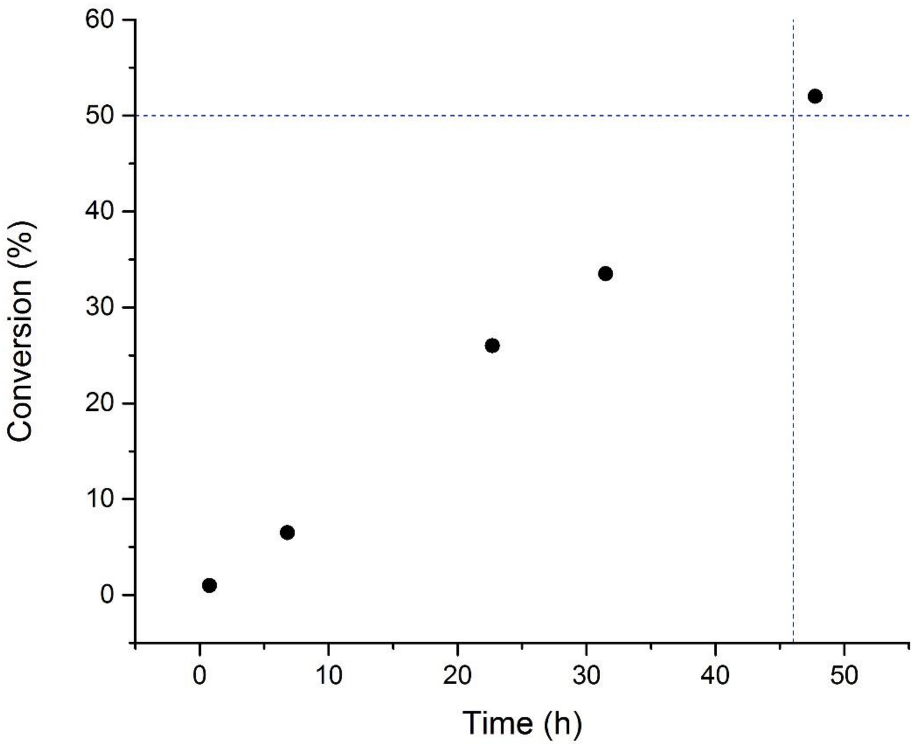

Figure S23. Plot for conversion (%) *vs* time (h) of the fragmentation of **18b**. % Conversion was determined by ^{1}H NMR (300 MHz, DMSO-d$_6$, 303 K).

6.4. Synthesis of *N*-(2-(((*rac*)-3-tosyl-7-oxabicyclo[2.2.1]hept-5-en-2-yl)thio)ethyl)acetamide (**23**)

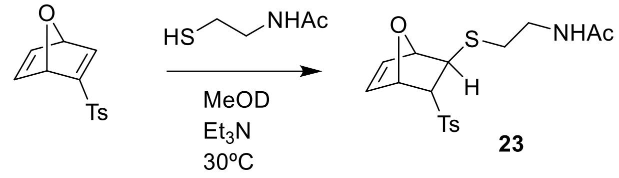

23

A solution of 2-[(4-Methylphenyl)sulfonyl]-7-oxabicyclo[2.2.1]hepta-2,5-diene[4] (100 mg, 0.40 mmol) in MeOH (1 mL), were added *N*-acetylcysteamine (72 mg, 0.60 mmol) in MeOH (0.5 mL) and Et_3N (0.84 mL, 0.60 mmol). The reaction mixture was stirred at r.t for 5 minutes. Then, the solvents were evaporated, and the residue was purified by chromatography column (AcOEt:Cy 10:1 → AcOEt) to obtain compound **23** (109 mg, 0.3 mmol, 74%) as a colourless oil. ^{1}H NMR (300 MHz, C_6D_6, δ ppm, *J* Hz): δ 7.64 (d, 2H, *J*=8.2 Hz, Ar-H), 6.79 (d, 2H, *J*=8.3 Hz, ArH), 6.38 (dd, 1H, *J*=5.3 Hz, *J*=1.5 Hz, H-5 or H-6), 6.18 (dd, 1H, *J*=6.0 Hz, *J*=1.5 Hz, H-5 or H-6), 5.50 (br s, 1H, NH), 4.71-4.68 (m, 2H, H-1, H-4), 3.63 (t, 1H, *J*=4.3 Hz, H-2 or H-3), 3.41-3.20 (m, 2H, H-1' or H-2'), 3.15 (d, 1H, *J*=4.4 Hz, H-2 or H-3), 2.69-2.42 (m, 2H, H-1' or H-2'), 1.89 (s, 3H, CH_3 of Ts), 1.64 (s, 3H, CH_3 of CH_3CO). ^{13}C NMR (75 MHz, C_6D_6, δ ppm): δ 169.0 (C=O), 144.2, 137.5 (C_qAr), 136.3, 133.4 (C-5 or C-6), 129.7, 127.9 (CAr), 86.5, 78.9 (C-1, C-4), 71.2, 46.1 (C-2, C-3), 39.1, 31.9 (C-1', C-2'), 22.5 (CH_3 of CH_3CO), 20.9 (CH_3 of Ts). HRMS (ESI) m/z: found, 390.0805; calcd. for $C_{17}H_{21}O_4NNaS_2$ $[M+Na]^+$: 390.0810.

7. Quantum mechanical calculations

Full geometry optimizations and transition structure (TS) searches were carried out with Gaussian 16[10] using the M06-2X hybrid functional[11] and 6-311+G(d,p) basis set with ultrafine integration grids. Bulk solvent effects in methanol were considered implicitly through the IEF-PCM polarizable continuum model.[12] The possibility of different conformations was taken into account for all structures. All stationary points were characterized by a frequency analysis performed at the same level used in the geometry optimizations from which thermal corrections were obtained at 303.15 K. Scaled frequencies were not considered. Mass-weighted intrinsic reaction coordinate (IRC) calculations were carried out using the Hessian-based predictor-corrector integrator scheme by Hratchian and Schlegel[13] in order to ensure that the TSs indeed connected the appropriate reactants and products. Gibbs free energies (ΔG) were used for the discussion on the relative stabilities of the considered structures. The lowest energy conformer for each calculated stationary point (Figure S25-S27) was considered in the discussion; all the computed structures can be obtained from authors upon request. Cartesian coordinates, electronic energies, entropies, enthalpies, Gibbs free energies, and lowest frequencies of the calculated structures are summarized in Table S1.

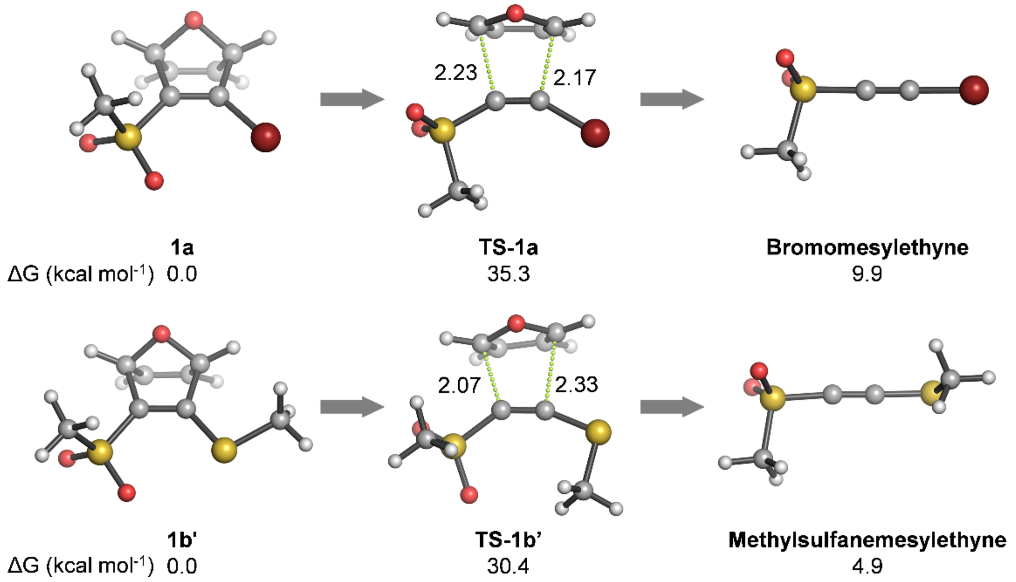

Figure S24. Minimum energy structures and Gibbs free energies (ΔG) for the rDA reaction of Br-OND **1a** and thio-OND **1b** calculated at the PCM(MeOH)/M06-2X/6-311+G(d,p) level. The thio-OND has been labelled as **1b'** to differentiate from the corresponding thioketal-OND intermediate also labelled as **1b**. Distances are given in angstrom.

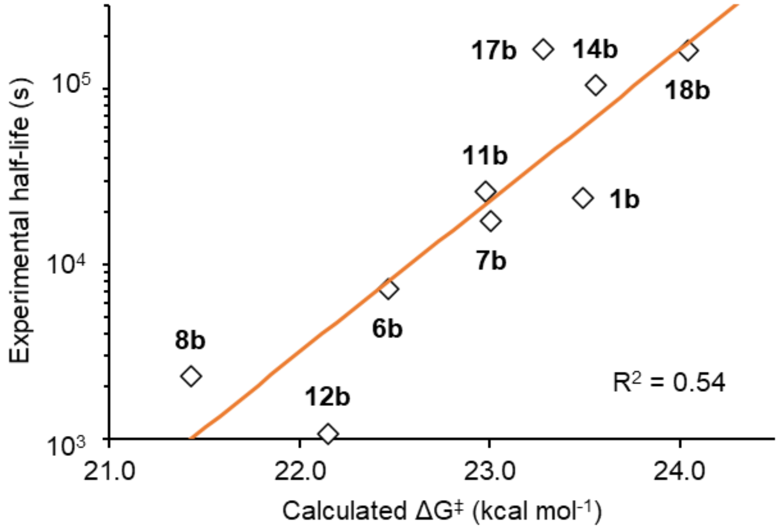

Figure S25. Observed half-life vs activation energies ($\Delta G^{\ddagger}$) calculated at the PCM/M06-2X/6-311+G(d,p) level for all retro-Diels–Alder reactions considered in this work.

OND derivative	ΔG_{rxn}	$\Delta G^{\ddagger}$
**1a**	9.9	35.3
**1b'**	4.9	30.4
**1b**	-10.4	23.5
**6b**	-12.4	22.5
**7b**	-10.5	23.0
**8b**	-12.1	21.4
**11b**	-10.9	23.1
**12b**	-11.4	22.2
**14b**	-10.4	23.6
**17b**	-7.0	23.2
**18b**	-7.7	240
**17b'**	-8.0	24.7
**18b'**	-7.7	24.7
**22**	-7.1	27.4
**23**	-11.8	29.9
**24**	-1.8	27.2

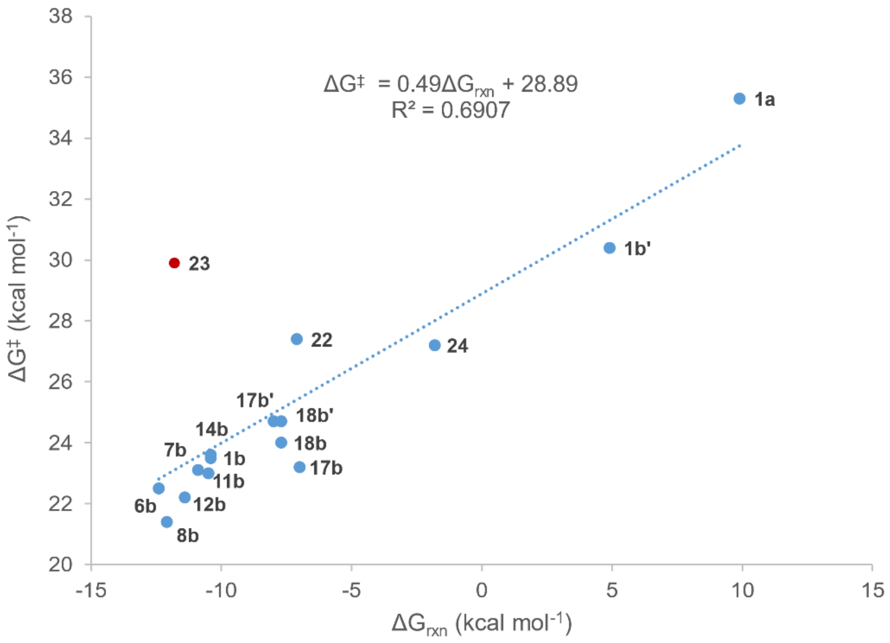

Figure S26 Bell-Evans-Polanyi (BEP) plot of the rDA reactions calculated at the PCM(MeOH)/M06-2X/6-311+G(d,p) level. Reaction from ketal derivative **23** deviates from the normal trend and is highlighted in red.

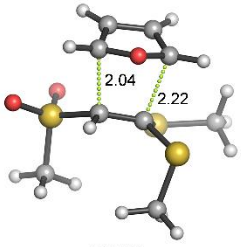

TS-1b
ΔG‡ = 23.5 kcal mol-1

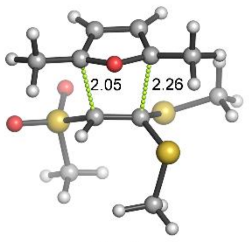

TS-6b
ΔG‡ = 22.5 kcal mol-1

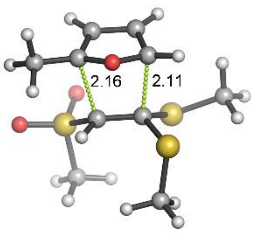

TS-7b
ΔG‡ = 23.0 kcal mol-1

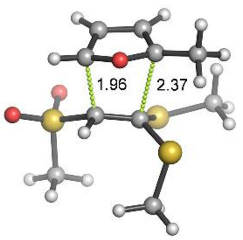

TS-8b
ΔG‡ = 21.4 kcal mol-1

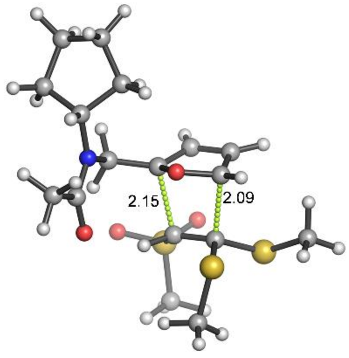

TS-11b
ΔG‡ = 23.0 kcal mol-1

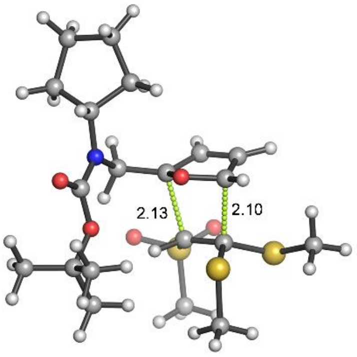

TS-12b
ΔG‡ = 22.2 kcal mol-1

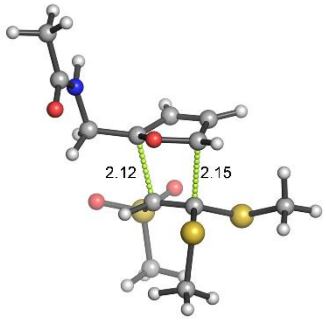

TS-14b
ΔG‡ = 23.6 kcal mol-1

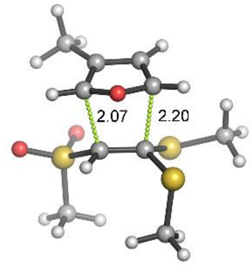

TS-17b'
ΔG‡ = 24.7 kcal mol-1

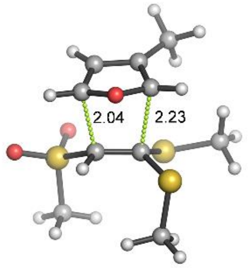

TS-18b'
ΔG‡ = 24.7 kcal mol-1

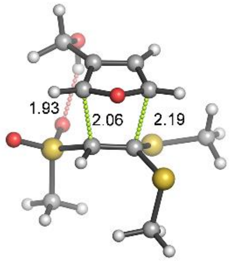

TS-17b
ΔG‡ = 23.3 kcal mol-1

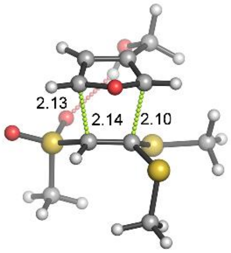

TS-18b
ΔG‡ = 24.0 kcal mol-1

Figure S27. Transition structures and activation free energies (ΔG‡) calculated at the PCM/M06-2X/6-311+G(d,p) level used for plots in figures 2A and S24. Distances are given in angstrom.

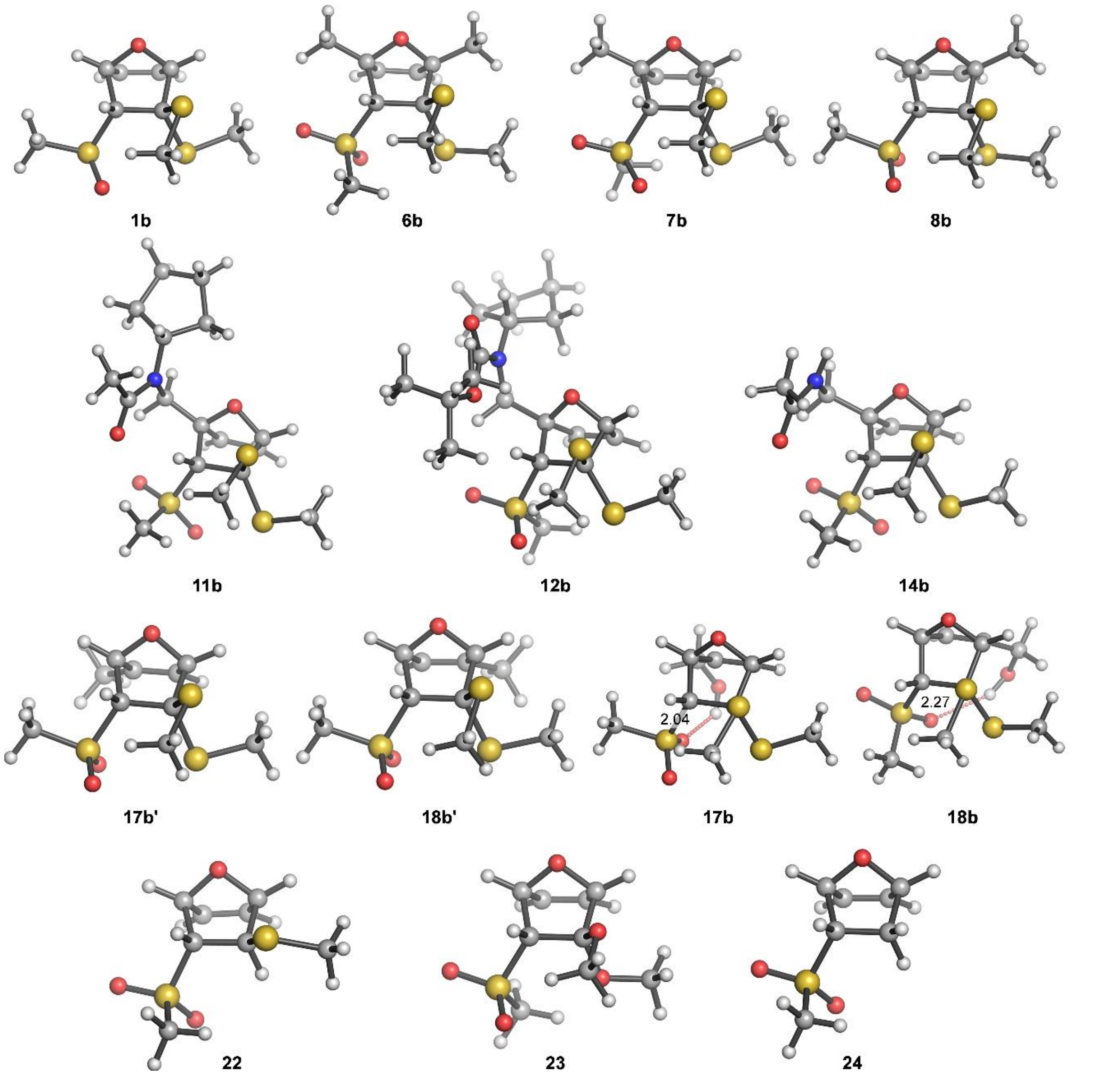

Figure S28. Minimum-energy structures for starting reagents calculated at the PCM/M06-2X/6-311+G(d,p) level. Distances are given in angstrom.

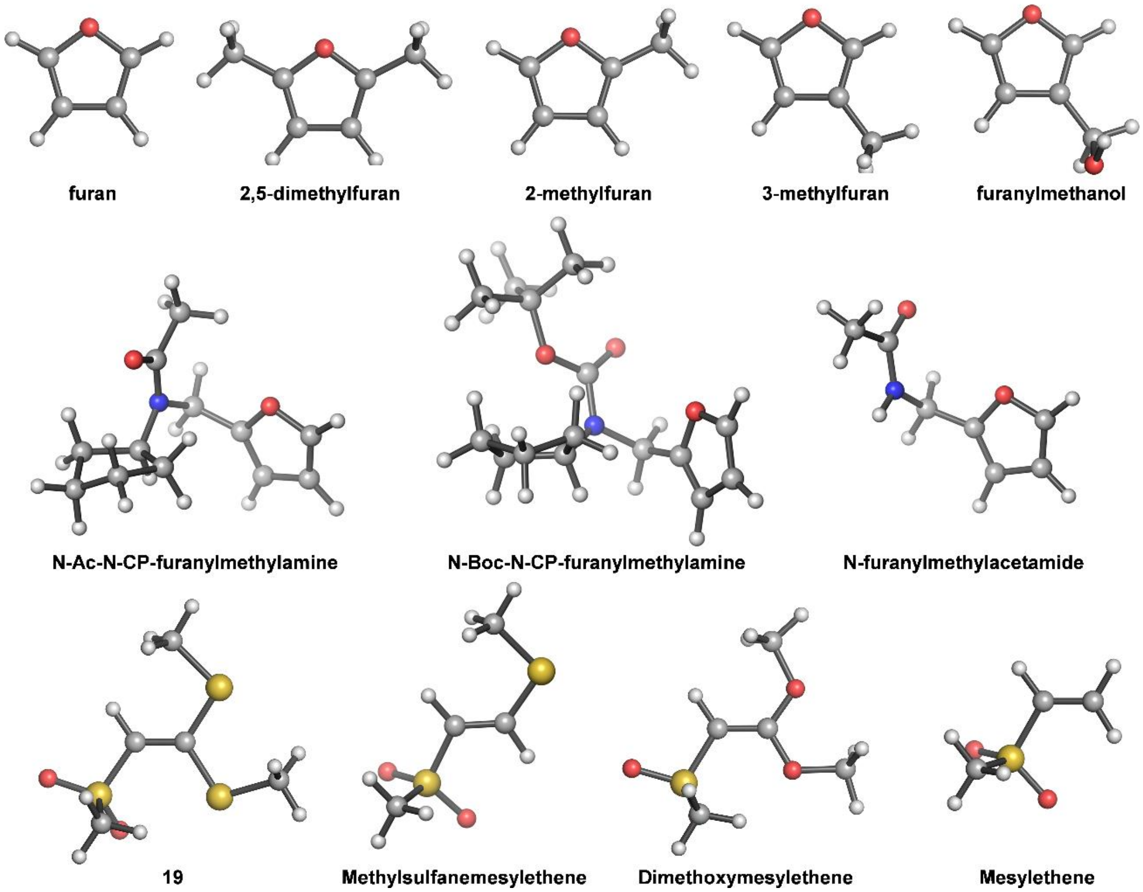

Figure S29. Minimum-energy structures for fragmentation products calculated at the PCM/M06-2X/6-311+G(d,p) level.

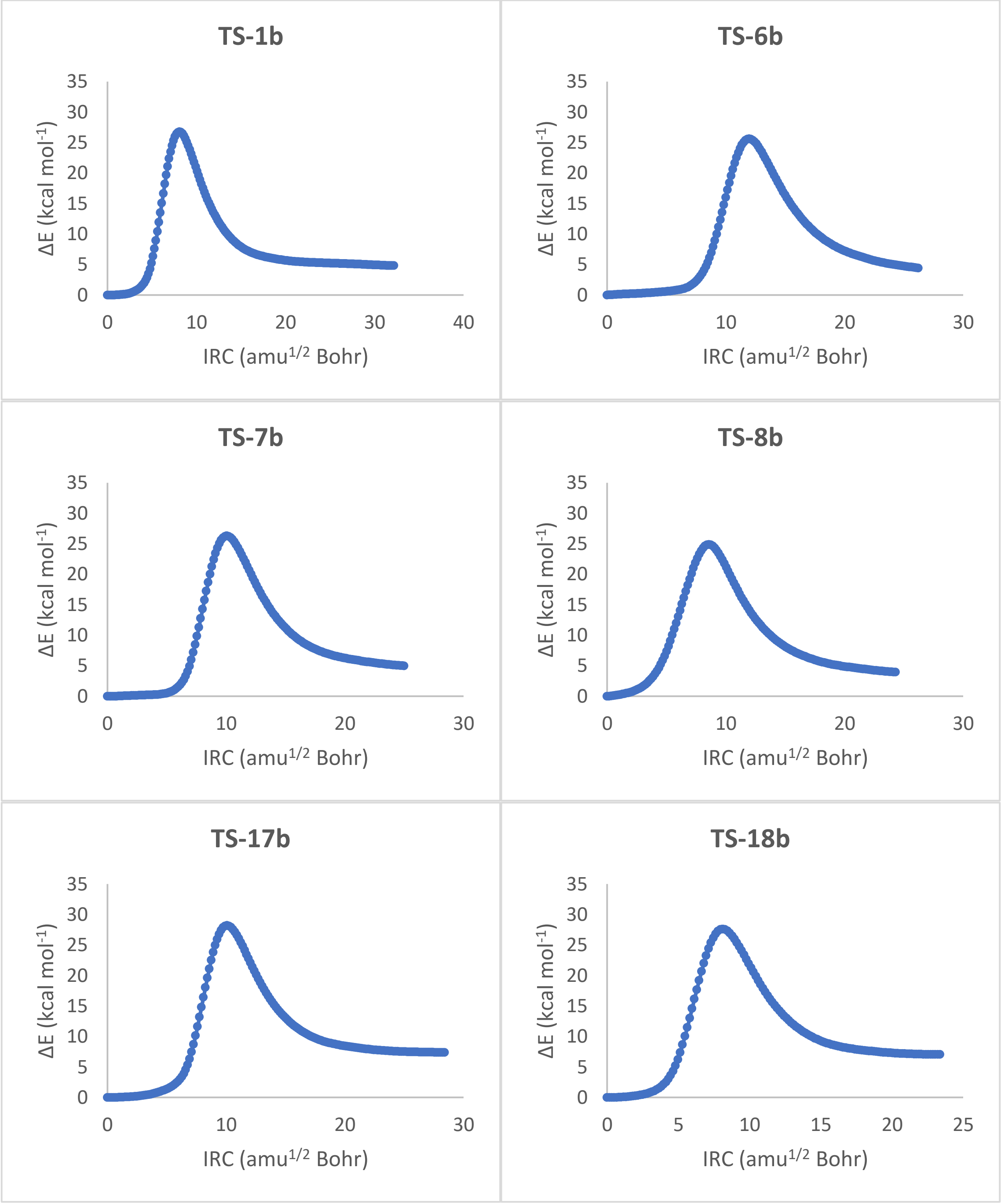

Figure S30. Intrinsic Reaction Coordinate (IRC) plots calculated at the PCM/M06-2X/6-311+G(d,p) level from the lowest energy rDA transition states **TS-1b**, **TS-6b**, **TS-7b**, **TS-8b**, **TS-17b**, **TS-18b**, **TS-22**, **TS-23** and **TS-24**. The zero of the relative electronic energy along the IRC has been arbitrarily set at the first calculated IRC point, which corresponds to the reactant OND intermediate.

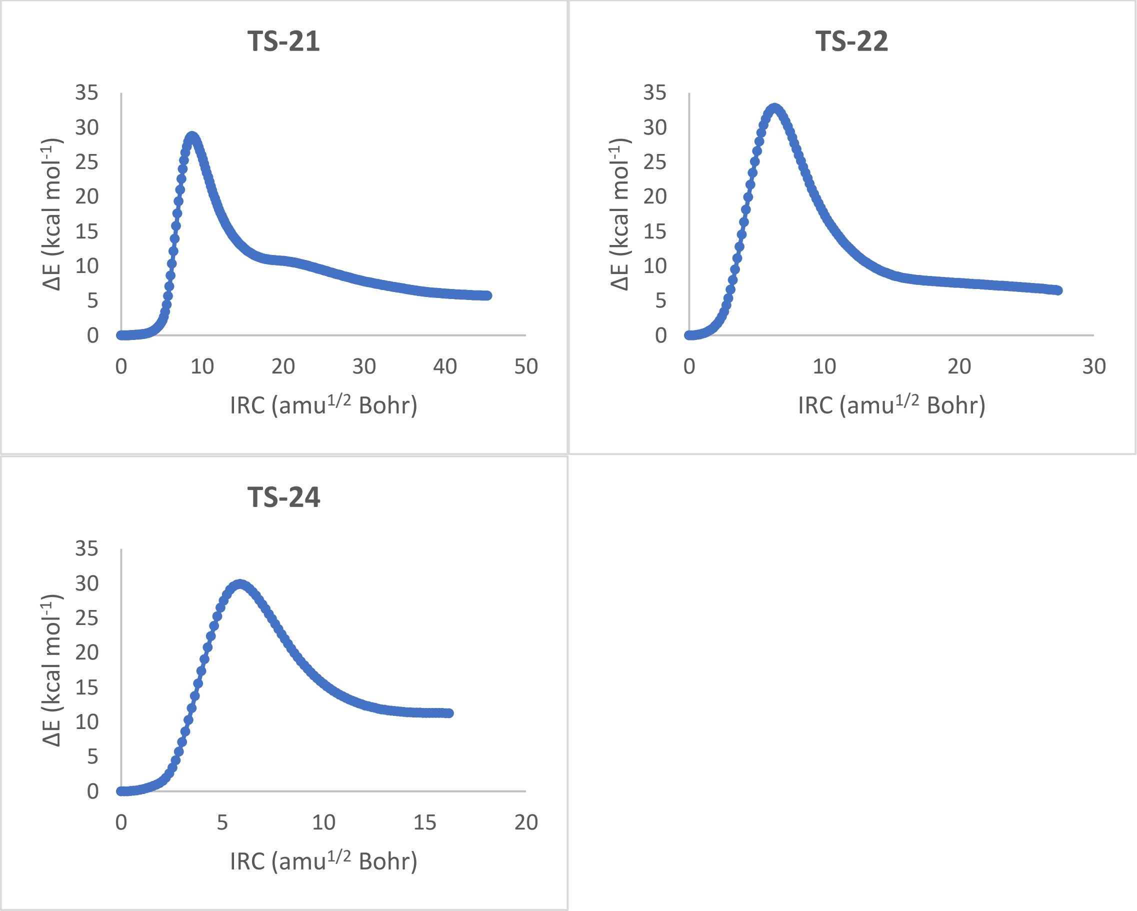

Figure S30 (cont.). Intrinsic Reaction Coordinate (IRC) plots calculated at the PCM/M06-2X/6-311+G(d,p) level from the lowest energy rDA transition states **TS-1b**, **TS-6b**, **TS-7b**, **TS-8b**, **TS-17b**, **TS-18b**, **TS-22**, **TS-23** and **TS-24**. The zero of the relative electronic energy along the IRC has been arbitrarily set at the first calculated IRC point, which corresponds to the reactant OND intermediate.

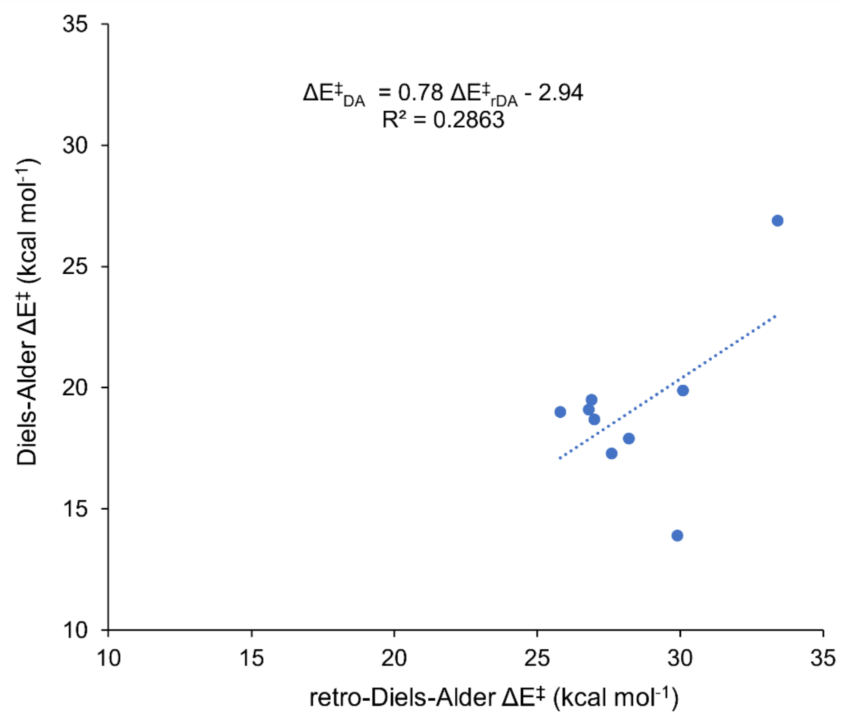

Figure S31. Activation electronic energies ($\Delta E^{\ddagger}_{DA}$) for the *forward* unimolecular retro-Diels-Alder ($\Delta E^{\ddagger}_{rDA}$) vs. the *reverse* bimolecular Diels-Alder ($\Delta E^{\ddagger}_{DA}$) reactions, calculated at the PCM(MeOH)/M06-2X/6-311+G(d,p) level. No significant correlation between both quantities is observed. One plausible explanation for this lack of correlation might be the inconsistent thermodynamics (i.e., stability of the separated fragments) calculated for some *forward* rDA reactions, which largely affect $\Delta E^{\ddagger}$ of the *reverse* DA reactions.

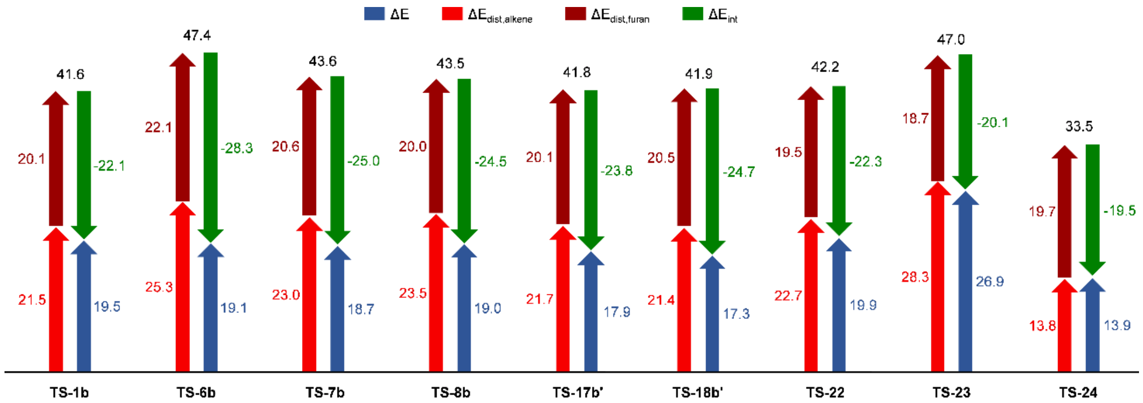

Figure S32. Distortion-interaction analysis[14-17] of the reverse bimolecular [4+2] Diels-Alder reaction between furan and alkene fragments to give oxanorbornene adducts, calculated at the PCM(MeOH)/M06-2X/6-311+G(d,p) level. Transition states **TS-1b**, methylated **TS-6b**, **TS-7b**, **TS-8b**, **TS-17b'**, **TS-18b'**, as well as **TS-22**, **TS-23**, and **TS-24** were included in the analysis. As in previous studies (refs. 4 and 6 in the main text), the presence of one methyl group at any of the bridgehead positions (**TS-7b**, **TS-8b**) causes an increase in the distortion energy (also known as deformation or strain) of ca. 2 kcal mol^{-1}. This is magnified by the presence of a second methyl group (**TS-6b**) where an increase of ca. 6 kcal mol^{-1} is observed. However, there is hardly any effect when a methyl group is placed at other positions (**TS-17b'**, **TS-18b'**). On the other hand, each addition of a methyl group to any of the positions of the furan moiety results in an increase in the interaction energy as the diene becomes more electron rich. In the case of thioether derivative (**TS-22**), there is barely any effect on either the interaction or distortion energies with respect to **TS-1b**. However, ketal derivative (**TS-23**) displays a higher distortion energy, mainly focused on the dienophile, but also a lower interaction energy. Finally, **TS-24** shows a significant decrease in both distortion and interaction energies due to the lack of substituents. As expected, these results do not relate to the experimental reaction rates measured for the rDA reactions.

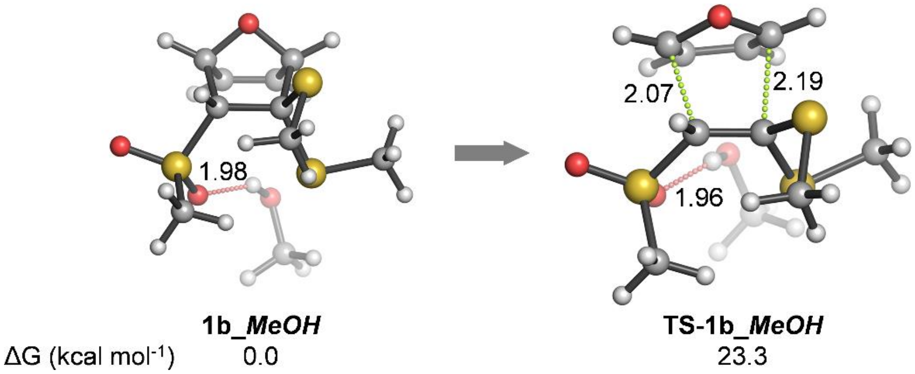

Figure S33. Minimum energy structures and activation Gibbs free energy for the rDA reaction of OND **1b** with one explicit MeOH molecule calculated at the PCM(MeOH)/M06-2X/6-311+G(d,p) level. To test the influence of explicit solvation, one explicit MeOH molecule was placed in different positions (i.e., near the two sulfonyl and the furan oxygen atoms, in different orientations). In the most stable optimized complex, where MeOH interacts with an oxygen of the sulfonyl group, no catalytic effect due partial protonation of the tosylate group through hydrogen bonding with methanol is observed ($\Delta G^{\ddagger}$ = 23.3 kcal mol^{-1} vs. $\Delta G^{\ddagger}$ = 23.5 kcal mol^{-1} for the unsolvated system). For all the other calculated complexes the activation energies are even higher.

Table S1. Energies, entropies, lowest frequencies of the lowest energy calculated structures.[a]

Structure	E_{elec} (Hartree)	E_{elec} + ZPE (Hartree)	H (Hartree)	S (cal mol^{-1} K^{-1})	G (Hartree)	Lowest freq. (cm^{-1})	# of imag freq.
**1b**	–1771.429073	–1771.203583	–1771.186013	127.6	–1771.247662	37.5	0
**TS-1b**	–1771.386315	–1771.164684	–1771.146369	132.2	–1771.210225	–508.5	1
**6b**	–1850.050688	–1849.769914	–1849.749491	137.0	–1849.815683	55.1	0
**TS-6b**	–1850.008885	–1849.731673	–1849.710231	144.2	–1849.779879	–464.2	1
**7b**	–1810.741703	–1810.488476	–1810.469499	131.9	–1810.533222	70.0	0
**TS-7b**	–1810.698751	–1810.449409	–1810.429494	138.8	–1810.496556	–494.1	1
**8b**	–1810.738607	–1810.485490	–1810.466406	133.3	–1810.530819	37.6	0
**TS-8b**	–1810.698283	–1810.449032	–1810.428966	140.1	–1810.496670	–458.4	1
**11b**	–2214.048804	–2213.616914	–2213.588571	178.4	–2213.674746	25.0	0
**TS-11b**	–2214.007101	–2213.578447	–2213.549425	183.3	–2213.637977	–506.5	1
**12b**	–2407.196955	–2406.675681	–2406.642198	202.3	–2406.739911	13.7	0
**TS-12b**	–2407.155821	–2406.638300	–2406.603936	208.4	–2406.704612	–515.7	1
**14b**	–2018.744572	–2018.435108	–2018.411524	156.3	–2018.487035	33.2	0
**TS-14b**	–2018.698502	–2018.393264	–2018.368448	167.8	–2018.449491	–491.4	1
**17b**	–1885.961766	–1885.702265	–1885.682196	139.1	–1885.749404	38.3	0
**TS-17b**	–1885.920308	–1885.664453	–1885.643903	141.7	–1885.712371	–503.6	1
**18b**	–1885.960817	–1885.701669	–1885.681674	137.9	–1885.748314	51.3	0
**TS-18b**	–1885.918629	–1885.662787	–1885.642330	140.1	–1885.710001	–523.5	1
**17b'**	–1810.739908	–1810.486619	–1810.467209	136.3	–1810.533051	36.6	0
**TS-17b'**	–1810.695686	–1810.446240	–1810.426196	139.6	–1810.493647	–499.4	1
**18b'**	–1810.740318	–1810.487113	–1810.467704	136.1	–1810.533440	42.4	0
**TS-18b'**	–1810.696788	–1810.447170	–1810.427266	138.4	–1810.494129	–503.4	1
**19**	–1541.424298	–1541.274488	–1541.260336	113.4	–1541.315109	45.7	0
**furan**	–229.993051	–229.922369	–229.917635	65.2	–229.949133	614.8	0
**2,5-dimethylfuran**	–308.615069	–308.488978	–308.480876	81.7	–308.520336	137.7	0
**2-methylfuran**	–269.304204	–269.205820	–269.199427	73.5	–269.234923	141.7	0
**3-methylfuran**	–269.299980	–269.201455	–269.195043	73.6	–269.230614	137.3	0
**N-Ac-N-CP-furanylmethylamine**	–672.611227	–672.333572	–672.318211	121.6	–672.376961	26.3	0
**N-Boc-N-CP-furanylmethylamine**	–865.760448	–865.393637	–865.373165	144.5	–865.442971	21.2	0
**N-furanylmethylacetamide**	–477.304443	–477.149839	–477.138780	102.9	–477.188469	27.6	0
**furanylmethanol**	–344.518344	–344.414162	–344.406711	80.2	–344.445476	65.2	0
**22**	–1333.949214	–1333.752353	–1333.737948	115.1	–1333.793556	40.2	0
**TS-22**	–1333.901270	–1333.708344	–1333.693448	116.7	–1333.749819	–506.1	1
**Methylsulfanemesylethene**	–1103.939967	–1103.819302	–1103.808283	98.3	–1103.855755	50.5	0
**23**	–1125.489278	–1125.255274	–1125.239283	119.4	–1125.296979	50.1	0
**TS-23**	–1125.436120	–1125.206531	–1125.189767	123.3	–1125.249346	–554.2	1
**Dimethoxymesylethene**	–895.485902	–895.327917	–895.314897	107.2	–895.366668	51.1	0
**24**	–896.461538	–896.293454	–896.282511	98.2	–896.329936	29.9	0
**TS-24**	–896.413825	–896.249954	–896.238462	99.7	–896.286606	–534.0	1
**Mesylethene**	–666.442995	–666.352361	–666.344304	81.6	–666.383742	96.9	0
**1a**	–3468.774992	–3468.641574	–3468.629137	111.4	–3468.682935	6.2	0
**TS-1a**	–3468.714770	–3468.584754	–3468.571456	114.5	–3468.626749	–384.6	1
**Bromomesylethyne**	–3238.740028	–3238.682275	–3238.672951	93.4	–3238.718068	52.7	0
**1b'**	–1332.702459	–1332.529601	–1332.515425	114.5	–1332.570754	31.1	0
**TS-1b'**	–1332.647418	–1332.478690	–1332.463345	121.9	–1332.522253	–374.8	1
**Methylsulfanemesylethyne**	–1102.671831	–1102.575108	–1102.563653	103.7	–1102.613756	26.0	0
**MeOH**	–115.710137	–115.658342	–115.653988	57.0	–115.681542	319.8	0
**1b_*MeOH***	–1887.150615	–1886.871563	–1886.849186	153.6	–1886.923370	28.5	0
**TS-1b_*MeOH***	–1887.108498	–1886.833354	–1886.810179	157.4	–1886.886202	–507.8	1

[a]Energy values calculated at the PCM(MeOH)/M06-2X/6-311+G(d,p). level. 1 Hartree = 627.51 kcal mol^{-1}. Thermal corrections at 303.15 K.

Cartesian coordinates of the lowest energy structures calculated with PCM(H2O)/M06-2X/6-31+G(d,p)

Structure **1b**

C	0.663059	2.475586	0.015679
C	0.745829	1.557005	-1.191640
C	-1.296160	1.503034	-0.584016
C	-0.611579	2.442771	0.391375
C	0.612106	0.070503	-0.707128
H	0.724300	-0.587614	-1.570099
C	-0.872968	0.022548	-0.212648
H	1.532687	1.727977	-1.920178
H	-2.361095	1.627012	-0.761576
O	-0.543530	1.708017	-1.776540
H	-1.074109	2.903489	1.251803
H	1.501184	2.959593	0.492081
S	1.920338	-0.506909	0.403516
O	1.581014	-1.875318	0.797932
O	2.197221	0.470600	1.454832
C	3.338568	-0.569201	-0.673745
H	3.562302	0.436498	-1.025776
H	4.154342	-0.945151	-0.056350
H	3.131346	-1.252905	-1.494529
S	-1.875447	-1.085963	-1.281827
C	-1.105303	-2.682625	-0.914127
H	-1.126811	-2.866777	0.158741
H	-1.699328	-3.434147	-1.432640
H	-0.077725	-2.719657	-1.275556
S	-1.092476	-0.366306	1.564517
C	-2.800253	0.206648	1.768068
H	-3.443022	-0.265087	1.025354
H	-3.107201	-0.112398	2.762984
H	-2.872953	1.292019	1.705729

Structure **TS-1b**

C	0.862354	2.432982	0.350546
C	0.869032	1.940246	-0.984268
C	-1.193711	2.042438	-0.399136
C	-0.457839	2.489127	0.718621
C	0.543287	-0.052511	-0.683047
H	0.640317	-0.319383	-1.731970
C	-0.758970	-0.116344	-0.136594
H	1.675682	1.975501	-1.701506
H	-2.253164	2.121529	-0.594376
O	-0.386651	2.073828	-1.494159
H	-0.865227	2.701502	1.694649
H	1.735146	2.583741	0.964869
S	2.037825	-0.587946	0.121875
O	2.117122	-0.103321	1.499888
O	3.136753	-0.234990	-0.783414
C	1.900227	-2.364778	0.170006
H	1.833702	-2.734413	-0.851529
H	2.806480	-2.727352	0.654980
H	1.016558	-2.622769	0.753089
S	-2.042880	-0.669394	-1.271809
C	-1.732738	-2.459265	-1.251841
H	-2.512479	-2.922132	-1.856095
H	-0.757788	-2.682539	-1.684482
H	-1.792770	-2.835737	-0.230644
S	-0.954715	-0.545574	1.595659
C	-2.697594	-0.142855	1.859938
H	-3.344568	-0.786530	1.268210
H	-2.881577	-0.306724	2.921146
H	-2.886085	0.903544	1.618180

Structure **6b**

C	-1.119859	1.916360	-1.077402
C	-1.059094	1.623760	0.416987
C	0.994773	1.612506	-0.266768
C	0.138850	1.886531	-1.497557
C	-0.687552	0.088048	0.548785
H	-0.772904	-0.200821	1.598542
C	0.815852	0.071474	0.089841
O	0.201879	2.191108	0.769464
H	0.525171	1.969750	-2.503167
H	-2.031079	2.014423	-1.650463
S	-1.934163	-0.970160	-0.256098
O	-1.834117	-0.907870	-1.713337
O	-3.214789	-0.586869	0.347531
C	-1.574329	-2.635216	0.259690
H	-1.665197	-2.696015	1.342138
H	-2.339990	-3.244913	-0.221097
H	-0.582958	-2.907812	-0.095512
S	1.888842	-0.278304	1.537854
C	1.410669	-1.976935	1.931137
H	1.519221	-2.625545	1.061456
H	2.096180	-2.306881	2.710997
H	0.393932	-2.027066	2.318970
S	1.173083	-1.036575	-1.345454
C	2.957263	-1.338791	-1.208813
H	3.195416	-1.975113	-0.359414
H	3.212001	-1.857948	-2.133230
H	3.523937	-0.412976	-1.151413
C	-2.151904	2.146155	1.311408
H	-2.182846	3.233646	1.227207
H	-3.115386	1.733154	1.019339
H	-1.950865	1.875812	2.349715
C	2.391717	2.177424	-0.300384
H	2.923444	2.013662	0.636778
H	2.956712	1.725556	-1.117023
H	2.328925	3.250879	-0.485453

Structure **TS-6b**

C	0.414670	2.129324	1.078472
C	0.577297	2.037227	-0.338258
C	-1.521529	1.626452	0.081121
C	-0.901253	1.859502	1.333971
C	0.629912	-0.006848	-0.539319
H	0.818624	0.012702	-1.609869
C	-0.657465	-0.449277	-0.141139
O	-0.673724	2.063835	-0.891169
H	-1.376951	1.736117	2.295241
H	1.220959	2.253681	1.783602
S	2.162815	-0.514216	0.227480
O	2.176212	-0.236576	1.664460
O	3.241659	0.068608	-0.578638
C	2.234713	-2.283520	0.004270
H	2.218373	-2.493622	-1.063488
H	3.181685	-2.597862	0.443526
H	1.394399	-2.744183	0.520877
S	-1.727806	-0.993315	-1.483890
C	-0.911433	-2.553153	-1.929345
H	-0.805569	-3.180341	-1.044069
H	-1.556086	-3.053215	-2.651811
H	0.059876	-2.360703	-2.384300
S	-0.808705	-1.351682	1.406010
C	-2.569969	-1.185767	1.774662
H	-3.162588	-1.398546	0.885634
H	-2.789328	-1.924958	2.543870
H	-2.792786	-0.191587	2.159055
C	1.656078	2.650705	-1.171584
H	1.460625	3.720171	-1.277757
H	2.622658	2.509111	-0.692919
H	1.687798	2.198980	-2.163407
C	-2.972781	1.674543	-0.261572
H	-3.562329	1.126209	0.470604
H	-3.306358	2.715810	-0.262096
H	-3.156477	1.257573	-1.251192

Structure **7b**

C	1.122557	1.975759	0.927977

C	1.128040	1.512654	-0.524914
C	-0.937751	1.787781	0.001352
C	-0.154618	2.138615	1.255514
C	0.570754	0.035362	-0.570381
H	0.628966	-0.298405	-1.610385
C	-0.929111	0.218609	-0.166116
H	-1.915349	2.243189	-0.133569
O	-0.037363	2.173127	-1.030745
H	-0.580140	2.406629	2.211738
H	2.001107	2.097368	1.545331
S	1.578604	-1.262477	0.191750
O	2.822495	-1.291467	-0.583260
O	0.759729	-2.476168	0.215392
C	2.004118	-0.845551	1.872363
H	1.106781	-0.553909	2.413211
H	2.422253	-1.769510	2.273692
H	2.756112	-0.061267	1.861764
S	-2.039320	-0.148859	-1.584379
C	-1.730483	-1.919898	-1.797315
H	-1.899763	-2.442032	-0.857022
H	-2.441253	-2.263882	-2.547673
H	-0.715371	-2.105996	-2.146949
S	-1.457515	-0.673267	1.345085
C	-2.948793	0.282427	1.731348
H	-3.596864	0.324613	0.856098
H	-3.456365	-0.262364	2.525866
H	-2.711512	1.285388	2.083728
C	2.342790	1.797421	-1.364780
H	2.546574	2.868991	-1.349462
H	3.208826	1.263858	-0.972579
H	2.173780	1.477974	-2.394479

Structure **TS-7b**

C	-1.329918	1.838391	-1.052291
C	-1.362045	1.758164	0.369687
C	0.692294	2.142485	-0.181045
C	-0.033022	2.109042	-1.396829
C	-0.455632	-0.192835	0.553264
H	-0.654150	-0.241496	1.619993
C	0.876015	0.067269	0.151867
H	1.673419	2.555754	0.005454
O	-0.201822	2.281030	0.842391
H	0.396044	2.138087	-2.386319
H	-2.160823	1.608435	-1.700210
S	-1.573422	-1.315981	-0.243573
O	-1.618380	-1.114673	-1.692730
O	-2.843118	-1.226283	0.487491
C	-0.892971	-2.938216	0.060861
H	-0.853988	-3.097111	1.136956
H	-1.575784	-3.645857	-0.409694
H	0.096655	-2.993128	-0.390972
S	2.056598	0.200407	1.518822
C	2.175768	-1.547073	1.994067
H	2.456725	-2.147854	1.129198
H	2.955324	-1.616044	2.752211
H	1.231285	-1.894507	2.411823
S	1.496326	-0.703697	-1.353944
C	2.988238	0.270910	-1.661598
H	3.661782	0.224661	-0.808262
H	3.464004	-0.181113	-2.530931
H	2.734806	1.305634	-1.891573
C	-2.539988	1.828572	1.279827
H	-2.880039	2.864482	1.352703
H	-3.346230	1.211954	0.887552
H	-2.276922	1.475615	2.277107

Structure **8b**

C	0.718869	2.387935	0.550888
C	0.736816	1.774456	-0.838500
C	-1.269736	1.486419	-0.128869
C	-0.518467	2.194366	0.992707
C	0.700195	0.215333	-0.687392
H	0.791191	-0.236131	-1.676317
C	-0.752404	-0.022536	-0.134685
H	1.458455	2.142144	-1.562145
O	-0.593995	1.990411	-1.286995
H	-0.936523	2.414159	1.964125
H	1.573283	2.794416	1.071151
S	2.122075	-0.490669	0.189238
O	2.102407	-1.939690	-0.017499
O	2.206649	0.014716	1.559248
C	3.498643	0.178602	-0.725409
H	3.547642	1.253277	-0.558614
H	4.382369	-0.308936	-0.313978
H	3.382127	-0.066928	-1.779596
S	-1.722949	-0.962550	-1.372226
C	-0.831544	-2.538588	-1.356917
H	-0.846665	-2.980509	-0.361056
H	-1.358734	-3.187101	-2.055700
H	0.201429	-2.414849	-1.679757
S	-0.801797	-0.794160	1.543835
C	-2.476047	-1.486405	1.636407
H	-2.596211	-2.340641	0.973773
H	-2.573822	-1.814304	2.671701
H	-3.238647	-0.740792	1.425925
C	-2.754723	1.728992	-0.195142
H	-3.204260	1.256398	-1.068516
H	-3.237546	1.348510	0.705892
H	-2.931445	2.804410	-0.246981

Structure **TS-8b**

C	-0.028882	-2.448938	0.519025
C	-0.213133	-2.039389	-0.834468
C	1.802786	-1.459264	-0.296006
C	1.234472	-2.039768	0.858916
C	-0.643075	-0.132537	-0.664664
H	-0.774477	0.059141	-1.727537
C	0.477476	0.493988	-0.068724
H	-0.941985	-2.417984	-1.536747
O	1.024345	-1.776201	-1.361719
H	1.697817	-2.065675	1.833522
H	-0.796914	-2.849539	1.160305
S	-2.295912	-0.176013	0.025230
O	-2.302980	-0.654741	1.407204
O	-3.115200	-0.911302	-0.944777
C	-2.816740	1.528131	0.025619
H	-2.803203	1.893677	-0.999295
H	-3.830686	1.534327	0.425366
H	-2.141911	2.093750	0.667359
S	1.461882	1.525697	-1.155979
C	0.373892	2.975130	-1.299039
H	-0.531626	2.719789	-1.848971
H	0.127977	3.350120	-0.305555
H	0.927542	3.737041	-1.847371
S	0.383192	0.979066	1.651184
C	2.130644	1.117863	2.092125
H	2.660204	1.726903	1.361131
H	2.161414	1.604081	3.066308
H	2.583937	0.130197	2.167521
C	3.225500	-1.135990	-0.590830
H	3.303202	-0.450241	-1.434111
H	3.703878	-0.688054	0.278709
H	3.761279	-2.054947	-0.844982

Structure **11b**

C	-0.442110	-0.282826	-2.414503
C	0.223268	-0.074797	-1.054641
C	-0.927963	-1.870851	-0.860893
C	-1.177515	-1.383827	-2.278168
C	-0.948192	0.392896	-0.097658
H	-0.535941	0.766889	0.835542
C	-1.719235	-0.948102	0.137916

H	-1.029209	-2.934559	-0.661623
O	0.398173	-1.418920	-0.620366
H	-1.873060	-1.824879	-2.976326
H	-0.394387	0.410192	-3.242479
S	-1.791058	1.869433	-0.779922
O	-3.006166	1.519037	-1.516217
O	-0.791254	2.649255	-1.514623
C	-2.257669	2.765015	0.683084
H	-1.350565	2.979850	1.246526
H	-2.736948	3.679385	0.334275
H	-2.963771	2.152264	1.243653
S	-1.288604	-1.567926	1.821160
C	-2.187903	-0.392338	2.860494
H	-3.251793	-0.399127	2.623695
H	-2.040707	-0.723199	3.887972
H	-1.781586	0.614737	2.756312
S	-3.529812	-0.935504	-0.166928
C	-3.984417	-2.567117	0.485184
H	-3.906926	-2.602459	1.569750
H	-5.023525	-2.706927	0.187029
H	-3.380111	-3.356465	0.040503
C	1.524820	0.708340	-0.991659
H	1.308983	1.752001	-1.214791
H	2.187503	0.316830	-1.764546
N	2.212373	0.614872	0.296626
C	1.917358	1.548558	1.243303
O	0.991245	2.345677	1.090734
C	3.427959	-0.209346	0.374383
C	3.322938	-1.638162	-0.169084
C	4.638700	0.403545	-0.371421
H	3.677644	-0.305416	1.429305
C	4.795659	-2.043821	-0.243691
H	2.884556	-1.646797	-1.169950
H	2.711684	-2.278113	0.467159
C	5.485675	-0.802740	-0.843854
H	5.195097	1.078014	0.282050
H	4.290320	0.996676	-1.220213
H	5.175465	-2.236184	0.764501
H	4.958738	-2.944455	-0.836587
H	6.529962	-0.724083	-0.540419
H	5.467681	-0.865296	-1.934083
C	2.741793	1.579193	2.511626
H	2.635454	0.646217	3.068435
H	3.801233	1.731413	2.300038
H	2.374210	2.401216	3.120198

Structure **TS-11b**

C	-0.066699	1.393757	-1.546097
C	-0.558486	0.445661	-0.601719
C	0.505765	1.938446	0.531727
C	0.586706	2.353377	-0.821415
C	1.316607	-0.525720	-0.193415
H	0.803728	-1.307414	0.362579
C	2.001653	0.472804	0.537067
H	0.667138	2.527675	1.423619
O	-0.486083	1.002994	0.628303
H	1.174787	3.177823	-1.193474
H	-0.123195	1.284667	-2.617994
S	1.854832	-1.219545	-1.731856
O	2.272430	-0.199350	-2.695467
O	0.800970	-2.149504	-2.160946
C	3.291177	-2.192982	-1.312682
H	2.994330	-2.948157	-0.587044
H	3.624409	-2.657148	-2.241124
H	4.056616	-1.529010	-0.912612
S	2.012726	0.201950	2.329713
C	3.092938	-1.254034	2.406593
H	4.033417	-1.044109	1.896934
H	3.288117	-1.449398	3.460658
H	2.598400	-2.117579	1.962266
S	3.507958	1.186052	-0.151210
C	3.683856	2.677418	0.856950
H	3.685573	2.425465	1.915810
H	4.643880	3.112287	0.582002
H	2.892084	3.392679	0.634854
C	-1.631670	-0.604094	-0.814455
H	-2.249543	-0.293982	-1.659504
H	-1.166039	-1.553570	-1.071940
N	-2.483731	-0.779009	0.348357
C	-2.067270	-1.648278	1.306133
C	-3.740701	-0.027725	0.394884
O	-0.985093	-2.225963	1.212951
C	-2.972843	-1.898357	2.491046
C	-3.597689	1.483661	0.148638
C	-4.797515	-0.513620	-0.636292
H	-4.148669	-0.147708	1.395438
H	-3.974050	-2.196153	2.176299
H	-2.527261	-2.692625	3.084319
H	-3.060161	-1.000914	3.106824
C	-5.018446	1.884182	-0.249504
H	-2.921544	1.680154	-0.689882
H	-3.209313	2.006535	1.023490
C	-5.428132	0.767793	-1.222867
H	-5.540084	-1.139716	-0.138408
H	-4.330996	-1.127603	-1.409048
H	-5.667635	1.874029	0.631938
H	-5.072501	2.878595	-0.694106
H	-6.508473	0.687570	-1.346452
H	-4.998448	0.976206	-2.206566

Structure **12b**

C	0.191861	-2.494733	0.421915
C	-0.192181	-1.029797	0.232926
C	0.983903	-1.557261	-1.480880
C	0.926445	-2.817864	-0.636893
C	1.116289	-0.167195	0.449988
H	0.818543	0.881962	0.382883
C	1.981675	-0.545918	-0.797221
H	1.127191	-1.665919	-2.552533
O	-0.264061	-0.937785	-1.187909
H	1.433713	-3.746849	-0.853751
H	-0.065973	-3.099860	1.279916
S	1.834330	-0.142426	2.110767
O	0.787826	0.412724	2.976192
O	3.095630	0.596898	2.008973
C	2.199528	-1.783563	2.706672
H	2.743054	-2.337638	1.945365
H	2.820855	-1.610401	3.586235
H	1.268724	-2.268506	2.987300
S	2.108651	0.886636	-1.949303
C	3.358648	1.895559	-1.108496
H	4.353969	1.489211	-1.275034
H	3.285814	2.894603	-1.538173
H	3.155623	1.937998	-0.037796
S	3.616763	-1.279015	-0.436250
C	3.956209	-2.032723	-2.049622
H	3.863467	-1.282377	-2.834560
H	4.985952	-2.383745	-2.005666
H	3.299348	-2.878993	-2.247212
C	-1.470873	-0.528417	0.929781
H	-1.199970	0.177058	1.713145
H	-1.957108	-1.378507	1.411447
N	-2.439203	0.070682	0.030318
C	-2.346252	1.371132	-0.356461
O	-3.174869	1.952307	-1.034330
C	-3.584390	-0.729649	-0.405660
C	-3.214440	-2.071757	-1.058671
C	-4.570438	-1.087263	0.745204
H	-4.102730	-0.119161	-1.141638
C	-4.480831	-2.905640	-0.862397
H	-2.384849	-2.545443	-0.523860
H	-2.909363	-1.950677	-2.098480

C	-4.867067	-2.592345	0.590045
H	-5.480411	-0.492747	0.646446
H	-4.139813	-0.850664	1.720282
H	-5.265645	-2.559017	-1.542787
H	-4.324670	-3.969902	-1.044353
H	-5.901928	-2.845674	0.823163
H	-4.225841	-3.171179	1.261774
O	-1.215034	1.934445	0.107559
C	-0.983535	3.375496	-0.014736
C	-0.893198	3.787846	-1.479889
C	-2.064485	4.137086	0.743262
C	0.370041	3.554857	0.662354
H	-0.191056	3.134786	-2.003932
H	-1.863540	3.742106	-1.969384
H	-0.515410	4.811720	-1.532735
H	-2.110127	3.793475	1.779379
H	-1.815589	5.200439	0.744821
H	-3.039827	4.008009	0.276646
H	0.612500	4.616962	0.727810
H	0.354657	3.132291	1.669757
H	1.151571	3.062934	0.078679

Structure **TS-12b**

C	0.131305	-2.506704	0.293482
C	-0.391493	-1.185620	0.167891
C	0.713488	-1.579840	-1.639997
C	0.813566	-2.761710	-0.864677
C	1.428373	-0.127578	0.512214
H	0.902682	0.822178	0.549910
C	2.168864	-0.418828	-0.658844
H	0.893752	-1.455648	-2.698308
O	-0.307376	-0.824424	-1.134551
H	1.428445	-3.616494	-1.098931
H	0.071713	-3.111241	1.185524
S	1.925286	-0.539944	2.160088
O	2.439768	-1.907287	2.251901
O	0.806555	-0.181818	3.042228
C	3.269677	0.580046	2.509587
H	2.896021	1.598995	2.419731
H	3.585600	0.370967	3.531721
H	4.074559	0.381465	1.802584
S	2.187944	0.919759	-1.878779
C	3.517264	1.947957	-1.193661
H	4.463351	1.407698	-1.214072
H	3.585380	2.832145	-1.827347
H	3.281300	2.249873	-0.172779
S	3.680137	-1.392164	-0.516652
C	4.056237	-1.708543	-2.257210
H	4.252118	-0.782870	-2.794537
H	4.950460	-2.330742	-2.254775
H	3.241824	-2.255181	-2.732707
C	-1.500783	-0.540197	0.980450
H	-1.976592	-1.318573	1.580282
H	-1.085560	0.186375	1.672638
N	-2.507529	0.087592	0.148198
C	-2.396869	1.386043	-0.249626
C	-3.650717	-0.702965	-0.308819
O	-3.237693	1.972827	-0.906374
C	-3.316869	-2.097755	-0.849709
C	-4.706100	-0.967167	0.784474
H	-4.107910	-0.129739	-1.114849
C	-4.706567	-2.731064	-0.939112
H	-2.706832	-2.655115	-0.130510
H	-2.780534	-2.064909	-1.799454
C	-5.411490	-2.279339	0.360544
H	-5.391037	-0.123260	0.877967
H	-4.209741	-1.089650	1.750721
H	-5.231195	-2.329907	-1.810993
H	-4.673603	-3.815936	-1.043643
H	-6.482748	-2.142510	0.210147
H	-5.289050	-3.038569	1.135308
O	-1.244861	1.934396	0.175988
C	-0.984870	3.365775	0.005321
C	-0.905923	3.723482	-1.474688
C	-2.036712	4.179050	0.750726
C	0.381714	3.537047	0.657613
H	-0.220642	3.040408	-1.982389
H	-1.883194	3.675455	-1.950150
H	-0.513906	4.738776	-1.570411
H	-2.084543	3.864470	1.795932
H	-1.754642	5.233725	0.722560
H	-3.019239	4.068279	0.295260
H	0.656510	4.593208	0.666986
H	0.364229	3.169115	1.686469
H	1.138143	2.989556	0.090935

Structure **14b**

C	0.205720	1.519805	2.102945
C	-0.685945	0.473678	1.441614
C	1.154754	-0.534803	1.862417
C	1.358511	0.895153	2.333932
C	-0.090324	0.277902	-0.009168
H	-0.785569	-0.293811	-0.620053
C	1.196659	-0.559379	0.287582
H	1.741488	-1.317055	2.336807
O	-0.239122	-0.721342	2.076413
H	2.286498	1.302771	2.705946
H	-0.042452	2.564851	2.222207
S	-0.014468	1.875828	-0.897833
O	1.294444	2.516806	-0.778651
O	-1.170754	2.661888	-0.459081
C	-0.260656	1.392855	-2.590466
H	-1.223589	0.886710	-2.655854
H	-0.257886	2.316028	-3.169351
H	0.570663	0.752139	-2.884992
S	0.866590	-2.311628	-0.182535
C	0.858240	-2.190041	-1.987077
H	1.798280	-1.777217	-2.353111
H	0.738896	-3.207783	-2.356997
H	0.016617	-1.591101	-2.337976
S	2.769657	0.079471	-0.408470
C	3.875648	-1.316242	-0.058945
H	3.653524	-2.172884	-0.691676
H	4.875429	-0.944215	-0.282682
H	3.831757	-1.603894	0.990404
C	-2.196564	0.580394	1.529789
H	-2.536699	1.432780	0.945130
H	-2.472952	0.724987	2.574934
N	-2.848916	-0.622023	1.043560
C	-3.223235	-0.801075	-0.244241
O	-3.038064	0.053902	-1.106321
C	-3.858724	-2.129927	-0.570875
H	-3.153870	-2.704123	-1.175848
H	-4.122379	-2.707251	0.313917
H	-4.749769	-1.950489	-1.171739
H	-2.950566	-1.394975	1.683515

Structure **TS-14b**

C	0.827031	0.233895	-1.841192
C	1.255727	0.112153	-0.487500
C	0.202804	-1.707625	-0.958313
C	0.169194	-0.929662	-2.139036
C	-0.602695	0.457952	0.476249
H	-0.169459	0.469088	1.472664
C	-1.351029	-0.688042	0.117142
H	-0.000476	-2.761956	-0.836939
O	1.158222	-1.195807	-0.132621
H	-0.392008	-1.164705	-3.029962
H	0.907274	1.129383	-2.437400
S	-1.040461	2.133198	0.072610
O	-1.378763	2.281359	-1.343086
O	0.034477	2.981147	0.601217

C	-2.509742	2.460135	1.030362
H	-2.262250	2.354975	2.085140
H	-2.796279	3.486728	0.801714
H	-3.287389	1.761154	0.724605
S	-1.499357	-1.931796	1.420075
C	-2.736333	-1.125525	2.476173
H	-3.632844	-0.905474	1.896645
H	-2.981159	-1.830778	3.269866
H	-2.329443	-0.214060	2.913298
S	-2.769167	-0.491568	-0.974445
C	-3.117901	-2.211037	-1.411652
H	-3.376508	-2.793605	-0.530256
H	-3.966332	-2.175316	-2.093985
H	-2.265223	-2.657216	-1.923667
C	2.321798	0.906692	0.215023
H	2.155723	1.965398	0.024862
H	2.265882	0.731137	1.288994
N	3.649479	0.525851	-0.232454
C	4.432618	-0.344025	0.452823
O	4.081392	-0.852203	1.509390
C	5.779654	-0.640823	-0.161224
H	5.950735	-0.113519	-1.098419
H	6.553269	-0.364269	0.555954
H	5.849887	-1.715487	-0.332778
H	3.984616	0.899737	-1.107323

Structure **17b**

C	-1.653209	-1.296333	-0.701670
C	-0.896635	-0.373293	-1.647648
C	0.571023	-1.720608	-0.897529
C	-0.726748	-2.128837	-0.230345
C	0.000503	0.592915	-0.787583
H	0.483166	1.307313	-1.456614
C	1.076508	-0.388764	-0.212321
H	-1.446191	0.113043	-2.449545
H	1.344829	-2.473098	-1.024191
O	0.122063	-1.222187	-2.156725
H	-0.833687	-2.881832	0.537115
S	-0.871310	1.680419	0.369889
O	0.143058	2.465688	1.071492
O	-1.847286	0.942269	1.178108
C	-1.780643	2.760747	-0.714777
H	-2.514734	2.175657	-1.266462
H	-2.279158	3.475666	-0.060318
H	-1.081592	3.266515	-1.377938
S	2.728728	-0.026088	-0.930212
C	3.039899	1.614081	-0.229627
H	2.962807	1.575517	0.855674
H	4.054628	1.882015	-0.521276
H	2.342293	2.352325	-0.624555
S	1.140685	-0.505388	1.616127
C	2.064892	-2.057768	1.764581
H	2.983207	-2.001925	1.180641
H	2.315514	-2.157678	2.819583
H	1.464714	-2.914206	1.459038
C	-3.101433	-1.170585	-0.334115
H	-3.461046	-0.166744	-0.585200
H	-3.690622	-1.881034	-0.919027
O	-3.337073	-1.464690	1.027436
H	-2.824809	-0.812598	1.523933

Structure **TS-17b**

C	-1.722022	-1.413440	-0.575891
C	-1.118655	-0.774398	-1.698875
C	0.367440	-2.107376	-0.917518
C	-0.758220	-2.259906	-0.082315
C	0.113398	0.600872	-0.776395
H	0.432495	1.059166	-1.708816
C	1.067555	-0.195367	-0.102342
H	-1.607792	-0.212436	-2.481858
H	1.242986	-2.735086	-0.999013

O	-0.024399	-1.490258	-2.068195
H	-0.794019	-2.825765	0.835844
S	-1.011749	1.738892	-0.009814
O	-1.589866	1.181525	1.220284
O	-1.946503	2.175923	-1.050206
C	0.004594	3.121596	0.465076
H	0.458414	3.540514	-0.431164
H	-0.662116	3.842839	0.937359
H	0.753885	2.762561	1.170314
S	2.688462	-0.271869	-0.886250
C	3.356579	1.341734	-0.388548
H	4.382533	1.384708	-0.753133
H	2.777931	2.148405	-0.837953
H	3.353465	1.427535	0.698041
S	1.019072	-0.282708	1.693213
C	2.158489	-1.652255	2.003266
H	3.166636	-1.408515	1.675845
H	2.146212	-1.806659	3.081630
H	1.809449	-2.557981	1.506651
C	-3.077563	-1.113122	-0.005482
H	-3.418428	-0.137876	-0.370796
H	-3.789852	-1.862451	-0.358095
O	-3.090520	-1.169376	1.405418
H	-2.523608	-0.440527	1.696493

Structure **18b**

C	1.917364	-0.658009	1.183764
C	0.918296	0.210143	1.918145
C	-0.205486	-1.459716	1.219014
C	1.212791	-1.688603	0.717934
C	0.018468	0.887920	0.811556
H	-0.608596	1.654869	1.268145
C	-0.846026	-0.321494	0.325756
H	1.269388	0.891535	2.685025
H	-0.846426	-2.326162	1.364579
O	0.003974	-0.748615	2.433209
S	1.064056	1.832124	-0.337683
O	1.745544	0.956261	-1.295051
O	1.900504	2.671995	0.523379
C	-0.055983	2.875873	-1.244091
H	-0.561726	3.539941	-0.545512
H	0.571861	3.445798	-1.929273
H	-0.751176	2.242285	-1.793496
S	-2.571535	-0.133562	0.926736
C	-3.159334	1.223946	-0.114019
H	-3.082025	0.965669	-1.170109
H	-4.206579	1.371276	0.147223
H	-2.615656	2.145904	0.093680
S	-0.751016	-0.663181	-1.477309
C	-2.014717	-1.951442	-1.650324
H	-3.014372	-1.549133	-1.501924
H	-1.910364	-2.314815	-2.672385
H	-1.834905	-2.774553	-0.959613
C	1.634294	-2.704532	-0.299909
H	0.746370	-3.133995	-0.776215
H	2.181530	-3.522700	0.174034
H	2.936606	-0.384192	0.951888
O	2.501843	-2.133355	-1.264702
H	2.129067	-1.272646	-1.505579

Structure **TS-18b**

C	-1.878554	0.970581	1.149652
C	-1.121837	0.210972	2.075212
C	0.217392	1.699987	1.308428
C	-1.020936	1.918528	0.648137
C	-0.009092	-0.936343	0.707435
H	0.347586	-1.635274	1.457761
C	0.910073	0.008833	0.194527
H	-1.470261	-0.498899	2.810220
H	1.063814	2.368731	1.383908
O	-0.009625	0.913531	2.401324

S	-1.249931	-1.751652	-0.261326
O	-1.846613	-0.826541	-1.234441
O	-2.157801	-2.420762	0.674809
C	-0.364965	-3.001395	-1.173199
H	0.111291	-3.672805	-0.460870
H	-1.108768	-3.529666	-1.769542
H	0.365234	-2.503128	-1.810218
S	2.547262	-0.073234	0.953946
C	3.230425	-1.492254	0.050558
H	4.251408	-1.626937	0.406297
H	2.649725	-2.390530	0.259837
H	3.240995	-1.289208	-1.020285
S	0.865439	0.434749	-1.560648
C	2.134135	1.721838	-1.648744
H	3.129354	1.309567	-1.497428
H	2.053923	2.138469	-2.652530
H	1.942860	2.506108	-0.916756
C	-1.280687	2.806475	-0.532266
H	-1.774002	3.726838	-0.212034
H	-0.329816	3.087660	-0.996703
H	-2.869676	0.728547	0.799501
O	-2.144678	2.184360	-1.464405
H	-1.833969	1.274688	-1.589447

Structure **17b'**

C	-1.505382	-1.852765	-0.171628
C	-1.112885	-0.921845	-1.314957
C	0.721048	-1.768768	-0.645456
C	-0.349185	-2.369453	0.243607
C	-0.382846	0.344425	-0.737523
H	-0.164342	1.021101	-1.565387
C	0.958983	-0.268644	-0.210037
H	-1.860103	-0.705004	-2.074613
H	1.637419	-2.333135	-0.797797
O	0.017879	-1.580783	-1.874733
H	-0.174744	-3.038869	1.074164
S	-1.359685	1.399706	0.361852
O	-0.556430	2.592927	0.635977
O	-1.895182	0.655340	1.500209
C	-2.727968	1.865928	-0.680435
H	-3.328636	0.982148	-0.891169
H	-3.304165	2.585770	-0.099202
H	-2.345021	2.324620	-1.590230
S	2.378274	0.337367	-1.209968
C	2.420472	2.080020	-0.722515
H	2.623984	2.167571	0.343257
H	3.230013	2.534126	-1.292765
H	1.480585	2.577082	-0.961604
S	1.248600	-0.078316	1.587686
C	2.623843	-1.242476	1.784291
H	3.429834	-0.995238	1.094782
H	2.973071	-1.122165	2.808819
H	2.295855	-2.271630	1.639877
C	-2.899481	-2.070967	0.306137
H	-3.525679	-2.438914	-0.511386
H	-2.916161	-2.799964	1.116559
H	-3.336665	-1.140210	0.673068

Structure **TS-17b'**

C	-1.641189	-1.817599	-0.075333
C	-1.288338	-1.234802	-1.327773
C	0.493366	-2.191155	-0.617905
C	-0.486701	-2.421542	0.368705
C	-0.231018	0.438404	-0.723842
H	-0.119868	0.822709	-1.734218
C	0.930386	-0.090750	-0.119347
H	-1.956067	-0.865924	-2.093355
H	1.453544	-2.668821	-0.748540
O	-0.127871	-1.793125	-1.762906
H	-0.313470	-2.875806	1.332667
S	-1.449560	1.448404	0.079777
O	-1.749932	0.966959	1.427843
O	-2.569616	1.579649	-0.859466
C	-0.659456	3.039881	0.231363
H	-0.409067	3.401964	-0.764282
H	-1.384776	3.696587	0.711599
H	0.227273	2.918725	0.853326
S	2.425267	0.012472	-1.120898
C	2.842574	1.764131	-0.882516
H	3.789263	1.937860	-1.393323
H	2.072777	2.401302	-1.317568
H	2.958473	1.977258	0.180161
S	1.132708	0.037733	1.662535
C	2.527984	-1.080645	1.932915
H	3.426679	-0.718943	1.438321
H	2.682807	-1.103781	3.011101
H	2.284178	-2.084204	1.583199
C	-2.973545	-1.706108	0.593180
H	-2.862218	-1.634878	1.674476
H	-3.509278	-0.823385	0.243159
H	-3.579891	-2.585151	0.360188

Structure **18b'**

C	-0.363257	2.371335	0.438799
C	-0.688879	1.293473	1.455252
C	1.358444	0.996409	0.954457
C	0.919751	2.195576	0.124019
C	-0.759716	-0.096079	0.731999
H	-1.044081	-0.861436	1.456153
C	0.736815	-0.311809	0.315237
H	-1.492795	1.474828	2.162965
H	2.414460	0.911160	1.201125
O	0.558033	1.124732	2.127073
S	-2.067298	-0.247344	-0.511570
O	-2.128345	-1.655950	-0.909356
O	-1.934996	0.760671	-1.561940
C	-3.531822	0.128043	0.432269
H	-3.510175	1.178169	0.718562
H	-4.366829	-0.064287	-0.241399
H	-3.576935	-0.531028	1.297331
S	1.454745	-1.720515	1.252473
C	0.630708	-3.116837	0.446486
H	0.984927	-3.231246	-0.576824
H	0.890210	-4.000283	1.028542
H	-0.450939	-2.979418	0.447754
S	1.005533	-0.451457	-1.490795
C	2.809085	-0.627925	-1.529071
H	3.119691	-1.560462	-1.062215
H	3.078702	-0.636242	-2.584730
H	3.299672	0.217103	-1.045273
C	1.791619	2.926095	-0.838018
H	1.269791	3.790191	-1.249588
H	2.705756	3.267182	-0.344732
H	2.084840	2.274233	-1.664988
H	-1.068011	3.075298	0.020786

Structure **TS-18b'**

C	0.052000	-2.457051	-0.344099
C	-0.338382	-1.704440	-1.483989
C	1.675796	-1.118438	-1.040948
C	1.342102	-2.075786	-0.053155
C	-0.748735	0.127010	-0.692890
H	-1.091122	0.564016	-1.627055
C	0.496069	0.583923	-0.201945
H	-1.167425	-1.894071	-2.149904
H	2.651263	-0.726105	-1.293822
O	0.788347	-1.203581	-2.066512
S	-2.197784	-0.179444	0.291208
O	-1.885299	-0.943920	1.499263
O	-3.201371	-0.739630	-0.620955
C	-2.741876	1.442842	0.792379
H	-2.960819	2.024489	-0.101005

H	-3.641969	1.293557	1.388514
H	-1.951086	1.898585	1.387663
S	1.310989	1.829198	-1.214930
C	0.356572	3.299137	-0.736773
H	0.815351	4.151661	-1.236949
H	-0.679435	3.202241	-1.060801
H	0.407025	3.440787	0.342866
S	0.750780	0.679176	1.569763
C	2.531204	0.983397	1.651489
H	2.783793	1.963867	1.254281
H	2.792599	0.931415	2.708154
H	3.072052	0.208521	1.106879
C	2.193350	-2.477282	1.107519
H	1.963803	-1.862624	1.981869
H	2.001542	-3.518033	1.369987
H	3.253961	-2.367940	0.876696
H	-0.595298	-3.096059	0.236065

Structure **19**

C	-0.359476	0.843913	-0.103041
H	-0.373018	1.923097	-0.063291
C	0.770095	0.106848	-0.121755
S	-1.986681	0.179963	-0.108523
O	-2.091959	-0.905767	-1.087772
O	-2.895629	1.321302	-0.247754
C	-2.227549	-0.523178	1.511235
H	-2.137280	0.276855	2.243246
H	-3.231093	-0.948351	1.515665
H	-1.476387	-1.296773	1.662489
S	2.368262	0.829032	-0.044501
C	2.002040	2.588818	0.138196
H	1.416111	2.768295	1.038598
H	2.973819	3.069665	0.237262
H	1.495848	2.976188	-0.744523
S	0.734623	-1.654371	-0.282051
C	2.371054	-2.161865	0.318626
H	2.546167	-1.806391	1.331458
H	2.327532	-3.250209	0.318173
H	3.162767	-1.837865	-0.353241

Structure **furan**

C	-0.718278	0.954896	-0.000121
C	-1.091872	-0.347433	-0.000032
C	1.091875	-0.347422	-0.000017
C	0.718268	0.954903	0.000161
H	-2.047019	-0.845001	-0.000158
H	2.047028	-0.844979	0.000054
O	0.000006	-1.152147	0.000007
H	1.376074	1.808750	0.000267
H	-1.376094	1.808736	-0.000169

Structure **2,5-dimethylfuran**

C	-0.719728	1.296326	-0.000021
C	-1.104634	-0.003660	-0.000020
C	1.104628	-0.003638	0.000025
C	0.719722	1.296350	-0.000134
O	0.000029	-0.804874	-0.000024
H	1.379088	2.149890	-0.000213
H	-1.379149	2.149825	-0.000019
C	-2.424774	-0.685408	0.000023
H	-2.540055	-1.316846	0.883988
H	-3.219348	0.059850	-0.000070
H	-2.540017	-1.317032	-0.883813
C	2.424769	-0.685403	0.000114
H	2.539961	-1.316881	0.884062
H	2.540001	-1.317014	-0.883734
H	3.219389	0.059798	0.000075

Structure **2-methylfuran**

C	0.215471	1.161276	-0.000032
C	-0.637013	0.104602	-0.000021
C	1.394621	-0.730501	-0.000045
C	1.545118	0.614386	0.000008
H	2.086245	-1.556046	-0.000070
O	0.074305	-1.055795	0.000036
H	2.479094	1.152850	0.000019
H	-0.068333	2.201628	-0.000065
C	-2.117605	-0.018324	0.000034
H	-2.464779	-0.557458	0.884218
H	-2.565391	0.974557	-0.000148
H	-2.464828	-0.557809	-0.883912

Structure **3-methylfuran**

C	0.675918	0.007576	-0.000000
C	-0.132833	-1.080762	0.000001
C	-1.472033	0.641625	0.000000
C	-0.213412	1.141375	0.000002
O	-1.442616	-0.713046	-0.000004
H	0.061927	2.184586	0.000004
H	-2.449312	1.094834	0.000001
H	0.064047	-2.140384	-0.000002
C	2.172287	0.031115	0.000001
H	2.575757	-0.981996	-0.000007
H	2.554477	0.550881	-0.881079
H	2.554474	0.550866	0.881091

Structure **N-Ac-N-CP-furanylmethylamine**

C	2.527581	-1.275628	0.909673
C	1.917801	-0.097629	0.626239
C	3.137217	-0.594183	-1.123654
C	3.331101	-1.599249	-0.237082
H	3.516718	-0.393248	-2.111438
O	2.281211	0.328116	-0.613005
H	3.960601	-2.462856	-0.377690
H	2.414931	-1.843984	1.819474
C	0.923211	0.746258	1.353307
H	0.771996	0.322188	2.347113
H	1.302685	1.757097	1.487550
N	-0.359693	0.783789	0.651336
C	-0.688708	1.783883	-0.213131
C	-1.088401	-0.493684	0.601145
O	-1.652705	1.685851	-0.966802
C	0.160592	3.038726	-0.211063
C	-2.569273	-0.396469	1.020921
C	-1.071476	-1.204046	-0.783329
H	-0.574039	-1.134914	1.318570
H	1.182206	2.817979	-0.525604
H	-0.286768	3.738439	-0.912446
H	0.196982	3.496457	0.778956
C	-3.241459	-1.523441	0.230463
H	-2.976538	0.565598	0.709649
H	-2.687266	-0.491511	2.101722
C	-2.547222	-1.447856	-1.135974
H	-0.536250	-2.152717	-0.691410
H	-0.554407	-0.603146	-1.531736
H	-3.031374	-2.490169	0.701097
H	-4.325218	-1.410034	0.170288
H	-2.695360	-2.344081	-1.740844
H	-2.934877	-0.589451	-1.689233

Structure **N-Boc-N-CP-furanylmethylamine**

C	-3.861300	-0.157652	-0.455175
C	-2.634097	-0.731893	-0.528731
C	-3.390499	-1.154439	1.482179
C	-4.359499	-0.438497	0.863015
H	-3.301971	-1.587758	2.464331
O	-2.336732	-1.344393	0.647453
H	-5.305787	-0.143605	1.286758
H	-4.345263	0.403722	-1.238784
C	-1.572941	-0.750199	-1.578914
H	-1.319613	-1.771153	-1.859049

H	-1.953006	-0.231694	-2.461096
N	-0.353169	-0.091448	-1.115866
C	0.675429	-0.891329	-0.708772
C	-0.487721	1.323841	-0.740521
O	0.717875	-2.096390	-0.887831
C	-0.674875	1.579788	0.770196
C	0.661133	2.263841	-1.202248
H	-1.401774	1.643541	-1.245329
C	-0.167956	3.012658	0.949418
H	-0.043098	0.891672	1.337472
H	-1.712095	1.431825	1.080716
C	1.079319	3.044149	0.055509
H	0.278236	2.946163	-1.964302
H	1.487480	1.706297	-1.639861
H	-0.913500	3.724053	0.577925
H	0.043158	3.262920	1.990464
H	1.419953	4.055912	-0.170194
H	1.892066	2.516960	0.559778
O	1.640055	-0.180148	-0.107845
C	2.849560	-0.827232	0.398634
C	2.495440	-1.839898	1.482738
C	3.636947	-1.449827	-0.749102
C	3.624406	0.338069	1.000323
H	1.854668	-1.371776	2.234203
H	1.986605	-2.707740	1.068060
H	3.414127	-2.169032	1.973521
H	3.810330	-0.705525	-1.530046
H	4.606677	-1.784349	-0.373791
H	3.111460	-2.302277	-1.175145
H	4.570184	-0.020937	1.410020
H	3.835305	1.089445	0.236172
H	3.048384	0.802647	1.803744

Structure **N-furanylmethylacetamide**

C	-2.328232	0.962068	-0.222245
C	-1.131847	0.487207	0.206747
C	-2.411460	-1.261807	-0.102650
C	-3.169075	-0.185795	-0.422150
H	-2.595712	-2.322996	-0.096496
O	-1.170877	-0.868812	0.284108
H	-4.192560	-0.201863	-0.759877
H	-2.579439	1.999319	-0.377300
C	0.151406	1.148209	0.581803
H	0.004584	2.228384	0.556017
H	0.446871	0.863474	1.592287
N	1.248062	0.782323	-0.300842
C	2.221364	-0.089785	0.055741
O	2.275712	-0.602299	1.167066
C	3.256624	-0.388360	-1.002671
H	3.081608	0.142226	-1.937431
H	4.237596	-0.117039	-0.611048
H	3.255913	-1.462622	-1.190592
H	1.233342	1.133317	-1.246510

Structure **furanylmethanol**

C	-0.208885	0.136608	0.236679
C	0.700956	1.107225	-0.028706
C	1.809002	-0.772094	-0.065138
C	0.525677	-1.102238	0.208850
O	1.932620	0.572280	-0.210379
H	0.137129	-2.095184	0.371538
H	2.713151	-1.345372	-0.183688
H	0.625941	2.179327	-0.112134
C	-1.672977	0.319570	0.480911
H	-1.908540	1.387708	0.519454
H	-1.958858	-0.123589	1.435842
O	-2.471102	-0.339705	-0.497623
H	-2.223601	0.002083	-1.362576

Structure **22**

C	0.648890	2.307839	0.437667
C	0.421623	1.614034	-0.896045
C	-1.431905	1.446768	0.151835
C	-0.510088	2.211794	1.083751
C	0.338368	0.070060	-0.638765
H	0.256900	-0.438305	-1.602148
C	-0.951547	-0.047092	0.199354
H	-0.756782	-0.370826	1.222789
H	1.035825	1.898281	-1.743458
H	-2.503373	1.592636	0.247404
O	-0.961687	1.854604	-1.134497
H	-0.747415	2.498746	2.097957
H	1.595064	2.695526	0.785928
S	1.814586	-0.624390	0.122502
O	1.803313	-0.388694	1.567199
O	2.958221	-0.121811	-0.644553
C	1.647827	-2.374554	-0.153631
H	1.657101	-2.562083	-1.225526
H	2.505446	-2.839458	0.331912
H	0.715893	-2.704805	0.304551
S	-2.092695	-1.228297	-0.579032
C	-3.324538	-1.348250	0.746057
H	-3.808298	-0.387790	0.918540
H	-4.072297	-2.067961	0.416826
H	-2.858888	-1.707360	1.663179

Structure **TS-22**

C	0.720638	2.378964	0.210127
C	0.229206	1.868700	-1.015900
C	-1.441745	1.865172	0.330917
C	-0.350024	2.382624	1.067963
C	0.088220	-0.210949	-0.490743
H	-0.090394	-0.549771	-1.506679
C	-0.949033	-0.172222	0.446034
H	-0.699209	-0.265292	1.497035
H	0.694709	1.873224	-1.989426
H	-2.495316	1.926273	0.561786
O	-1.123862	1.916731	-0.994801
H	-0.355587	2.588913	2.126852
H	1.756539	2.580978	0.431071
S	1.723228	-0.605580	0.037493
O	1.885641	-0.218787	1.442964
O	2.679692	-0.062994	-0.935339
C	1.816034	-2.384351	-0.053252
H	1.615274	-2.691695	-1.078089
H	2.830540	-2.654341	0.239755
H	1.087212	-2.799316	0.640700
S	-2.554755	-0.829593	-0.009627
C	-2.158742	-2.603597	-0.045389
H	-1.796520	-2.931411	0.928370
H	-3.083830	-3.129136	-0.281313
H	-1.418069	-2.811311	-0.817052

Structure **Methylsulfanemesylethene**

C	-0.121063	-0.340659	-0.084068
H	-0.363642	-1.390941	-0.178765
C	-0.982363	0.672435	0.043468
H	-0.603502	1.687831	0.121934
S	1.601973	0.013352	-0.152152
O	1.784999	1.467026	-0.178544
O	2.187065	-0.777641	-1.238573
C	2.262080	-0.600771	1.383883
H	2.051087	-1.666732	1.447435
H	3.336352	-0.420392	1.350605
H	1.796574	-0.050525	2.198983
S	-2.708883	0.590045	0.093248
C	-2.999686	-1.186693	-0.069626
H	-2.551190	-1.723934	0.765320
H	-4.079322	-1.320761	-0.049613
H	-2.606104	-1.549842	-1.018455

Structure **23**

C	0.070025	2.353421	0.408889
C	0.137161	1.700768	-0.963451
C	-1.771751	1.158572	-0.166105
C	-1.113854	2.008377	0.907694
C	0.310381	0.159359	-0.781664
H	0.403223	-0.294334	-1.771627
C	-1.062681	-0.241349	-0.158394
H	0.784809	2.141430	-1.713631
H	-2.855406	1.104799	-0.202861
O	-1.231704	1.714477	-1.365997
H	-1.523362	2.216297	1.885626
H	0.861469	2.928581	0.867231
S	1.852377	-0.388942	-0.024853
O	2.902940	0.377808	-0.706323
O	1.889291	-1.848849	-0.142460
C	1.922734	0.025770	1.705868
H	1.059418	-0.402946	2.204622
H	2.853397	-0.432860	2.042468
H	1.968621	1.105005	1.815845
C	-1.301165	-2.389918	-1.146916
H	-1.370602	-2.939917	-0.205924
H	-1.921380	-2.869815	-1.900447
H	-0.258145	-2.384566	-1.473905
C	-2.094885	-1.044322	1.815779
H	-2.765405	-1.687182	1.242420
H	-1.811187	-1.540089	2.741104
H	-2.602926	-0.104711	2.050733
O	-1.806336	-1.064887	-1.001475
O	-0.883735	-0.803635	1.101981

Structure **TS-23**

C	-0.215160	2.441556	0.494449
C	-0.221643	2.136452	-0.883265
C	-1.967846	1.185838	-0.073367
C	-1.347901	1.857140	1.006778
C	0.347599	-0.013955	-0.740094
H	0.466323	-0.118893	-1.811071
C	-0.832702	-0.518195	-0.158609
H	0.403143	2.520035	-1.675510
H	-2.986313	0.834685	-0.151118
O	-1.438969	1.688312	-1.239418
H	-1.640410	1.785805	2.043113
H	0.589213	2.921816	1.025930
S	1.903960	-0.070004	0.056980
O	1.874875	0.547663	1.385252
O	2.880640	0.465428	-0.902937
C	2.250897	-1.806301	0.279548
H	2.285935	-2.274099	-0.702784
H	3.218345	-1.871002	0.777303
H	1.459269	-2.225721	0.898140
C	-1.263990	-2.501142	-1.334253
H	-1.065457	-3.098218	-0.440561
H	-2.065343	-2.958119	-1.910462
H	-0.357550	-2.438809	-1.941899
C	-1.936216	-1.359607	1.768167
H	-2.493642	-2.116300	1.214167
H	-1.652071	-1.748925	2.742555
H	-2.560905	-0.472919	1.898625
O	-1.716028	-1.190563	-0.979821
O	-0.718285	-1.029589	1.107644

Structure **Dimethoxymesylethene**

C	0.024450	-0.844087	-0.003566
H	0.014260	-1.917809	0.077609
C	-1.093659	-0.076255	0.008206
S	1.612840	-0.151002	-0.133462
O	1.715949	0.749331	-1.287211
O	2.551599	-1.279545	-0.080127
C	1.856555	0.836713	1.330269
H	1.792651	0.179753	2.195205
H	2.849132	1.278235	1.244840
H	1.087018	1.605665	1.352021
C	-2.535162	-1.946659	0.087494
H	-2.083399	-2.375136	0.982870
H	-3.611156	-2.086827	0.105281
H	-2.108357	-2.396183	-0.809676
C	-2.208753	2.024455	-0.100867
H	-2.822482	1.863222	0.783556
H	-1.869630	3.054715	-0.146039
H	-2.766568	1.767438	-0.999786
O	-2.332837	-0.529258	0.067597
O	-1.003147	1.244217	-0.022721

Structure **24**

C	1.413501	-1.384774	0.219943
C	1.115585	-0.523257	-0.995873
C	2.246845	0.726089	0.318481
C	2.124113	-0.606535	1.032881
C	0.053267	0.554756	-0.577478
H	-0.257075	1.109812	-1.463689
C	0.851173	1.423483	0.415479
H	0.908704	2.452843	0.066218
H	0.449871	1.402491	1.428102
H	0.919363	-1.011725	-1.944180
H	3.099444	1.355916	0.550746
O	2.259800	0.321757	-1.057042
H	2.457150	-0.806425	2.041361
H	1.020606	-2.375947	0.394569
S	-1.466748	-0.171180	0.057455
O	-1.340354	-0.477736	1.484317
O	-1.825151	-1.271303	-0.845629
C	-2.668219	1.131541	-0.117172
H	-2.778409	1.367639	-1.173581
H	-3.598624	0.742253	0.295207
H	-2.325020	1.992454	0.455234

Structure **TS-24**

C	1.465692	-1.312441	0.415340
C	1.418069	-0.826463	-0.909026
C	2.415811	0.695919	0.232421
C	2.118793	-0.347879	1.142825
C	-0.123669	0.721645	-0.634264
H	-0.253064	0.875775	-1.699464
C	0.601147	1.614014	0.160255
H	0.976341	2.515507	-0.309049
H	0.344126	1.710111	1.208093
H	1.138604	-1.338035	-1.816914
H	3.137630	1.490066	0.348790
O	2.300043	0.190941	-1.036206
H	2.248810	-0.295643	2.212632
H	0.962668	-2.195782	0.775012
S	-1.474828	-0.160629	0.053554
O	-1.283678	-0.290918	1.503561
O	-1.693054	-1.393113	-0.717091
C	-2.900697	0.880282	-0.209953
H	-2.741123	1.816267	0.321715
H	-3.016679	1.044623	-1.279630
H	-3.757423	0.341440	0.194246

Structure **Mesylethene**

C	1.199273	0.386566	-0.618023
H	1.153399	0.879545	-1.581695
C	2.294249	0.157631	0.087396
H	3.262440	0.471156	-0.283454
H	2.245384	-0.350200	1.044098
S	-0.372483	-0.175309	-0.016353
O	-0.163558	-0.908474	1.233453
O	-1.054483	-0.860532	-1.117537
C	-1.265891	1.319662	0.347772
H	-1.335372	1.909050	-0.564574
H	-2.254736	1.010523	0.685367
H	-0.732835	1.853769	1.131708

Structure **1a**

C	0.176053	2.571105	-0.912342
C	-0.439082	1.981692	0.373605
C	1.606489	1.435637	0.447563
C	1.456559	2.226542	-0.868335
H	-0.377640	3.097182	-1.674926
H	2.247665	2.387845	-1.584291
C	-0.436771	0.438984	0.192099
C	0.847702	0.098831	0.230004
H	-1.349629	2.427547	0.760667
H	2.587729	1.359488	0.903388
S	-1.869950	-0.486813	-0.201208
O	-2.701575	0.390222	-1.026482
O	-1.479651	-1.788871	-0.730967
C	-2.678344	-0.728526	1.363735
H	-2.024201	-1.329762	1.992458
H	-2.877599	0.246997	1.803955
H	-3.609074	-1.253794	1.148292
Br	1.722694	-1.513111	-0.035588
O	0.660228	2.107000	1.277124

Structure **TS-1a**

C	0.242527	2.522181	0.930163
C	0.664425	2.227948	-0.379340
C	-1.454851	1.900364	-0.365284
C	-1.121380	2.318480	0.937653
C	0.473945	0.021171	-0.130575
C	-0.758053	-0.142594	-0.110857
H	1.617153	2.393049	-0.859550
H	-2.421702	1.805138	-0.835711
H	0.893588	2.738885	1.761764
H	-1.796156	2.338877	1.778764
S	2.068222	-0.596266	-0.002620
O	2.751847	-0.385644	-1.277555
O	2.684641	-0.021250	1.191837
O	-0.412292	2.159148	-1.190289
C	1.850481	-2.344666	0.249543
H	1.329881	-2.749119	-0.616279
H	2.850772	-2.768423	0.339208
H	1.280840	-2.486848	1.165827
Br	-2.183488	-1.279047	0.020804

Structure **Bromomesylethyne**

C	-0.337293	-0.074557	0.000001
C	0.865622	-0.033956	-0.000047
S	-2.062474	-0.158824	0.000109
O	-2.475361	-0.776198	-1.250886
O	-2.475200	-0.776065	1.251222
C	-2.538484	1.549974	0.000050
H	-2.145198	2.013354	-0.902314
H	-3.628730	1.553934	0.000106
H	-2.145102	2.013439	0.902327
Br	2.645258	0.020851	-0.000131

Structure **1b'**

C	0.471311	2.367222	-0.895896
C	-0.175129	1.844581	0.403417
C	1.783348	1.037869	0.404110
C	1.695649	1.854526	-0.900437
H	-0.031748	2.968238	-1.638272
H	2.474384	1.913116	-1.645570

Structure **MeOH**

O	-0.747697	0.123119	0.000000
H	-1.141539	-0.753037	0.000000
C	0.666639	-0.019951	0.000000
H	1.089040	0.983885	-0.000001
H	1.017120	-0.548048	0.890856
H	1.017120	-0.548050	-0.890855

Structure **1b_*MeOH***

C	0.995368	0.335206	2.210052
C	-0.239086	1.183205	1.950892
C	-0.897848	-0.837330	1.782783
C	0.582411	-0.924823	2.099397
C	-0.570873	1.099324	0.412466
H	-1.391583	1.785037	0.195486
C	-1.048720	-0.385003	0.272177
H	-0.279040	2.187207	2.358714
H	-1.534290	-1.672785	2.061366
O	-1.282654	0.359901	2.454130
H	1.166082	-1.832890	2.125034
H	1.996823	0.714930	2.352359
S	0.796225	1.787335	-0.558636
O	1.944888	0.876305	-0.591556
O	1.014451	3.121900	0.003077
C	0.183047	1.956888	-2.219323
H	-0.656354	2.649677	-2.214556
H	1.015647	2.367058	-2.791256
H	-0.091948	0.970460	-2.590362
S	-2.851775	-0.448251	-0.071772
C	-2.916545	0.152511	-1.775962
H	-3.959872	0.080189	-2.080782
H	-2.606589	1.195364	-1.840932
H	-2.304824	-0.471111	-2.427766
S	-0.056444	-1.389157	-0.896777
C	-0.961772	-2.958471	-0.852525
H	-1.947528	-2.857890	-1.301305
H	-0.358134	-3.652281	-1.436979
H	-1.045236	-3.332256	0.167207
C	3.647637	-1.872377	-0.939501
H	4.434002	-2.619648	-0.831294
H	2.822179	-2.312657	-1.509008
H	4.046943	-1.022313	-1.501338
O	3.234215	-1.490645	0.360282
H	2.556711	-0.809385	0.250181

Structure **TS-1b_*MeOH***

C	-1.055676	-0.001344	2.199703
C	0.147856	-0.745205	2.341273
C	0.745190	1.272901	1.921127
C	-0.667673	1.285396	1.926754
C	0.705628	-0.963689	0.362095
H	1.539402	-1.624461	0.582947
C	1.020783	0.354080	-0.042123
H	0.280820	-1.727182	2.770300
H	1.433172	2.105359	1.956431
O	1.171346	0.129706	2.525494
H	-1.300774	2.108837	1.634856
H	-2.050750	-0.417191	2.182123
S	-0.590775	-1.974882	-0.313691
O	-1.849358	-1.229867	-0.444256
O	-0.621012	-3.199227	0.490541
C	-0.040527	-2.389858	-1.957025
H	0.899335	-2.933248	-1.879808
H	-0.819344	-3.019454	-2.387308
H	0.071170	-1.465896	-2.523742
S	2.785540	0.716416	-0.153508
C	3.186544	-0.168686	-1.687743
H	4.225567	0.060313	-1.922813
H	3.074376	-1.243706	-1.548542
H	2.545471	0.182967	-2.495852
S	-0.041877	1.171577	-1.235737
C	0.409388	2.905012	-0.993347
H	1.478226	3.046357	-1.141700
H	-0.143824	3.465653	-1.746026
H	0.112892	3.245286	-0.001517
C	-3.694842	1.567099	-1.208099
H	-4.513203	2.277828	-1.091712
H	-2.902988	2.040078	-1.798951
H	-4.066243	0.694579	-1.755047
O	-3.238920	1.217984	0.086159
H	-2.545971	0.551101	-0.025876

8. ^{1}H and ^{13}C-NMR spectra for new compounds.

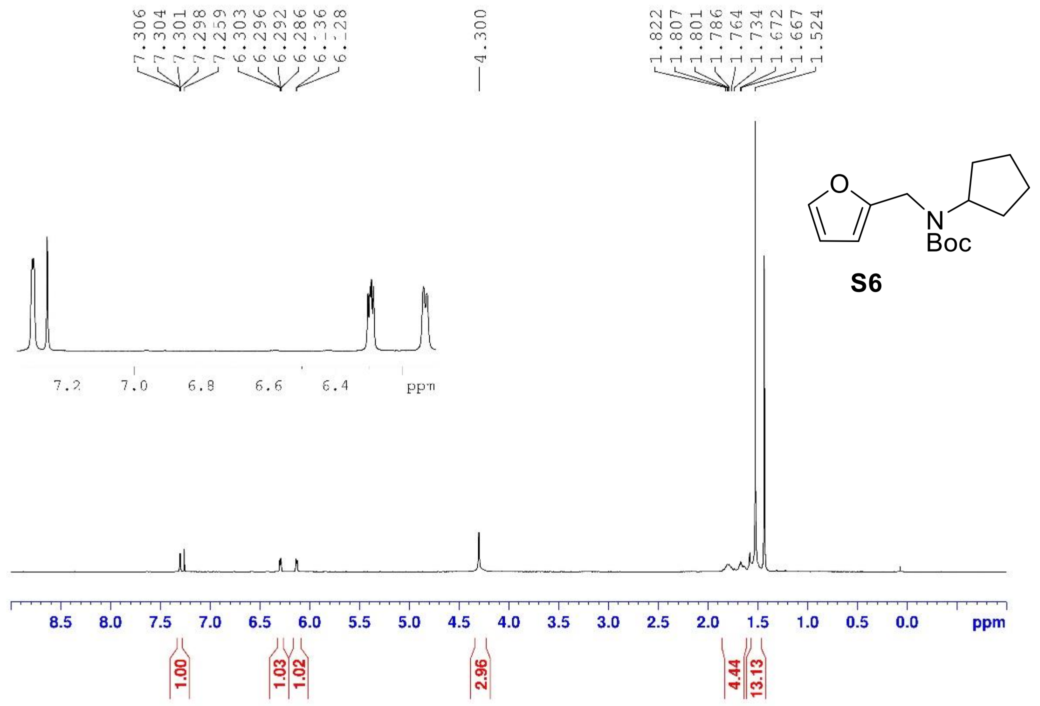

^{1}H NMR ($CDCl_3$, 300 MHz) of compound **S6**

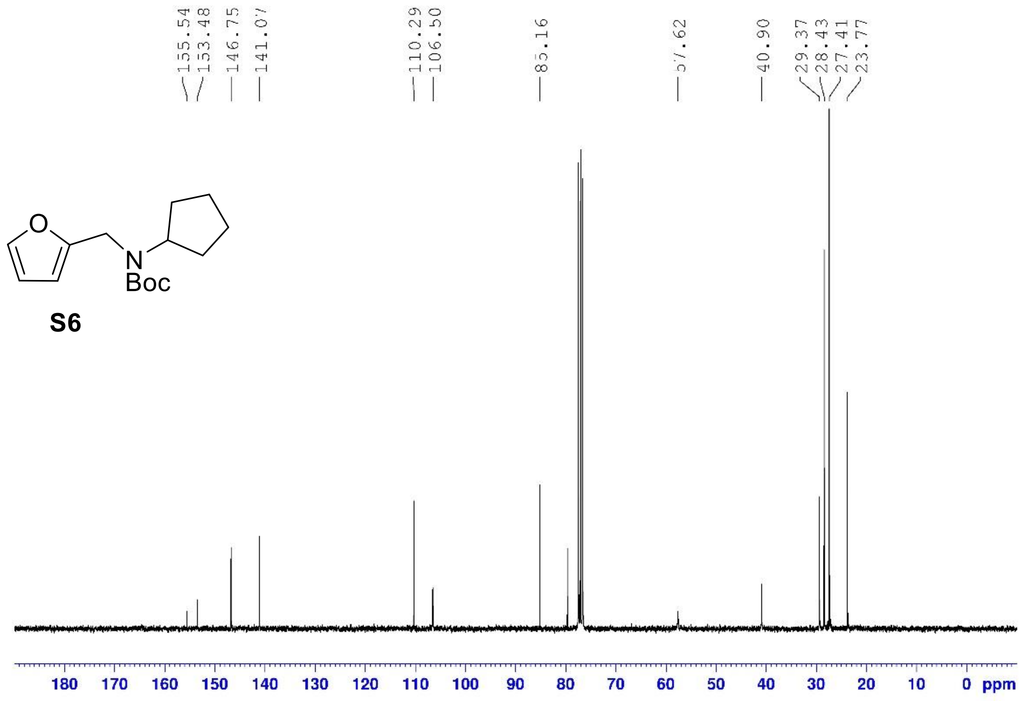

^{13}C NMR ($CDCl_3$, 75 MHz) of compound **S6**

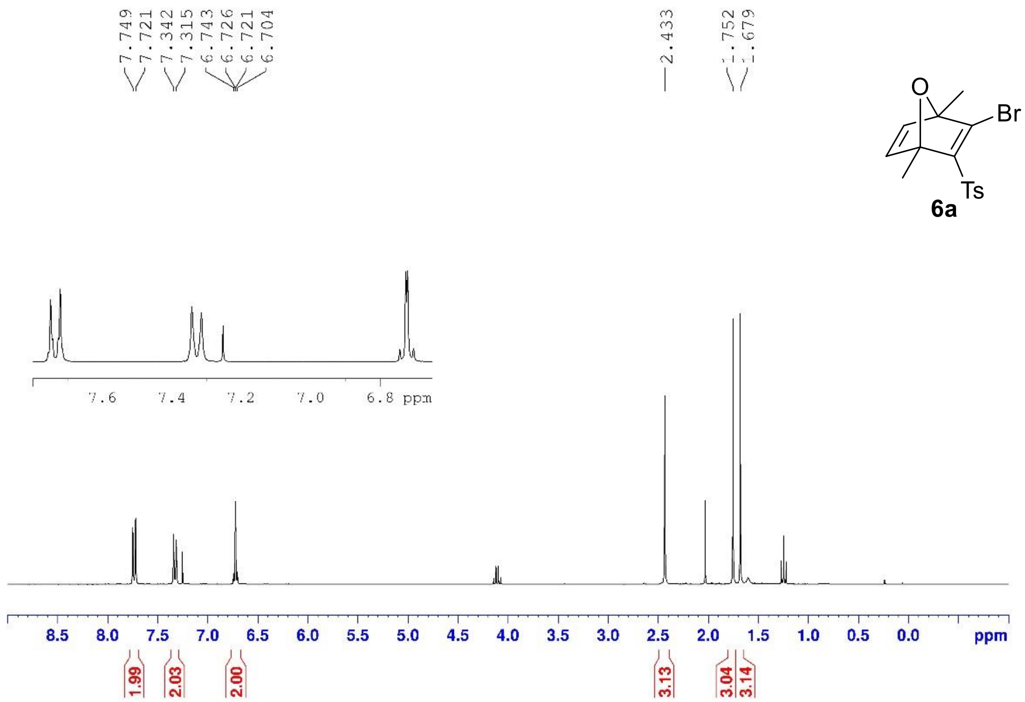

^{1}H NMR ($CDCl_3$, 300 MHz) of compound **6a**

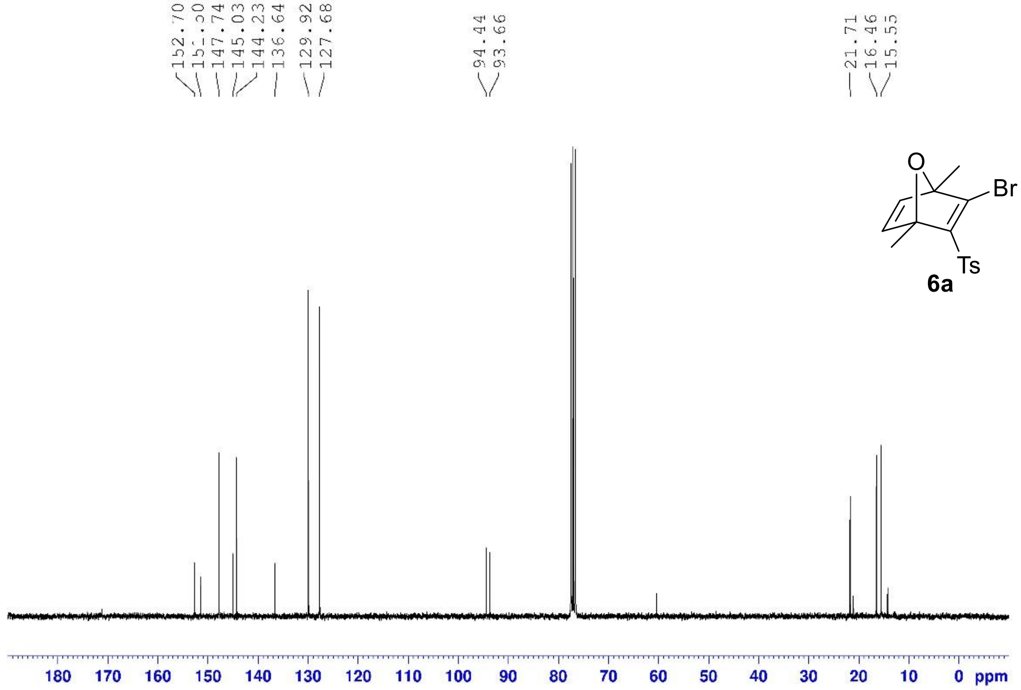

^{13}C NMR ($CDCl_3$, 75 MHz) of compound **6a**

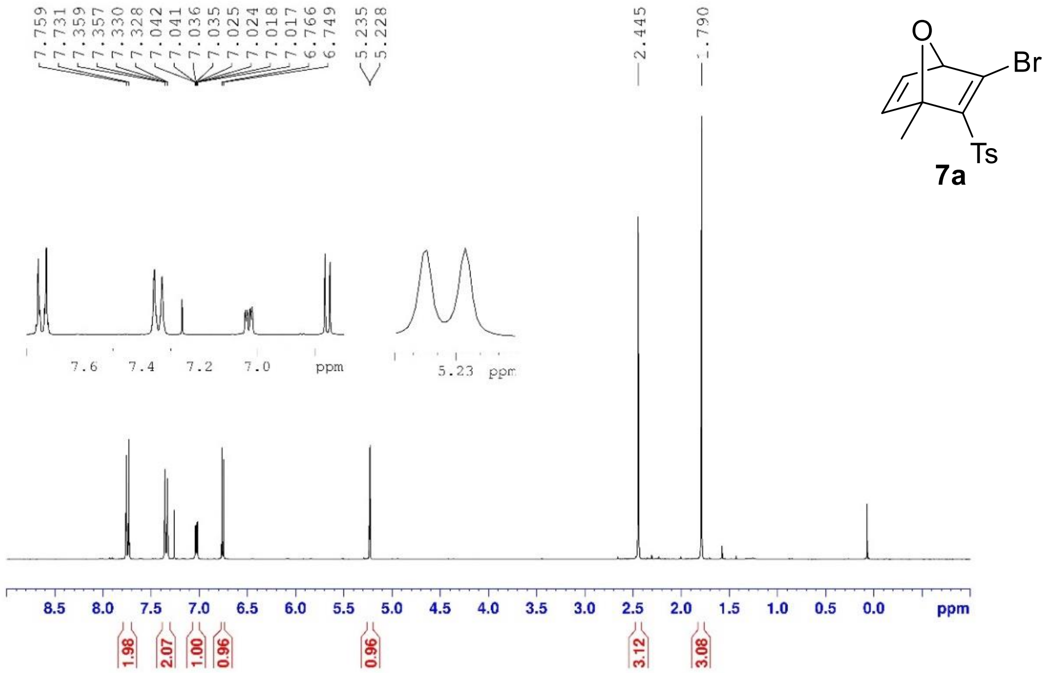

^1H NMR ($CDCl_3$, 300 MHz) of compound **7a**

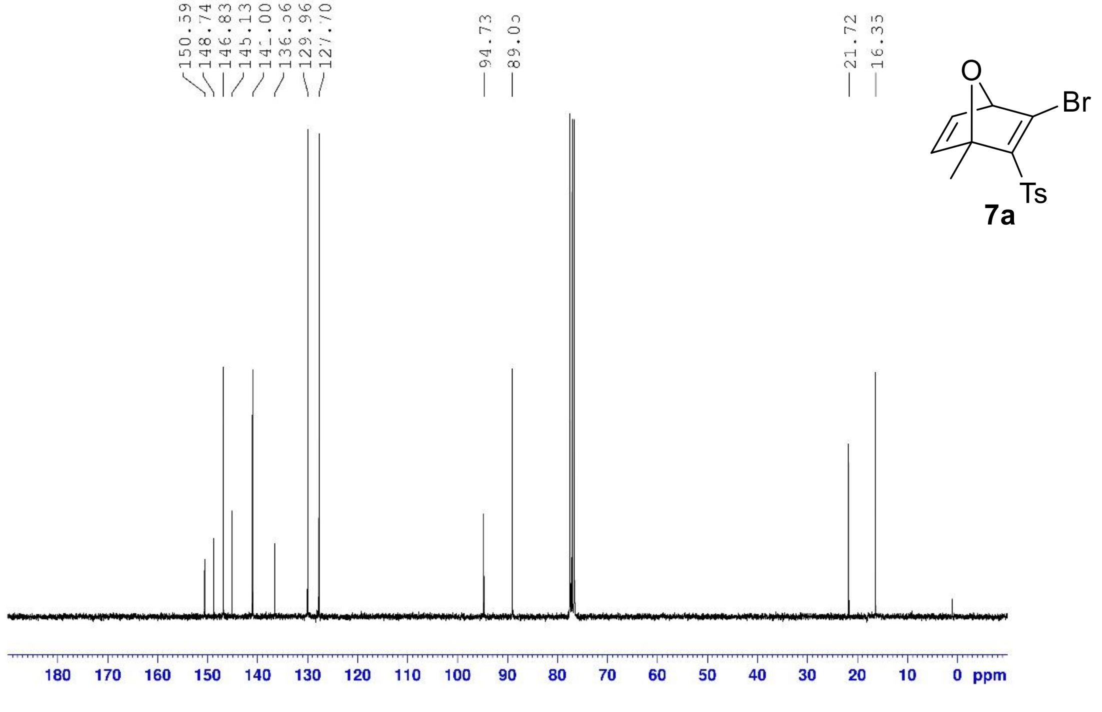

^{13}C NMR ($CDCl_3$, 75 MHz) of compound **7a**

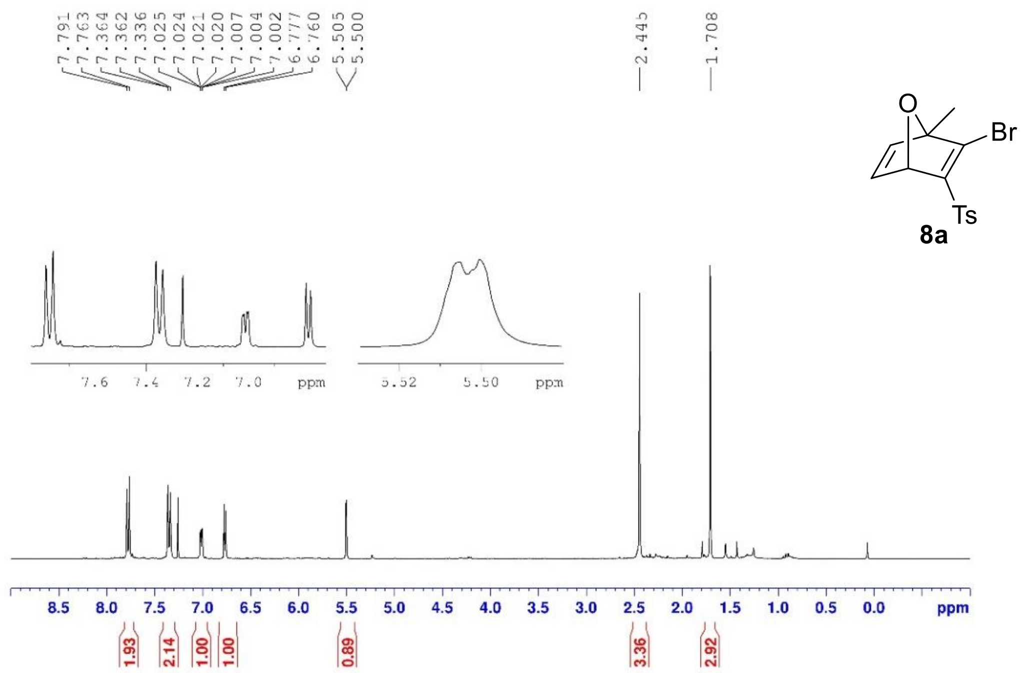

^{1}H NMR ($CDCl_3$, 300 MHz) of compound **8a**

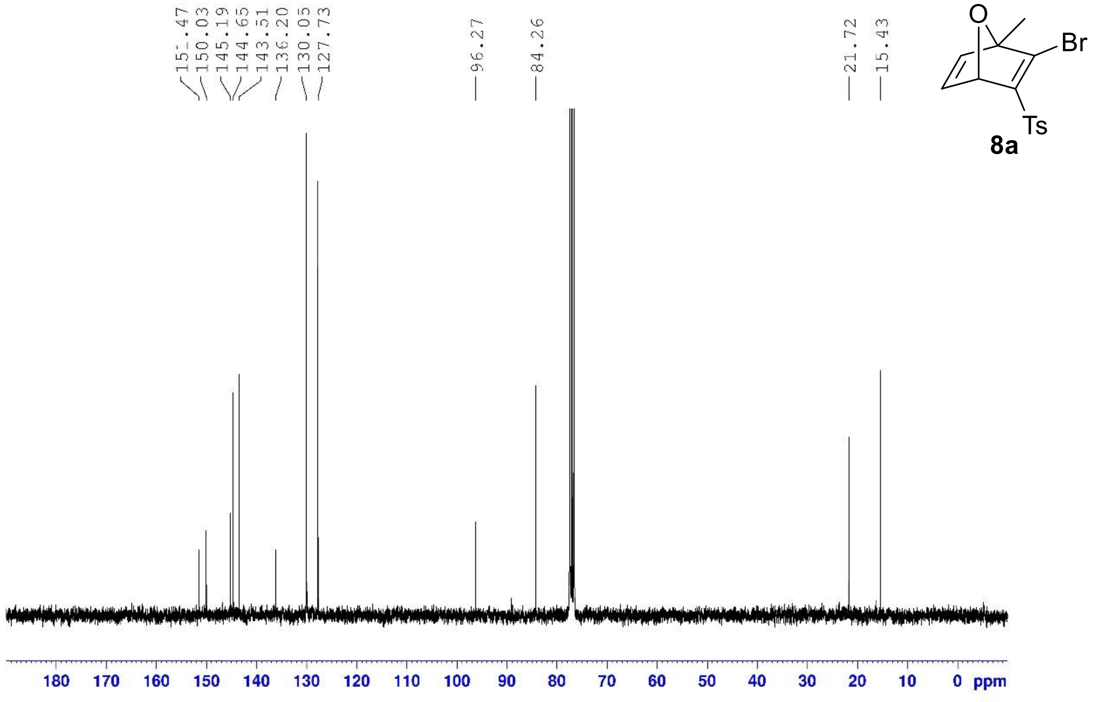

^{13}C NMR ($CDCl_3$, 75 MHz) of compound **8a**

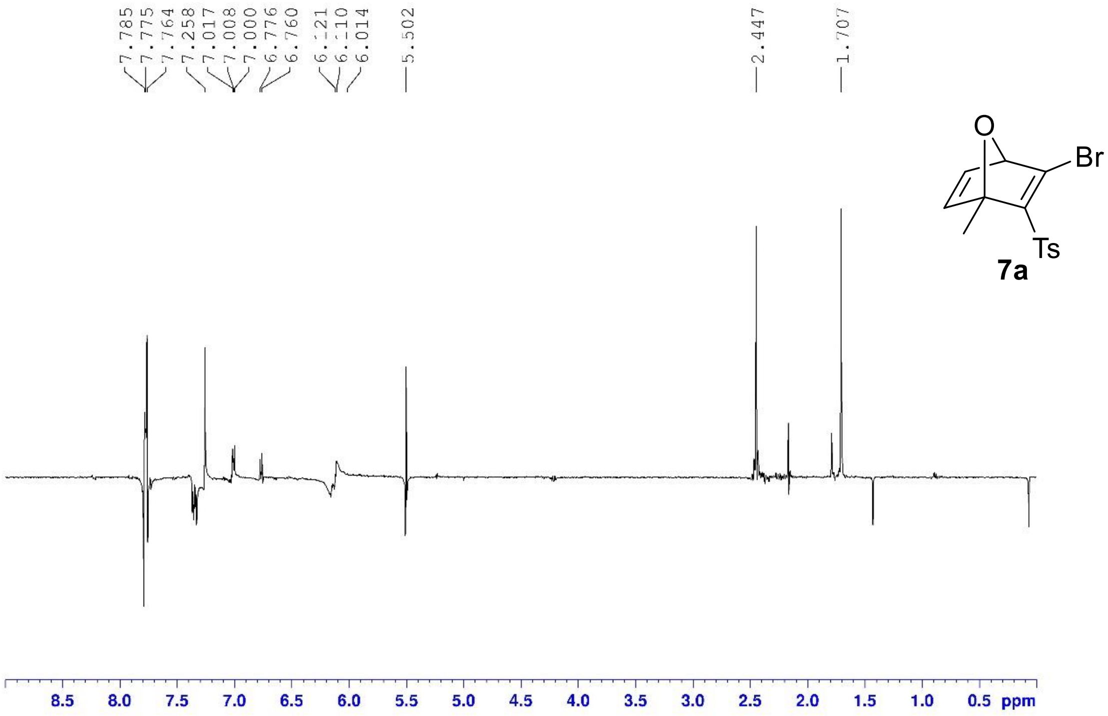

1D-NOE ($CDCl_3$, 300 MHz) of compound **7a**

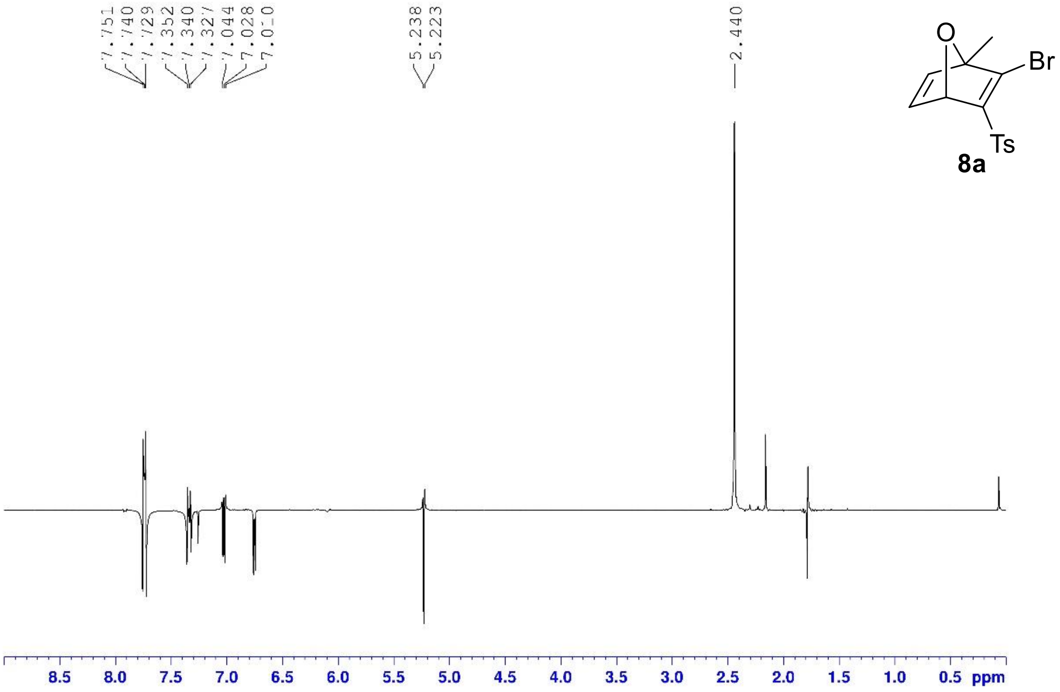

1D-NOE ($CDCl_3$, 300 MHz) of compound **8a**

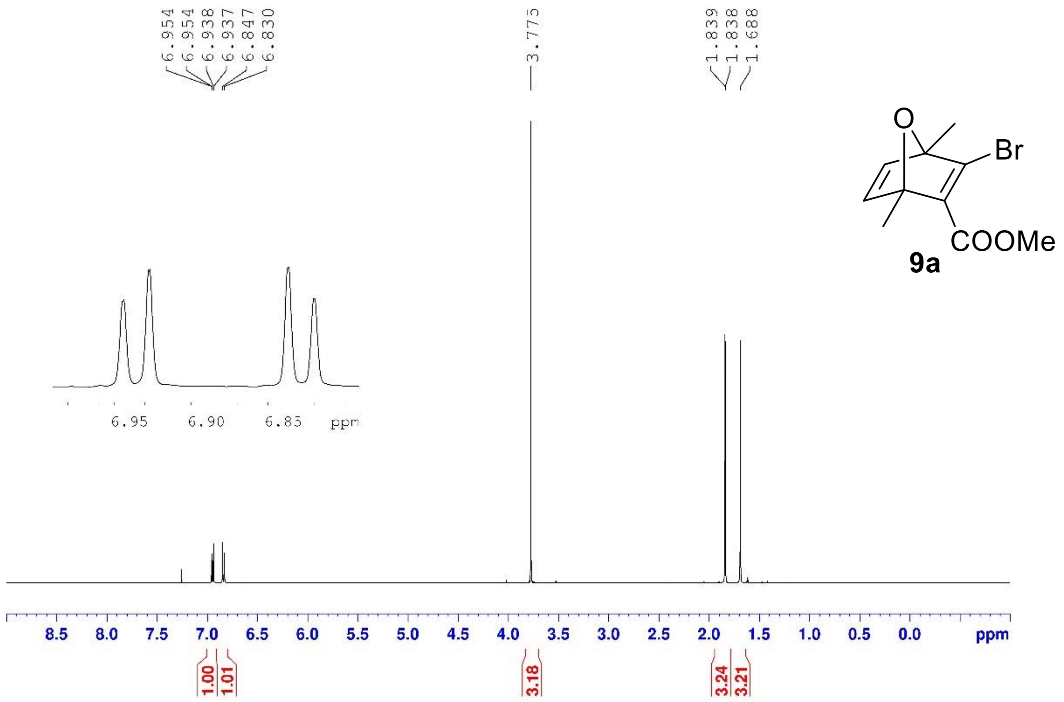

^{1}H NMR ($CDCl_3$, 300 MHz) of compound **9a**

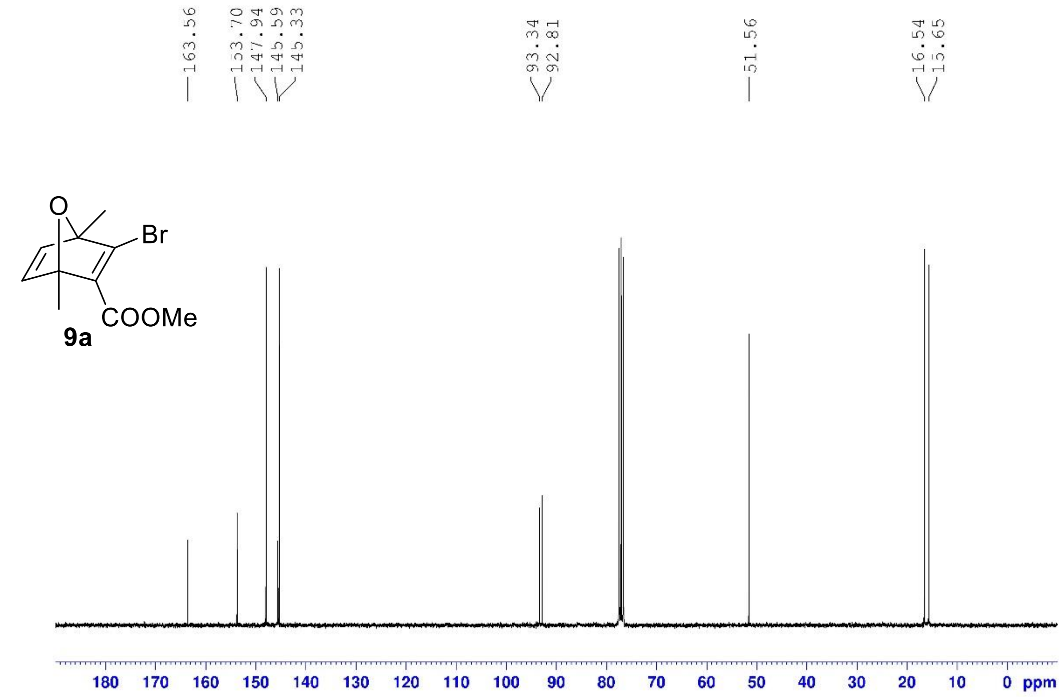

^{13}C NMR ($CDCl_3$, 75 MHz) of compound **9a**

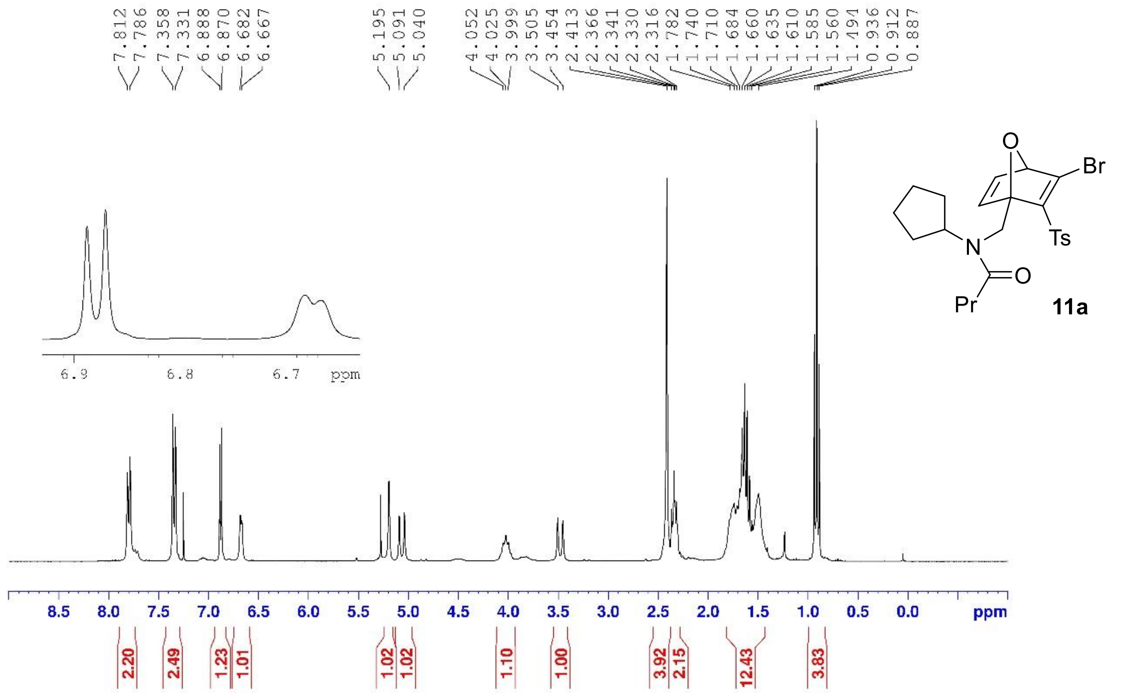

^{1}H NMR (CDCl$_3$, 300 MHz) of compound **11a**

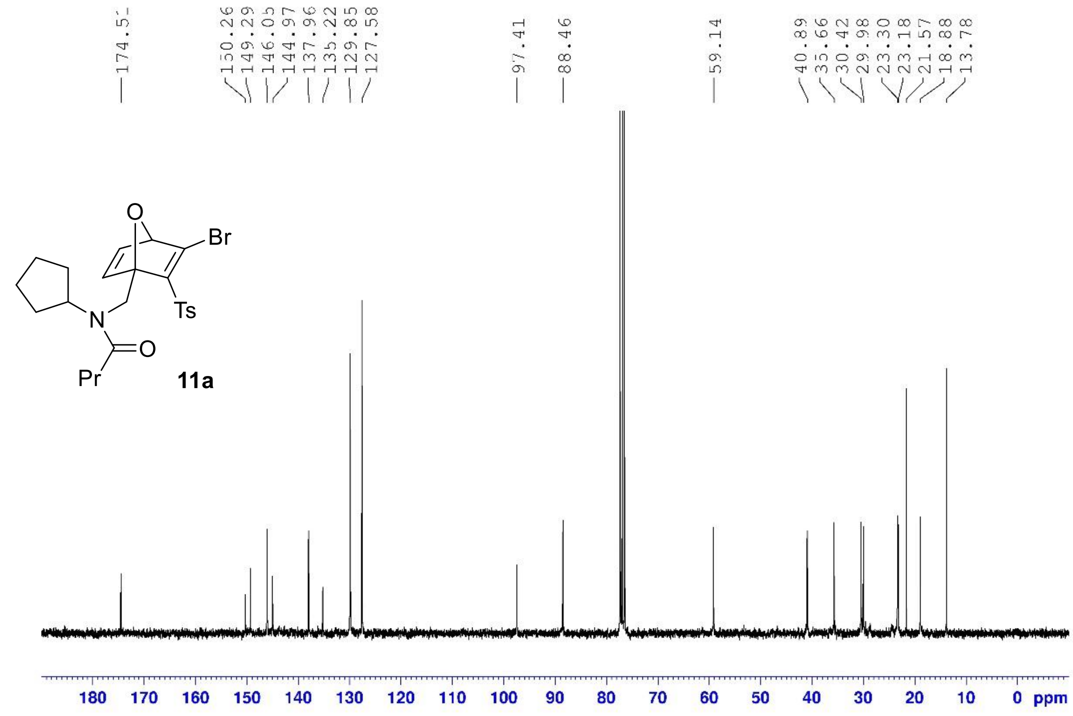

^{13}C NMR (CDCl$_3$, 75 MHz) of compound **11a**

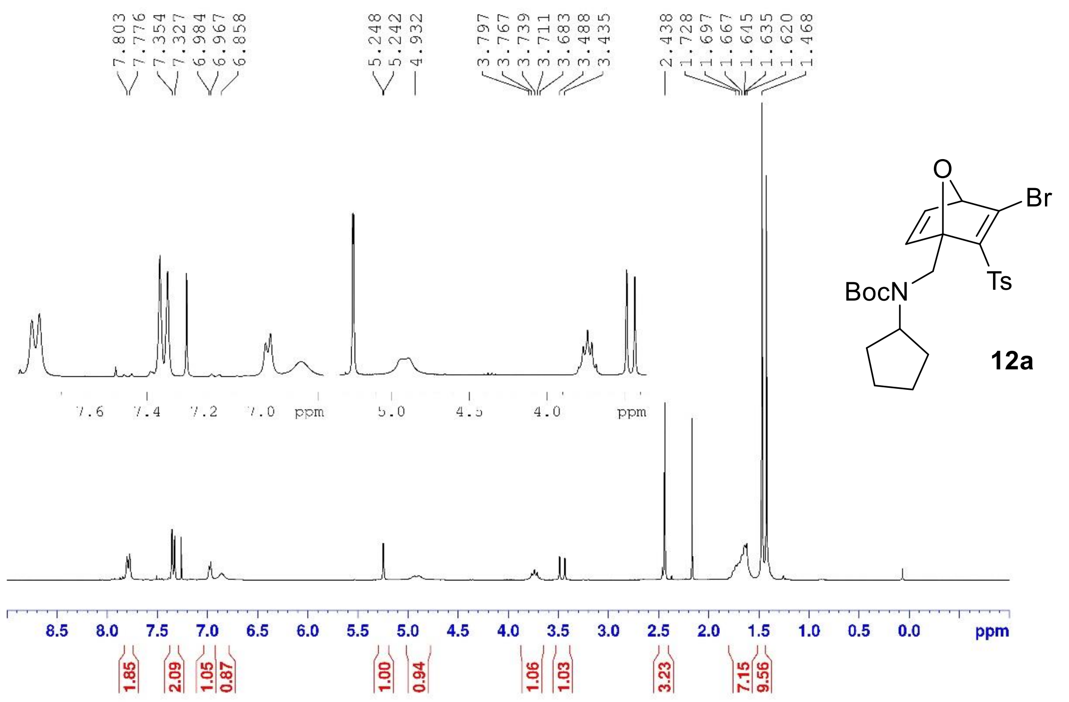

^{1}H NMR ($CDCl_3$, 300 MHz) of compound **12a**

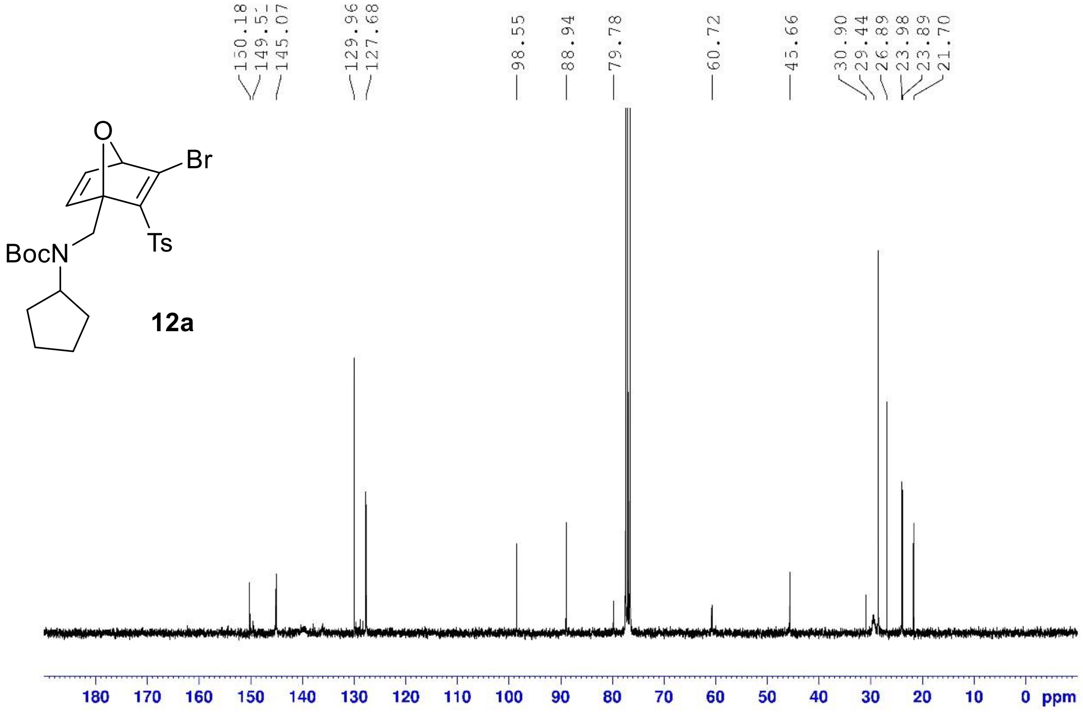

^{13}C NMR ($CDCl_3$, 75 MHz) of compound **12a**

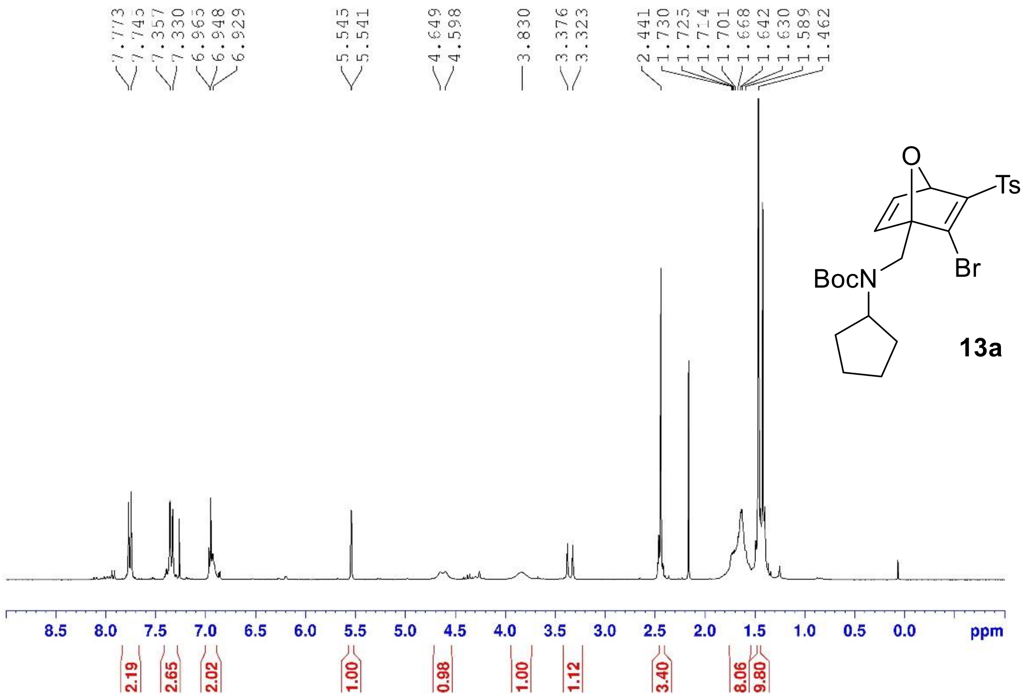

^1H NMR ($CDCl_3$, 300 MHz) of compound **13a**

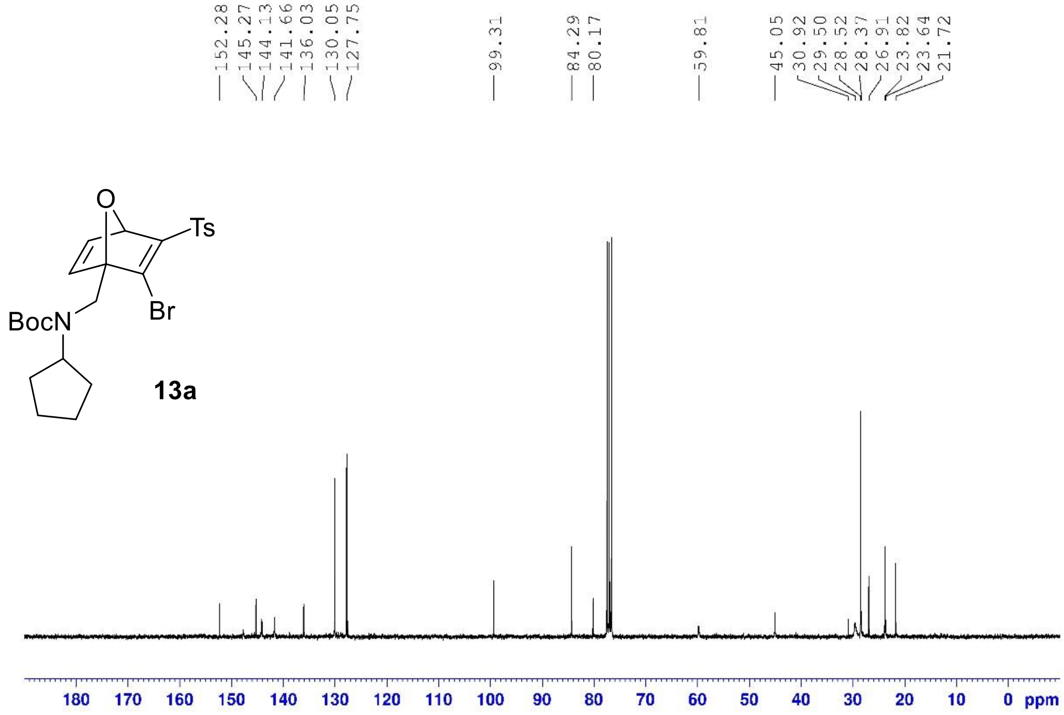

^{13}C NMR ($CDCl_3$, 75 MHz) of compound **13a**

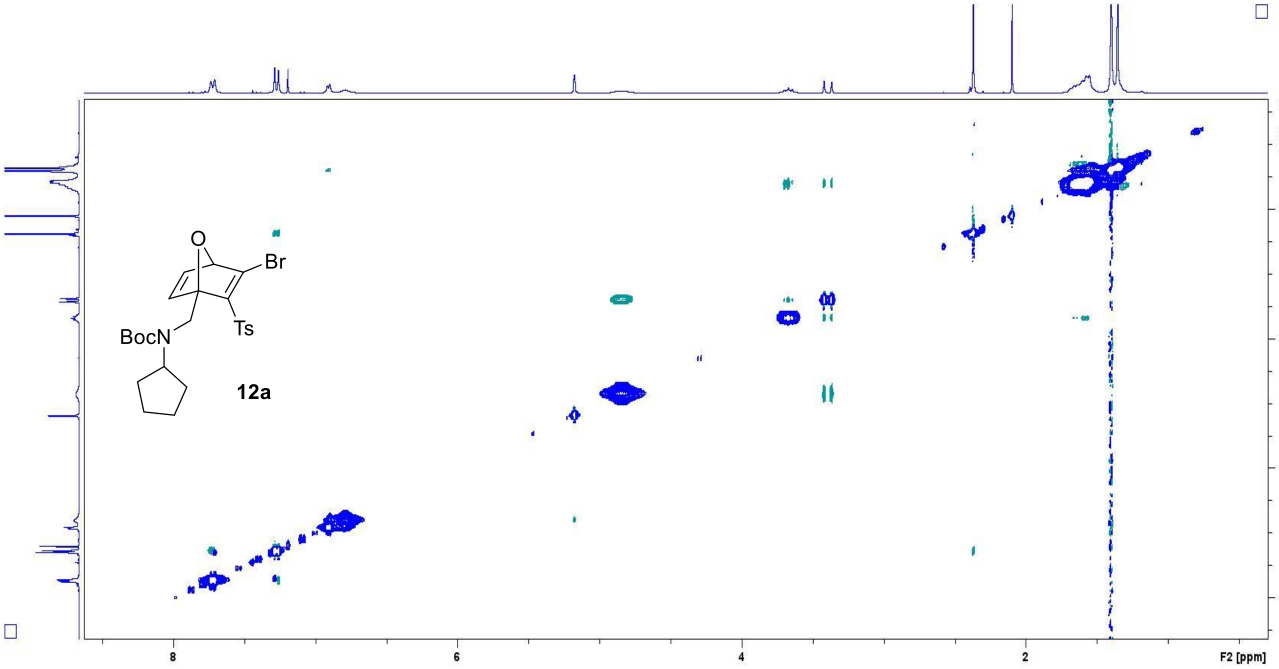

NOESY ($CDCl_3$, 300 MHz) of compound **12a**

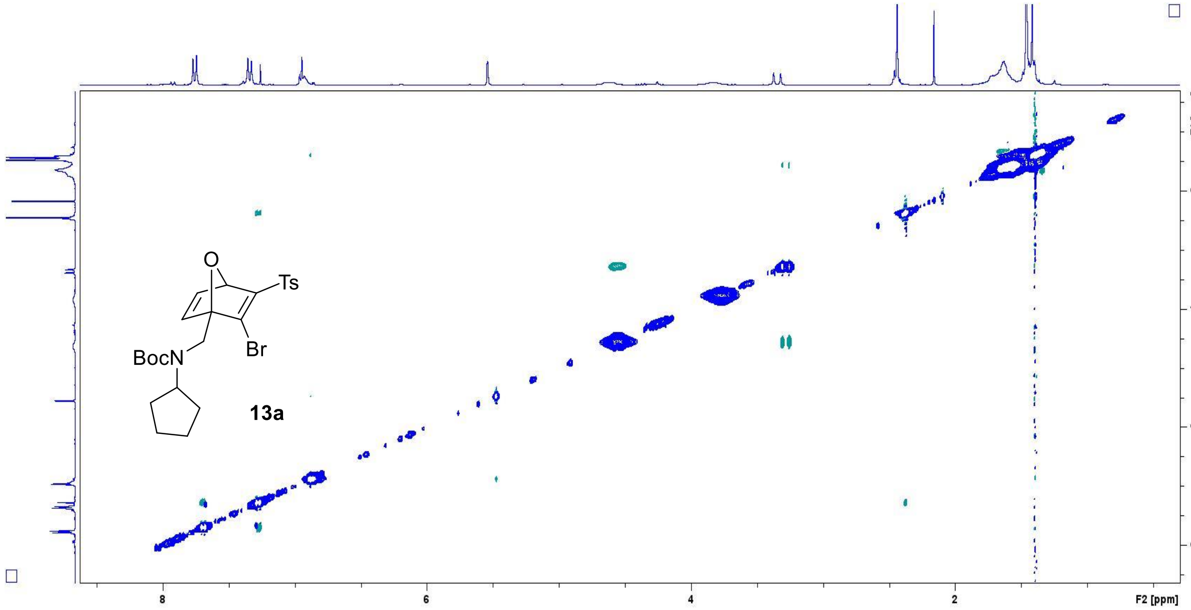

NOESY ($CDCl_3$, 300 MHz) of compound **13a**

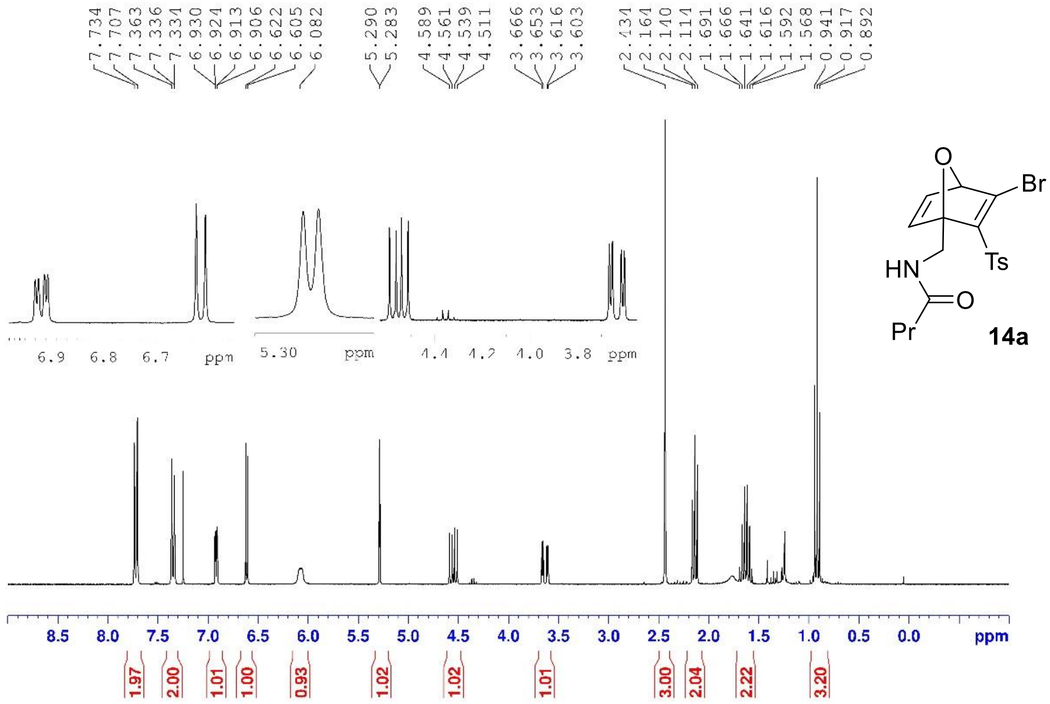

^{1}H NMR ($CDCl_3$, 300 MHz) of compound **14a**

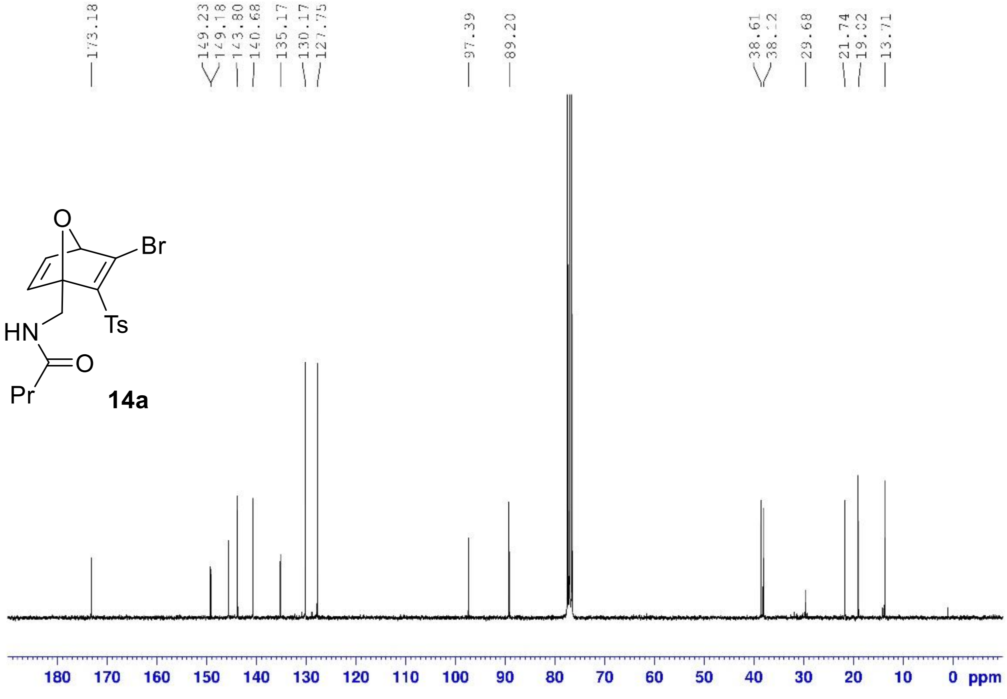

^{13}C NMR ($CDCl_3$, 75 MHz) of compound **14a**

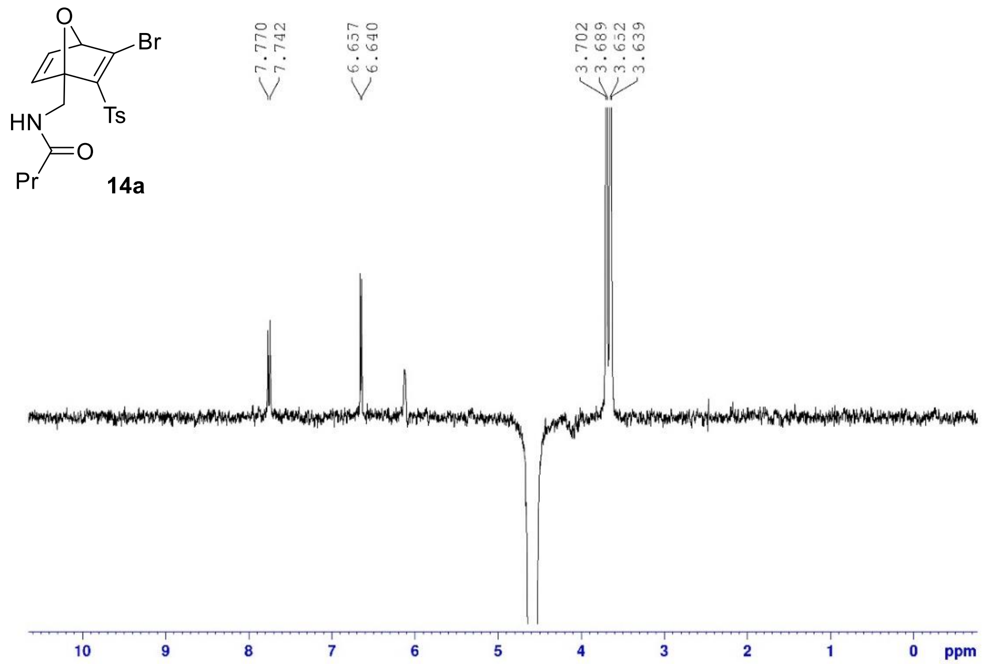

1D-NOE ($CDCl_3$, 300 MHz) of compound **14a**

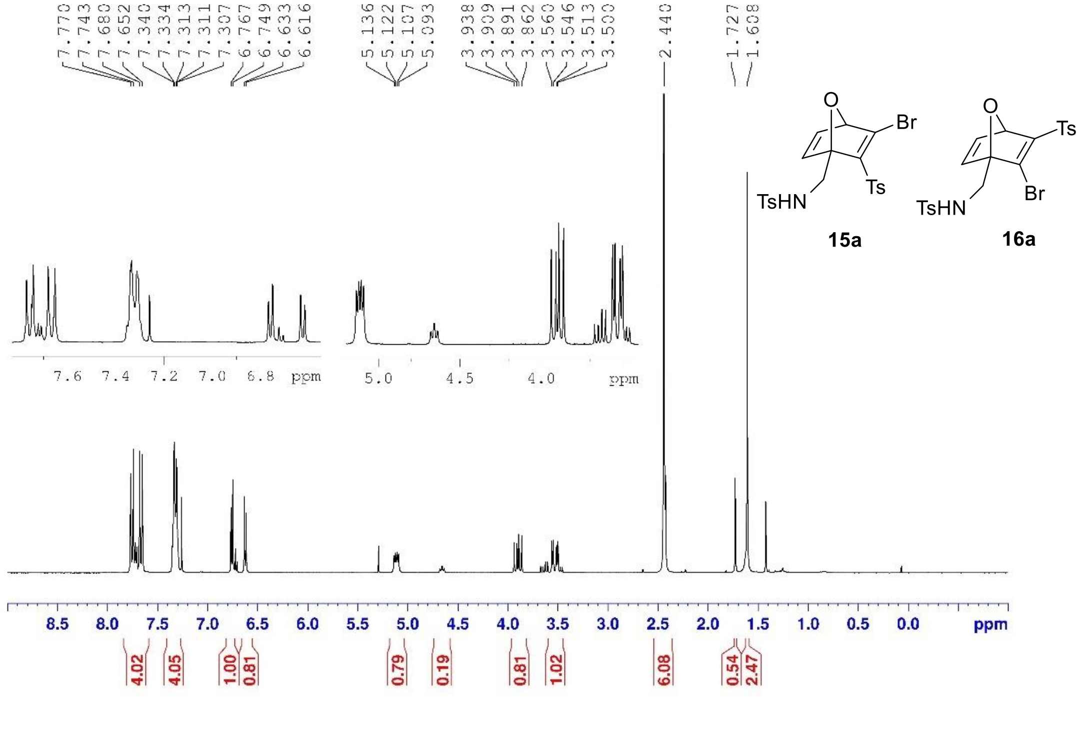

^1H NMR ($CDCl_3$, 300 MHz) of compounds **15a** and **16a**

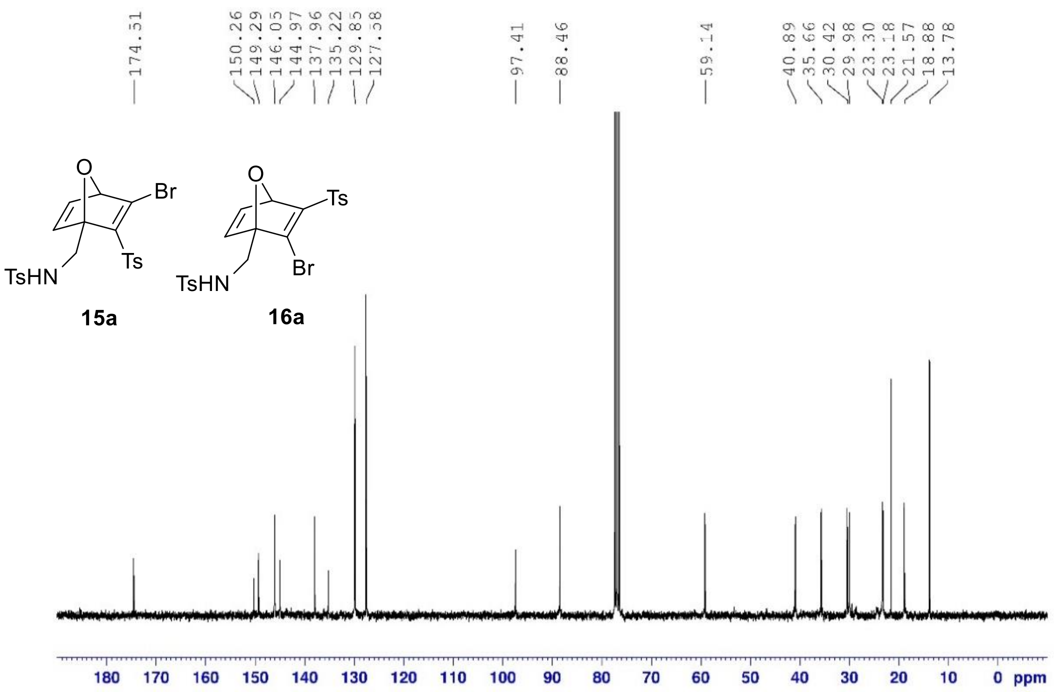

^{13}C NMR ($CDCl_3$, 75 MHz) of compounds **15a** and **16a**

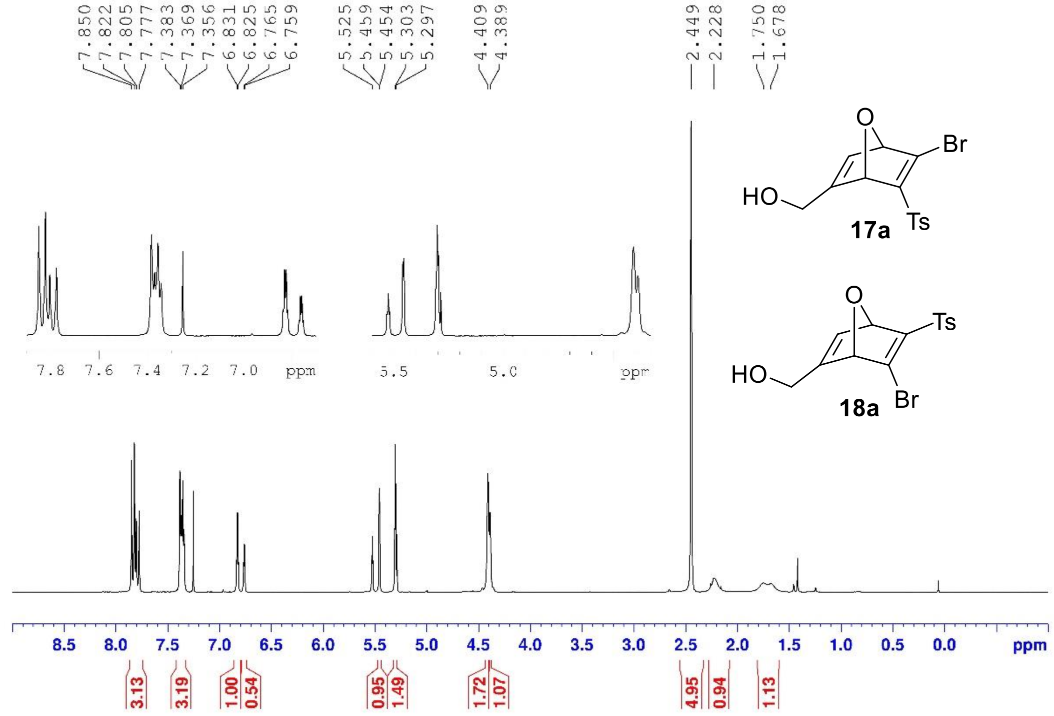

^{1}H NMR ($CDCl_3$, 300 MHz) of compounds **17a** and **18a**

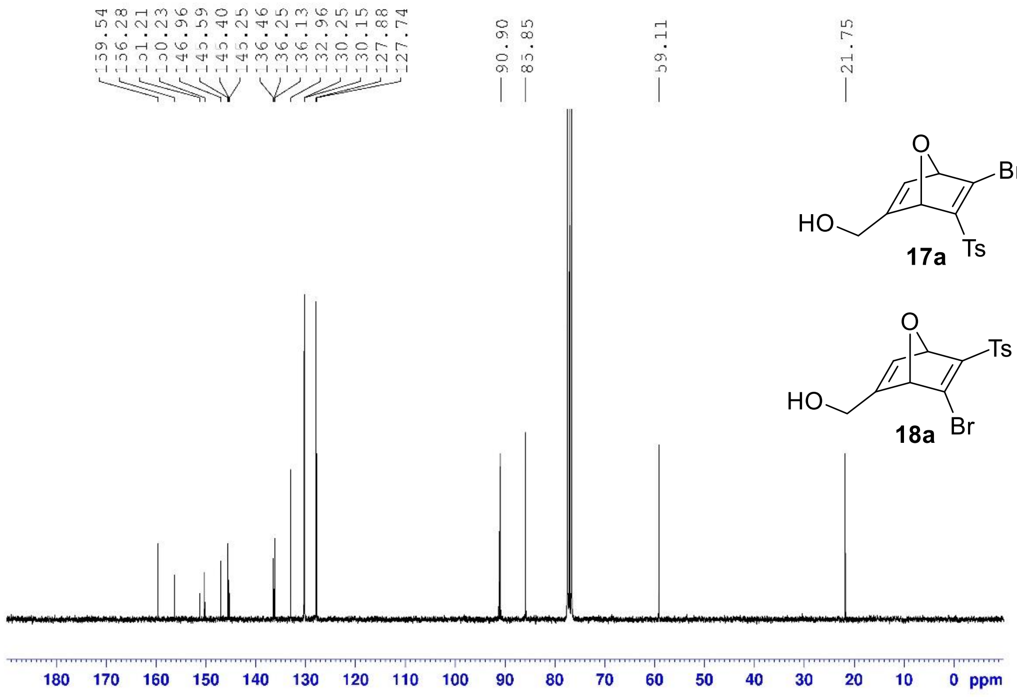

^{13}C NMR ($CDCl_3$, 75 MHz) of compounds **17a** and **18a**

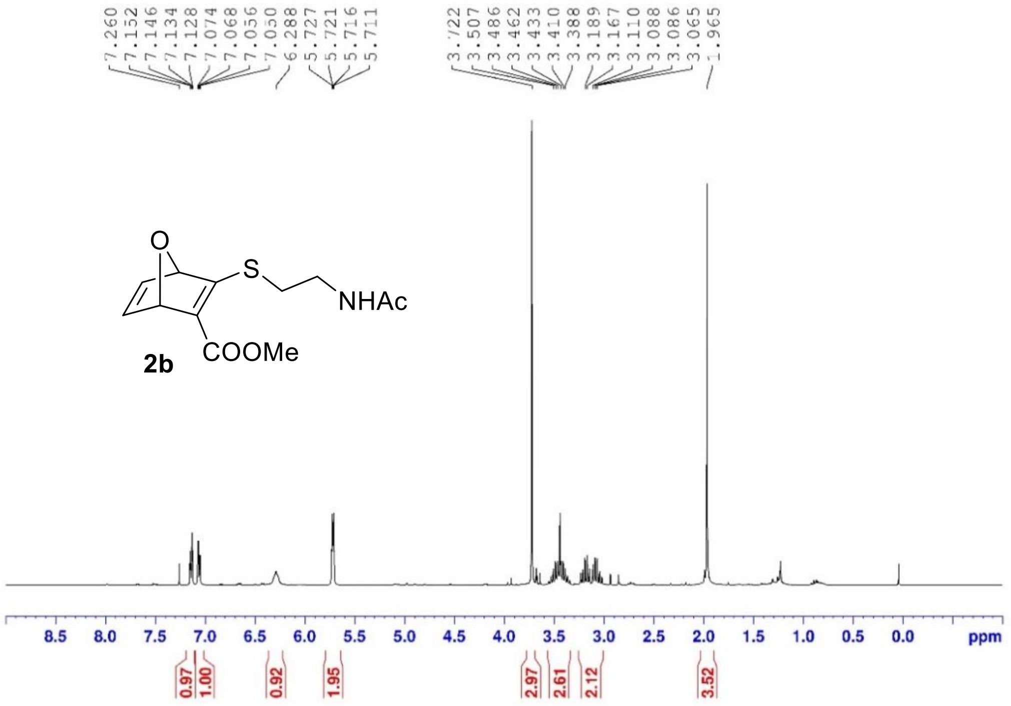

^{1}H NMR ($CDCl_3$, 300 MHz) of compound **2b**

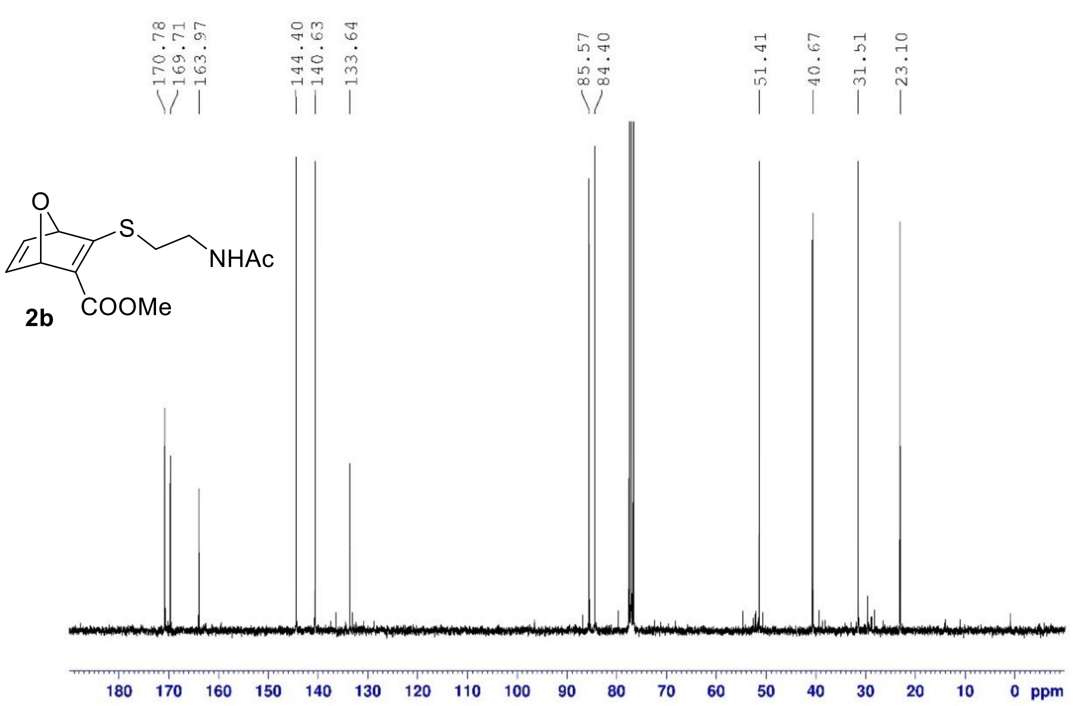

^{13}C NMR ($CDCl_3$, 75 MHz) of compound **2b**

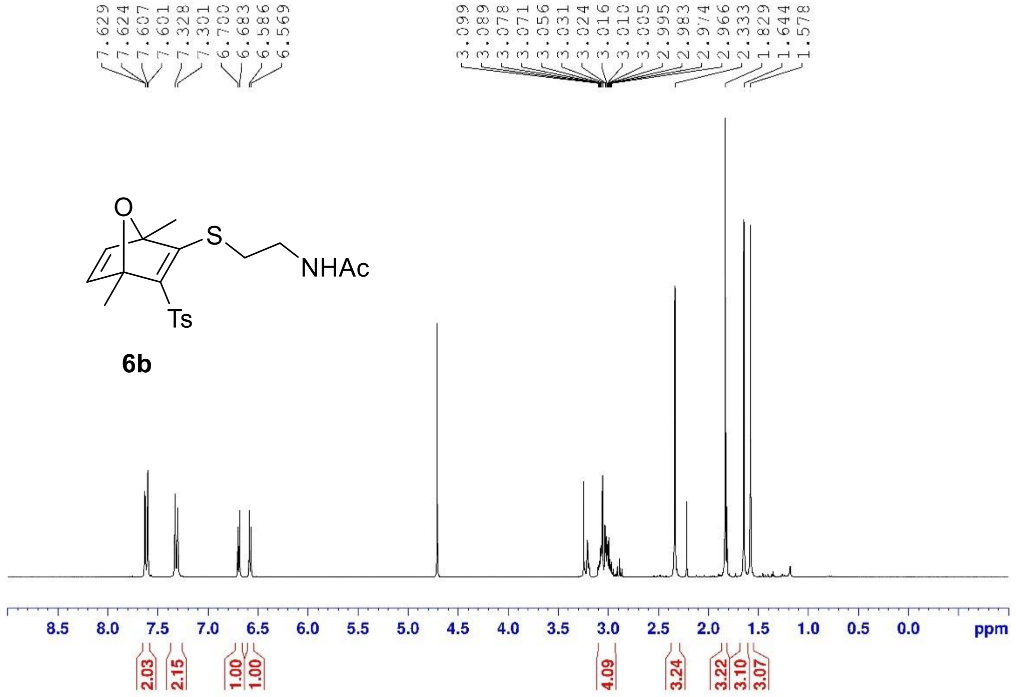

^{1}H NMR (CD_3OD, 300 MHz) of compound **6b**

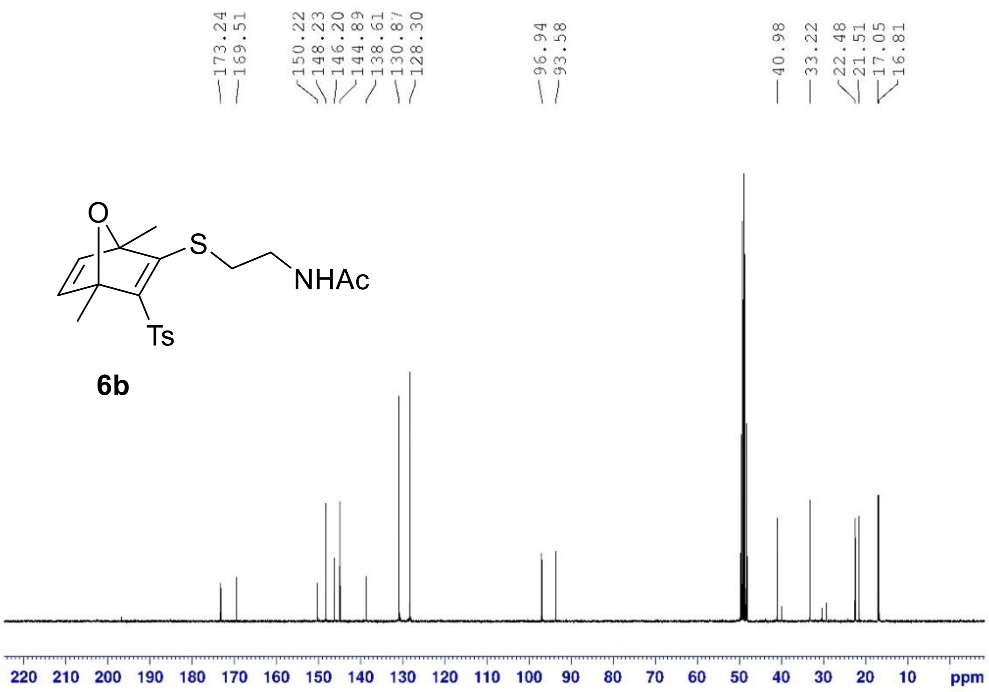

^{13}C NMR (CD_3OD, 75 MHz) of compound **6b**

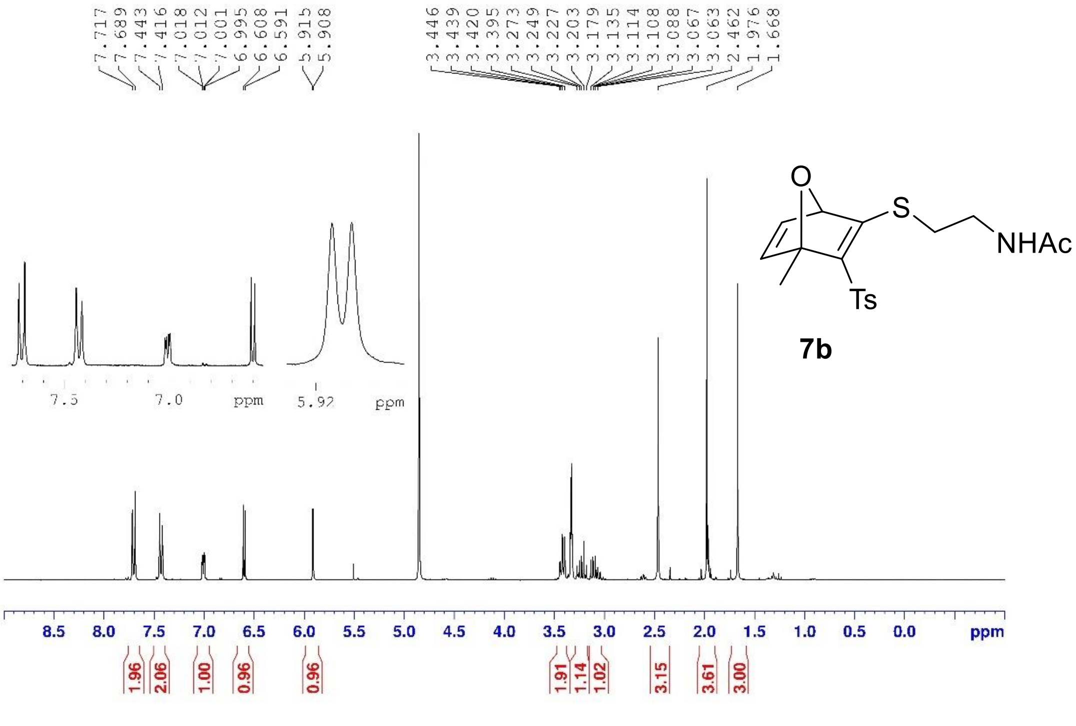

^{1}H NMR (CD_3OD, 300 MHz) of compound **7b**

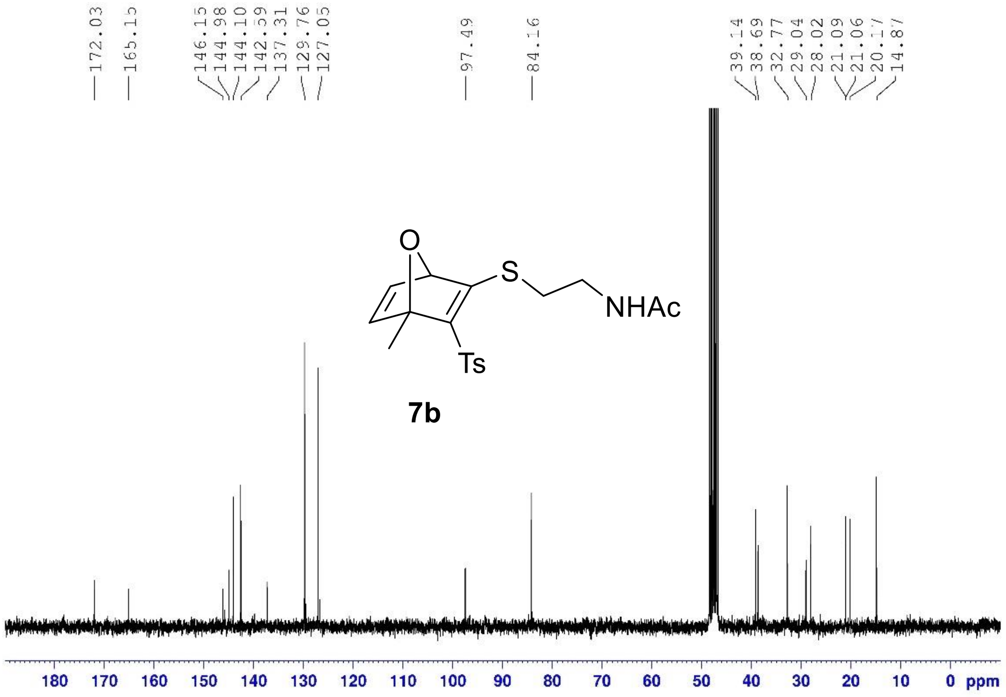

^{13}C NMR (CD$_3$OD, 75 MHz) of compound **7b**

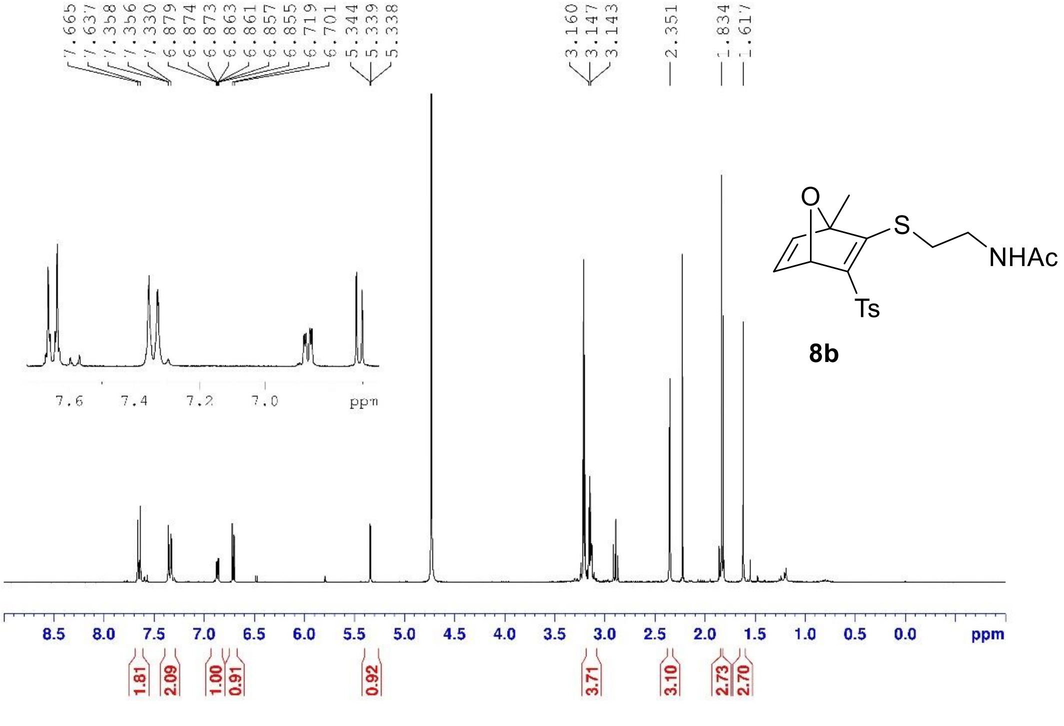

^{1}H NMR (CD$_3$OD, 300 MHz) of compound **8b**

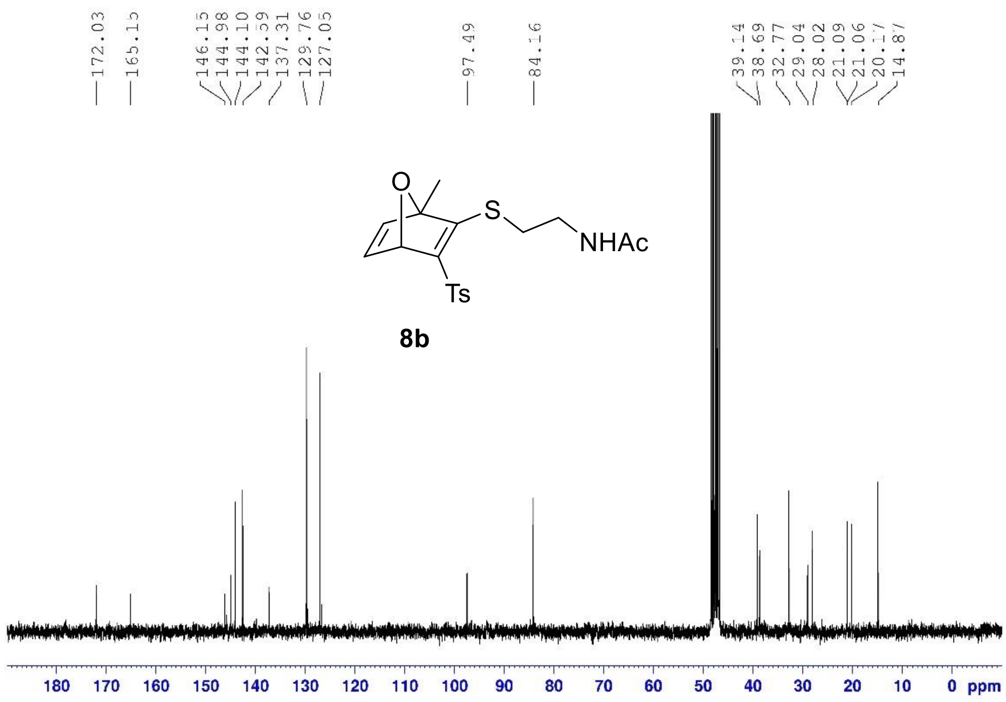

^{13}C NMR (CD_3OD, 75 MHz) of compound **8b**

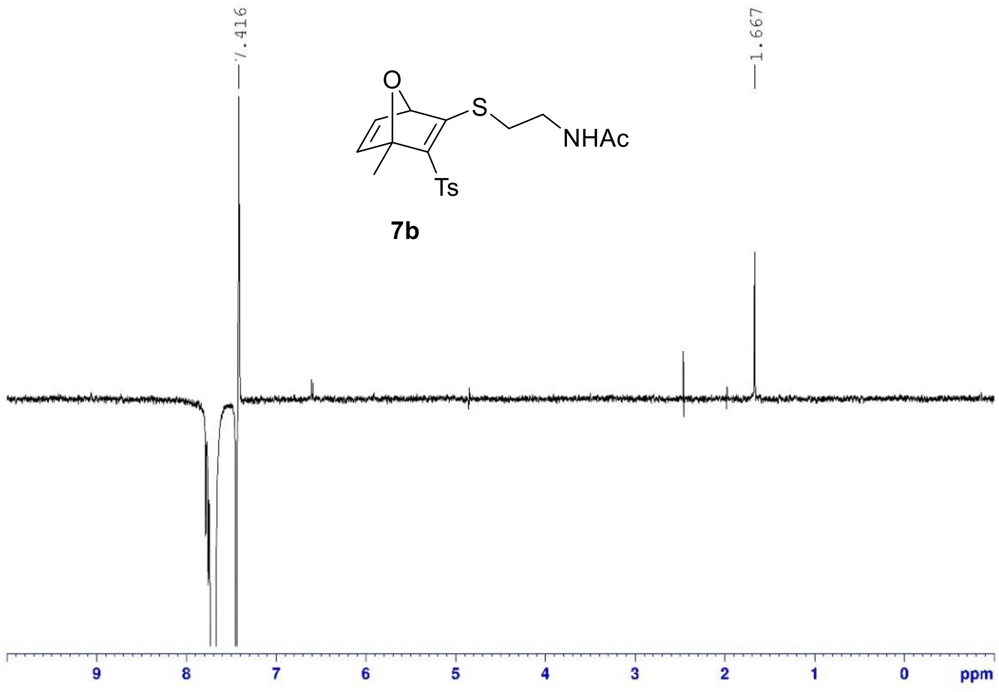

1D NOE (CD_3OD, 300 MHz) of compound **7b**

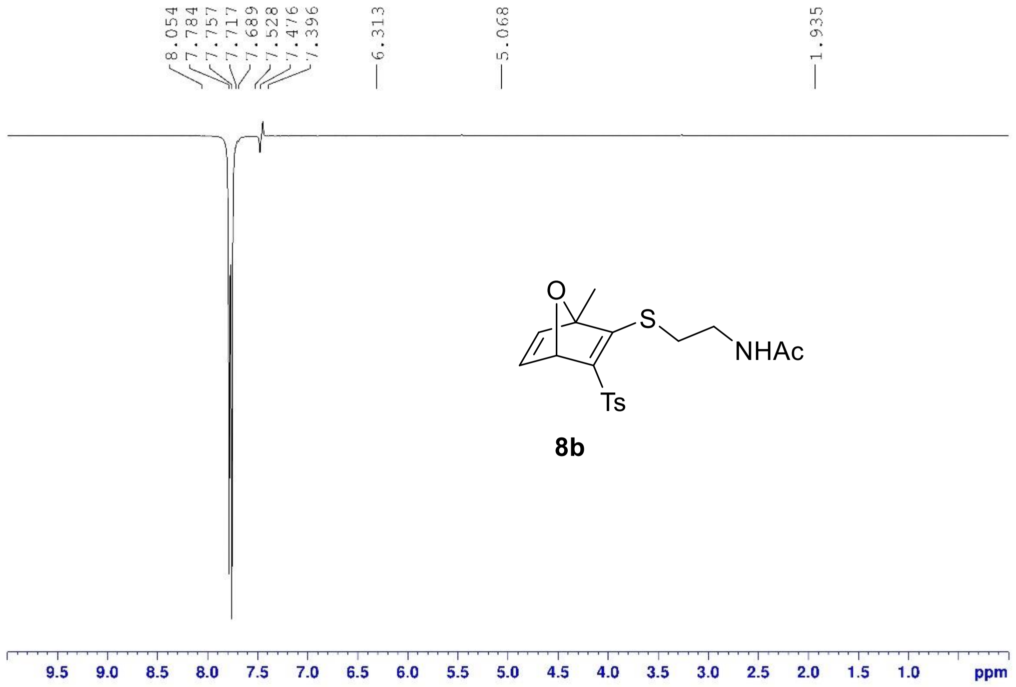

1D NOE (CD_3OD, 300 MHz) of compound **8b**

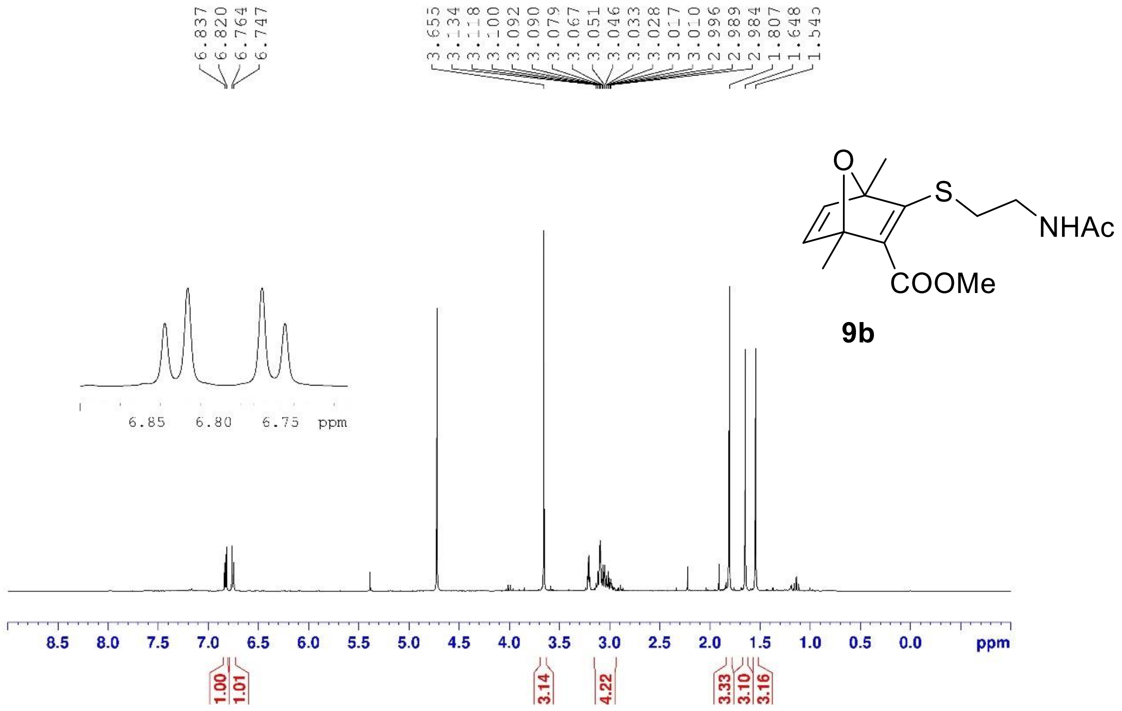

^{1}H NMR (CD_3OD, 300 MHz) of compound **9b**

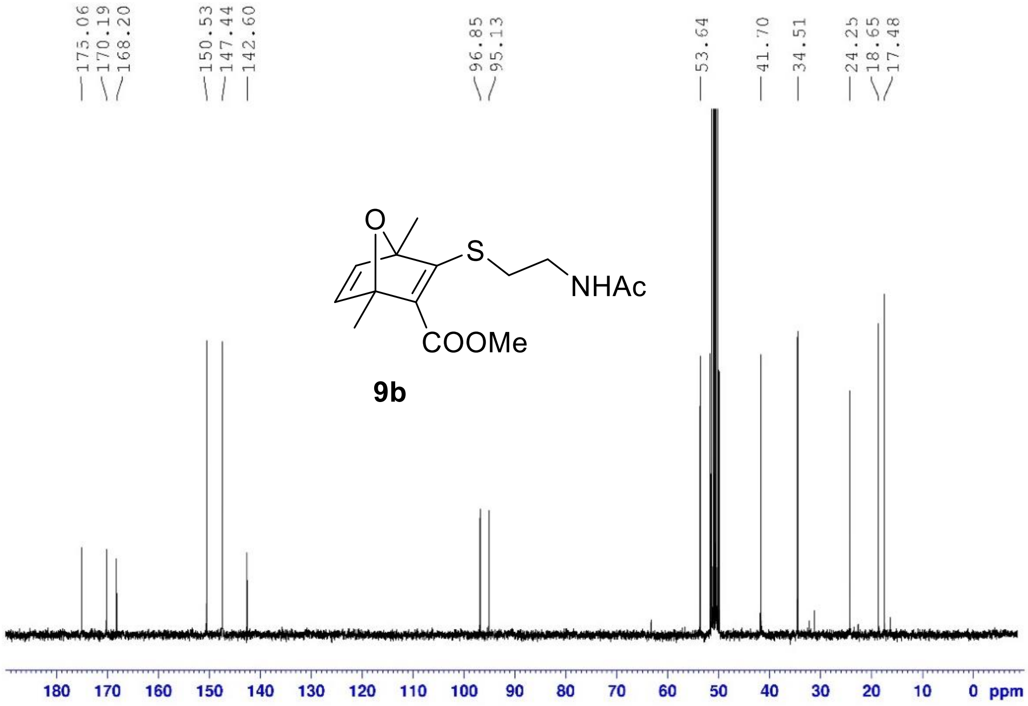

^{13}C NMR (CD_3OD, 75 MHz) of compound **9b**

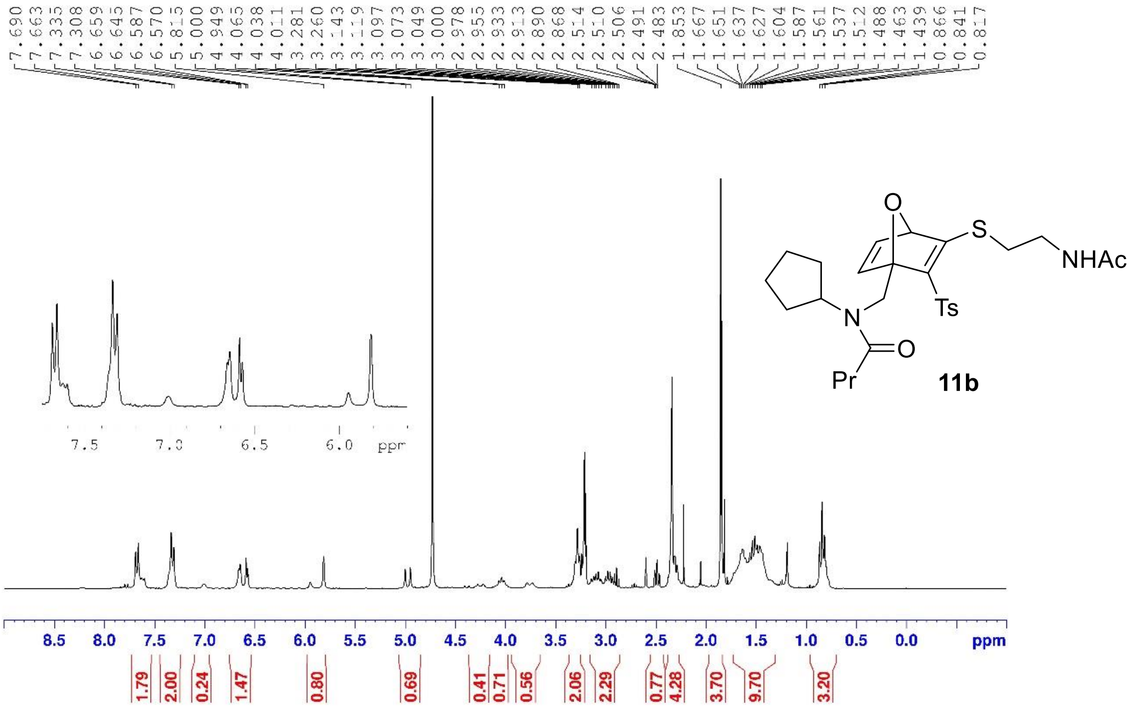

^{1}H NMR (CD_3OD, 300 MHz) of compound **11b**

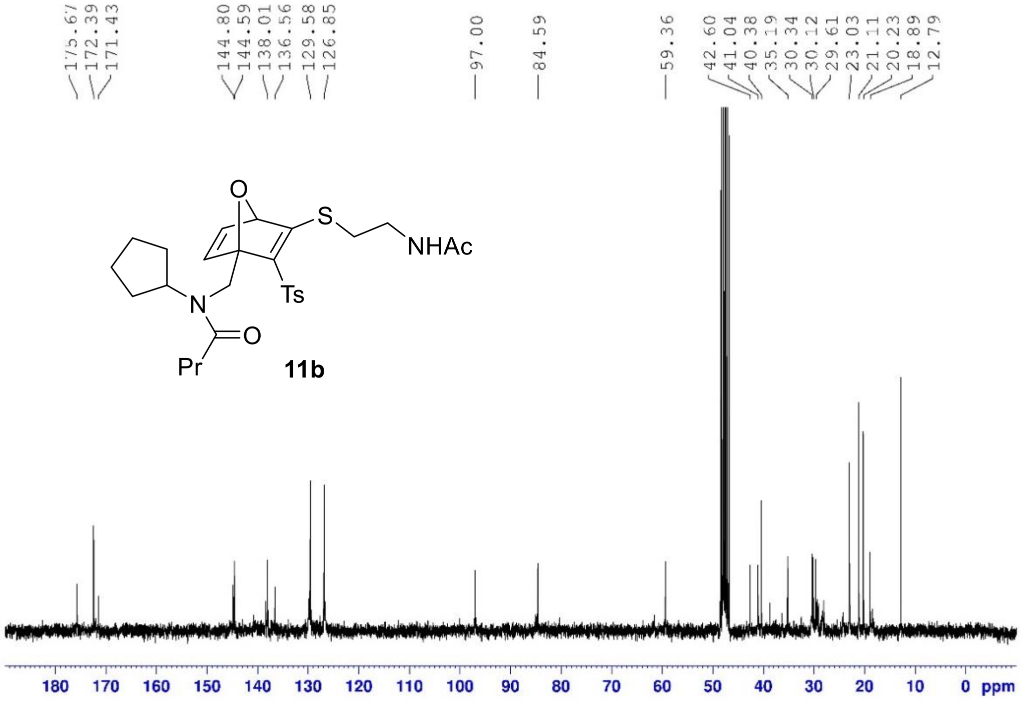

^{13}C NMR (CD_3OD, 75 MHz) of compound **11b**

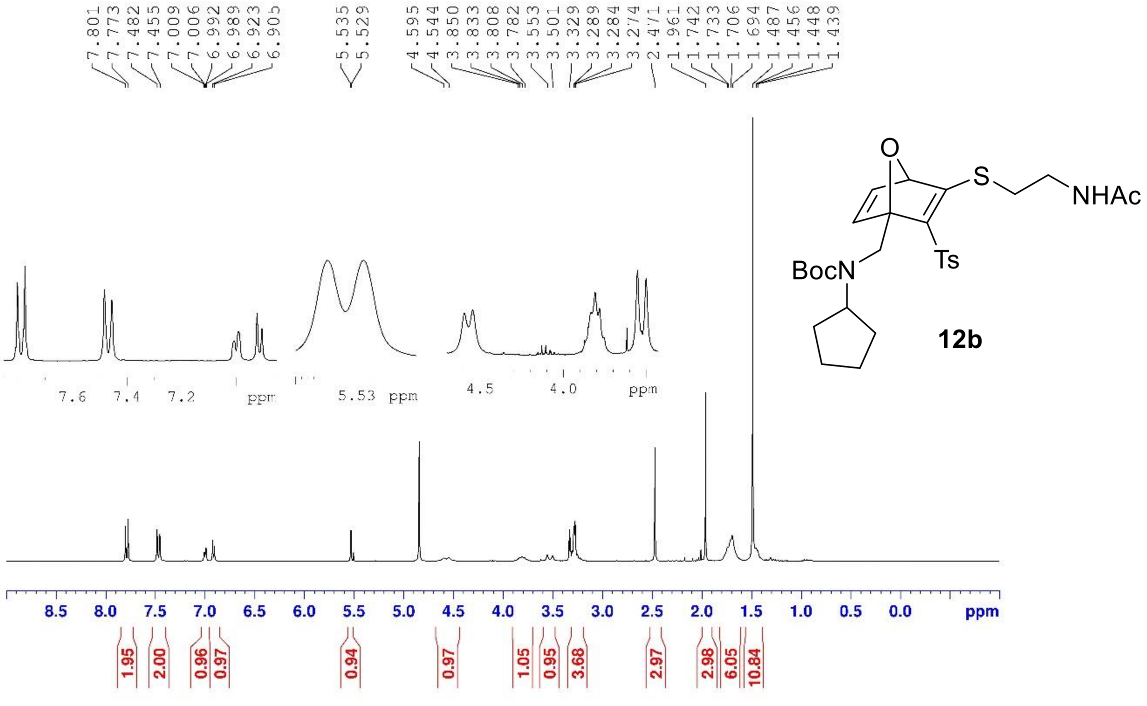

^{1}H NMR (CD_3OD, 300 MHz) of compound **12b**

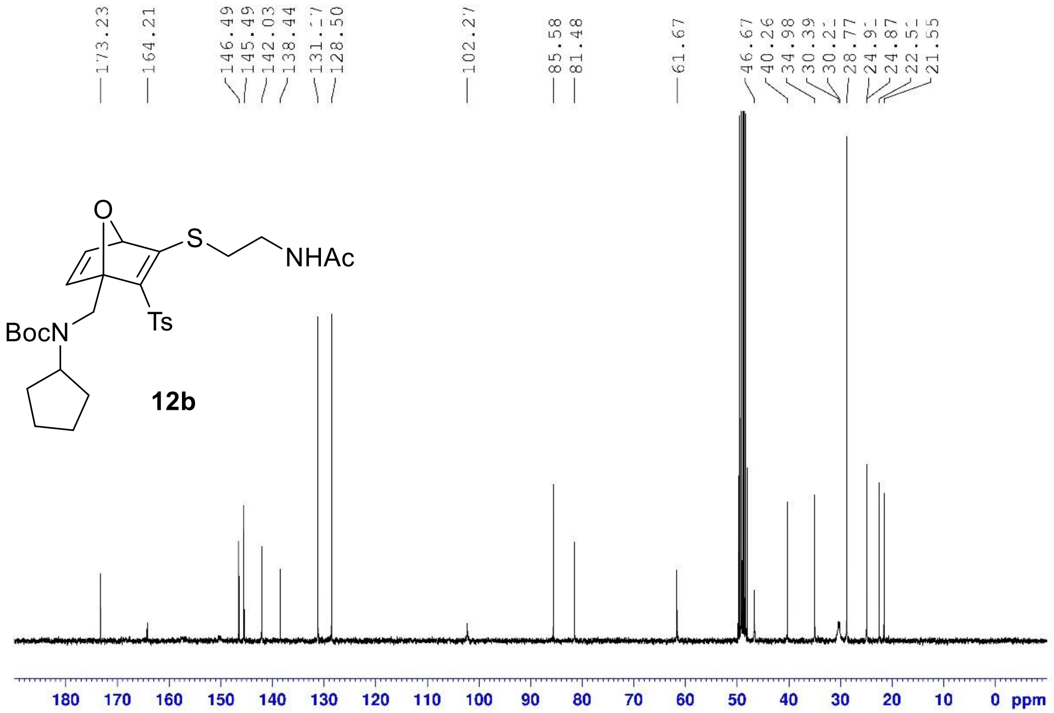

^{13}C NMR (CD_3OD, 75 MHz) of compound **12b**

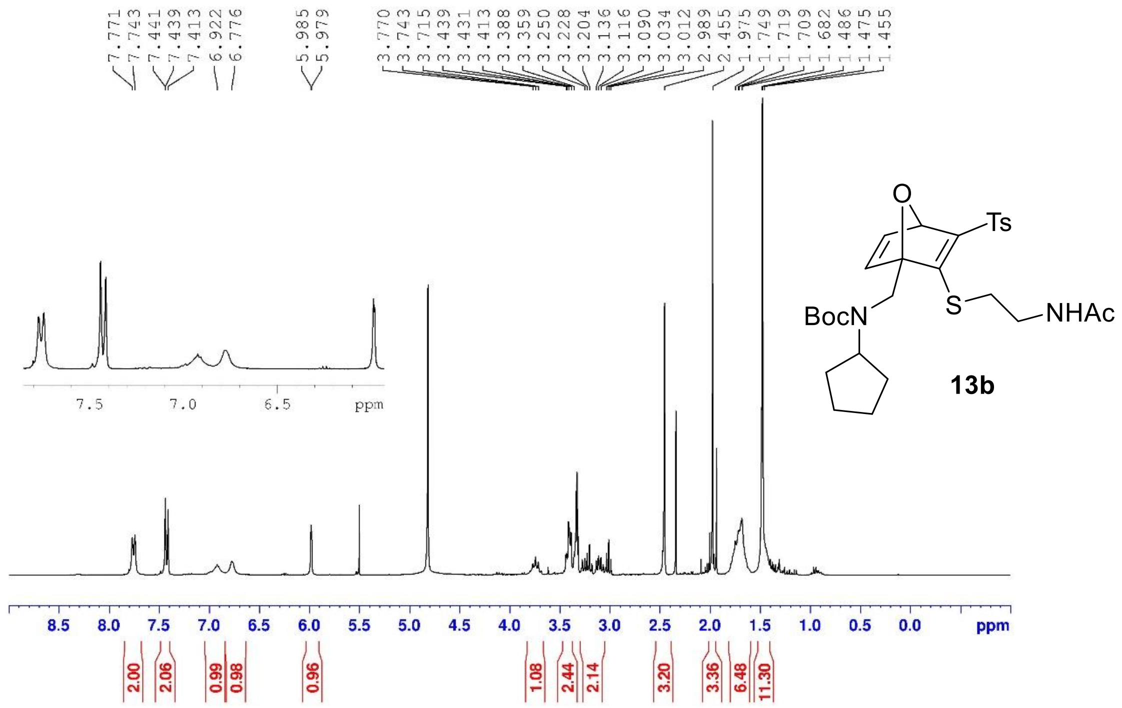

^{1}H NMR (CD_3OD, 300 MHz) of compound **13b**

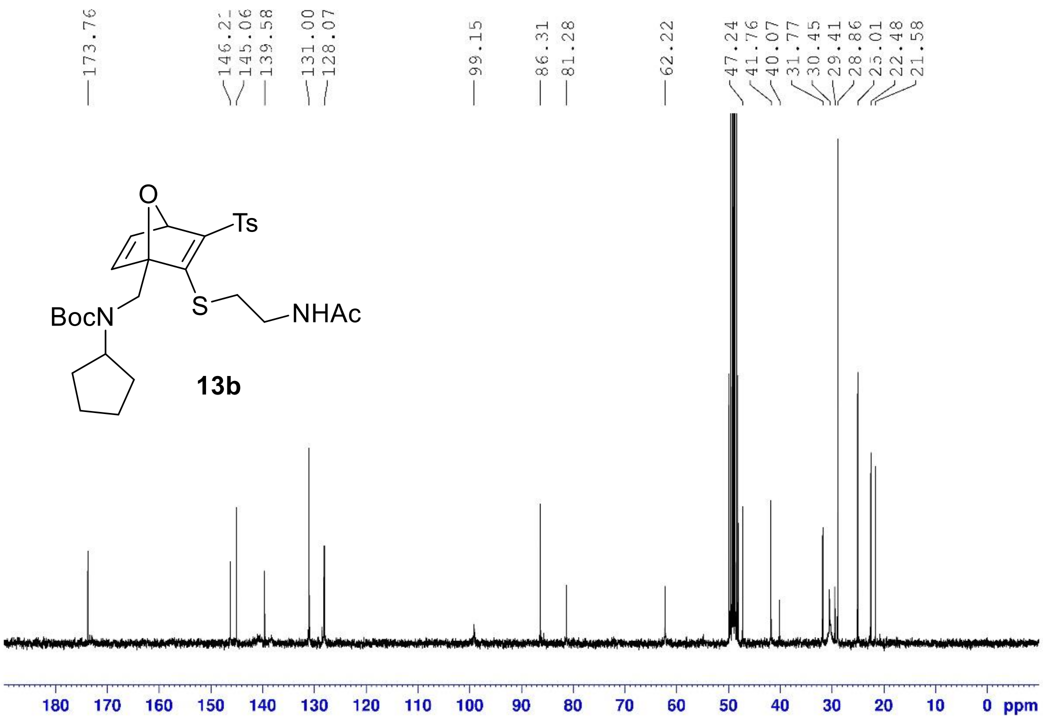

^{13}C NMR (CD$_3$OD, 75 MHz) of compound **13b**

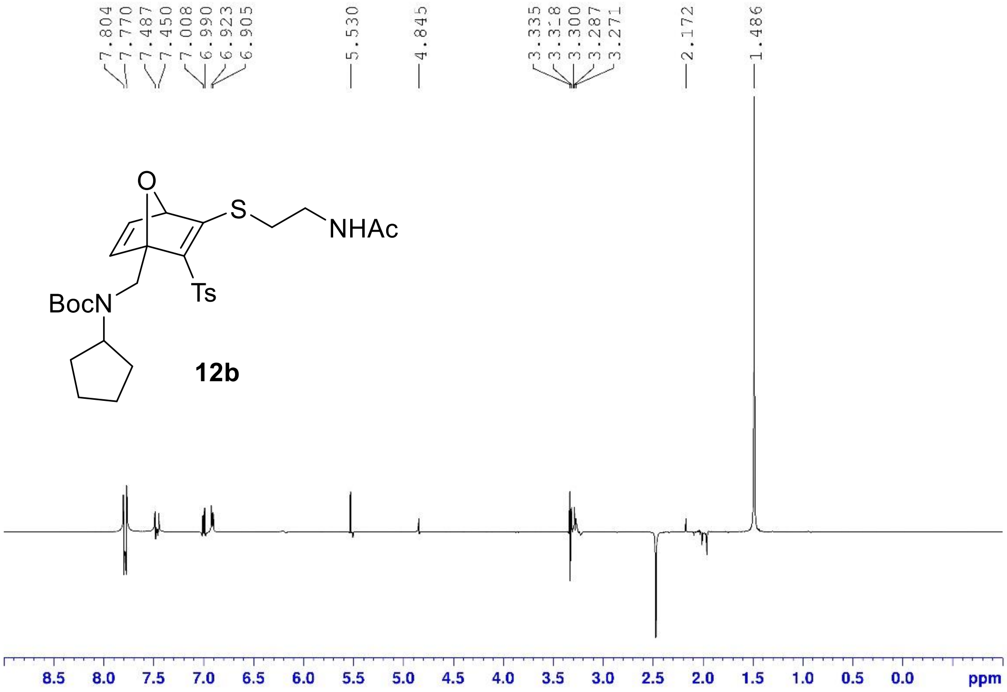

1D NOE (CD$_3$OD, 300 MHz) of compound **12b**

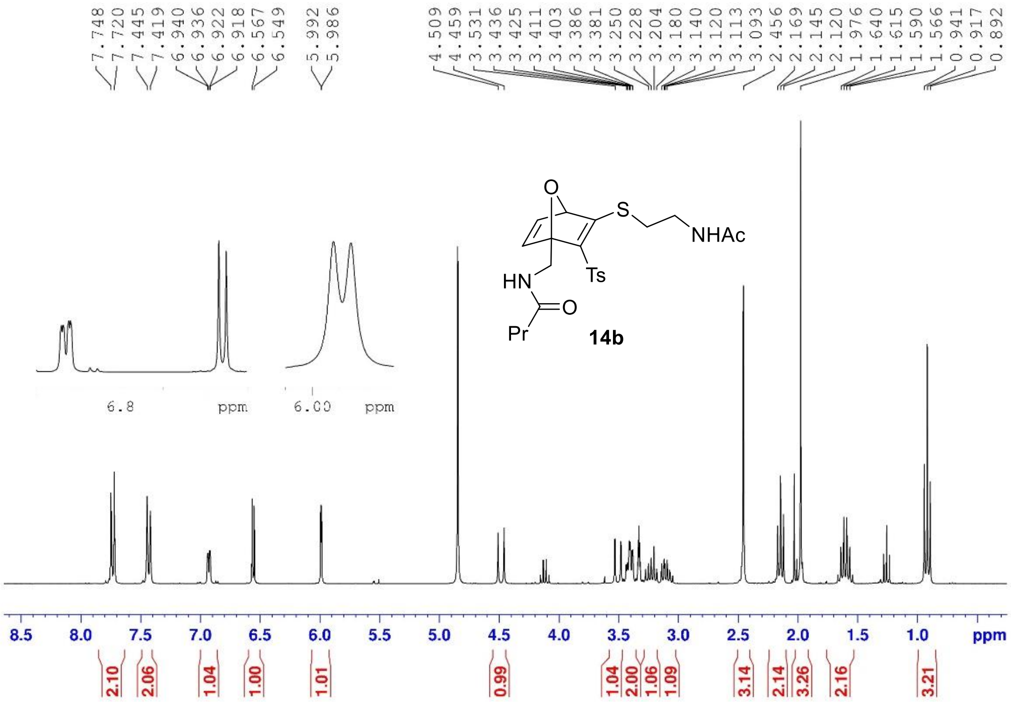

^{1}H NMR (CD_3OD, 300 MHz) of compound **14b**

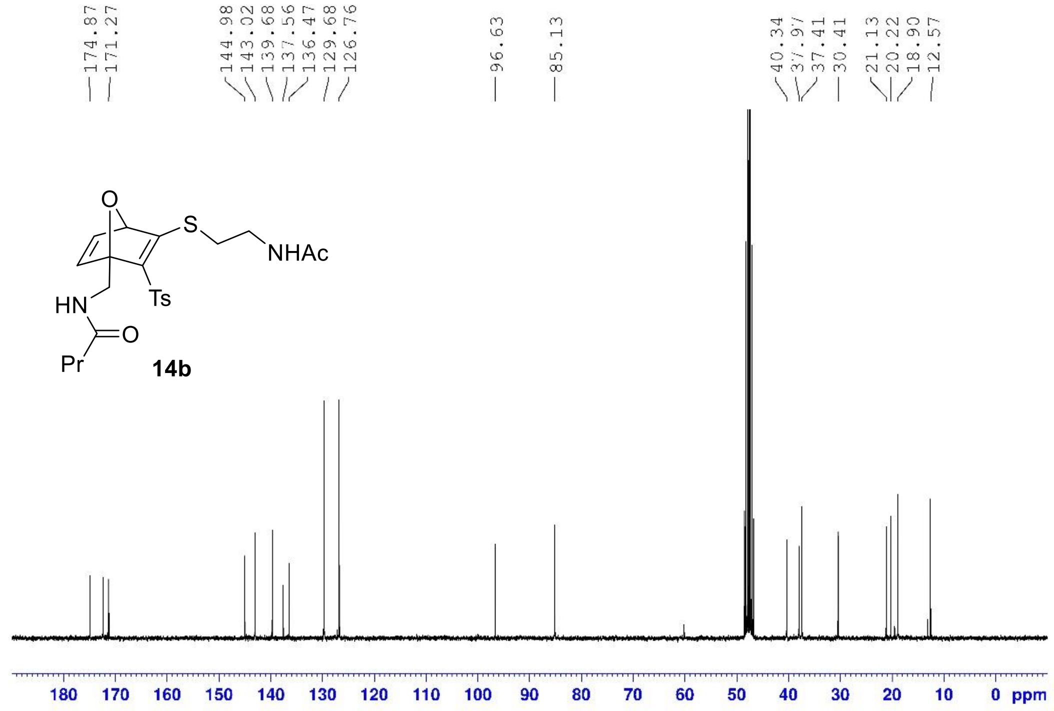

^{13}C NMR (CD_3OD, 75 MHz) of compound **14b**

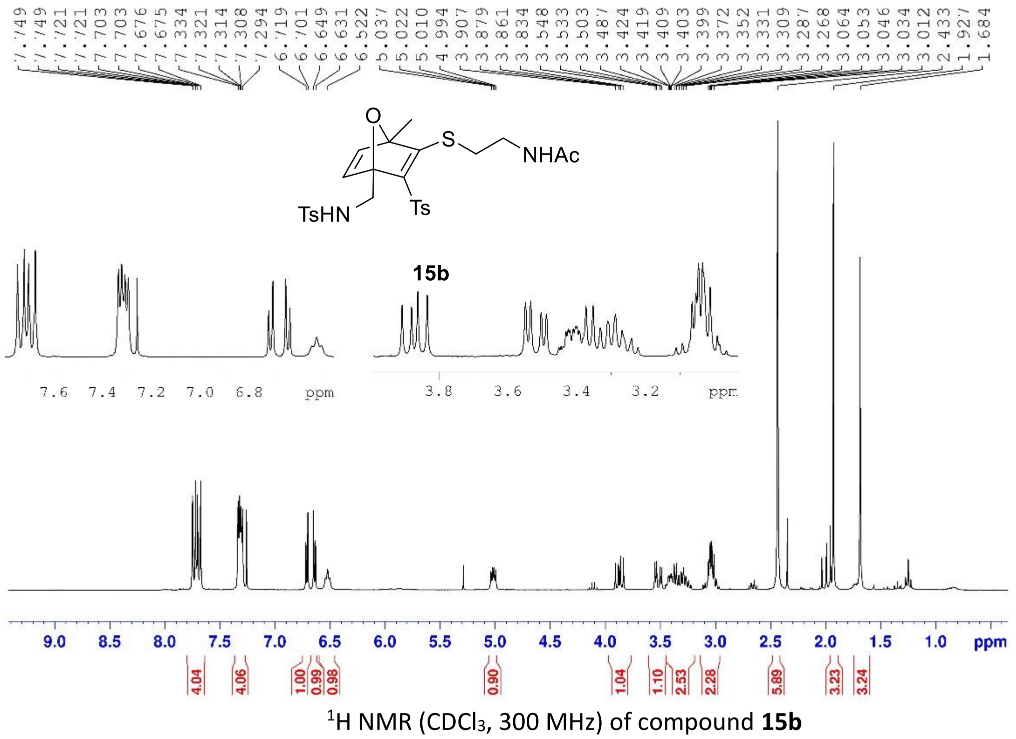

^{1}H NMR ($CDCl_3$, 300 MHz) of compound **15b**

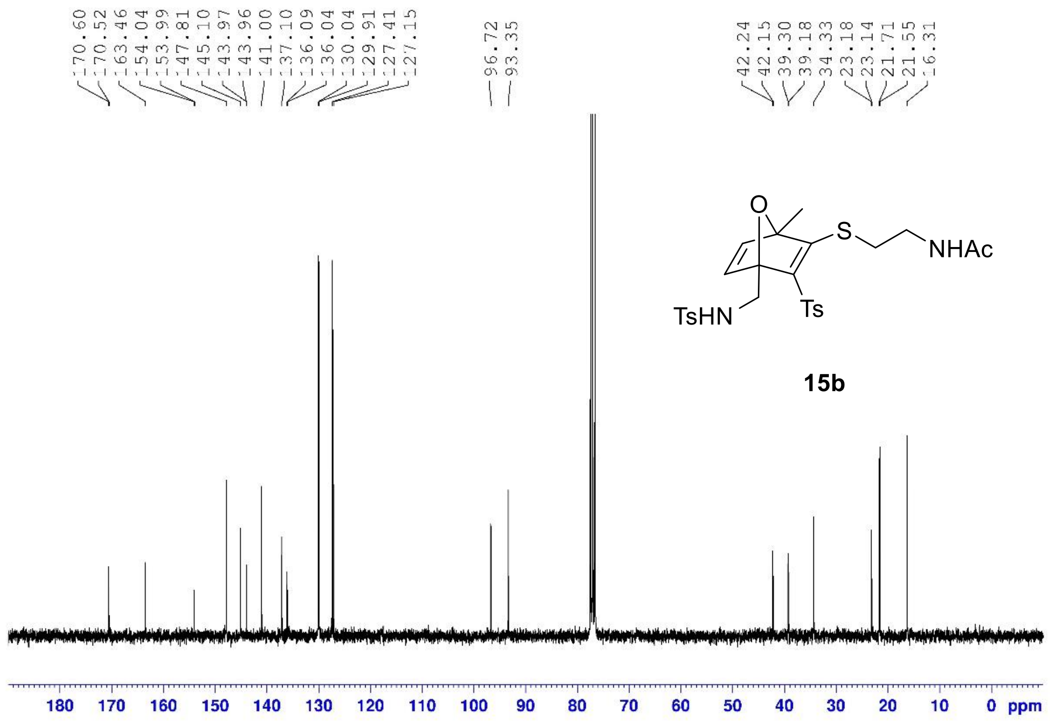

^{13}C NMR ($CDCl_3$, 75 MHz) of compound **15b**

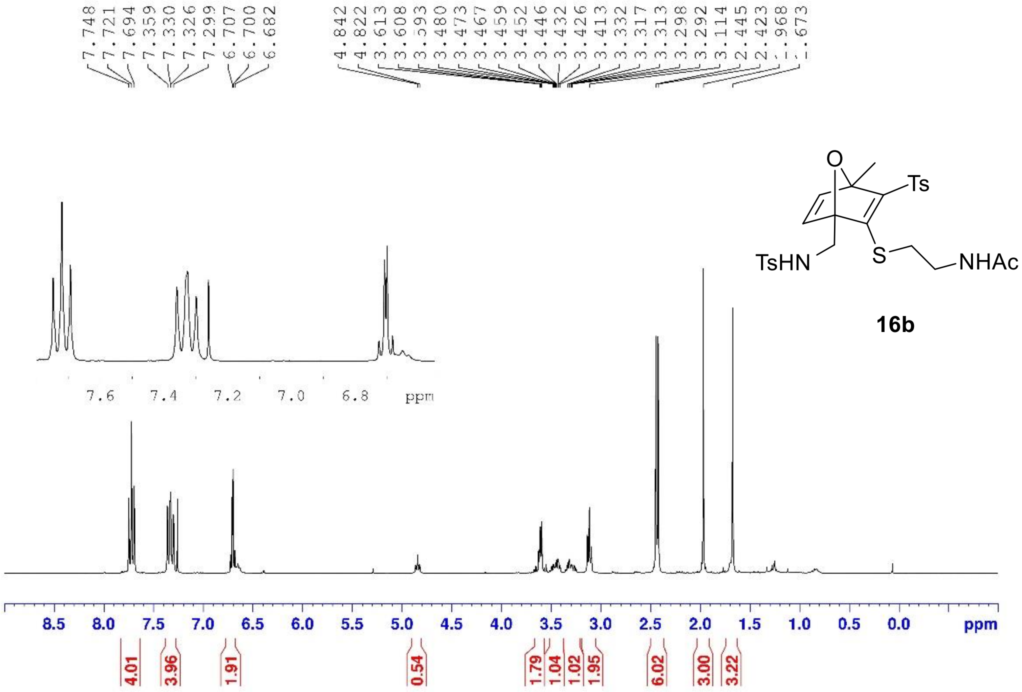

^{1}H NMR ($CDCl_3$, 300 MHz) of compound **16b**

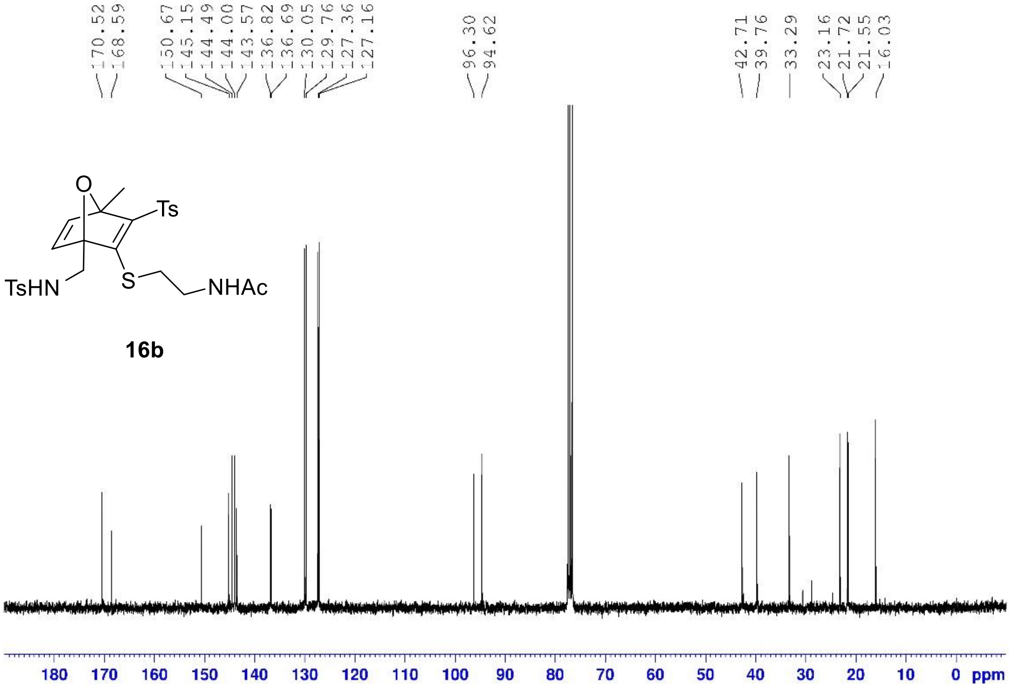

^{13}C NMR ($CDCl_3$, 75 MHz) of compound **16b**

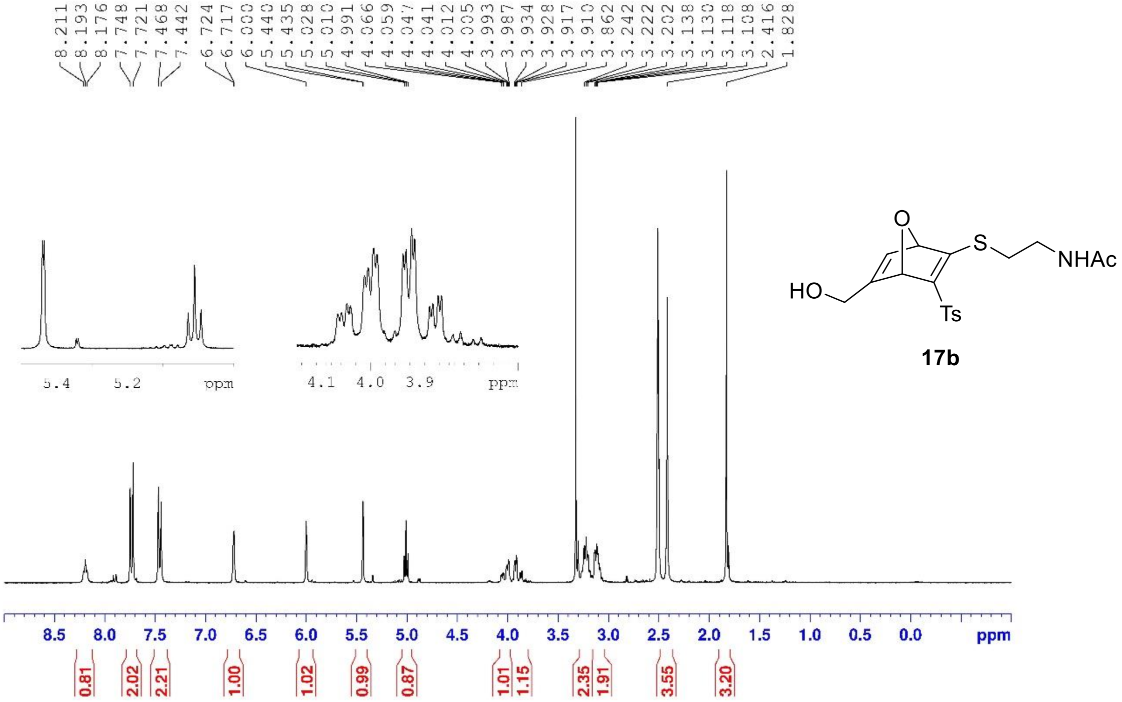

^{1}H NMR (DMSO-d_6, 300 MHz) of compound **17b**

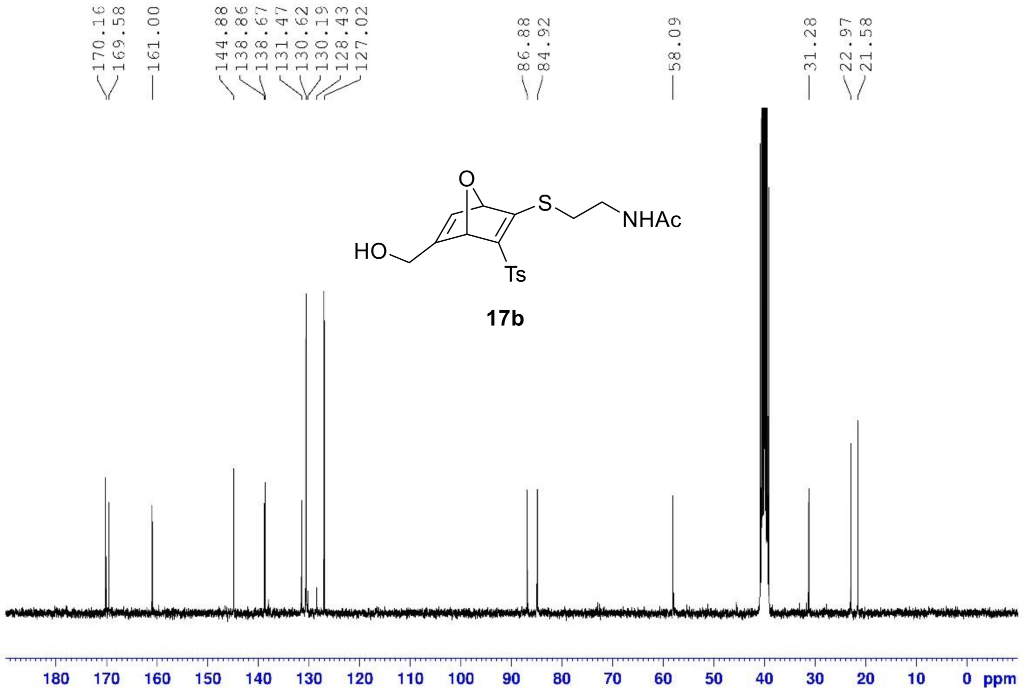

^{13}C NMR (DMSO-d_6, 75 MHz) of compound **17b**

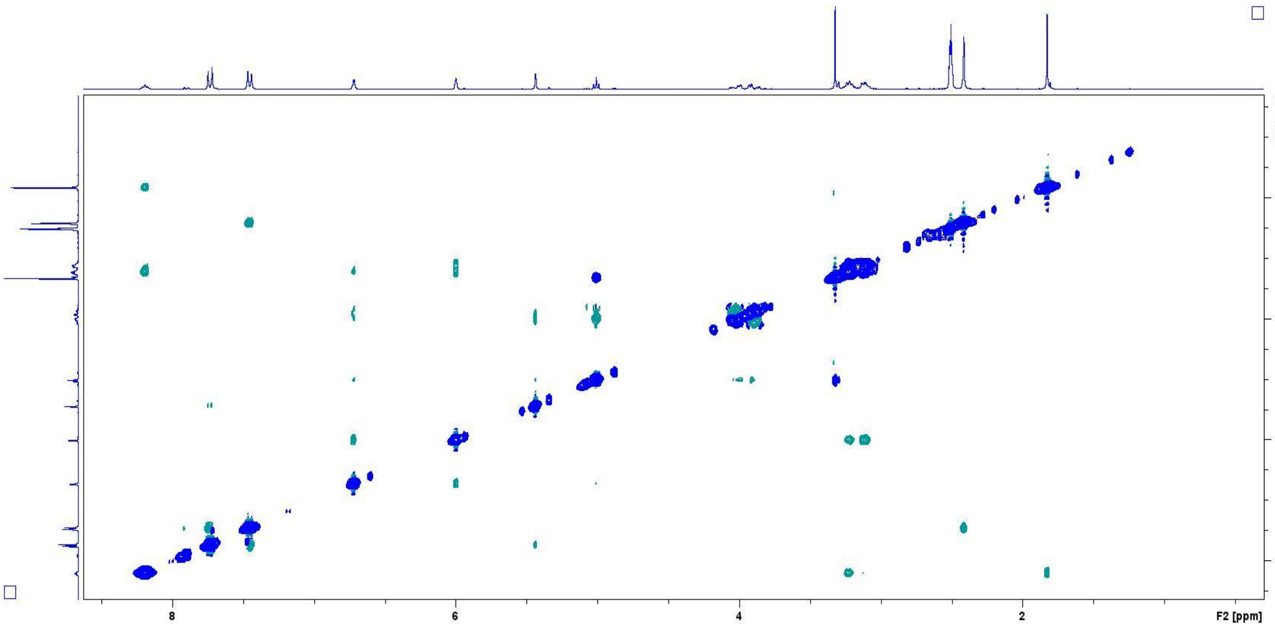

NOESY (DMSO-d_6, 300 MHz) of compound **17b**

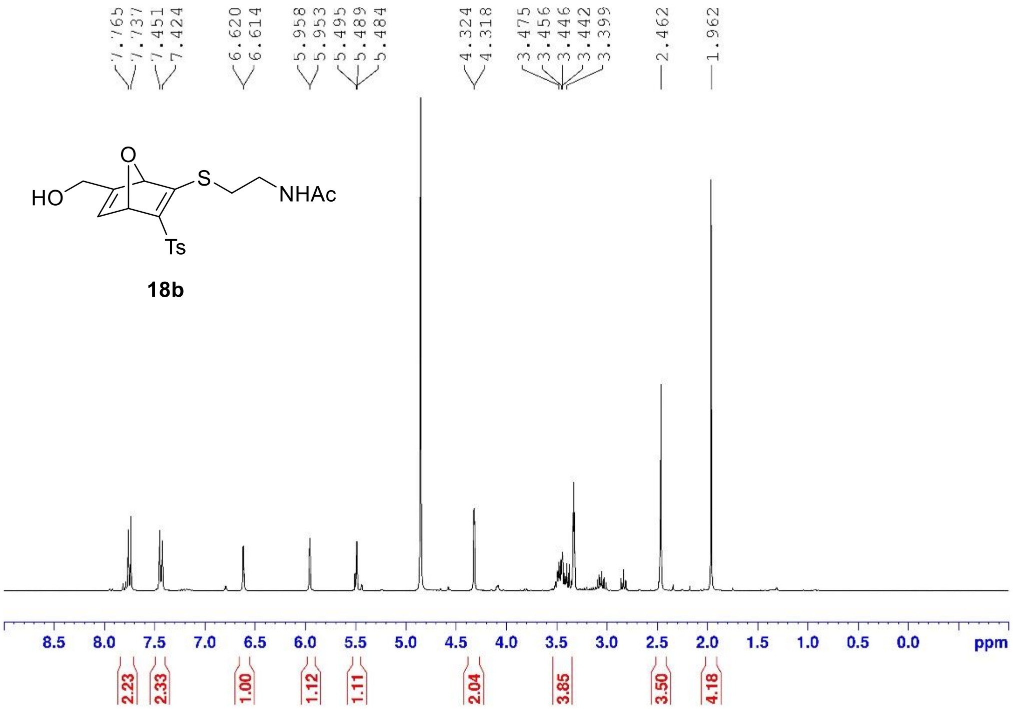

^{1}H NMR (CD_3OD, 300 MHz) of compound **18b**

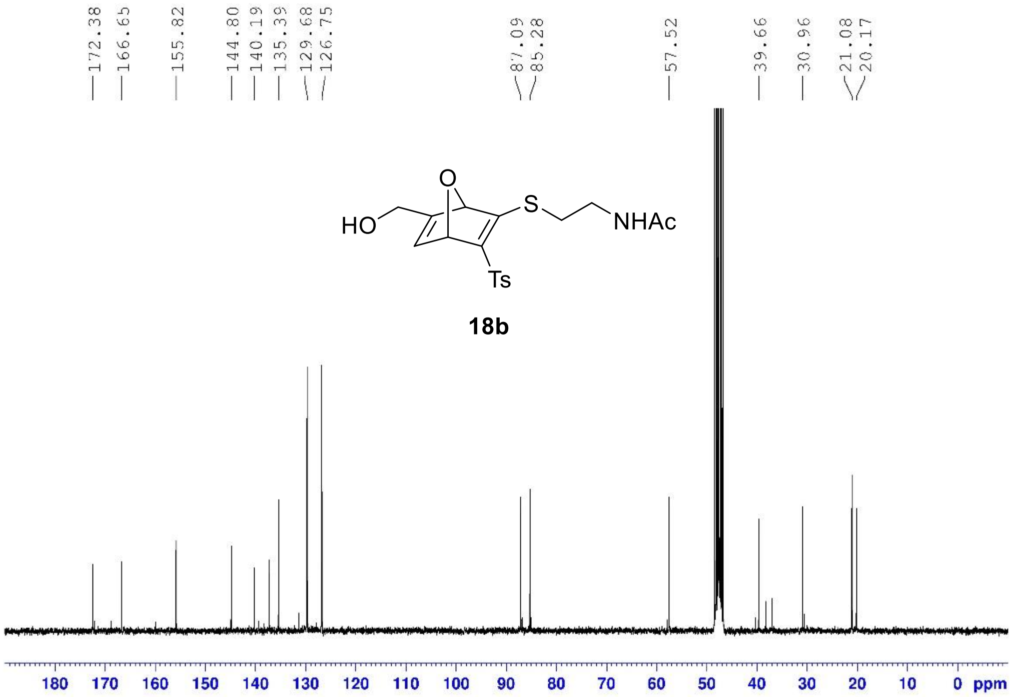

^{13}C NMR (CD_3OD, 75 MHz) of compound **18b**

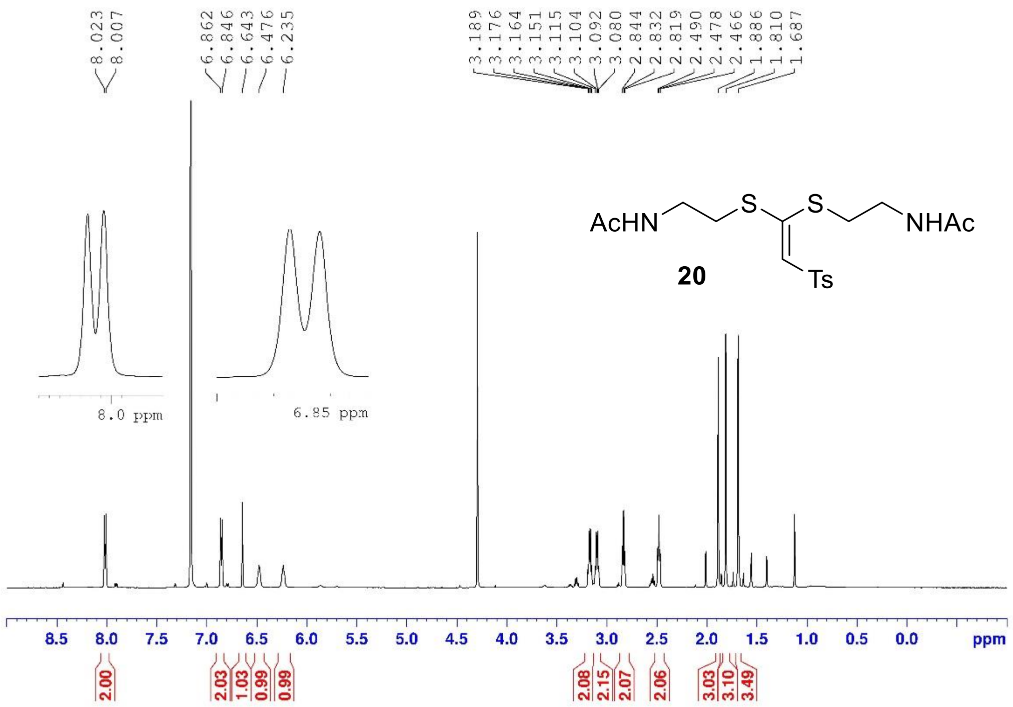

^{1}H NMR (C_6D_6, 500 MHz) of compound **20**

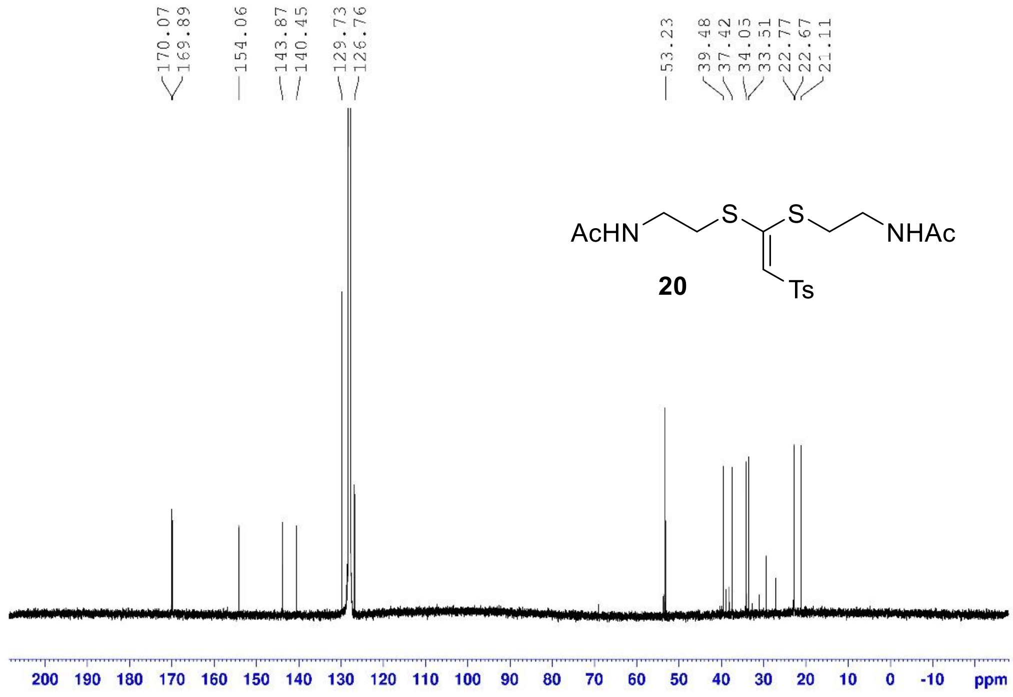

^{13}C NMR (C_6D_6, 125 MHz) of compound **20**

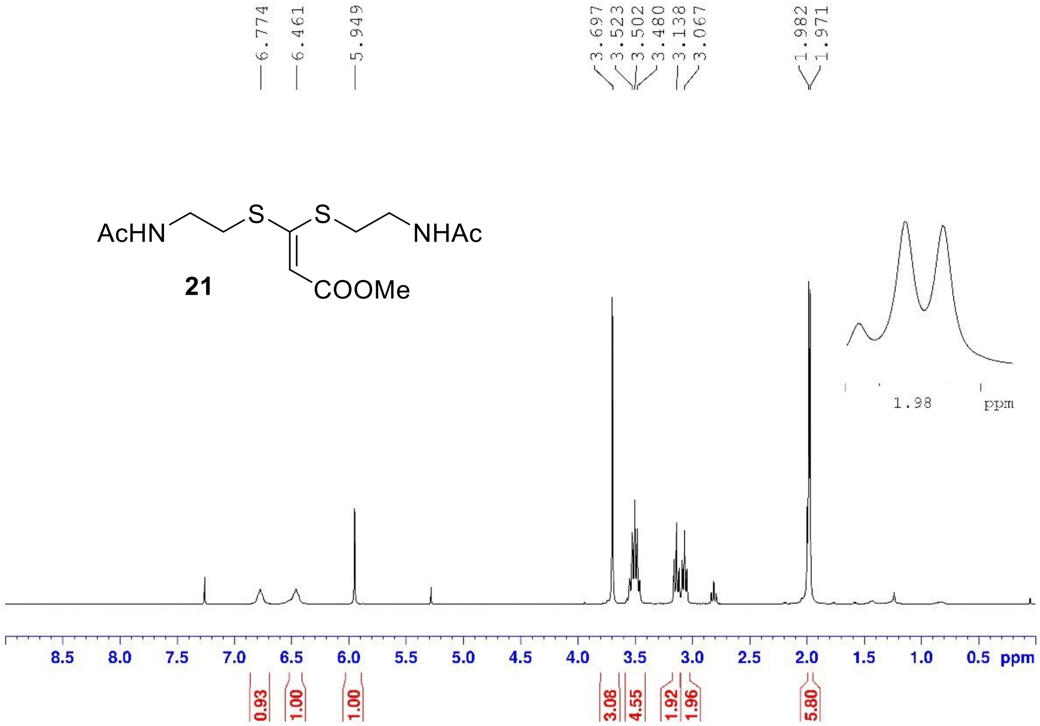

^{1}H NMR ($CDCl_3$, 300 MHz) of compound **21**

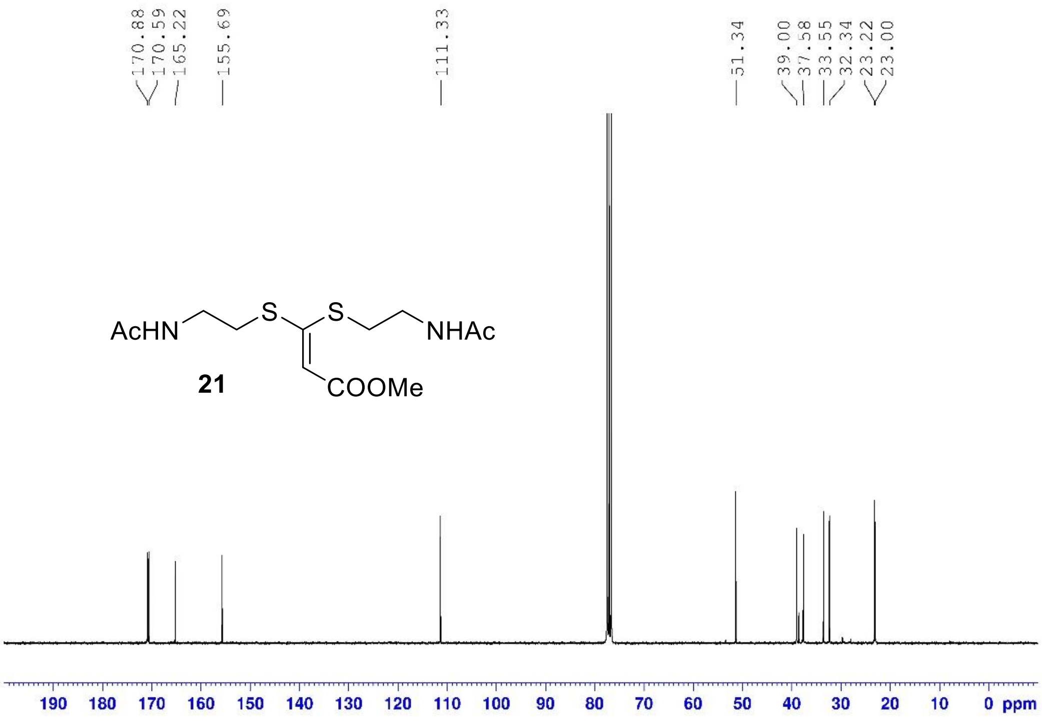

^{13}C NMR ($CDCl_3$, 75 MHz) of compound **21**

9. References

1. Zhang, C.; Ballay II, C. J.; Trudell, M. L. 2-Bromoethynyl Aryl Sulfones as Versatile Dienophiles: a Formal Synthesis of Epibatidine. *J. Chem. Soc., Perkin Trans. 1*, **1999**, 675−676.
2. Leroy, J. *Synth. Commun*. **1992**, *22*, 567.
3. Poulsen, T. B.; Bernardi, L.; Alemán, J.; Overgaard, J.; Jørgensen, K. A. Organocatalytic Asymmetric Direct α-Alkynylation of Cyclic β-Ketoesters. *J. Am. Chem. Soc*. **2007**, *129*, 441−449.
4. Gil de Montes, E.; Martínez-Bailén, M.; Carmona, A. T.; Robina, I.; Moreno-Vargas, A. J. Regioselectivity of the 1,3-Dipolar Cycloaddition of Organic Azides to 7-Heteronorbornadienes. Synthesis of β-Substituted Furans/Pyrroles. *J. Org. Chem*. **2020**, *85*, 8923-8932.
5. (a) Wanat, P.; Walczak, S.; Wojtczak, B. A.; Nowakowska, M.; Jemielity, J.; Kowalska, J. Ethynyl, 2-Propynyl, and 3-Butynyl C-Phosphonate Analogues of Nucleoside Di- and Triphosphates: Synthesis and Reactivity in CuAAC. Org. Lett., 2015, 17, 3062). (b) Oakdale, J. S.; Sit, R. K.; Fokin, V. V. Ruthenium-Catalyzed Cycloadditions of 1-Haloalkynes with Nitrile Oxides and Organic Azides: Synthesis of 4-Haloisoxazoles and 5-Halotriazoles. *Chem. Eur. J.* **2014**, *20*, 11101.
6. Stein, P. M.; Rudolph, M.; Hashmi, A. S. K. Water Can Accelerate Homogeneous Gold Catalysis. *Adv. Synth. Catal*. **2021**, *363*, 4264–42.
7. García-Domínguez, J.; Carranza, M.; Jansons, E.; Carmona, A. T.; Robina, I.; Moreno-Vargas, A. J. Transferring Substituents from Alkynes to Furans and Pyrroles through Heteronorbornadienes as Intermediates: Synthesis of β-Substituted Pyrroles/Furans. *J. Org. Chem.,* DOI: 10.1021/acs.joc.3c01145.
8. Xu, P., Chen, D.-S., Xi, J. and Yao, Z. Short Protecting Group-free Syntheses of Camptothecin and 10-Hydroxycamptothecin Using Cascade Methodologies. *Chem. Asian J.* **2015**, *10*, 976-981.
9. (a) For the preparation of halo-OND **1a**, see: Moreno-Clavijo, E.; Moreno-Vargas, A. J.; Kieffer, R.; Sigstam, T.; Carmona, A. T.; Robina, I. Exploiting the Ring Strain in Bicyclo[2.2.1]heptane Systems for the Stereoselective Preparation of Highly Functionalized Cyclopentene, Dihydrofuran, Pyrroline, and Pyrrolidine Scaffolds. *Org. Lett*., **2011**, *13*, 6244-6247. (b) For the preparation of halo-OND **2a**, see: Leroy, J. Diels-Alder reaction of furan with methyl 3-bromopropiolate: a route to methyl 3-oxo-7-oxabicyclo[2.2.1]hept-5-en-2-carboxylate *Tetrahedron Lett*. **1992**, *33*, 2969-2972. (c) For the preparation of halo-OND **4** and **5**, see reference [4]. (d) For the preparation of halo-OND **3a** and **10a**, see reference [7].
10. Gaussian 16, Revision C.01, Frisch, M. J.; Trucks, G. W.; Schlegel, H. B.; Scuseria, G. E.; Robb, M. A.; Cheeseman, J. R.; Scalmani, G.; Barone, V.; Petersson, G. A.; Nakatsuji, H.; Li, X.; Caricato,

M.; Marenich, A. V.; Bloino, J.; Janesko, B. G.; Gomperts, R.; Mennucci, B.; Hratchian, H. P.; Ortiz, J. V.; Izmaylov, A. F.; Sonnenberg, J. L.; Williams-Young, D.; Ding, F.; Lipparini, F.; Egidi, F.; Goings, J.; Peng, B.; Petrone, A.; Henderson, T.; Ranasinghe, D.; Zakrzewski, V. G.; Gao, J.; Rega, N.; Zheng, G.; Liang, W.; Hada, M.; Ehara, M.; Toyota, K.; Fukuda, R.; Hasegawa, J.; Ishida, M.; Nakajima, T.; Honda, Y.; Kitao, O.; Nakai, H.; Vreven, T.; Throssell, K.; Montgomery, J. A., Jr.; Peralta, J. E.; Ogliaro, F.; Bearpark, M. J.; Heyd, J. J.; Brothers, E. N.; Kudin, K. N.; Staroverov, V. N.; Keith, T. A.; Kobayashi, R.; Normand, J.; Raghavachari, K.; Rendell, A. P.; Burant, J. C.; Iyengar, S. S.; Tomasi, J.; Cossi, M.; Millam, J. M.; Klene, M.; Adamo, C.; Cammi, R.; Ochterski, J. W.; Martin, R. L.; Morokuma, K.; Farkas, O.; Foresman, J. B.; Fox, D. J. Gaussian, Inc., Wallingford CT, 2016.

11. Zhao, Y.; Truhlar, D. G. The M06 suite of density functionals for main group thermochemistry, thermochemical kinetics, noncovalent interactions, excited states, and transition elements: two new functionals and systematic testing of four M06-class functionals and 12 other functionals. *Theor. Chem. Acc.* **2008**, *120*, 215–241.
12. Scalmani, G.; Frisch, M. J. Continuous surface charge polarizable continuum models of solvation. I. General formalism. *J. Chem. Phys.* **2010**, *132*, 114110.
13. (a) Hratchian, H. P.; Schlegel, H. B. Accurate reaction paths using a Hessian based predictor-corrector integrator. *J. Chem. Phys.* **2004**, *120*, 9918–9924. (b) Hratchian, H. P.; Schlegel, H. B. Using Hessian updating to increase the efficiency of a Hessian based predictor-corrector reaction path following method. *J. Chem. Theory Comput.* **2005**, *1*, 61–69.
14. Ess, D. H.; Houk, K. N. Distortion/Interaction Energy Control of 1,3-Dipolar Cycloaddition Reactivity. *J. Am. Chem. Soc.* **2007**, *129*, 10646–10647.
15. Ess, D. H.; Houk, K. N. Theory of 1,3-Dipolar Cycloadditions: Distortion/Interaction and Frontier Molecular Orbital Models. *J. Am. Chem. Soc.* **2008**, *130*, 10187–10198.
16. van Zeist, W.-J.; Bickelhaupt, F. M. The activation strain model of chemical reactivity. *Org. Biomol. Chem.* **2010**, *8*, 3118–3127.
17. Fell, J. S.; Martin, B. N.; Houk, K. N. Origins of the Unfavorable Activation and Reaction Energies of 1-Azadiene Heterocycles Compared to 2-Azadiene Heterocycles in Diels–Alder Reactions. *J. Org. Chem.* **2017**, *82*, 1912–1919.
